# Direct Dehydrogenative Access to Unsymmetrical Phenones

**DOI:** 10.1002/anie.202201142

**Published:** 2022-03-19

**Authors:** Congjun Yu, Raolin Huang, Frederic W. Patureau

**Affiliations:** ^1^ Institute of Organic Chemistry RWTH Aachen University Landoltweg 1 52074 Aachen Germany

**Keywords:** Arylation, C−H Functionalization, Cross Dehydrogenative Coupling, Electrochemistry, Hexafluoroisopropanol

## Abstract

The first non‐directed dehydrogenative phenone coupling method of methylarenes with aromatic C−H bonds, displaying a large substrate scope, is herein reported. This reaction represents a far more direct atom‐ and step‐efficient alternative to the classical Friedel–Crafts or Suzuki–Miyaura derived acylation reactions. The method can be carried out on a gram scale and was successfully applied to the synthesis of several Ketoprofen drug analogues.

## Graphical abstract

The Friedel–Crafts reaction is an emblematic method for constructing C−C bonds at aromatic positions. Rendering this reaction dehydrogenative on a broad scope of substrates under simple reaction conditions constitutes an important milestone for synthetic chemistry.

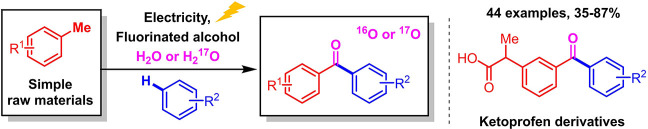

Toluene is one of the simplest raw materials as it occurs naturally in crude oil and is a byproduct in the production of gasoline and coke.^[1]^ The annual global production of toluene is approximately 30 million metric tons.^[2]^ Therefore, toluene as well as its derivatives (methylarenes) are ideal raw materials for organic synthesis. Their most common use involves oxidation towards carboxylic acids, aldehydes, esters, and related essential structures.^[3]^ However, their oxidative cross‐coupling with a *different* arene to furnish highly functionalized, non‐symmetrical hetero‐combined benzophenones is considerably more challenging. Such a method would be desirable in view of the high value of some the products (Scheme 1a–d). Indeed, unsymmetrical biaryl ketones are important motifs in organic chemistry, and are traditionally constructed by Friedel–Crafts reactions, through high‐energy acylating reagents.^[4]^ Nevertheless, there are intrinsic limitations associated to Friedel–Crafts chemistry, for example the use of very corrosive reagents such as SOCl_2_ or PCl_3_ in order to prepare the acylation reagents, or the requirement for strong Lewis acid catalysts. Those constraints led to severe functional group tolerance limitations (Scheme 1a). In the past few years, the development of transition‐metal catalyzed cross‐coupling reactions has provided alternative retrosynthetic strategies (Scheme 1b). In 1993, Suzuki notably reported the first palladium‐catalyzed carbonylative cross‐coupling of arylboronic acids with iodoarenes, an approach which was since considerably developed.^[5]^ Several other routes such as with organo‐zinc reagents,^[6]^ aryl halides^[7]^ organo‐tin and even organo‐indium nucleophiles^[8]^ were thereafter reported in combination with various leaving groups. Furthermore, the utilization of the C−H bond activation concept in the frame of carbonylative cross coupling has also been developed, both in intra‐^[9]^ and intermolecular fashion.^[10]^ Those methods still require however the use of metal additives as well as pre‐functionalization of the other coupling partner, usually with a halide, and are thus typically not dehydrogenative. The few dehydrogenative methods that do exist usually require structurally inconvenient directing groups (DG), onerous palladium salts as catalysts, and stoichiometric, non‐atom efficient chemical oxidants, usually peroxides (Scheme 1c).^[11]^ Clearly, the development of a general cross dehydrogenative coupling strategy^[12]^ for the synthesis of unsymmetrical benzophenones would be considerably more step and atom efficient compared to all the above mentioned approaches.[^1^, ^2^, ^3^, ^4^, ^5^, ^6^, ^7^, ^8^, ^9^, ^10^, ^11^]

**Scheme 1:**
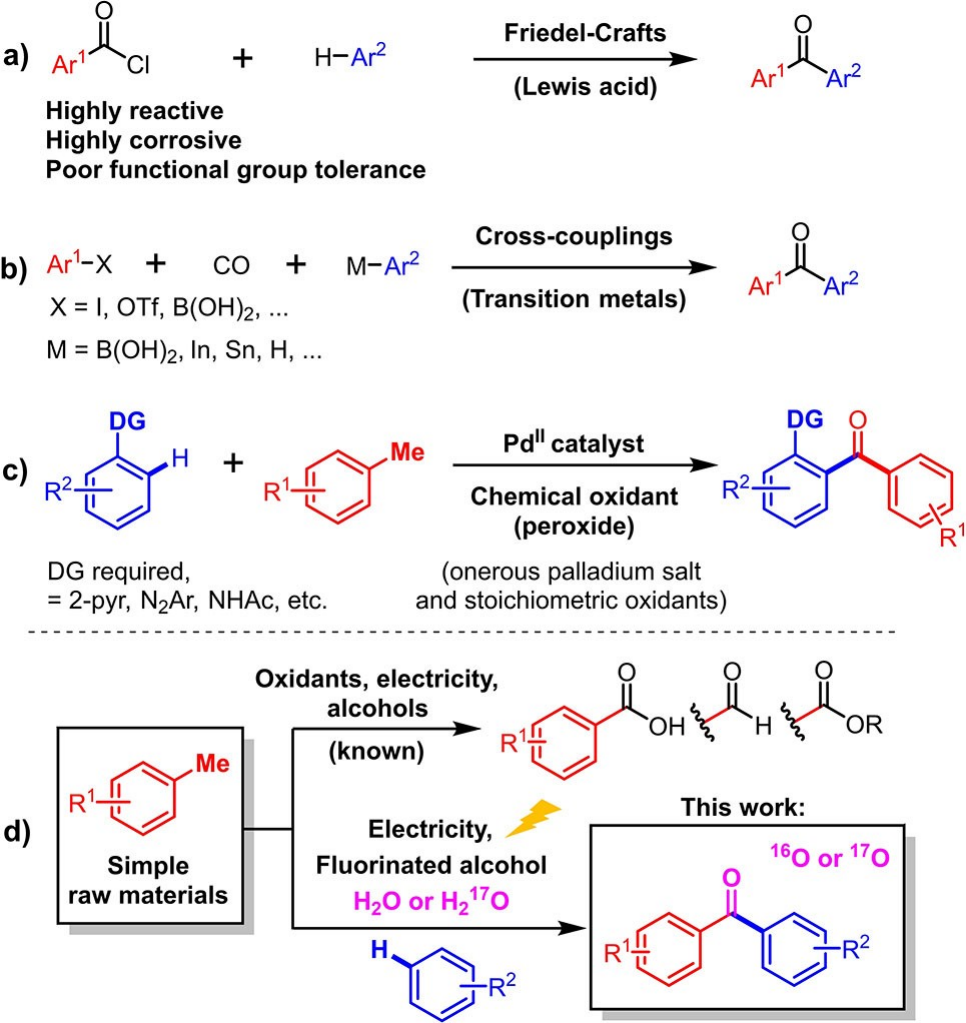
Methods to construct unsymmetrical biaryl ketones.

Meanwhile, electro‐oxidative synthetic methods are lately at the center of a regain of interest, due to their efficiency as well as to their suppressing the need for chemical oxidants.^[13]^ Inspired by some previous works regarding the oxidative esterification of methylarenes by electricity with alcohols,^[3h]^ we turned our attention to other important classes of nucleophiles such as simple arenes in order to directly access unsymmetrical diaryl ketones. Toluene (**a1**) and benzene (**b1**) were selected as the two simplest possible test substrates. Upon condition optimization, the electro‐oxidative intermolecular phenone coupling of toluene with an aromatic C−H bond of benzene was thus realized with an NMR yield of 88 % (81 % isolated yield, Table 1). In the optimal conditions, hexafluoro‐2‐propanol (HFIP) was selected as solvent, three equivalents of sodium dodecylbenzenesulfonate (mixture) were applied as the electrolyte and a small amount of water was added. The reaction was carried out in an undivided cell (graphite as the anode and aluminum as the cathode with a 10.0 mA current). Interestingly, 10 % (NMR yield) benzaldehyde was typically detected as the byproduct. In order to inhibit the homo‐coupling product and to improve the yield, a large amount of benzene (30 equivalents) was applied. However, a good yield (64 %) can still be achieved when this amount was lowered to 10 equivalents (Table 1, Entry 2). Fluorinated alcohols showed a unique ability as solvents to promote the reaction. No other non‐fluorinated alcohols tested were found operational (Table 1, Entries 3 and 4).^[14]^ Both lower and higher currents were found slightly detrimental to the yield (Table 1, Entry 5). The reaction still operates in water and oxygen‐free conditions or in the presence of a large excess of water, albeit with reduced yields (Table 1, Entry 6). The reaction is moreover not very sensitive towards the nature of the cathode (Table 1, Entry 7), in contrast to the nature of the anode. When the anode was changed to BDD (boron‐doped diamond), the reaction was significantly suppressed (Table 1, Entry 8). The electrolyte is also essential to the reaction. Sulfonates showed superior electrolytic ability (Table 1, Entry 9) while other electrolytes generally delivered lower yields (Table 1, Entry 10, see Supporting Information for a full list). The good performance of the sodium dodecylbenzenesulfonate electrolyte might arise from its hydrophobic anionic character, thus reducing water concentration in the electric double layer, in turn reducing unwanted trapping of the benzylic carbocation with H_2_O. When a lower amount of the electrolyte was applied, the reaction still occurred, albeit with a slightly reduced yield (Table 1, Entry 11). Noteworthy, 78 % of the electrolyte could be recovered at the end of the reaction (see Supporting Information).

**Table 1:** Optimization of the reaction conditions.^[a]^

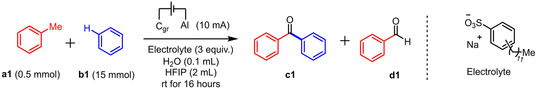			
Entry	Deviation from standard conditions	Yield of **c1**	Yield of **d1**
1	None	**88 %** ^[b]^	**10 %**
2	**b1** (5 mmol)	64 %	25 %
3	MeOH or Cl_3_CCH_2_OH as solvent	0 %	0 %^[14]^
4	F_3_CCH_2_OH as solvent	39 %	22 %
5	5 mA or 12.5 mA	70 % or 75 %	17 % or 15 %
6	No water, or 0.2 mL water	70 %,^[c]^ or 80 %	13 %, or 14 %
7	Ni plate, stainless steel plate, Pt plate as cathode	75 %, 74 %, 79 %	17 %, 16 %, 12 %
8	BDD as anode	5 %	6 %
9	NaOTs, *n*Bu_4_NOTs, *n*Bu_4_NOTf, *n*Bu_4_NOMs as electrolyte	65 %,72 %,45 %,54 %	17 %,10 %,21 %,20 %
10	*n*Bu_4_NBF_4_, *n*Bu_4_NClO_4_ as electrolyte	11 %, 12 %	3 %, 11 %
11	Electrolyte (2 equiv)	75 %	14 %

[a] An undivided cell was utilized. (anode: graphite: 52×8×2 mm, of which 20×8×2 mm immerged, cathode: aluminum, same dimensions, see Supporting Information). The yields were determined by ^1^H NMR analysis utilizing 1,3,5‐trimethoxybenzene as the internal standard (added at the end of the reaction). [b] The isolated yield under optimal conditions (Entry 1) is 81 %, Faradaic efficiency: 41 %. The NMR yield under N_2_ atm is 90 %. At 2 h reaction time, the yield was only 12 %. [c] Identical result under air or under N_2_ atm.

With the objective of elucidating the reaction mechanism, three possible intermediates were tested separately with the standard reaction conditions (Scheme 2a). Only diphenylmethane **e1** reacted under the standard conditions to give benzophenone product **c1** with an impressive quantitative yield (100 % NMR yield). The mesylated intermediate **f1** didn't give any further oxidation products. In contrast, intermediate **g1**, which could potentially arise from the reaction of toluene and HFIP, forms aldehyde **d1** (53 % NMR yield) under standard conditions. These results indicate that diarylmethane **e1**, or a derivative of it, is a very plausible reaction intermediate, in contrast to **f1** and **g1**. In addition, the byproduct benzaldehyde **d1** was also tested with the standard reaction conditions, but did not deliver any desired benzophenone‐coupling product either. Aldehyde **d1** can therefore be reasonably excluded as an intermediate or substrate for this reaction. In order to investigate the role of HFIP, different alcohols were tested for the product formation from both toluene **a1** and intermediate **e1**. Interestingly, non‐fluorinated alcohols such as methanol cannot convert toluene **a1** to the product **c1** but can convert intermediate **e1** to the product with an 8 % NMR yield. All yields are much higher in converting intermediate **e1** to the product than converting the starting material **a1** to the product, regardless of the applied fluorinated solvents (Scheme 2b). These results indicate that the fluorinated solvent HFIP is essential for the intermolecular C−C bond formation process. Thus, the high acidity and low nucleophilicity of fluorinated alcohols^[15]^ might facilitate the nucleophilic attack from arenes to the aromatic methyl cation, which is generated by anode oxidation.^[16]^ Interestingly, HFIP has also been known to modulate the nucleophilicity of substrates in the context of electro‐oxidative cross dehydrogenative coupling reactions.^[17]^ The most plausible mode of action here is that the strong H‐bond donor character of HFIP neutralizes unwanted polar nucleophiles such as water, thus favoring low polarity arene nucleophiles.^[18]^ Once intermediate diphenylmethane **e1** is formed, it is then further oxidized to the final product **c1**. When non‐fluorinated alcohols are utilized, the nucleophilic attack from the alcohols is faster than that of the arenes, yielding the expected acetals, aldehydes, or esters upon further oxidation (Scheme 2c).[^3h^, ^14^] Finally, in order to formally determine the oxygen source towards product **c1**, we performed the standard reaction (Table 1, Entry 1) with ^17^O‐labeled water (H_2_^17^O, ^17^O content: 35–40 %, Scheme 2d). As expected, the ^17^O‐labeled benzophenone product ^**17**^ **O‐c1** was isolated with 37 % ^17^O‐content, which is three orders of magnitude greater than natural ^17^O abundance (see Supporting Information). This thus corresponds to a label incorporation of at least 92 %. It can therefore be concluded that water is the main oxygen source in this reaction. Moreover, this makes the present synthetic method an excellent tool to readily furnish O‐labeled phenones. In the case of ^**17**^ **O‐c1**, a very strong ^17^O NMR signal was obtained with only 512 scans at +547 ppm versus the D_2_O internal standard (Scheme 2d, see Supporting Information), which is in line with literature values for acetone (+576 ppm), or acetophenone (+553 ppm).^[19]^

**Scheme 2:**
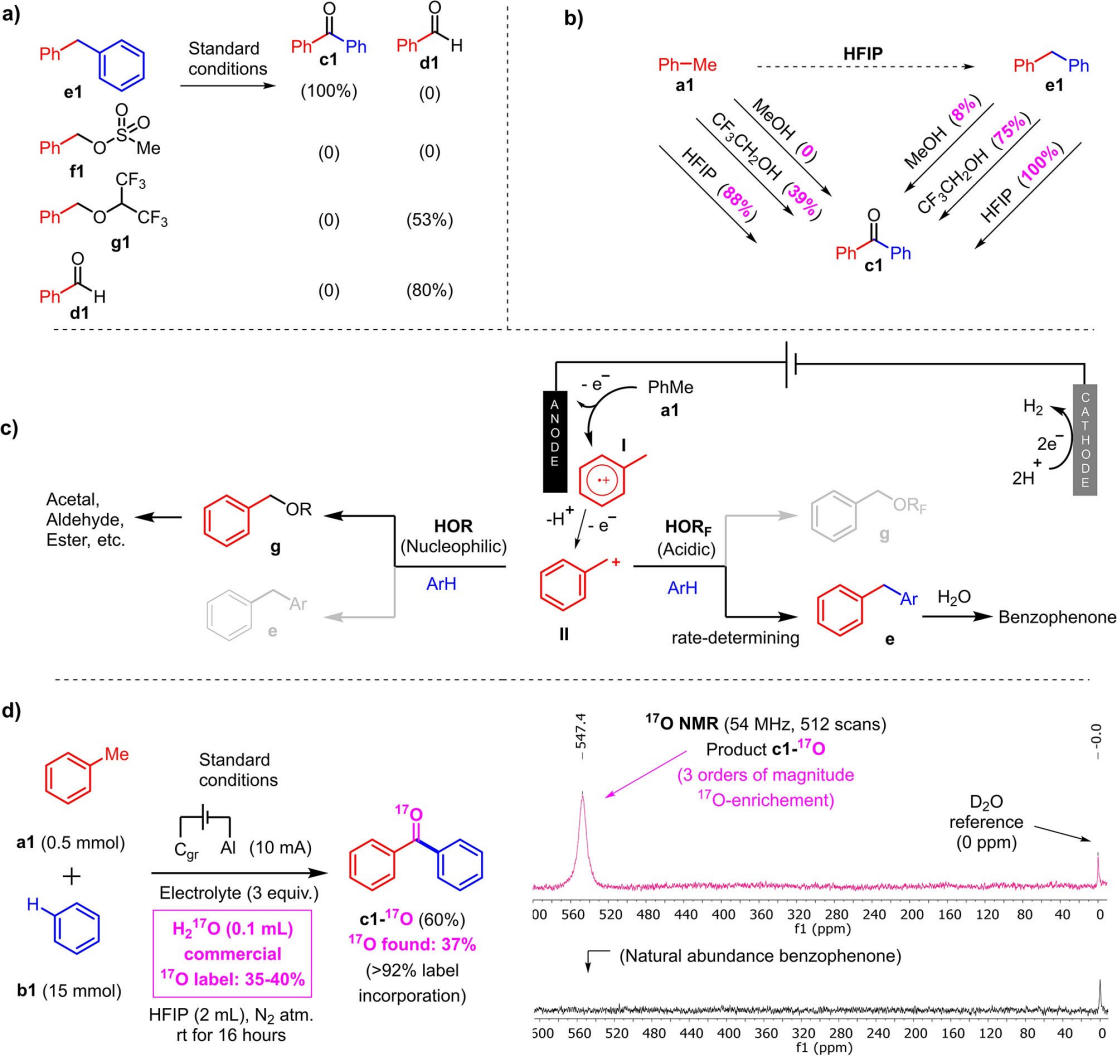
Mechanistic studies. a) Identification of the reaction intermediate. All the yields in the mechanistic studies were determined by ^1^H NMR analysis utilizing 1,3,5‐trimethoxybenzene as the internal standard. b) Solvents tests. c) Mechanism proposal. d) ^17^O‐labeling experiment, D_2_O standard (see Supporting Information).

The substrate scope was subsequently examined and found to be quite broad (**c1** to **c46**, Scheme 3), including sensitive functional groups such as for example other benzylic positions (**c9** to **c11**), a Michael acceptor (**c15**), and diverse bioactive heterocycles (see Supporting Information). A moderate Faradaic efficiency of 41 % was calculated for product **c1**,^[20]^ indicating the possible involvement of some yet unidentified side reactions.

**Scheme 3:**
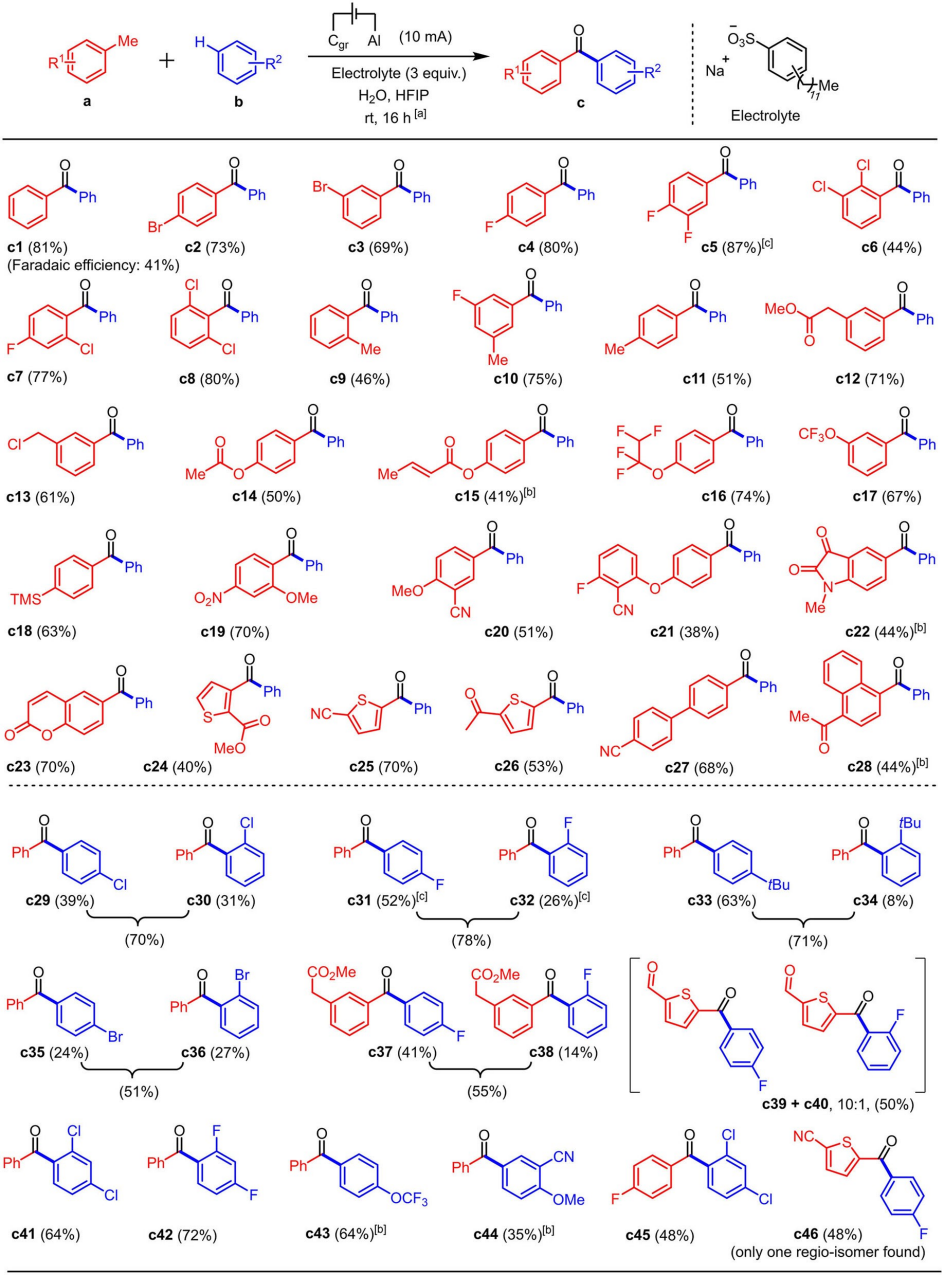
Electro‐oxidative phenone coupling of methylarenes with aromatic C−H bonds, isolated yields.^[a]^ [a] Standard conditions: **a** (0.5 mmol), **b** (15 mmol), electrolyte (1.5 mmol), Water (0.1 mL), and HFIP (2 mL) were applied. The reaction was carried out in an undivided cell (see pictures in the Supporting Information). Graphite was used as the anode (52×8×2 mm, of which 20×8×2 mm immersed, current density: *J*=24 A m^−2^) and aluminum was used as the cathode (same dimensions). The reaction was performed at room temperature and stirred (circa 400 rpm) for 16 hours. [b] 5 mA current was applied. [c] 9 mA current was applied. (Selected failed substrates: see Supporting Information).

In order to further demonstrate the applicability of this method, a large‐scale batch of phenone product **c1** was performed. When 1.0 gram of toluene was applied under standard conditions, the benzophenone product was obtained with a 52 % isolated yield. Because the electrode size was not enlarged for the large‐scale reaction, a longer reaction time (72 hours) was necessary (Scheme 4a, see Supporting Information). In view of the relatively simple reaction conditions, electrochemical flow reactors may be applicable to the herein described method in the future.^[21]^ Furthermore, this method was then applied to the synthesis of Ketoprofen, a nonsteroidal anti‐inflammatory drug with analgesic and antipyreptic properties,^[22]^ from simple commercially available starting materials. This represents a rare retrosynthetic cut, at the carbonyl group, for the synthesis of Ketoprofen and its derivatives. These are also associated to important or potential biological activity. Ketoprofen **c47** was obtained with a 66 % overall yield starting from **a47**. Two fluorinated and one trifluoromethoxylated Ketoprofen derivatives were likewise synthesized with encouraging yields (**c48** to **c50**). These molecules are related to numerous bioactivity indicators^[23]^ (Scheme 4b).

**Scheme 4:**
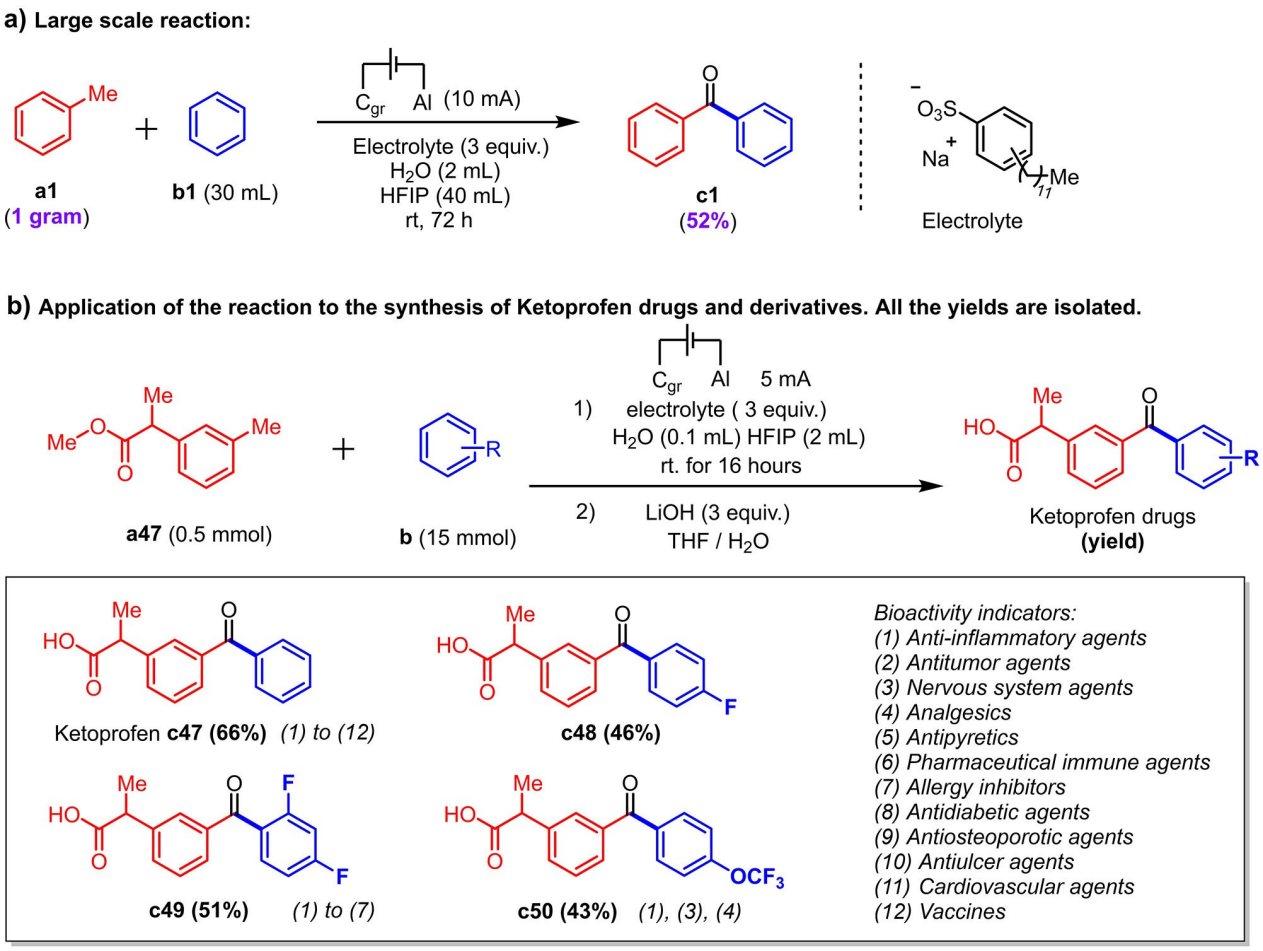
Some applications of the electro‐oxidative intermolecular phenone coupling of methylarenes with aromatic C−H bonds.

In summary, the herein described electro‐oxidative phenome‐coupling reaction represents a new type of cross dehydrogenative coupling involving trivial methylarenes and simple arenes. With its large substrate scope (44 examples, not counting regioisomers), this electrochemical synthetic method will allow direct access to many current and future biologically active targets containing a phenone moiety.

## Conflict of interest

The authors declare no conflict of interest.

## Supporting information

As a service to our authors and readers, this journal provides supporting information supplied by the authors. Such materials are peer reviewed and may be re‐organized for online delivery, but are not copy‐edited or typeset. Technical support issues arising from supporting information (other than missing files) should be addressed to the authors.

Supporting Information

## Data Availability

The data that support the findings of this study are available in the Supporting Information of this article.
