# Supplementary material for: Direct Dehydrogenative Access to Unsymmetrical Phenones
Source: Angew Chem Int Ed Engl. 2022 Mar 19;61(20):e202201142. doi: 10.1002/anie.202201142 (PMC9314079; doi:10.1002/anie.202201142)
Supplement: Supplementary file 1 — Supporting Information [file ANIE-61-0-s001.pdf]

Angewandte Chemie
Eine Zeitschrift der Gesellschaft Deutscher Chemiker

Supporting Information

Direct Dehydrogenative Access to Unsymmetrical Phenones

*C. Yu, R. Huang, F. W. Patureau**

Table of Contents

1. General information……………………………………………………………………………2
2. Condition optimization…………………………………………………………………………2
3. General procedure for the electro-oxidative reactions……………………………………….…7
4. Characterization of the products.………………………………..……….…………………..9
5. Synthesis of the Ketoprofen drugs.…………………………………...………...………...27
6. Selected unsuccessful substrates….………………………………..………………..…...……29
7. Mechanistic studies ……………………………………………..………………..…...……30
8. Copies of ^{1}H and ^{13}C Spectra ………………………………...………………..…………34

1. General information

NMR spectra were obtained on an Agilent VNMRS 400 or a Bruker Av 600 using $CDCl_3$ as solvents. Chemical shifts are given in ppm and coupling constants (*J*) in Hz. ^1H spectra were calibrated in relation to the deuterated solvent, namely $CDCl_3$ (7.26 ppm). ^{13}C spectra were calibrated in relation to the deuterated solvent, namely $CDCl_3$ (77.16 ppm). The following abbreviations were used for ^1H NMR spectra to indicate the signal multiplicity: s (singlet), d (doublet), t (triplet), q (quartet) and m (multiplet) as well as combinations of them. Flash chromatography was performed on silica gel (60 M, 0.04-0.063 mm) by standard technique. All the chemicals used for synthesis were purchased from Sigma Aldrich, abcr, Alfa Aesar, TCI, Fisher, or chemPUR. High resolution mass spectra (HRMS) were recorded on ThermoFisher Scientific LTQ Orbitrap XL spectrometer. All the electrodes were purchased from IKA.

2. Condition optimization

(1) The yields of two products with different conditions (^1H NMR yields with 1,3,5-trimethoxybenzene as an internal standard).

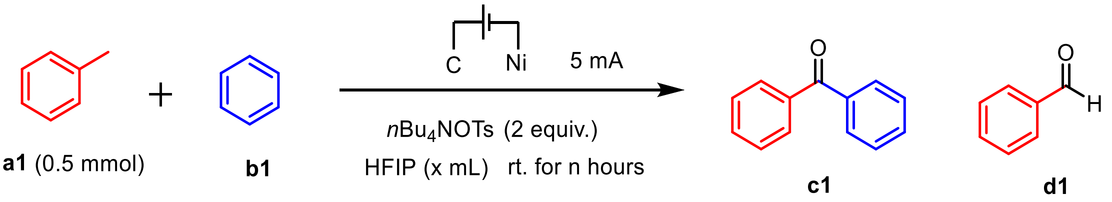

Entry	Benzene	HFIP	Reaction time	Yield of c1	Yield of d1
1	10 mmol	3 mL	16 h	13%	3%
2	15 mmol	2 mL	16 h	25%	10%
3	15 mmol	4 mL	16 h	19%	17%
4	15 mmol	1 mL	16 h	7%	1%
5	15 mmol	2 mL	3 h	9%	8%
6	15 mmol	2 mL	8 h	14%	3%

(2) The yields of two products with different electrolytes (^1H NMR yields with 1,3,5-trimethoxybenzene as an internal standard).

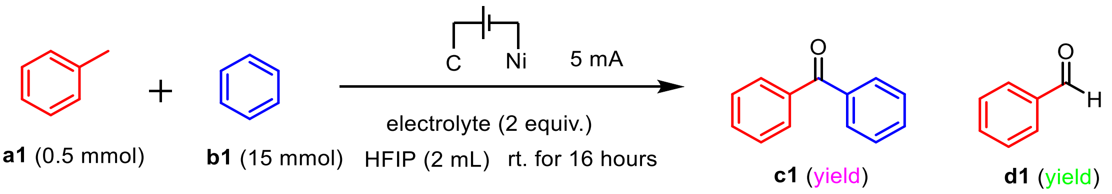

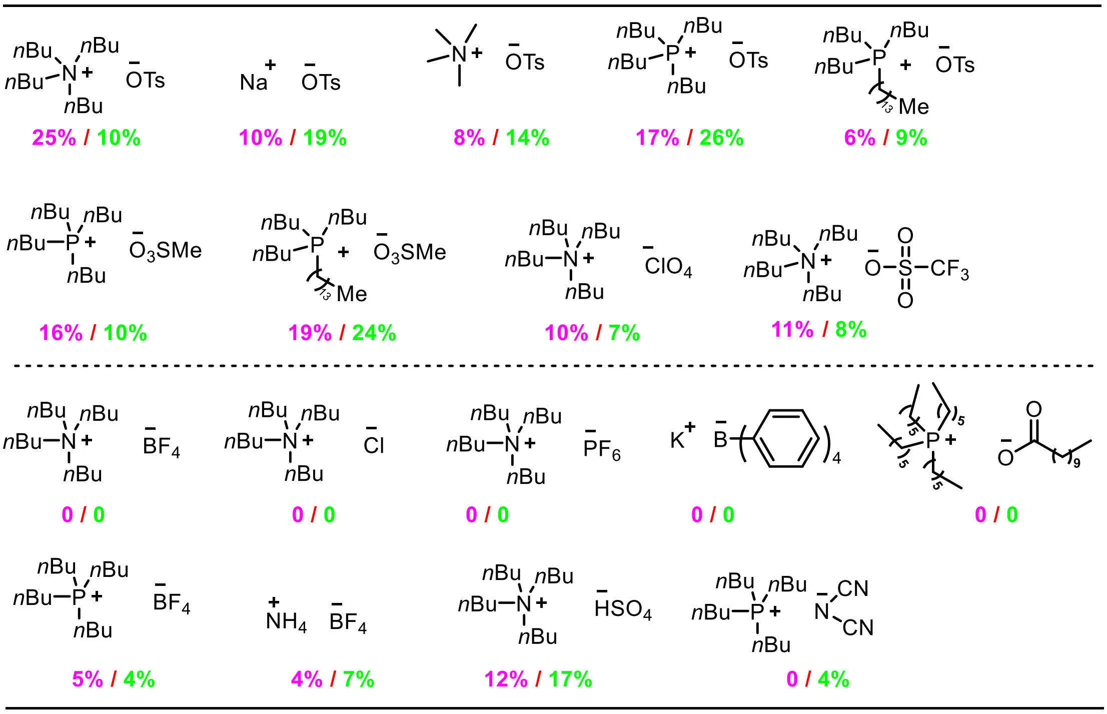

(3) The yields of two products with different solvents and different currents (^{1}H NMR yields with 1,3,5-trimethoxybenzene as an internal standard).

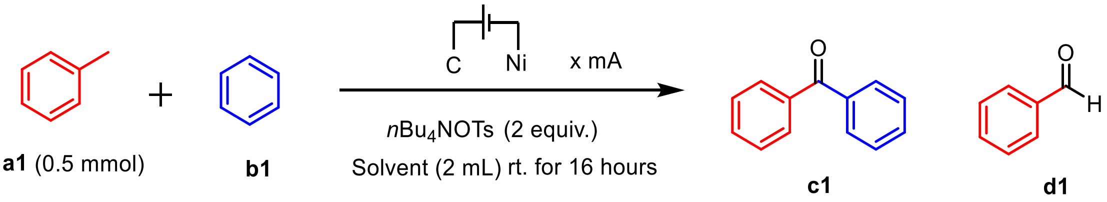

Entry	Benzene	Solvent	Current	Yield of c1	Yield of d1
1	15 mmol	CH_3CN	5 mA	0	0
2	15 mmol	$HOCH_3$	5 mA	0	0
3	15 mmol	$HOCH_2CF_3$	5 mA	20%	24%
4	15 mmol	$HOCH_2CCl_3$	5 mA	8%	0
8	15 mmol	CH_3CN/HFIP (1/1)	5 mA	trace	0
9	15 mmol	HFIP	2.5 mA	21%	6%
10	15 mmol	HFIP	7.5 mA	25%	10%

(4) The yields of two products with different electrodes (^{1}H NMR yields with 1,3,5-trimethoxybenzene as an internal standard).

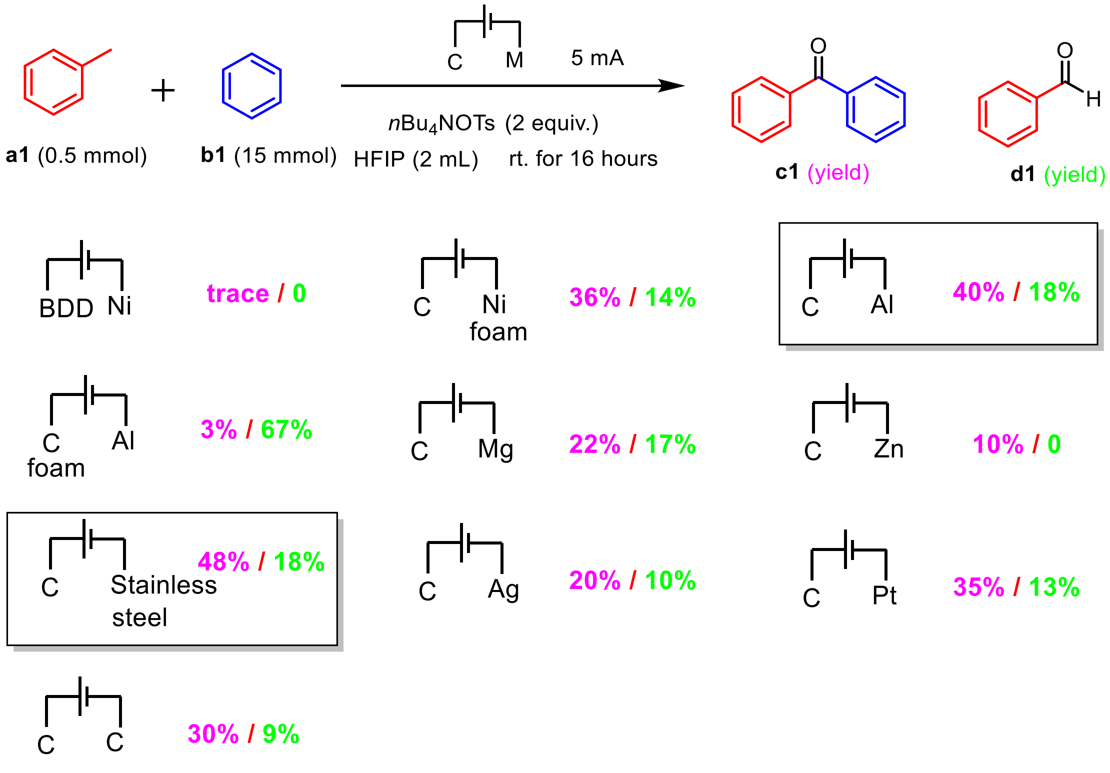

(5) The yields of two products with different electrolytes and cathode material (^{1}H NMR yields with 1,3,5-trimethoxybenzene as an internal standard).

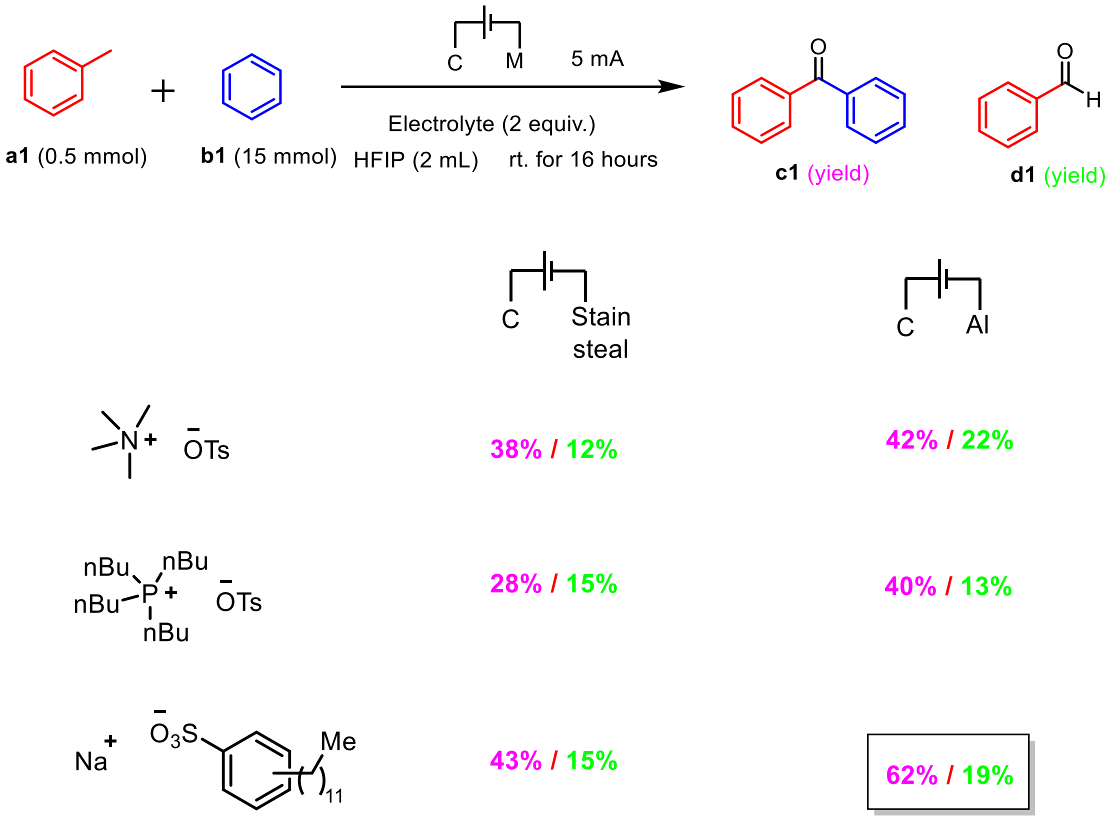

(6) The yields of two products with different currents and additives (^{1}H NMR yields with 1,3,5-trimethoxybenzene as an internal standard).

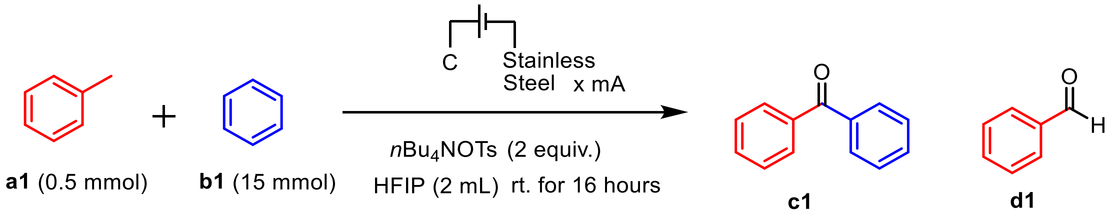

Entry	Benzene	Additive	Current	Yield of c1	Yield of d1
1	15 mmol		2.5 mA	16%	12%
2	15 mmol		5 mA	48%	18%
3	15 mmol		7.5 mA	57%	17%
4	15 mmol	H_2O (0.1 mL)	5 mA	58%	17%

(7) The yields of two products with different currents (^{1}H NMR yields with 1,3,5-trimethoxybenzene as an internal standard).

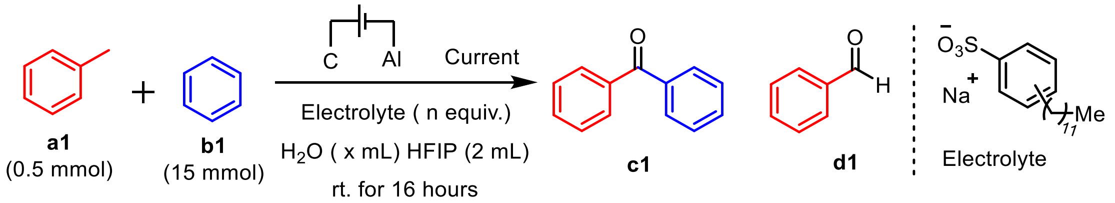

Entry	Electrolyte	Water	Current	Yield of c1	Yield of d1
1	2 equiv.	0.1 mL	2.5 mA	32%	10%
2	2 equiv.	0.1 mL	5 mA	65%	3%
3	2 equiv.	0.1 mL	7.5 mA	74%	10%
**4**	**2 equiv.**	**0.1 mL**	**10 mA**	**75%**	**14%**
5	2 equiv.	0.2 mL	12.5 mA	79%	9%
**6**	**2 equiv.**	**0.2 mL**	**15 mA**	**81%**	**14%**
7	2 equiv.	0.2 mL	17.5 mA	78%	11%

(8) The yields of two products with different addition amounts of water (^{1}H NMR yields with 1,3,5-trimethoxybenzene as an internal standard).

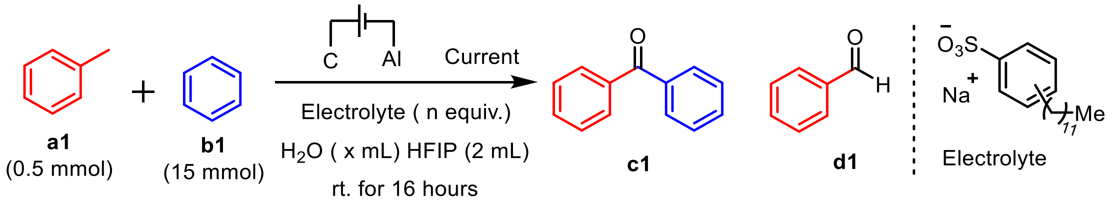

Entry	Electrolyte	Water	Current	Yield of c1	Yield of d1
1	2 equiv.	0.05 mL	5 mA	66%	19%
2	2 equiv.	0.1 mL	5 mA	65%	12%
**3**	**2 equiv.**	**0.2 mL**	**5 mA**	**69%**	**10%**
4	2 equiv.	0.3 mL	5 mA	66%	12%
5	2 equiv.	0.5 mL	5 mA	47%	13%
6	2 equiv.	1.0 mL	5 mA	33%	9%

(9) The yields of two products with different addition amounts of electrolyte (^{1}H NMR yields with 1,3,5-trimethoxybenzene as an internal standard).

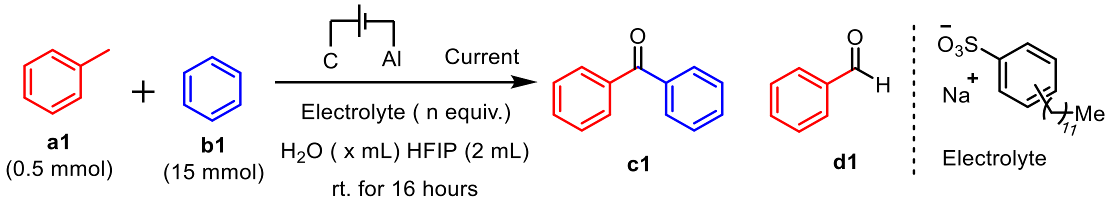

Entry	Electrolyte	Water	Current	Yield of c1	Yield of d1
1	1.5 equiv.	0.1 mL	5 mA	65%	16%
2	2 equiv.	0.1 mL	5 mA	65%	12%
3	2.5 equiv.	0.1 mL	5 mA	59%	19%
**4**	**3 equiv.**	**0.1 mL**	**5 mA**	**70%**	**17%**
5	3.5 equiv.	0.1 mL	5 mA	60%	13%

(10) The yields of two products with different addition amounts of electrolyte (^{1}H NMR yields with 1,3,5-trimethoxybenzene as an internal standard).

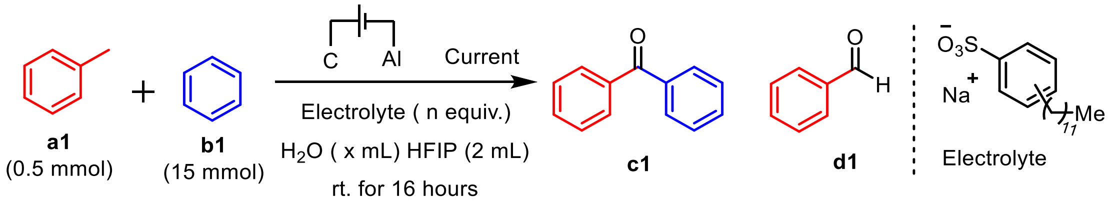

Entry	Electrolyte	Water	Current	Yield of c1	Yield of d1
1	3 equiv.	0.1 mL	5 mA	70%	17%
2	3 equiv.	0.1 mL	7.5 mA	80%	13%
**3**	**3 equiv.**	**0.1 mL**	**10 mA**	**88%**	**10%**
4	3 equiv.	0.1 mL	12.5 mA	75%	15%
5	3 equiv.	0.1 mL	15 mA	No data	No data
6	3 equiv.	0.2 mL	5 mA	69%	16%
7	3 equiv.	0.2 mL	7.5 mA	82%	10%
8	3 equiv.	0.2 mL	10 mA	80%	14%
9	3 equiv.	0.2 mL	12.5 mA	75%	13%
10	3 equiv.	0.2 mL	15 mA	80%	13%
11	3 equiv.	0.3 mL	10 mA	80%	13%

3. General procedure for the electro-oxidative reactions.

(1) The equipment.

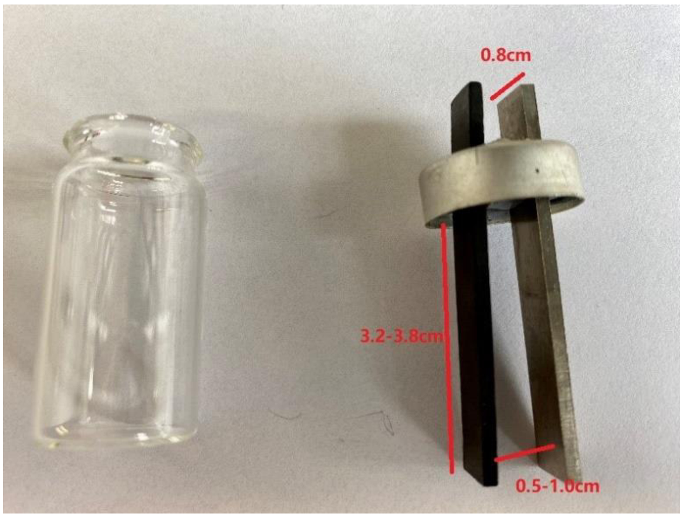

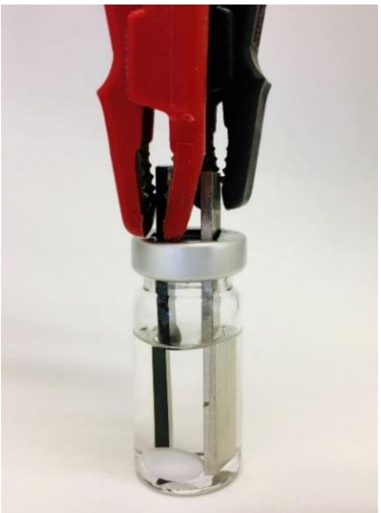

(Volume of liquid in the picture: 6 mL, For most examples in this article, the crude volume is around 3.5 mL)

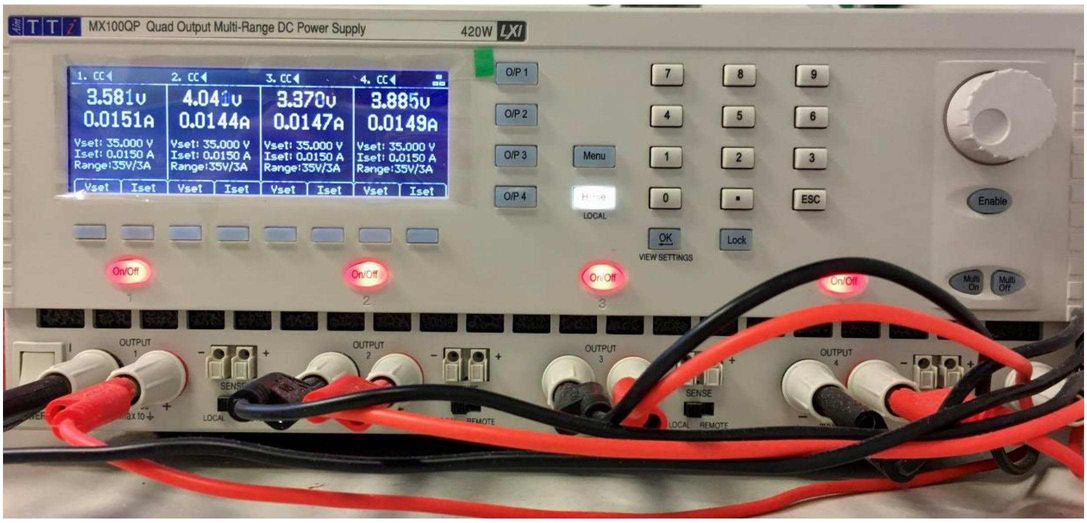

(2) General procedure.

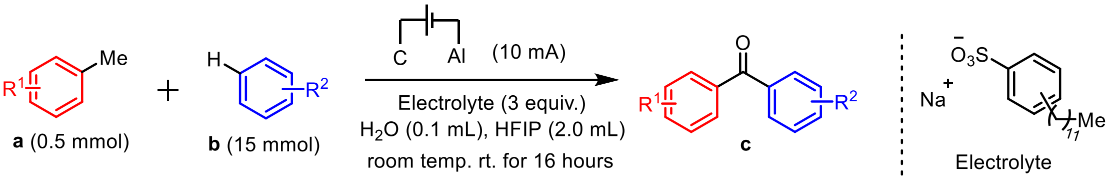

Unless otherwise specified, the methyl arene **a** (0.5 mmol scale), the arene **b** (15.0 mmol), the electrolyte (1.5 mmol), and water (0.1 mL) are added into the solvent HFIP (2.0 mL) in a reaction vial. The reaction vial is then sealed with an aluminous headspace cap with electrodes (see the pictures above). The current is set to 10 mA or 5 mA or other currents. Keep the reaction stirring (circa 400 rpm) at room temperature for 16 hours. After that, the solvent is removed and the crude is directly engaged on SiO_2 gel column chromatography for purification.

(3) Scale-up experiment.

Large scale reaction: pictures from left to right: reaction equipment, reaction before starting, reaction after 72 hours, TLC at the end of the reaction.

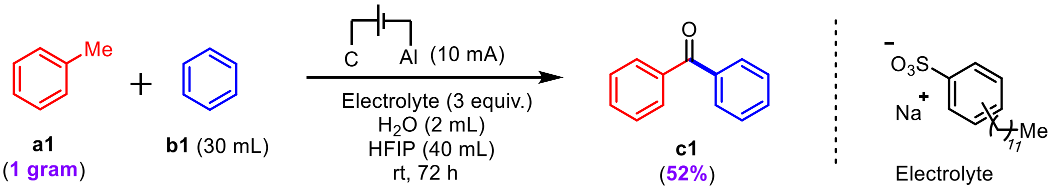

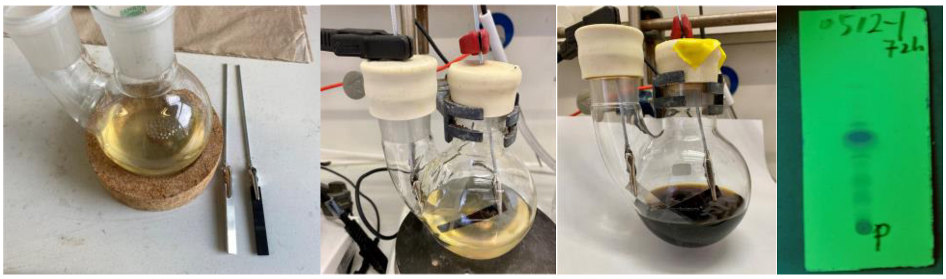

4. Characterization of the products.

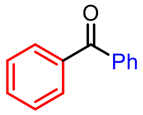

Chemical Formula: $C_{13}H_{10}O$

c1: Following the general procedure, 46 mg (0.5 mmol, 1 equiv.) toluene and 1.33 mL (15.0 mmol, 30 equiv.) benzene were used, the current was 10 mA. The crude mixture was purified by SiO_2 gel column chromatography with pentane/EA (from 50:1 to 40:1). 74 mg product was obtained by 81% isolated yield as a colorless oil.

^{1}H NMR (600 MHz, Chloroform-*d*) δ 7.84 – 7.78 (m, 4H), 7.61 – 7.56 (m, 2H), 7.51 – 7.45 (m, 4H).

^{13}C NMR (151 MHz, Chloroform-*d*) δ 196.83, 137.72, 132.51, 130.15, 128.38.

ESI-HRMS: mass spectrometry: m/z calc. 205.06239 $[C_{13}H_{10}ONa]^+$, measured 205.06197.

IR (neat, cm^{-1}): ṽ: 3867, 3305, 3060, 2873, 2665, 2325, 2113, 1994, 1910, 1821, 1657, 1597, 1445, 1313, 1274, 1175, 1150, 1073, 1027, 998, 919, 849, 809, 763, 697.

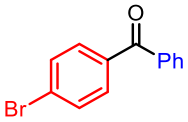

Chemical Formula: $C_{13}H_9BrO$

c2: Following the general procedure, 85.5 mg (0.5 mmol, 1 equiv.) 4-bromotoluene and 1.33 mL (15.0 mmol, 30 equiv.) benzene were used, the current was 10 mA. The crude mixture was purified by SiO_2 gel column chromatography with pentane/EA (from 50:1 to 40:1). 95 mg product was obtained by 73% isolated yield as white solid.

^{1}H NMR (600 MHz, Chloroform-*d*) δ 7.80 – 7.75 (m, 2H), 7.70 – 7.66 (m, 2H), 7.65 – 7.58 (m, 3H), 7.52 – 7.46 (m, 2H).

^{13}C NMR (151 MHz, Chloroform-*d*) δ 195.77, 137.33, 136.47, 132.82, 131.77, 131.71, 130.08, 128.56, 127.65.

ESI-HRMS: mass spectrometry: m/z calc. 282.97290 $[C_{13}H_9OBrNa]^+$, measured 282.97253.

IR (neat, cm^{-1}): ṽ: 3857, 3285, 2930, 2322, 2114, 1925, 1798, 1646, 1578, 1475, 1392, 1278, 1147, 1066, 1006, 938, 917, 841, 787, 724, 692, 656.

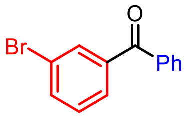

Chemical Formula: $C_{13}H_9BrO$

c3: Following the general procedure, 85.5 mg (0.5 mmol, 1 equiv.) 3-bromotoluene and 1.33 mL (15.0 mmol, 30 equiv.) benzene were used, the current was 10 mA. The crude mixture was purified by SiO_2 gel column chromatography with pentane/EA (from 50:1 to 40:1). 90 mg product was obtained by 69% isolated yield as white solid.

^{1}H NMR (600 MHz, Chloroform-*d*) δ 7.93 (t, *J* = 1.8 Hz, 1H), 7.82 – 7.77 (m, 2H), 7.74 – 7.68 (m, 2H), 7.63 – 7.58 (m, 1H), 7.50 (dd, *J* = 8.6, 7.0 Hz, 2H), 7.36 (t, *J* = 7.9 Hz, 1H).

^{13}C NMR (151 MHz, Chloroform-*d*) δ 195.25, 139.61, 137.04, 135.38, 132.97, 132.90, 130.14, 130.00, 128.67, 128.59, 122.71.

ESI-HRMS: mass spectrometry: m/z calc. 282.97290 $[C_{13}H_9OBrNa]^+$, measured 282.97241.

IR (neat, cm^{-1}): ṽ: 3815, 3284, 3059, 2958, 2322, 1913, 1732, 1649, 1562, 1445, 1415, 1311, 1272, 1179, 1148, 1070, 951, 900, 812, 779, 706, 667.

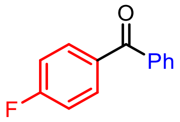

Chemical Formula: $C_{13}H_9FO$

c4: Following the general procedure, 55 mg (0.5 mmol, 1 equiv.) 4-fluorotoluene and 1.33 mL (15.0 mmol, 30 equiv.) benzene were used, the current was 10 mA. The crude mixture was purified by SiO_2 gel column chromatography with pentane/EA (from 50:1 to 40:1). 80 mg product was obtained by 80% isolated yield as white solid.

^1H NMR (600 MHz, Chloroform-*d*) δ 7.88 – 7.82 (m, 2H), 7.81 – 7.74 (m, 2H), 7.62 – 7.57 (m, 1H), 7.49 (t, J = 7.7 Hz, 2H), 7.20 – 7.12 (m, 2H).

^{13}C NMR (151 MHz, Chloroform-*d*) δ 195.41, 165.55 (d, J = 254.3 Hz), 137.66, 133.96 (d, J = 3.5 Hz), 132.81 (d, J = 9.6 Hz), 132.61, 130.02, 128.50, 115.60 (d, J = 21.8 Hz).

^{19}F NMR (565 MHz, Chloroform-*d*) δ -105.94 – -106.02 (m, 1F).

ESI-HRMS: mass spectrometry: m/z calc. 223.05296 $[C_{13}H_9OFNa]^+$, measured 223.05237.

IR (neat, cm^{-1}): ṽ: 3288, 3062, 2959, 2319, 2116, 1922, 1799, 1705, 1647, 1584, 1501, 1479, 1445, 1395, 1277, 1226, 1179, 1148, 1107, 1066, 1006, 970, 920, 842, 788, 726, 691, 657.

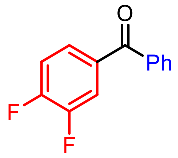

Chemical Formula: $C_{13}H_8F_2O$

c5: Following the general procedure, 64 mg (0.5 mmol, 1 equiv.) 3,4-difluorotoluene and 1.33 mL (15.0 mmol, 30 equiv.) benzene were used, the current was 9 mA. The crude mixture was purified by SiO_2 gel column chromatography with pentane/EA (from 50:1 to 40:1). 95 mg product was obtained by 87% isolated yield as white solid.

^1H NMR (600 MHz, Chloroform-*d*) δ 7.82 – 7.73 (m, 2H), 7.72 – 7.65 (m, 1H), 7.64 – 7.55 (m, 2H), 7.50 (t, J = 7.7 Hz, 2H), 7.32 – 7.22 (m, 1H).

^{13}C NMR (151 MHz, Chloroform-*d*) δ 194.20, 153.38 (dd, J = 256.7 Hz, 13.3 Hz), 150.30 (dd, J = 256.7 Hz, 13.3 Hz), 137.03, 135.11 – 134.23 (m), 132.93, 129.98, 128.64, 127.23 (dd, J = 7.3, 3.6 Hz), 119.45 (d, J = 17.8 Hz), 117.41 (d, J = 17.2 Hz).

^{19}F NMR (565 MHz, Chloroform-*d*) δ -130.45 – -130.96 (m, 1F), -135.72 – -136.63 (m, 1F).

APCI-HRMS: mass spectrometry: m/z calc. 219.06160 $[C_{13}H_9OF_2]^+$, measured 219.06250.

IR (neat, cm^{-1}): ṽ: 3066, 2959, 2932, 2870, 2714, 2616, 2471, 2343, 2149, 2051, 1981, 1923, 1790, 1732, 1650, 1601, 1510, 1422, 1285, 1236, 1201, 1106, 1071, 1026, 970, 939, 896, 856, 837, 796, 767, 725, 695.

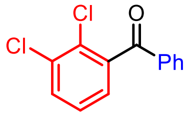

Chemical Formula: $C_{13}H_8Cl_2O$

c6: Following the general procedure, 80 mg (0.5 mmol, 1 equiv.) 1,2-dichlorotoluene and 1.33 mL (15.0 mmol, 30 equiv.) benzene were used, the current was 10 mA. The crude mixture was purified by SiO_2 gel column chromatography with pentane/EA (from 50:1 to 40:1). 55 mg product was obtained by 44% isolated yield as yellow oil.

^{1}H NMR (600 MHz, Chloroform-*d*) δ 7.83 – 7.78 (m, 2H), 7.65 – 7.58 (m, 2H), 7.51 – 7.45 (m, 2H), 7.33 (t, *J* = 7.8 Hz, 1H), 7.28 – 7.26 (m, 1H).

^{13}C NMR (151 MHz, Chloroform-*d*) δ 194.33, 140.91, 136.09, 134.18, 134.07, 131.83, 130.22, 129.72, 128.91, 127.71, 127.01.

ESI-HRMS: mass spectrometry: m/z calc. 272.98444 $[C_{13}H_8OCl_2Na]^+$, measured 272.98401.

IR (neat, cm^{-1}): ṽ: 3334, 3066,2957, 2870, 2323, 2079, 1917, 1732, 1672, 1592, 1448, 1409, 1313, 1281, 1202, 1158, 1100, 1048, 954, 857, 795, 766, 703, 656.

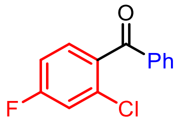

Chemical Formula: $C_{13}H_8ClFO$

c7: Following the general procedure, 72 mg (0.5 mmol, 1 equiv.) 2-chloro-4-fluorotoluene and 1.33 mL (15.0 mmol, 30 equiv.) benzene were used, the current was 10 mA. The crude mixture was purified by SiO_2 gel column chromatography with pentane/EA (from 50:1 to 40:1). 90 mg product was obtained by 77% isolated yield as yellow oil.

^{1}H NMR (600 MHz, Chloroform-*d*) δ 7.82 – 7.77 (m, 2H), 7.63 – 7.59 (m, 1H), 7.50 – 7.45 (m, 2H), 7.40 (dd, *J* = 8.5, 5.9 Hz, 1H), 7.22 (dd, *J* = 8.5, 2.4 Hz, 1H), 7.10 (td, *J* = 8.2, 2.5 Hz, 1H).

^{13}C NMR (151 MHz, Chloroform-*d*) δ 194.47, 163.43 (d, *J* = 254.2 Hz), 136.66, 134.91 (d, *J* = 3.6 Hz), 133.94, 133.14 (d, *J* = 10.9 Hz), 131.10 (d, *J* = 9.5 Hz), 130.18, 128.82, 117.83 (d, *J* = 24.3 Hz), 114.32 (d, *J* = 21.7 Hz).

^{19}F NMR (565 MHz, Chloroform-*d*) δ -107.78 – -107.84 (m, 1F).

ESI-HRMS: mass spectrometry: m/z calc. 257.01399 $[C_{13}H_8OClFNa]^+$, measured 257.01382.

IR (neat, cm^{-1}): ṽ: 3330, 3069, 2960, 2325, 2088, 1909, 1731, 1670, 1594, 1485, 1448, 1387, 1313, 1282, 1255, 1210, 11148, 1045, 938, 862, 824, 732, 700.

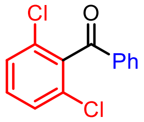

Chemical Formula: $C_{13}H_8Cl_2O$

c8: Following the general procedure, 80 mg (0.5 mmol, 1 equiv.) 2,6-dichlorotoluene and 1.33 mL (15.0 mmol, 30 equiv.) benzene were used, the current was 10 mA. The crude mixture was purified by SiO_2 gel column chromatography with pentane/EA (from 70:1 to 50:1). 100 mg product was obtained by 80% isolated yield as white solid.

^{1}H NMR (600 MHz, Chloroform-*d*) δ 7.86 – 7.80 (m, 2H), 7.65 – 7.60 (m, 1H), 7.49 (t, *J* = 8.3 Hz, 2H), 7.41 – 7.37 (m, 2H), 7.37 – 7.33 (m, 1H).

^{13}C NMR (151 MHz, Chloroform-*d*) δ 192.72, 137.93, 135.61, 134.43, 132.09, 130.86, 129.77, 129.10, 128.26.

ESI-HRMS: mass spectrometry: m/z calc. 272.98444 $[C_{13}H_8OCl_2Na]^+$, measured 272.98441.

IR (neat, cm^{-1}): ṽ: 3340, 3079, 2957, 2866, 2327, 2189, 1996, 1916, 1873, 1823, 1728, 1676, 1579, 1487, 1450, 1427, 1376, 1312, 1269, 1193, 1154, 1092, 1022, 998, 976, 926, 858, 809, 779, 754, 700, 655.

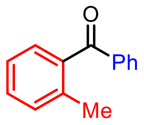

Chemical Formula: $C_{14}H_{12}O$

c9: Following the general procedure, 53 mg (0.5 mmol, 1 equiv.) *o*-xylene and 1.33 mL (15.0 mmol, 30 equiv.) benzene were used, the current was 10 mA. The crude mixture was purified by SiO_2 gel column chromatography with DCM. 45 mg product was obtained by 46% isolated yield as yellow oil.

^{1}H NMR (600 MHz, Chloroform-*d*) δ 7.83 – 7.78 (m, 2H), 7.60 – 7.56 (m, 1H), 7.46 (t, *J* = 7.8 Hz, 2H), 7.39 (td, *J* = 7.5, 1.5 Hz, 1H), 7.33 – 7.28 (m, 2H), 7.27 – 7.22 (m, 1H), 2.34 (s, 3H).

^{13}C NMR (151 MHz, Chloroform-*d*) δ 198.79, 138.78, 137.90, 136.89, 133.27, 131.13, 130.37, 130.27, 128.65, 128.60, 125.33, 20.10.

ESI-HRMS: mass spectrometry: m/z calc. 219.07804 $[C_{14}H_{12}ONa]^+$, measured 219.07750.

IR (neat, cm^{-1}): ṽ: 3860, 3317, 3061, 2859, 2927, 2324, 2085, 1917, 1662, 1596, 1448, 1311, 1264, 1154, 924, 762, 730, 702.

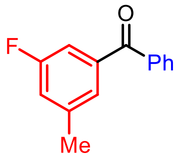

Chemical Formula: $C_{14}H_{11}FO$

c10: Following the general procedure, 62 mg (0.5 mmol, 1 equiv.) 1-fluoro-3,5-dimethylbenzene and 1.33 mL (15.0 mmol, 30 equiv.) benzene were used, the current was 10 mA. The crude mixture was purified by SiO_2 gel column chromatography with pentane/EA (from 50:1 to 40:1). 80 mg product was obtained by 75% isolated yield as yellow oil.

^{1}H NMR (600 MHz, Chloroform-*d*) δ 7.81 – 7.77 (m, 2H), 7.63 – 7.58 (m, 1H), 7.49 (t, *J* = 7.8 Hz, 2H), 7.40 – 7.38 (m, 1H), 7.31 – 7.27 (m, 1H), 7.13 – 7.09 (m, 1H), 2.42 (s, 3H).

^{13}C NMR (151 MHz, Chloroform-*d*) δ 195.72, 162.52 (d, *J* = 247.1 Hz), 140.85 (d, *J* = 8.4 Hz), 139.55 (d, *J* = 7.2 Hz), 137.34, 132.83, 130.14, 128.53, 126.49 (d, *J* = 2.3 Hz), 120.10 (d, *J* = 21.7 Hz), 114.09 (d, *J* = 21.9 Hz), 21.44.

^{19}F NMR (565 MHz, Chloroform-*d*) δ -112.21 – -114.47 (m, 1F).

ESI-HRMS: mass spectrometry: m/z calc. 237.06861 $[C_{14}H_{11}OFNa]^+$, measured 237.06843.

IR (neat, cm^{-1}): ṽ: 3865, 3316, 3063, 2924, 2865, 2655, 2326, 2109, 1992, 1913, 1661, 1591, 1447, 1380, 1314, 1227, 1178, 1145, 1119, 1076, 1024, 984, 963, 867, 816, 722, 695, 663.

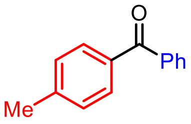

Chemical Formula: $C_{14}H_{12}O$

c11: Following the general procedure, 53 mg (0.5 mmol, 1 equiv.) *p*-xylene and 1.33 mL (15.0 mmol, 30 equiv.) benzene were used, the current was 10 mA. The crude mixture was purified by SiO_2 gel column chromatography with DCM. 50 mg product was obtained by 51% isolated yield as yellow oil.

^{1}H NMR (600 MHz, Chloroform-*d*) δ 7.81 – 7.76 (m, 2H), 7.75 – 7.69 (m, 2H), 7.60 – 7.55 (m, 1H), 7.47 (t, *J* = 7.7 Hz, 2H), 7.28 (d, *J* = 7.8 Hz, 2H), 2.44 (s, 3H).

^{13}C NMR (151 MHz, Chloroform-*d*) δ 196.65, 143.37, 138.12, 135.05, 132.29, 130.45, 130.07, 129.12, 128.35, 21.79.

ESI-HRMS: mass spectrometry: m/z calc. 219.07804 $[C_{14}H_{12}ONa]^{+}$, measured 219.07791.

IR (neat, cm^{-1}): ṽ: 3300, 3058, 3031, 2924, 2868, 2325, 2088, 1985, 1920, 1816, 1654, 1603, 1446, 1406, 1378, 1312, 1275, 1177, 1151, 1111, 1073, 1028, 1000, 923, 836, 785, 729, 697.

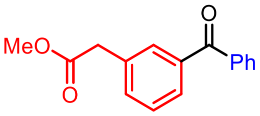

Chemical Formula: $C_{16}H_{14}O_3$

c12: Following the general procedure, 74 mg (0.5 mmol, 1 equiv.) methyl 2-(m-tolyl)acetate and 1.33 mL (15.0 mmol, 30 equiv.) benzene were used, the current was 10 mA. The crude mixture was purified by SiO_2 gel column chromatography with DCM/EA (80/1). 90 mg product was obtained by 71% isolated yield as yellow oil.

^{1}H NMR (600 MHz, Chloroform-*d*) δ 7.82 – 7.78 (m, 2H), 7.74 – 7.72 (m, 1H), 7.72 – 7.68 (m, 1H), 7.61 – 7.57 (m, 1H), 7.54 – 7.51 (m, 1H), 7.48 (t, *J* = 7.8 Hz, 2H), 7.44 (t, *J* = 7.6 Hz, 1H), 3.71 (s, 3H), 3.70 (s, 2H).

^{13}C NMR (151 MHz, Chloroform-*d*) δ 196.55, 171.67, 138.03, 137.63, 134.43, 133.46, 132.62, 131.02, 130.20, 129.09, 128.64, 128.43, 52.28, 41.01.

APCI-HRMS: mass spectrometry: m/z calc. 255.10157 $[C_{16}H_{15}O_3]^{+}$, measured 255.10146.

IR (neat, cm^{-1}): ṽ: 3459, 3060, 2953, 2160, 2028, 1736, 1657, 1596, 1483, 1439, 1313, 1279, 1216, 1158, 1084, 1010, 930, 847, 814, 785, 707.

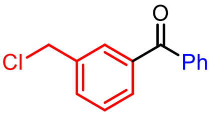

Chemical Formula: $C_{14}H_{11}ClO$

c13: Following the general procedure, 70 mg (0.5 mmol, 1 equiv.) 3-methylbenzyl chloride and 1.33 mL (15.0 mmol, 30 equiv.) benzene were used, the current was 10 mA. The crude mixture was purified by SiO_2 gel column chromatography with pentane/EA (from 50:1 to 40:1). 70 mg product was obtained by 61% isolated yield as brown oil.

^1H NMR (600 MHz, Chloroform-*d*) δ 7.85 – 7.82 (m, 1H), 7.82 – 7.78 (m, 2H), 7.75 (dt, *J* = 7.7, 1.5 Hz, 1H), 7.66 – 7.59 (m, 2H), 7.53 – 7.47 (m, 3H), 4.64 (s, 2H).
^{13}C NMR (151 MHz, Chloroform-*d*) δ 196.30, 138.28, 138.02, 137.47, 132.79, 132.61, 130.21, 130.20, 130.12, 128.92, 128.55, 45.72.
ESI-HRMS: mass spectrometry: m/z calc. 253.03906 $[C_{14}H_{11}OClNa]^+$, measured 253.03896.
IR (neat, cm^{-1}): ṽ: 3307, 3060, 2958, 2338, 1974, 1912, 1819, 1657, 1597, 1444, 1378, 1311, 1285, 1263, 1208, 1178, 1133, 1083, 982, 922, 840, 814, 784. 701.

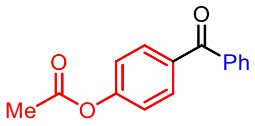

Chemical Formula: $C_{15}H_{12}O_3$

c14: Following the general procedure, 75 mg (0.5 mmol, 1 equiv.) *p*-tolyl acetate and 1.33 mL (15.0 mmol, 30 equiv.) benzene were used, the current was 10 mA. The crude mixture was purified by SiO_2 gel column chromatography with pentane/EA (from 20:1 to 8:1). 60 mg product was obtained by 50% isolated yield as brown solid.
^1H NMR (600 MHz, Chloroform-*d*) δ 7.87 – 7.84 (m, 2H), 7.81 – 7.78 (m, 2H), 7.62 – 7.57 (m, 1H), 7.49 (dd, *J* = 8.5, 7.1 Hz, 2H), 7.24 – 7.20 (m, 2H), 2.34 (s, 3H).
^{13}C NMR (151 MHz, Chloroform-*d*) δ 195.66, 169.03, 154.03, 137.64, 135.20, 132.60, 131.80, 130.08, 128.47, 121.66, 21.29.
ESI-HRMS: mass spectrometry: m/z calc. 263.06787 $[C_{15}H_{12}O_3Na]^+$, measured 263.06766.
IR (neat, cm^{-1}): ṽ: 3510, 3062, 2932, 2324, 2104, 1921, 1761, 1657, 1596, 1499, 1445, 1409, 1369, 1306, 1275, 1189, 1161, 1106, 1011, 913, 855, 786, 743, 700, 658.

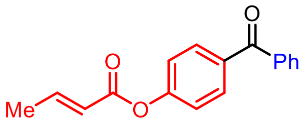

Chemical Formula: $C_{17}H_{14}O_3$

c15: Following the general procedure, 88 mg (0.5 mmol, 1 equiv.) *p*-tolyl crotonate and 1.33 mL (15.0 mmol, 30 equiv.) benzene were used, the current was 5 mA. The crude mixture was purified by SiO_2 gel column chromatography with pentane/EA (from 40:1 to 10:1). 55 mg product was obtained by 41% isolated yield as white solid.
^1H NMR (600 MHz, Chloroform-*d*) δ 7.88 – 7.84 (m, 2H), 7.82 – 7.78 (m, 2H), 7.62 – 7.56 (m, 1H), 7.49 (dd, *J* = 8.5, 7.0 Hz, 2H), 7.27 – 7.20 (m, 3H), 6.07 (dq, *J* = 14.0, 1.7 Hz, 1H), 1.99 (dd, *J* = 7.0, 1.7 Hz, 3H).
^{13}C NMR (151 MHz, Chloroform-*d*) δ 195.73, 164.39, 154.22, 147.99, 137.71, 132.56, 131.79, 130.09, 128.46, 121.86, 121.73, 18.42.
APCI-HRMS: mass spectrometry: m/z calc. 267.10157 $[C_{17}H_{15}O_3]^+$, measured 267.10148.
IR (neat, cm^{-1}): ṽ: 3442, 3051, 2951, 2409, 2292, 2197, 1972, 1918, 1726, 1651, 1595, 1498, 1442, 1374, 1311, 1278, 1203, 1155, 1100, 1015, 973, 934, 916, 851, 792, 757, 727, 694.

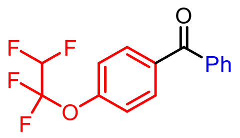

Chemical Formula: $C_{15}H_{10}F_4O_2$

c16: Following the general procedure, 104 mg (0.5 mmol, 1 equiv.) 3-(1,1,2,2-tetrafluoroethoxy)toluene and 1.33 mL (15.0 mmol, 30 equiv.) benzene were used, the current was 10 mA. The crude mixture was purified by SiO_2 gel column chromatography with pentane/EA (from 40:1 to 30:1). 110 mg product was obtained by 74% isolated yield as yellow oil.

^{1}H NMR (600 MHz, Chloroform-*d*) δ 7.89 – 7.83 (m, 2H), 7.82 – 7.76 (m, 2H), 7.64 – 7.56 (m, 1H), 7.50 (t, *J* = 7.8 Hz, 2H), 7.33 (d, *J* = 8.5 Hz, 2H), 5.95 (tt, *J* = 53.0, 2.8 Hz, 1H).

^{13}C NMR (151 MHz, Chloroform-*d*) δ 195.40, 152.15, 137.38, 135.76, 132.80, 132.00, 130.08, 128.55, 121.10, 116.63 (tt, *J* = 273.7, 28.8 Hz), 107.69 (tt, *J* = 252.7, 41.2 Hz).

^{19}F NMR (565 MHz, Chloroform-*d*) δ -136.68 – -136.74 (m, 2F), -136.75 – -136.84 (m, 2F).

ESI-HRMS: mass spectrometry: m/z calc. 321.05091 $[C_{15}H_{10}O_2F_4Na]^+$, measured 321.05101.

IR (neat, cm^{-1}): ṽ: 3065, 3004, 2928, 2161, 1979, 1992, 1650, 1597, 1500, 1447, 1418, 1303, 1275, 1194, 1100, 1015, 968, 923, 850, 797, 766, 728, 693.

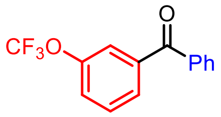

Chemical Formula: $C_{14}H_9F_3O_2$

c17: Following the general procedure, 88 mg (0.5 mmol, 1 equiv.) 3-trifluoromethoxytoluene and 1.33 mL (15.0 mmol, 30 equiv.) benzene were used, the current was 10 mA. The crude mixture was purified by SiO_2 gel column chromatography with pentane/EA (from 70:1 to 50:1). 90 mg product was obtained by 67% isolated yield as colorless oil.

^{1}H NMR (600 MHz, Chloroform-*d*) δ 7.83 – 7.78 (m, 2H), 7.73 (d, *J* = 7.6, 1H), 7.67 (s, 1H), 7.64 – 7.60 (m, 1H), 7.52 (dt, *J* = 13.2, 7.9 Hz, 3H), 7.47 – 7.43 (m, 1H).

^{13}C NMR (151 MHz, Chloroform-*d*) δ 195.13, 149.30, 139.63, 137.00, 133.07, 130.17, 130.00, 128.64, 128.48, 124.85, 122.49, 120.59 (q, *J* = 257.9 Hz).

^{19}F NMR (565 MHz, Chloroform-*d*) δ -57.88 (s, 3F).

ESI-HRMS: mass spectrometry: m/z calc. 289.04469 $[C_{14}H_9O_2F_3Na]^+$, measured 289.04460.

IR (neat, cm^{-1}): ṽ: 3071, 2930, 1907, 1734, 1664, 1586, 1483, 1442, 1250, 1211, 1162, 1084, 998, 909, 828, 785, 696.

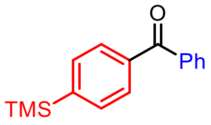

Chemical Formula: $C_{16}H_{18}OSi$

c18: Following the general procedure, 82 mg (0.5 mmol, 1 equiv.) *p*-tolyltrimethylsilane and 1.33 mL (15.0 mmol, 30 equiv.) benzene were used, the current was 10 mA. The crude mixture was purified by SiO_2 gel column chromatography with pentane/Et_2O (from 50:1 to 40:1). 80 mg product was obtained by 63% isolated yield as colorless oil.

^{1}H NMR (600 MHz, Chloroform-*d*) δ 7.83 – 7.80 (m, 2H), 7.78 – 7.75 (m, 2H), 7.66 – 7.62 (m, 2H), 7.61 – 7.57 (m, 1H), 7.48 (dd, *J* = 8.5, 7.0 Hz, 2H), 0.32 (s, 9H).
^{13}C NMR (151 MHz, Chloroform-*d*) δ 198.13, 147.55, 138.98, 138.90, 134.42, 133.63, 131.32, 130.26, 129.50, 0.05.
ESI-HRMS: mass spectrometry: m/z calc. 277.10191 $[C_{16}H_{18}OSiNa]^+$, measured 277.10162.
IR (neat, cm^{-1}): ṽ: 3310, 2955, 2326, 2077, 1733, 1659, 1595, 1447, 1387, 1315, 1276, 1251, 1102, 925, 836, 726, 697, 661.

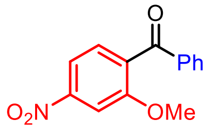

Chemical Formula: $C_{14}H_{11}NO_4$

c19: Following the general procedure, 83.5 mg (0.5 mmol, 1 equiv.) 2-methyl-5-nitroanisole and 1.33 mL (15.0 mmol, 30 equiv.) benzene were used, the current was 10 mA. The crude mixture was purified by SiO_2 gel column chromatography with pentane/EA (from 20:1 to 10:1). 90 mg product was obtained by 70% isolated yield as white solid.
^{1}H NMR (600 MHz, Chloroform-*d*) δ 7.94 (dd, *J* = 8.2, 2.0 Hz, 1H), 7.85 (d, *J* = 2.0 Hz, 1H), 7.78 (dd, *J* = 8.3, 1.4 Hz, 2H), 7.61 (tt, *J* = 7.3, 1.3 Hz, 1H), 7.51 – 7.44 (m, 3H), 3.84 (s, 3H).
^{13}C NMR (151 MHz, Chloroform-*d*) δ 194.55, 157.79, 150.09, 136.63, 135.05, 134.01, 129.87, 129.71, 128.76, 115.96, 106.64, 56.4.
ESI-HRMS: mass spectrometry: m/z calc. 280.05803 $[C_{14}H_{11}O_4NNa]^+$, measured 280.05783.
IR (neat, cm^{-1}): ṽ: 3344, 3106, 2945, 2860, 2630, 2490, 2323, 2171, 2046, 1982, 1922, 1827, 1734, 1677, 1592, 1514, 1482, 1447, 1400, 1346, 1313, 1278, 1246, 1177, 1147, 1117, 1084, 1015, 967, 935, 906, 867, 836, 800, 738, 713, 690, 659.

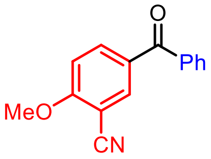

Chemical Formula: $C_{15}H_{11}NO_2$

c20: Following the general procedure, 73.5 mg (0.5 mmol, 1 equiv.) 2-methoxy-5-methylbenzonitrile and 1.33 mL (15.0 mmol, 30 equiv.) benzene were used, the current was 10 mA. The crude mixture was purified by SiO_2 gel column chromatography with pentane/EA (from 10:1 to 4:1). 60 mg product was obtained by 51% isolated yield as yellow solid.
^{1}H NMR (600 MHz, Chloroform-*d*) δ 8.08 (dd, *J* = 8.8, 2.2 Hz, 1H), 8.04 (d, *J* = 2.2 Hz, 1H), 7.74 – 7.70 (m, 2H), 7.64 – 7.59 (m, 1H), 7.50 (t, *J* = 7.8 Hz, 2H), 7.08 (d, *J* = 8.8 Hz, 1H), 4.03 (s, 3H).
^{13}C NMR (151 MHz, Chloroform-*d*) δ 193.70, 164.15, 137.11, 136.68, 136.41, 132.85, 130.54, 129.80, 128.69, 115.61, 111.28, 102.14, 56.72.
ESI-HRMS: mass spectrometry: m/z calc. 260.06820 $[C_{15}H_{11}O_2NNa]^+$, measured 260.06766.
IR (neat, cm^{-1}): ṽ: 3288, 7088, 2924, 2854, 2232, 1835, 1645, 1595, 1494, 1445, 1278, 1150, 1108, 1009, 979, 919, 833, 796, 740, 702

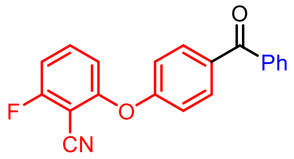

Chemical Formula: $C_{20}H_{12}FNO_2$

c21: Following the general procedure, 113.5 mg (0.5 mmol, 1 equiv.) 2-fluoro-6-(*p*-tolyloxy)benzonitrile and 1.33 mL (15.0 mmol, 30 equiv.) benzene were used, the current was 10 mA. The crude mixture was purified by SiO_2 gel column chromatography with pentane/EA (from 10:1 to 4:1). 60 mg product was obtained by 38% isolated yield as brown solid.

^{1}H NMR (600 MHz, Chloroform-*d*) δ 7.92 – 7.87 (m, 2H), 7.82 – 7.78 (m, 2H), 7.63 – 7.58 (m, 1H), 7.55 – 7.48 (m, 3H), 7.20 – 7.16 (m, 2H), 6.99 (t, *J* = 8.4, 1H), 6.78 (d, *J* = 8.5, 1H).

^{13}C NMR (151 MHz, Chloroform-*d*) δ 195.40, 164.27 (d, *J* = 260.5 Hz), 159.64 (d, *J* = 3.7 Hz), 158.41, 137.53, 135.22 (d, *J* = 9.7 Hz), 134.65, 132.71, 130.05, 128.55, 119.33, 113.53 (d, *J* = 3.5 Hz), 111.15, 111.01, 110.85, 94.99 (d, *J* = 17.6 Hz).

^{19}F NMR (565 MHz, Chloroform-*d*) δ -103.80 (s, 1F).

ESI-HRMS: mass spectrometry: m/z calc. 340.07443 $[C_{20}H_{12}O_2NFNa]^+$, measured 340.07376.

IR (neat, cm^{-1}): ṽ: 3429, 3065, 2931, 2236, 2040, 1921, 1788, 1656, 1581, 1499, 1464, 1411, 1376, 1278, 1244, 1211, 1161, 1106, 1016, 924, 855, 786, 736, 700.

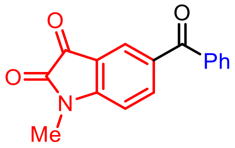

Chemical Formula: $C_{16}H_{11}NO_3$

c22: Following the general procedure, 87.5 mg (0.5 mmol, 1 equiv.) 1,5-dimethylisatin and 1.33 mL (15.0 mmol, 30 equiv.) benzene were used, the current was 5 mA. The crude mixture was purified by SiO_2 gel column chromatography with pentane/EA (from 10:1 to 1:1). 58 mg product was obtained by 44% isolated yield as red solid.

^{1}H NMR (600 MHz, Chloroform-*d*) δ 8.20 (dd, *J* = 8.2, 1.8 Hz, 1H), 8.01 (d, *J* = 1.8 Hz, 1H), 7.75 – 7.71 (m, 2H), 7.64 – 7.59 (m, 1H), 7.50 (t, *J* = 7.8 Hz, 2H), 7.04 (d, *J* = 8.2 Hz, 1H), 3.33 (s, 3H).

^{13}C NMR (151 MHz, Chloroform-*d*) δ 194.25, 182.53, 158.46, 154.47, 140.72, 137.04, 133.45, 132.95, 129.77, 128.76, 127.35, 116.93, 110.11, 26.70.

ESI-HRMS: mass spectrometry: m/z calc. 288.06311 $[C_{16}H_{11}O_3NNa]^+$, measured 288.06214.

IR (neat, cm^{-1}): ṽ: 3938, 3730, 3477, 3221, 3065, 2925, 2854, 2636, 2324, 2206, 2165, 2106, 2055, 2000, 1997, 1912, 1745, 1642, 1605, 1483, 1447, 1354, 1314, 1272, 1188, 1104, 1030, 998, 936, 838, 797, 736, 702, 665.

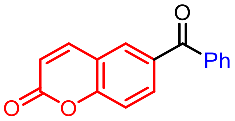

Chemical Formula: $C_{16}H_{10}O_3$

c23: Following the general procedure, 80 mg (0.5 mmol, 1 equiv.) 6-methylcoumarin and 1.33 mL (15.0 mmol, 30 equiv.) benzene were used, the current was 10 mA. The crude mixture was purified

by SiO_2 gel column chromatography with pentane/EA (from 10:1 to 3:1). 88 mg product was obtained by 70% isolated yield as brown solid.

^{1}H NMR (600 MHz, Chloroform-*d*) δ 8.01 – 7.94 (m, 2H), 7.80 – 7.73 (m, 3H), 7.65 – 7.59 (m, 1H), 7.54 – 7.48 (m, 2H), 7.41 (d, J = 8.4 Hz, 1H), 6.49 (d, J = 9.5 Hz, 1H).

^{13}C NMR (151 MHz, Chloroform-*d*) δ 194.76, 159.95, 156.63, 143.23, 137.18, 133.99, 133.48, 132.92, 130.39, 129.98, 128.65, 118.63, 117.74, 117.12.

ESI-HRMS: mass spectrometry: m/z calc. 273.05222 $[C_{16}H_{10}O_3Na]^+$, measured 273.05179.

IR (neat, cm^{-1}): ṽ: 3831, 3421, 3065, 2924, 2856, 2666, 2432, 2324, 2084, 1995, 1947, 1808, 1716, 1657, 1618, 1599, 1446, 1375, 1291, 1256, 1209, 1178, 1127, 1033, 997, 963, 910, 882, 826, 789, 759, 720, 694, 672.

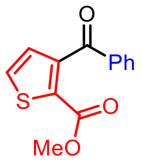

Chemical Formula: $C_{13}H_{10}O_3S$

c24: Following the general procedure, 78 mg (0.5 mmol, 1 equiv.) methyl 3-methylthiophene-2-carboxylate and 1.33 mL (15.0 mmol, 30 equiv.) benzene were used, the current was 10 mA. The crude mixture was purified by SiO_2 gel column chromatography with pentane/EA (from 40:1 to 10:1). 50 mg product was obtained by 40% isolated yield as white solid.

^{1}H NMR (600 MHz, Chlorof orm-*d*) δ 7.83 – 7.79 (m, 2H), 7.60 (d, J = 5.0 Hz, 1H), 7.60 – 7.55 (m, 1H), 7.48 – 7.43 (m, 2H), 7.16 (d, J = 5.0 Hz, 1H), 3.64 (s, 3H).

^{13}C NMR (151 MHz, Chloroform-*d*) δ 193.38, 161.68, 146.04, 137.18, 133.69, 131.81, 131.72, 129.52, 128.72, 128.54, 52.44.

APCI-HRMS: mass spectrometry: m/z calc. 247.04234 $[C_{13}H_{11}O_3S]^+$, measured 247.04198.

IR (neat, cm^{-1}): ṽ: 3400, 3119, 3073, 2952, 2677, 2326, 2200, 2163, 2055, 1999, 1920, 1801, 1705, 1661, 1592, 1530, 1436, 1402, 1368, 1266, 1176, 1103, 1076, 1009, 948, 904, 869, 776, 704.

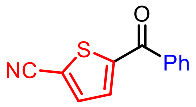

Chemical Formula: $C_{12}H_7NOS$

c25: Following the general procedure, 61.5 mg (0.5 mmol, 1 equiv.) 5-methylthiophene-2-carbonitrile and 1.33 mL (15.0 mmol, 30 equiv.) benzene were used, the current was 10 mA. The crude mixture was purified by SiO_2 gel column chromatography with pentane/EA (from 40:1 to 10:1). 75 mg product was obtained by 70% isolated yield as yellow solid.

^{1}H NMR (600 MHz, Chloroform-*d*) δ 7.89 – 7.82 (m, 2H), 7.68 – 7.63 (m, 2H), 7.61 (d, J = 3.9 Hz, 1H), 7.53 (t, J = 7.6 Hz, 2H).

^{13}C NMR (151 MHz, Chloroform-*d*) δ 186.96, 149.10, 137.40, 136.78, 133.50, 133.25, 129.38, 128.92, 116.51, 113.43.

APCI-HRMS: mass spectrometry: m/z calc. 214.03211 $[C_{12}H_8NOS]^+$, measured 214.03168.

IR (neat, cm^{-1}): ṽ: 3844, 3105, 3059, 2925, 2721, 2569, 2323, 2224, 2109, 1999, 1922, 1839, 1729, 1628, 1593, 1514, 1438, 1324, 1283, 1220, 1180, 1130, 1075, 1051, 1023, 981, 938, 866, 829, 793, 748, 706, 669.

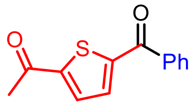

Chemical Formula: $C_{13}H_{10}O_2S$

c26: Following the general procedure, 81 mg (0.58 mmol, 1 equiv.) 2-acetyl-5-methylthiophene and 1.172 g (15.0 mmol, 30 equiv.) benzene were used, the current was 10 mA. The crude mixture was purified by SiO_2 gel column chromatography with pentane/EA (10:1). 70 mg product was obtained by 52.6% isolated yield as yellow solid.

^{1}H NMR (600 MHz, Chloroform-*d*) δ 7.85 (second order m, 2H), 7.68 (d, *J* = 4.0 Hz, 1H), 7.62 (d, *J* = 4.0 Hz, 1H), 7.61 (tt, *J* = 7.4 Hz, *J* = 1.3 Hz, 1H), 7.50 (broad t, *J* ≈ 7.6 Hz, 2H), 2.60 (s, 3H).

^{13}C NMR (151 MHz, Chloroform-*d*) δ 190.94, 188.14, 149.40, 148.41, 137.29, 134.23, 133.04, 131.78, 129.38, 128.70, 27.24.

APCI-HRMS: mass spectrometry: m/z calc. 231.04743 $[C_{13}H_{11}O_2S]^+$, measured 231.04783.

IR (neat, cm^{-1}): ṽ: 3092, 2924, 2856, 2705, 2323, 1831, 1659, 1628, 1577, 1516, 1443, 1361, 1268, 1132, 1104, 1075, 1021, 936, 867, 824, 782, 753, 694, 663.

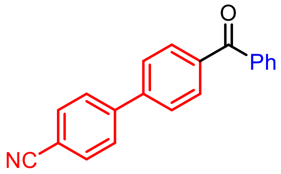

Chemical Formula: $C_{20}H_{13}NO$

c27: Following the general procedure, 96.5 mg (0.5 mmol, 1 equiv.) 4'-cyano-4-methylbiphenyl and 1.33 mL (15.0 mmol, 30 equiv.) benzene were used, the current was 10 mA. The crude mixture was purified by SiO_2 gel column chromatography with pentane/EA (from 40:1 to 10:1). 96 mg product was obtained by 68% isolated yield as brown solid.

^{1}H NMR (600 MHz, Chloroform-*d*) δ 7.94 – 7.89 (m, 2H), 7.85 – 7.81 (m, 2H), 7.79 – 7.73 (m, 4H), 7.73 – 7.68 (m, 2H), 7.62 (t, *J* = 7.4 Hz, 1H), 7.51 (t, *J* = 7.8 Hz, 2H).

^{13}C NMR (151 MHz, Chloroform-*d*) δ 196.12, 144.53, 143.04, 137.64, 137.51, 132.88, 132.78, 130.96, 130.13, 128.53, 128.07, 127.28, 118.78, 111.97.

APCI-HRMS: mass spectrometry: m/z calc. 284.10699 $[C_{20}H_{14}NO]^+$, measured 284.10753.

IR (neat, cm^{-1}): ṽ: 3902, 3746, 3434, 4276, 3060, 2925, 2858, 2664, 2324, 2222, 2082, 2003, 1910, 1734, 1664, 1597, 1521, 1489, 1442, 1392, 1331, 1275, 1209, 1175, 1147, 1073, 1000, 972, 923, 843, 823, 789, 748, 721, 694, 658.

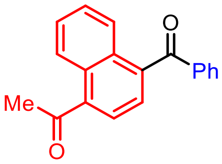

Chemical Formula: $C_{19}H_{14}O_2$

c28: Following the general procedure, 92 mg (0.5 mmol, 1 equiv.) 4'-methyl-1'-Acetonaphthone and 1.33 mL (15.0 mmol, 30 equiv.) benzene were used, the current was 5 mA. The crude mixture was purified by SiO_2 gel column chromatography with pentane/EA (from 20:1 to 5:1). 60 mg product was obtained by 44% isolated yield as brown solid.

^{1}H NMR (600 MHz, Chloroform-*d*) δ 8.69 – 8.64 (m, 1H), 7.97 – 7.92 (m, 1H), 7.91 (d, *J* = 7.3 Hz, 1H), 7.87 – 7.82 (m, 2H), 7.66 – 7.59 (m, 2H), 7.55 (d, *J* = 7.3 Hz, 1H), 7.54 – 7.49 (m, 1H), 7.49 – 7.43 (m, 2H), 2.79 (s, 3H).

^{13}C NMR (151 MHz, Chloroform-*d*) δ 202.02, 197.66, 141.01, 138.24, 137.60, 133.95, 131.48, 130.49, 130.32, 128.79, 128.44, 127.62, 126.37, 126.34, 126.13, 124.96, 30.47.

ESI-HRMS: mass spectrometry: m/z calc. 297.08860 $[C_{19}H_{14}O_2Na]^+$, measured 297.08813.

IR (neat, cm^{-1}): ṽ: 3854, 3320, 3062, 3009, 2924, 2856, 2288, 2198, 2102, 1918, 1814, 1726, 1663, 1583, 1551, 1452, 1418, 1351, 1321, 1288, 1243, 1212, 1177, 1106, 1072, 1045, 1022, 930, 960, 876, 843, 762, 709, 680.

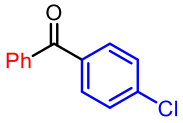

Chemical Formula: $C_{13}H_9ClO$

c29: Following the general procedure, 46 mg (0.5 mmol, 1 equiv.) toluene and 1.52 mL (15.0 mmol, 30 equiv.) chlorobenzene were used, the current was 10 mA. The crude mixture was purified by SiO_2 gel column chromatography with pentane/EA (30:1) and then DCM. 42 mg product was obtained by 39% isolated yield as white solid.

^{1}H NMR (600 MHz, Chloroform-*d*) δ 7.81 – 7.73 (m, 4H), 7.60 (t, *J* = 7.4 Hz, 1H), 7.52 – 7.43 (m, 4H).

^{13}C NMR (151 MHz, Chloroform-*d*) δ 195.58, 139.01, 137.37, 136.01, 132.75, 131.58, 130.04, 128.76, 128.52.

ESI-HRMS: mass spectrometry: m/z calc. 239.02341 $[C_{13}H_9OClNa]^+$, measured 239.02329.

IR (neat, cm^{-1}): ṽ: 3328, 3062, 2928, 2858, 2324, 2085, 1999, 1921, 1816, 1734, 1669, 1591, 1469, 1435, 1313, 1286, 1254, 1153, 1057, 1031, 1000, 927, 867, 800, 763, 740, 699.

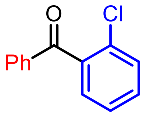

Chemical Formula: $C_{13}H_9ClO$

c30: Following the general procedure, 46 mg (0.5 mmol, 1 equiv.) toluene and 1.52 mL (15.0 mmol, 30 equiv.) chlorobenzene were used, the current was 10 mA. The crude mixture was purified by SiO_2 gel column chromatography with pentane/EA (30:1) and then DCM. 34 mg product was obtained by 31% isolated yield as colorless oil.

^{1}H NMR (600 MHz, Chloroform-*d*) δ 7.82 (d, *J* = 8.2, 2H), 7.63 – 7.56 (m, 1H), 7.50 – 7.41 (m, 4H), 7.40 – 7.35 (m, 2H).

^{13}C NMR (151 MHz, Chloroform-*d*) δ 195.40, 138.76, 136.63, 133.83, 131.45, 131.25, 130.21, 129.26, 128.75, 126.81.

ESI-HRMS: mass spectrometry: m/z calc. 239.02341 $[C_{13}H_9OClNa]^+$, measured 239.02296.

IR (neat, cm^{-1}): ṽ: 3285, 3063, 2967, 2929, 2865, 2740, 2566, 2322, 2165, 2081, 1982, 1923, 1801, 1647, 1581, 1481, 1444, 1398, 1275, 1174, 1149, 1007, 970, 919, 842, 787, 727, 691, 663.

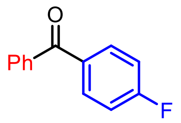

Chemical Formula: $C_{13}H_9FO$

c31: Following the general procedure, 46 mg (0.5 mmol, 1 equiv.) toluene and 1.40 mL (15.0 mmol, 30 equiv.) fluorobenzene were used, the current was 9 mA. The crude mixture was purified by SiO_2 gel column chromatography with DCM. 52 mg product was obtained by 52% isolated yield as yellow oil.

^{1}H NMR (600 MHz, Chloroform-*d*) δ 7.87 – 7.82 (m, 2H), 7.79 – 7.74 (m, 2H), 7.62 – 7.57 (m, 1H), 7.49 (t, *J* = 7.8 Hz, 2H), 7.19 – 7.13 (m, 2H).

^{13}C NMR (151 MHz, Chloroform-*d*) δ 195.39, 165.54 (d, *J* = 253.7 Hz), 137.65, 133.95 (d, *J* = 3.5 Hz), 132.80 (d, *J* = 9.6 Hz), 132.60, 130.01, 128.49, 115.59 (d, *J* = 21.8 Hz).

^{19}F NMR (565 MHz, Chloroform-*d*) δ -105.92 – -106.03 (m, 1F).

ESI-HRMS: mass spectrometry: m/z calc. 223.05296 $[C_{13}H_9OFNa]^+$, measured 223.05247

IR (neat, cm^{-1}): ṽ: 3904, 3320, 3221, 3064, 2958, 2745, 2324, 2080, 1991, 1924, 1814, 1731, 1664, 1607, 1482, 1449, 1292, 1219, 1154, 1102, 1028, 999, 929, 865, 821, 760, 697.

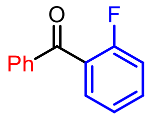

Chemical Formula: $C_{13}H_9FO$

c32: Following the general procedure, 46 mg (0.5 mmol, 1 equiv.) toluene and 1.40 mL (15.0 mmol, 30 equiv.) fluorobenzene were used, the current was 9 mA. The crude mixture was purified by SiO_2 gel column chromatography with DCM. 26 mg product was obtained by 26% isolated yield as colorless oil.

^{1}H NMR (600 MHz, Chloroform-*d*) δ 7.84 (d, *J* = 8.2 Hz, 2H), 7.60 (t, *J* = 7.4 Hz, 1H), 7.58 – 7.51 (m, 2H), 7.48 (t, *J* = 7.8 Hz, 2H), 7.30 – 7.25 (m, 1H), 7.16 (dd, *J* = 9.8, 8.4 Hz, 1H).

^{13}C NMR (151 MHz, Chloroform-*d*) δ 193.61, 160.25 (d, *J* = 252.0 Hz), 137.56, 133.55, 133.19 (d, *J* = 7.6 Hz), 130.89, 129.96, 128.61, 127.21 (d, *J* = 14.6 Hz), 124.42 (d, *J* = 3.6 Hz), 116.42 (d, *J* = 21.8 Hz).

^{19}F NMR (565 MHz, Chloroform-*d*) δ -110.96 – -111.09 (m, 1F).

ESI-HRMS: mass spectrometry: m/z calc. 223.05296 $[C_{13}H_9OFNa]^+$, measured 223.05252.

IR (neat, cm^{-1}): ṽ: 3912, 3439, 3309, 3193, 3064, 2958, 2745, 2324, 2086, 1999, 1911, 1658, 1596, 1503, 1446, 1407, 1370, 1301, 1274, 1230, 1153, 1097, 1008, 924, 849, 817, 792, 736, 697.

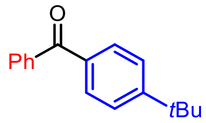

Chemical Formula: $C_{17}H_{18}O$

c33: Following the general procedure, 46 mg (0.5 mmol, 1 equiv.) toluene and 2.3 mL (15.0 mmol, 30 equiv.) *tert*-butylbenzene were used, the current was 10 mA. The crude mixture was purified by SiO_2 gel column chromatography with DCM. 75 mg product was obtained by 63% isolated yield as colorless oil.

^{1}H NMR (600 MHz, Chloroform-*d*) δ 7.81 (d, *J* = 7.9 Hz, 2H), 7.77 (d, *J* = 8.4 Hz, 2H), 7.60 – 7.55 (m, 1H), 7.52 – 7.45 (m, 4H), 1.37 (s, 9H).

^{13}C NMR (151 MHz, Chloroform-*d*) δ 196.68, 156.41, 138.20, 135.07, 132.40, 130.37, 130.21, 128.44, 125.48, 35.34, 31.38.

ESI-HRMS: mass spectrometry: m/z calc. 261.12499 $[C_{17}H_{18}ONa]^+$, measured 261.12471.

IR (neat, cm^{-1}): ṽ: 3307, 3062, 2961, 2870, 2325, 2079, 1999, 1906, 1817, 1736, 1658, 1595, 1477, 1449, 1423, 1365, 1288, 1251, 1201, 1177, 1155, 1090, 1026, 1000, 962, 924, 845, 816, 787, 759, 716, 696, 658.

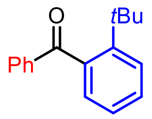

Chemical Formula: $C_{17}H_{18}O$

c34: Following the general procedure, 46 mg (0.5 mmol, 1 equiv.) toluene and 2.3 mL (15.0 mmol, 30 equiv.) *tert*-butylbenzene were used, the current was 10 mA. The crude mixture was purified by SiO_2 gel column chromatography with DCM. 9 mg product was obtained by 8% isolated yield as yellow oil.

^{1}H NMR (600 MHz, Chloroform-*d*) δ 7.89 – 7.86 (m, 1H), 7.83 – 7.79 (m, 2H), 7.65 – 7.62 (m, 1H), 7.61 – 7.56 (m, 2H), 7.48 (t, *J* = 7.8 Hz, 2H), 7.40 (t, *J* = 7.7 Hz, 1H), 1.36 (s, 9H).

^{13}C NMR (151 MHz, Chloroform-*d*) δ 197.27, 151.61, 138.01, 137.50, 132.44, 130.23, 129.68, 128.36, 128.02, 127.68, 127.02, 35.00, 31.40.

ESI-HRMS: mass spectrometry: m/z calc. 261.12499 $[C_{17}H_{18}ONa]^+$, measured 261.12476.

IR (neat, cm^{-1}): ṽ: 3300, 3060, 2961, 2906, 2870, 2327, 2086, 1917, 1851, 1732, 1656, 1602, 1448, 1403, 1365, 1313, 1276, 1179, 1154, 1105, 1074, 1023, 928, 848, 793, 750, 699, 667.

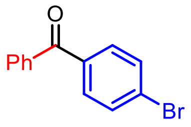

Chemical Formula: $C_{13}H_9BrO$

c35: Following the general procedure, 46 mg (0.5 mmol, 1 equiv.) toluene and 2.355 g (15.0 mmol, 30 equiv.) bromobenzene were used, the current was 10 mA. The crude mixture was purified by SiO_2 gel column chromatography with pentane/EA (from 50:1 to 40:1). 31.5 mg, 24%. See characterization data for **c2** (identical product obtained from a different disconnection).

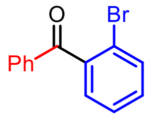

Chemical Formula: $C_{13}H_9BrO$

c36: Following the general procedure, 46 mg (0.5 mmol, 1 equiv.) toluene and 2.355 g (15.0 mmol, 30 equiv.) bromobenzene were used, the current was 10 mA. The crude mixture was purified by

SiO_2 gel column chromatography with pentane/EA (from 50:1 to 40:1). 35 mg product was obtained by 27% isolated yield as white solid.

^{1}H NMR (600 MHz, Chloroform-*d*) δ 7.82 (dd, J = 8.2 Hz, J = 1.1 Hz, 2H), 7.65 (d, J = 8.1 Hz, 1H), 7.61 (tt, J = 7.4 Hz, J = 1.2 Hz, 1H), 7.47 (≈ t, J ≈ 8.0 Hz, 2H), 7.42 (≈ td, J = 7.5 Hz, J = 1.1 Hz, 1H), 7.38-7.33 (m, 2H).

^{13}C NMR (151 MHz, Chloroform-*d*) δ 196.01, 140.84, 136.28, 133.87, 133.35, 131.29, 130.37, 129.13, 128.78, 127.33, 119.69.

ESI-HRMS: mass spectrometry: m/z calc. 282.97290 $[C_{13}H_9OBrNa]^+$, measured 282.97336.

IR (neat, cm^{-1}): ṽ: 3327, 3061, 2924, 2088, 1818, 1668, 1589, 1430, 1313, 1284, 1153, 1044, 1024, 927, 799, 762, 701, 664.

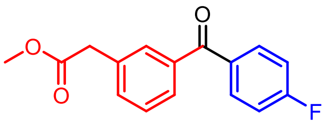

Chemical Formula: $C_{16}H_{13}FO_3$

c37: Following the general procedure, 82 mg (0.5 mmol, 1 equiv.) methyl 3-methylphenylacetate and 1.442 g (15.0 mmol, 30 equiv.) fluorobenzene were used, the current was 10 mA. The crude mixture was purified by SiO_2 gel column chromatography with pentane/EA (10:1). 55.5 mg product was obtained by 41% isolated yield as white solid.

^{1}H NMR (600 MHz, Chloroform-*d*) δ 7.85 (second order m, 2H), 7.69 (broad s, 1H), 7.66 (dt, J = 7.6 Hz, J = 1.5 Hz, 1H), 7.52 (dm, J = 7.7 Hz, 1H), 7.45 (t, J = 7.6 Hz, 1H), 7.16 (second order m, 2H), 3.71 (s, 3H), 3.70 (s, 2H).

^{13}C NMR (151 MHz, Chloroform-*d*) δ 194.95, 171.51, 165.45 (d, $^{1}J_{CF}$ = 254 Hz), 137.81, 134.41, 133.72 (d, $^{4}J_{CF}$ = 3 Hz), 133.40, 133.70 (d, $^{3}J_{CF}$ = 9 Hz), 130.76, 128.76, 128.59, 115.50 (d, $^{2}J_{CF}$ = 22 Hz), 52.19, 40.86.

^{19}F (565 MHz, Chloroform-*d*) δ -105.83 (m).

ESI-HRMS: mass spectrometry: m/z calc. 295.07409 $[C_{16}H_{13}O_3FNa]^+$, measured 295.07453.

IR (neat, cm^{-1}): ṽ: 3310, 2953, 1737, 1658, 1595, 1503, 1436, 1412, 1279, 1225, 1155, 1097, 1011, 971, 927, 851, 809, 752, 710.

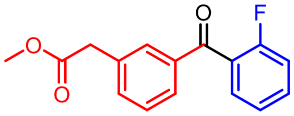

Chemical Formula: $C_{16}H_{13}FO_3$

c38: Following the general procedure, 82 mg (0.5 mmol, 1 equiv.) methyl 3-methylphenylacetate and 1.442 g (15.0 mmol, 30 equiv.) fluorobenzene were used, the current was 10 mA. The crude mixture was purified by SiO_2 gel column chromatography with pentane/EA (10:1). 19 mg product was obtained by 14% isolated yield as white solid.

^{1}H NMR (600 MHz, Chloroform-*d*) δ 7.79 (broad s, 1H), 7.45 (≈ dd, J = 7.7 Hz, J = 1.0 Hz, 1H), 7.59-7.53 (m, 3H), 7.46 (t, J = 7.7 Hz, 1H), 7.29 (td, J = 7.5 Hz, J = 0.8 Hz, 1H), 7.19 (broad t, J ≈ 9 Hz, 1H), 3.73 (s, 3H), 3.72 (s, 2H).

^{13}C NMR (151 MHz, Chloroform-*d*) δ 193.15, 171.47, 160.12 (d, $^{1}J_{CF}$ = 253 Hz), 137.70, 134.49, 134.35, 133.15 (d, $^{3}J_{CF}$ = 8 Hz), 130.81 (d, $^{4}J_{CF}$ = 3 Hz), 130.53, 128.86, 128.72, 126.94 (d, $^{2}J_{CF}$ = 15 Hz), 124.30 (d, $^{1}J_{CF}$ = 4 Hz), 116.32 (d, $^{2}J_{CF}$ = 22 Hz), 52.18, 40.87.

^{19}F (565 MHz, Chloroform-*d*) δ -111.00 (m).
ESI-HRMS: mass spectrometry: m/z calc. 295.07409 $[C_{16}H_{13}O_3FNa]^+$, measured 295.07442.
IR (neat, cm^{-1}): ṽ: 3456, 2953, 2851, 2327, 2089, 1736, 1665, 1607, 1482, 1447, 1339, 1296, 1216, 1158, 1102, 1011, 937, 852, 817, 755, 712.

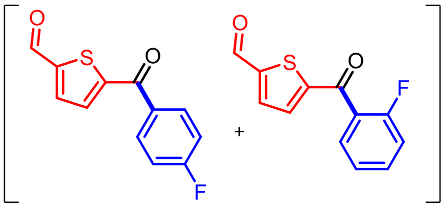

Chemical Formula: $C_{12}H_7FO_2S$

c39 + **c40** (10:1): Following the general procedure, 63 mg (0.5 mmol, 1 equiv.) 5-methylthiophene-2-carboxaldehyde and 1.442 g (15.0 mmol, 30 equiv.) fluorobenzene were used, the current was 10 mA. The crude mixture was purified by SiO_2 gel column chromatography with pentane/EA (10:1). 58 mg product was obtained by 49.5% isolated yield as white solid. Only the main (para) isomer NMR lines are reported:
^{1}H NMR (600 MHz, Chloroform-*d*) δ 10.04 (s, 1H), 7.95 (second order m, 2H), 7.82 (d, J = 4.0 Hz, 1H), 7.71 (d, J = 4.0 Hz, 1H), 7.23 (second order m, 2H).
^{13}C NMR (151 MHz, Chloroform-*d*) δ 186.47, 183.32, 165.78 (d, $^1J_{CF}$ = 256 Hz), 149.30, 148.35, 134.89, 133.77, 133.29 (d, $^4J_{CF}$ = 3 Hz), 132.04 (d, $^3J_{CF}$ = 9 Hz), 115.97 (d, $^2J_{CF}$ = 22 Hz).
^{19}F (565 MHz, Chloroform-*d*) δ -104.36 (m).
ESI-HRMS: mass spectrometry: m/z calc. 257.00430 $[C_{12}H_7O_2FNaS]^+$, measured 257.00421.
IR (neat, cm^{-1}): ṽ: 3084, 2922, 2858, 2325, 1876, 1673, 1632, 1596, 1503, 1452, 1405, 1286, 1208, 1154,

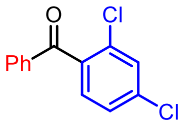

Chemical Formula: $C_{13}H_8Cl_2O$

c41: Following the general procedure, 46 mg (0.5 mmol, 1 equiv.) toluene and 1.70 mL (15.0 mmol, 30 equiv.) 1,3-dichlorobenzene were used, the current was 10 mA. The crude mixture was purified by SiO_2 gel column chromatography with DCM. 80 mg product was obtained by 64% isolated yield as white solid.
^{1}H NMR (600 MHz, Chloroform-*d*) δ 7.79 (dd, J = 8.3, 1.4 Hz, 2H), 7.62 (t, J = 7.4 Hz, 1H), 7.53 – 7.45 (m, 3H), 7.39 – 7.31 (m, 2H).
^{13}C NMR (151 MHz, Chloroform-*d*) δ 194.42, 137.12, 136.82, 136.41, 134.06, 132.61, 130.32, 130.20, 130.17, 128.86, 127.28.
ESI-HRMS: mass spectrometry: m/z calc. 272.98444 $[C_{13}H_8OCl_2Na]^+$, measured 272.98416.
IR (neat, cm^{-1}): ṽ: 3858, 3331, 3065, 2927, 2660, 2323, 2086, 1998, 1907, 1671, 1584, 1554, 1449, 1373, 1313, 1280, 1244, 1186, 1150, 1102, 1056, 1001, 928, 868, 827, 800, 731, 698, 663.

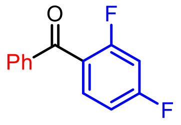

Chemical Formula: $C_{13}H_8F_2O$

c42: Following the general procedure, 46 mg (0.5 mmol, 1 equiv.) toluene and 1.47 mL (15.0 mmol, 30 equiv.) 1,3-difluorobenzene were used, the current was 10 mA. The crude mixture was purified by SiO_2 gel column chromatography with DCM. 78 mg product was obtained by 72% isolated yield as yellow oil.

^{1}H NMR (600 MHz, Chloroform-*d*) δ 7.81 (d, *J* = 8.0 Hz, 2H), 7.64 – 7.56 (m, 2H), 7.48 (t, *J* = 7.7 Hz, 2H), 7.01 (td, *J* = 8.3, 2.4 Hz, 1H), 6.91 (td, *J* = 9.4, 2.4 Hz, 1H).

^{13}C NMR (151 MHz, Chloroform-*d*) δ 192.42, 165.05 (dd, *J* = 253.7, 11.0 Hz), 161.09 (dd, *J* = 255.6, 12.2 Hz), 137.56, 133.60, 132.68 (dd, *J* = 10.3, 4.3 Hz), 129.83, 128.66, 123.52 (dd, *J* = 14.5, 3.6 Hz), 112.01 (dd, *J* = 21.2, 4.1 Hz), 104.82 (t, *J* = 26.0 Hz).

^{19}F NMR (565 MHz, Chloroform-*d*) δ -103.77 – -103.84 (m, 1F), -105.79 – -105.88 (m, 1F).

ESI-HRMS: mass spectrometry: m/z calc. 241.04354 $[C_{13}H_8OF_2Na]^+$, measured 241.04313.

IR (neat, cm^{-1}): ṽ: 3323, 3074, 2931, 2331, 2194, 1910, 1665, 1606, 1497, 1447, 1424, 1266, 1179, 1142, 1096, 1027, 1001, 970, 917, 852, 794, 734, 696.

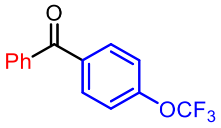

Chemical Formula: $C_{14}H_9F_3O_2$

c43: Following the general procedure, 46 mg (0.5 mmol, 1 equiv.) toluene and 1.98 mL (15.0 mmol, 30 equiv.) trifluoromethoxybenzene were used, the current was 5 mA. The crude mixture was purified by SiO_2 gel column chromatography with DCM. 85 mg product was obtained by 64% isolated yield as white solid.

^{1}H NMR (600 MHz, Chloroform-*d*) δ 7.91 – 7.84 (m, 2H), 7.82 – 7.76 (m, 2H), 7.61 (t, *J* = 7.4 Hz, 1H), 7.50 (t, *J* = 7.6 Hz, 2H), 7.32 (d, *J* = 8.3 Hz, 2H).

^{13}C NMR (151 MHz, Chloroform-*d*) δ 195.31, 152.31, 137.31, 136.03, 132.87, 132.12, 130.10, 128.59, 120.50 (q, *J* = 258.1 Hz), 120.39.

^{19}F NMR (565 MHz, Chloroform-*d*) δ -57.59 (s, 3F).

ESI-HRMS: mass spectrometry: m/z calc. 267.06274 $[C_{14}H_{10}O_2F_3]^+$, measured 267.06192.

IR (neat, cm^{-1}): ṽ: 3295, 3067, 2931, 2325, 2159, 1992, 1921, 1652, 1598, 1500, 1448, 1409, 1256, 1212, 1160, 1014, 967, 920, 854, 792, 740, 695.

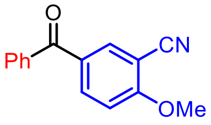

Chemical Formula: $C_{15}H_{11}NO_2$

c44: Following the general procedure, 46 mg (0.5 mmol, 1 equiv.) toluene and 1.83 mL (15.0 mmol, 30 equiv.) 2-methoxybenzonitrile were used, the current was 5 mA. The crude mixture was purified

by SiO_2 gel column chromatography with pentane/EA (from 10:1 to 4:1). 42 mg product was obtained by 35% isolated yield as brown solid.

^{1}H NMR (600 MHz, Chloroform-*d*) δ 8.09 (dd, *J* = 8.8, 2.1 Hz, 1H), 8.05 (d, *J* = 2.2 Hz, 1H), 7.74 (d, *J* = 7.6 Hz, 2H), 7.62 (t, *J* = 7.5 Hz, 1H), 7.51 (t, *J* = 7.6 Hz, 2H), 7.08 (d, *J* = 8.8 Hz, 1H), 4.04 (s, 3H).

^{13}C NMR (151 MHz, Chloroform-*d*) δ 193.74, 164.18, 137.15, 136.70, 136.47, 132.88, 130.59, 129.84, 128.72, 115.63, 111.30, 102.18, 56.74.

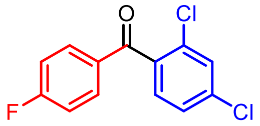

Chemical Formula: $C_{13}H_7Cl_2FO$

c45: Following the general procedure, 55 mg (0.5 mmol, 1 equiv.) 4-fluorotoluene and 2.205 g (15.0 mmol, 30 equiv.) 1,3-dichlorobenzene were used, the current was 10 mA. The crude mixture was purified by SiO_2 gel column chromatography with pentane/EA (from 50:1 to 40:1). 64.5 mg product was obtained by 48% isolated yield as colorless oil.

^{1}H NMR (600 MHz, Chloroform-*d*) δ 7.82 (second order m, 2H), 7.50 (d, *J* = 1.8 Hz, 1H), 7.38 (dd, *J* = 8.2 Hz, *J* = 1.8 Hz, 1H), 7.33 (d, *J* = 8.2 Hz, 1H), 7.15 (second order m, 2H).

^{13}C NMR (151 MHz, Chloroform-*d*) δ 192.71, 166.27 (d, $^1J_{CF}$ = 257 Hz), 136.84, 136.70, 132.72 (d, $^3J_{CF}$ = 9 Hz), 132.72, 132.34, 130.10, 130.07, 127.27, 116.00 (d, $^2J_{CF}$ = 22 Hz).

^{19}F (565 MHz, Chloroform-*d*) δ -103.31 (m).

ESI-HRMS: mass spectrometry: m/z calc. 290.97502 $[C_{13}H_7OCl_2FNa]^+$, measured 290.97522.

IR (neat, cm^{-1}): ṽ: 3873, 3080, 2928, 2865, 2325, 2086, 1915, 1672, 1592, 1466, 1373, 1281, 1237, 1148, 1101, 1013, 928, 826, 791, 765, 681.

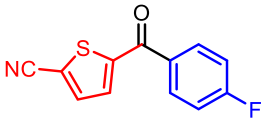

Chemical Formula: $C_{12}H_6FNOS$

c46: Following the general procedure, 62 mg (0.5 mmol, 1 equiv.) 5-methylthiophene-2-carbonitrile and 1.442 g (15.0 mmol, 30 equiv.) fluorobenzene were used, the current was 10 mA. The crude mixture was purified by SiO_2 gel column chromatography with pentane/EA (10:1). 56 mg product was obtained by 48.4% isolated yield as white solid.

^{1}H NMR (600 MHz, Chloroform-*d*) δ 7.90 (second order m, 2H), 7.65 (d, *J* = 4.0 Hz, 1H), 7.59 (d, *J* = 4.0 Hz, 1H), 7.21 (≈ t, *J* ≈ 8.5 Hz, 2H).

^{13}C NMR (151 MHz, Chloroform-*d*) δ 185.30, 165.85 (d, $^1J_{CF}$ = 256 Hz), 148.68, 137.31, 132.96, 132.91 (d, $^4J_{CF}$ = 3 Hz), 131.99 (d, $^3J_{CF}$ = 9 Hz), 116.50, 116.12 (d, $^2J_{CF}$ = 22 Hz), 113.22.

^{19}F (565 MHz, Chloroform-*d*) δ -103.81 (m).

ESI-HRMS: mass spectrometry: m/z calc. 254.00463 $[C_{12}H_6ONFNaS]^+$, measured 254.00492.

IR (neat, cm^{-1}): ṽ: 3198, 3100, 2230, 1850, 1623, 1597, 1503, 1432, 1327, 1293, 1236, 1136, 1099, 1052, 1011, 975, 951, 876, 839. 749, 695.

5. Synthesis of the Ketoprofen drugs.

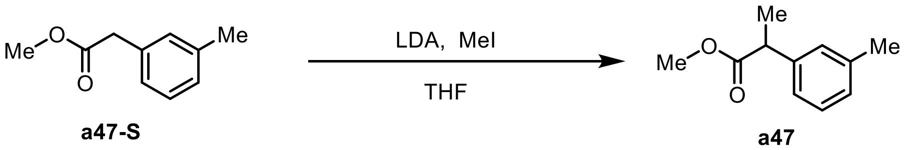

To a solution of methyl 3-methylphenylacetat **a47-S** (1.64 g, 10 mmol) in dry THF (20 mL) was added dropwise 7 mL (14 mmol) lithium diisopropylamide (LDA, 2 M in THF) at -78 °C. The mixture was stirred for 5 minutes at the same temperature. Then, iodomethane (1.99 g, 14 mmol) was added and the mixture was warmed to 0 °C over 1 hour. After quenching with saturated NH_4Cl solution, the mixture was extracted with ethyl acetate. The organic layer was washed with water and brine and dried over anhydrous Mg_2SO_4. After removal of the solvent, the residue was purified by silica gel column chromatography (pentane/EA = 30/1). 1.60 g product **a47** was obtained by 90% yield as a colorless oil.

^{1}H NMR (600 MHz, Chloroform-*d*) δ 7.25 (t, *J* = 7.6 Hz, 1H), 7.17 – 7.08 (m, 3H), 3.73 (q, *J* = 7.2 Hz, 1H), 3.70 (s, 3H), 2.39 (s, 3H), 1.53 (d, *J* = 7.2, 3H).

^{13}C NMR (151 MHz, Chloroform-*d*) δ 175.17, 140.62, 138.38, 128.64, 128.27, 128.01, 124.59, 52.07, 45.45, 21.50, 18.72.

APCI-HRMS: mass spectrometry: m/z calc. 179.10666 $[C_{11}H_{15}O_2]^+$, measured 179.10726.

IR (neat, cm^{-1}): ṽ: 3455, 2980, 2948, 2326, 2082, 2007, 1944, 1734, 1606, 1488, 1453, 1376, 1334, 1241, 1194, 1166, 1067, 1024, 971, 891, 846, 776, 700.

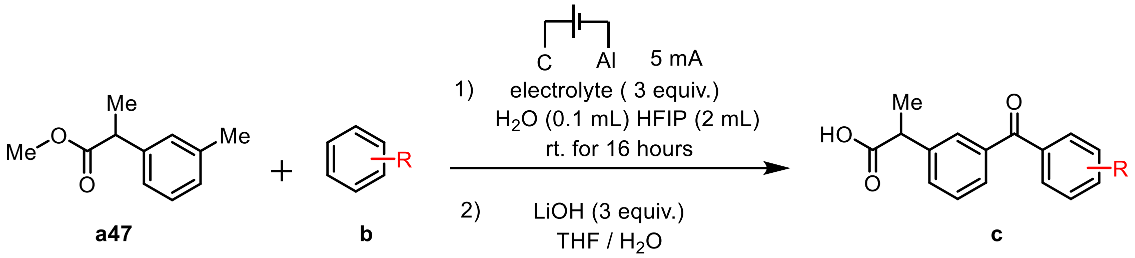

The methyl arene **a47** (0.5 mmol), the arene **b** (15.0 mmol), the electrolyte (1.5 mmol), and water (0.1 mL) are added into the solvent HFIP (2.0 mL) in a reaction vial. The reaction vial is then sealed with an aluminous headspace cap with electrodes (see the pictures in **3-1**). The current is set to 10 mA. Keep the reaction stirring at room temperature for 16 hours. After that, the solvent is removed and the crude is directly engaged on SiO_2 gel column chromatography for purification (pentane/EA = 50/1 to 20/1). The obtained product was dissolved in a 1:1mixture of THF/water (5 mL) to which lithium hydroxide (24 mg, 1.0 mmol) was added. After stirring for 24 hours at room temperature, the THF was evaporated in vacuo and 1M HCl was added until the pH of the solution was 2. Ethyl acetate was then added to the solution to extract the product. The water layer was washed by the ethyl acetate once. The combined organic layer was dried over anhydrous Mg_2SO_4. After removal of the solvent, product **c** was obtained.

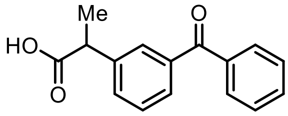

Chemical Formula: $C_{16}H_{14}O_3$

c47 According to the procedure above, 89 mg (0.5 mmol, 1 equiv.) methyl 2-(*m*-tolyl)propanoate **a47** and 1.33 mL (15 mmol, 30 equiv.) benzene were used. 84 mg product **c47** was obtained by 66% isolated yield as white solid.

^{1}H NMR (600 MHz, Chloroform-*d*) δ 7.82 – 7.77 (m, 3H), 7.69 (d, *J* = 7.7 Hz, 1H), 7.62 – 7.55 (m, 2H), 7.50 – 7.42 (m, 3H), 3.83 (q, *J* = 7.2 Hz, 1H), 1.56 (d, *J* = 7.2 Hz, 3H).

^{13}C NMR (151 MHz, Chloroform-*d*) δ 196.60, 179.45, 140.27, 138.12, 137.59, 132.69, 131.77, 130.25, 129.48, 129.42, 128.76, 128.47, 45.26, 18.29.

ESI-HRMS: mass spectrometry: m/z calc. 277.08352 $[C_{16}H_{14}O_3Na]^+$, measured 277.08342.

IR (neat, cm^{-1}): ṽ: 3061, 2980, 2935, 2728, 2627, 2531, 2325, 2086, 2019, 1919, 1705, 1655, 1595, 1448, 1411, 1382, 1282, 1226, 1176, 1073, 1000, 955, 913, 856, 821, 785, 700.

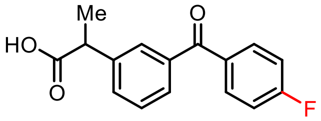

Chemical Formula: $C_{16}H_{13}FO_3$

c48 According to the procedure above, 89 mg (0.5 mmol, 1 equiv.) methyl 2-(*m*-tolyl)propanoate **a47** and 1.40 mL (15 mmol, 30 equiv.) fluorobenzene were used. 63 mg product **c48** was obtained by 46% isolated yield as white solid.

^{1}H NMR (600 MHz, Chloroform-*d*) δ 7.87 – 7.81 (m, 2H), 7.74 (s, 1H), 7.65 (d, *J* = 7.7, 1H), 7.56 (d, *J* = 7.7, 1H), 7.45 (t, *J* = 7.7 Hz, 1H), 7.18 – 7.12 (m, 2H), 3.82 (q, *J* = 7.2 Hz, 1H), 1.55 (d, *J* = 7.2 Hz, 3H).

^{13}C NMR (151 MHz, Chloroform-*d*) δ 195.14, 179.96, 165.61 (d, *J* = 254.6 Hz), 140.34, 138.00, 133.78, 132.86 (d, *J* = 9.0 Hz), 131.85, 129.33, 129.19, 128.82, 115.65 (d, *J* = 21.9 Hz), 45.31, 18.24.

^{19}F NMR (565 MHz, Chloroform-*d*) δ -105.62 – -105.70 (m, 1F).

ESI-HRMS: mass spectrometry: m/z calc. 295.07409 $[C_{16}H_{13}O_3FNa]^+$, measured 295.07392.

IR (neat, cm^{-1}): ṽ: 3867, 3645, 3455, 3313, 3073, 2977, 2937, 2703, 2522, 2326, 2208, 2171, 2079, 1986, 1920, 1708, 1657, 1595, 1504, 1455, 1408, 1375, 1282, 1228, 1155, 1096, 1012, 956, 912, 851, 754, 704.

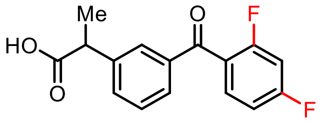

Chemical Formula: $C_{16}H_{12}F_2O_3$

c49 According to the procedure above, 89 mg (0.5 mmol, 1 equiv.) methyl 2-(*m*-tolyl)propanoate **a47** and 1.47 mL (15 mmol, 30 equiv.) 1,3-difluorobenzene were used. 74 mg product **c49** was obtained by 51% isolated yield as white solid.

^{1}H NMR (600 MHz, Chloroform-*d*) δ 7.81 – 7.78 (m, 1H), 7.70 – 7.66 (m, 1H), 7.62 – 7.55 (m, 2H), 7.44 (t, *J* = 7.7 Hz, 1H), 6.99 (td, *J* = 8.2, 2.4 Hz, 1H), 6.89 (td, *J* = 9.4, 2.4 Hz, 1H), 3.81 (q, *J* = 7.2 Hz, 1H), 1.54 (d, *J* = 7.1 Hz, 3H).

^{13}C NMR (151 MHz, Chloroform-*d*) δ 192.11, 180.01, 165.13 (dd, *J* = 256.7, 11.1 Hz), 161.12 (dd, *J* = 256.7, 12.1 Hz), 140.46, 137.85, 132.85, 132.76 (dd, *J* = 9.1, 4.5 Hz), 129.15, 129.00, 123.31 (dd, *J* = 14.0, 4.2 Hz), 112.05 (dd, *J* = 21.3, 3.9 Hz), 104.86 (t, *J* = 25.9 Hz), 45.26, 18.21.
^{19}F NMR (565 MHz, Chloroform-*d*) δ -102.58 – -103.93 (m, 1F), -105.06 – -106.37 (m, 1F).
ESI-HRMS: mass spectrometry: m/z calc. 313.06467 $[C_{16}H_{12}O_3F_2Na]^+$, measured 313.06419.
IR (neat, cm^{-1}): ṽ: 3080, 2980, 2938, 2727, 2634, 2527, 2164, 2108, 1707, 1663, 1606, 1496, 1425, 1266, 1141, 1096, 973, 913, 853, 751, 696.

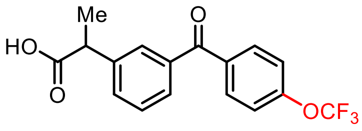

Chemical Formula: $C_{17}H_{13}F_3O_4$

c50 According to the procedure above, 89 mg (0.5 mmol, 1 equiv.) methyl 2-(*m*-tolyl)propanoate **a47** and 1.98 mL (15 mmol, 30 equiv.) trifluoromethoxybenzene were used. 73 mg product **c50** was obtained by 43% isolated yield as white solid.
^{1}H NMR (600 MHz, Chloroform-*d*) δ 7.88 – 7.83 (m, 2H), 7.77 (s, 1H), 7.69 – 7.65 (m, 1H), 7.58 (d, *J* = 7.8 Hz, 1H), 7.46 (t, *J* = 7.7 Hz, 1H), 7.31 (d, *J* = 7.7 Hz, 2H), 3.84 (q, *J* = 7.2 Hz, 1H), 1.57 (d, *J* = 7.2 Hz, 3H).
^{13}C NMR (151 MHz, Chloroform-*d*) δ 194.90, 179.95, 152.29, 140.31, 137.53, 135.68, 132.05, 131.98, 129.24, 129.18, 128.78, 120.36 (q, *J* = 259.1 Hz), 120.26, 45.19, 18.12.
^{19}F NMR (565 MHz, Chloroform-*d*) δ -57.56 (s, 3F).
ESI-HRMS: mass spectrometry: m/z calc. 361.06581 $[C_{17}H_{13}O_4F_3Na]^+$, measured 361.06578.
IR (neat, cm^{-1}): ṽ: 2984, 2624, 2161, 1708, 1771, 1599, 1503, 1458, 1412, 1250, 1208, 1163, 1015, 957, 923, 858, 756, 705.

6. Selected unsuccessful substrates.

For the following methyl arenes and arenes, the corresponding coupling products with respectively benzene and toluene could not be found nor isolated:

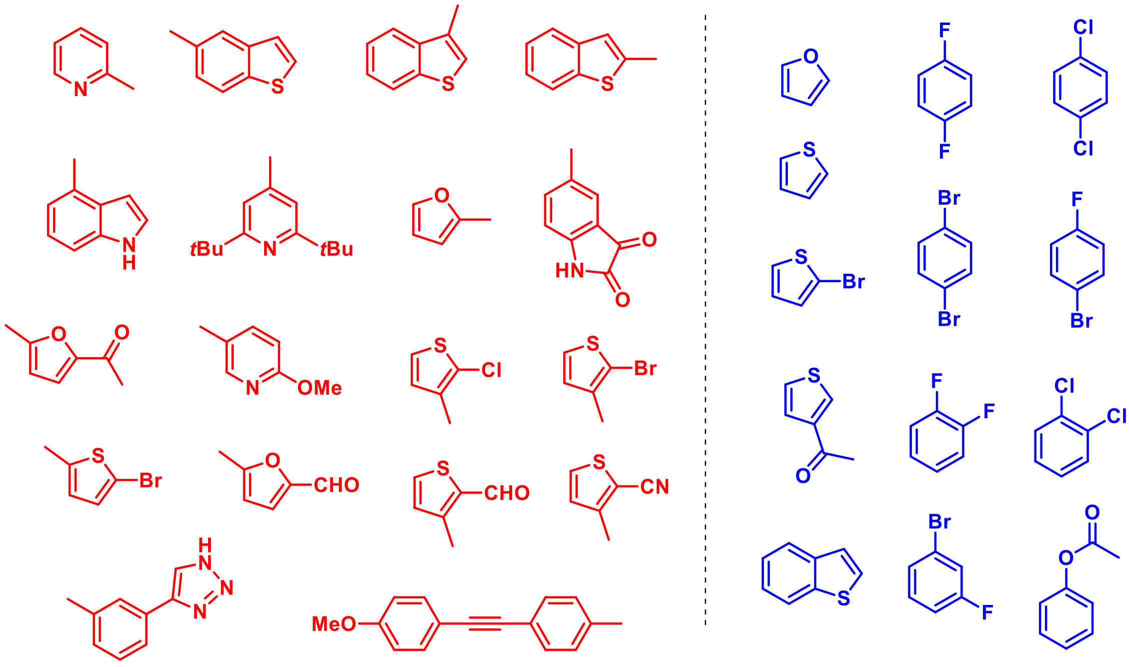

7. Mechanistic studies.

(1) Synthesis of some intermediates.

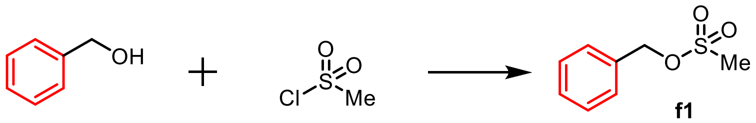

f1

f1 Benzyl alcohol (1.08 g, 10.0 mmol) and (triethyl)amine (2.8 mL, 20 mmol) were added to DCM (50 mL) in a 250 mL flask. The mixture was cooled to -40 °C, and methanesulfonyl chloride (1.1 mL, 14.0 mmol) was added to the mixture while being stirred vigorously. After 30 min at -40 °C, the reaction mixture was washed with a 1% HCl solution (3 × 100 mL) and a saturated aqueous $NaHCO_3$ solution (3 × 50 mL). The organic layer was dried (Na_2SO_4), filtered and the solvent was removed in vacuo. 1.7 g product **f1** (colorless oil) was obtained with a yield of 91%.
^{1}H NMR (600 MHz, Chloroform-*d*) δ 7.49 – 7.32 (m, 5H), 5.24 (s, 2H), 2.91 (s, 3H).
^{13}C NMR (151 MHz, Chloroform-*d*) δ 133.51, 129.53, 129.04, 129.00, 71.66, 38.46.

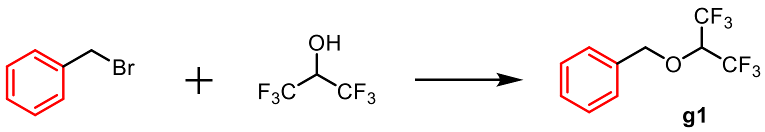

g1

g1 K_2CO_3 (3.9 g, 28 mmol), DMF (10 mL), HFIP (3 mL, 28 mmol), and benzyl bromide (1 mL, 8.3 mmol) were added into a 50 mL vial in order. Keep stirring the mixture at room temperature for 5 min and then close the vial with an aluminum cap. The mixture was thereafter stirred under 100 °C overnight. The reaction liquid layer was then taken out by a pipette without quenching the reaction. The liquid was washed with water and extracted by Et_2O. The solvent was then removed to get the

crude product. After a purification by SiO_2 gel column chromatography with pentane/EA (from 50:1 to 30:1), 1.4 g product was obtained by 65% isolated yield as a colorless oil.

^{1}H NMR (600 MHz, Chloroform-*d*) δ 7.48 – 7.32 (m, 5H), 4.89 (s, 2H), 4.14 (hept, *J* = 6.0 Hz, 1H).

^{13}C NMR (151 MHz, Chloroform-*d*) δ 134.84, 129.18, 128.93, 128.84, 125.95 – 118.17 (m), 76.17, 74.68 (h, *J* = 32.6 Hz).

^{19}F NMR (565 MHz, Chloroform-*d*) δ -73.60.

(2) Identification of the reaction intermediates.

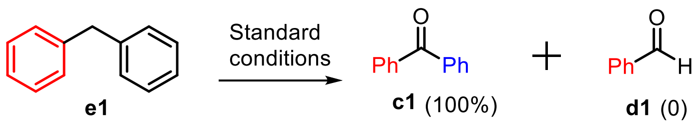

Following the general procedure, 84 mg (0.5 mmol, 1 equiv.) diphenylmethane **e1** and 1.33 mL (15.0 mmol, 30 equiv.) benzene were used, the current was 10 mA. After the reaction is done, the yield was determined by ^{1}H NMR analysis of the crude reaction mixture using 1,3,5-trimethoxybenzene as an internal standard.

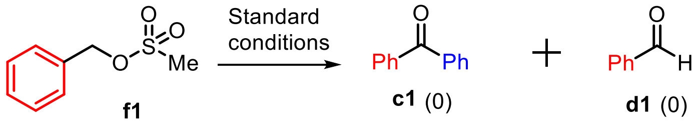

Following the general procedure, 93 mg (0.5 mmol, 1 equiv.) benzyl methanesulfonate **f1** and 1.33 mL (15.0 mmol, 30 equiv.) benzene were used, the current was 10 mA. After the reaction is done, the yield was determined by ^{1}H NMR analysis of the crude reaction mixture using 1,3,5-trimethoxybenzene as an internal standard.

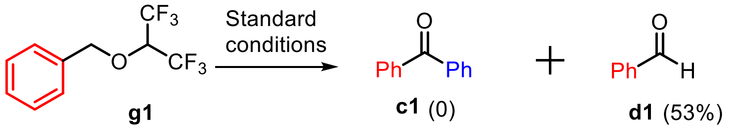

Following the general procedure, 129 mg (0.5 mmol, 1 equiv.) **g1** and 1.33 mL (15.0 mmol, 30 equiv.) benzene were used, the current was 10 mA. After the reaction is done, the yield was determined by ^{1}H NMR analysis of the crude reaction mixture using 1,3,5-trimethoxybenzene as an internal standard.

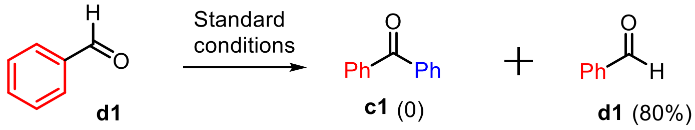

Following the general procedure, 53 mg (0.5 mmol, 1 equiv.) benzaldehyde **d1** and 1.33 mL (15.0 mmol, 30 equiv.) benzene were used, the current was 10 mA. After the reaction is done, the yield was determined by ^{1}H NMR analysis of the crude reaction mixture using 1,3,5-trimethoxybenzene as an internal standard.

(3) Solvent tests.

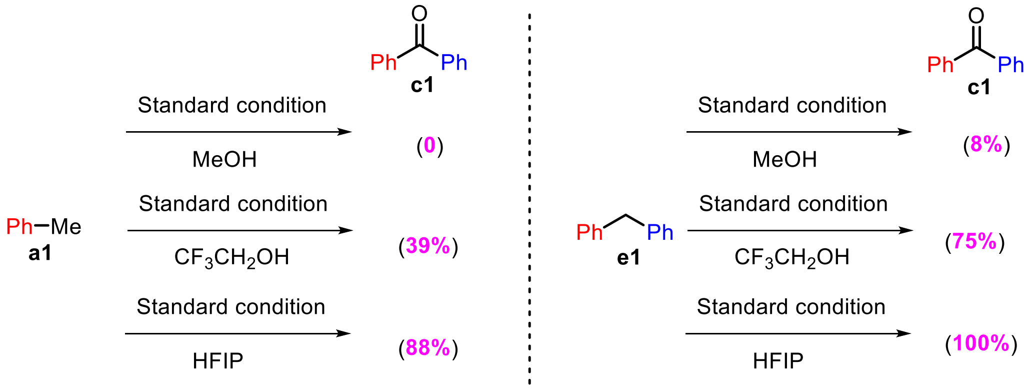

Following the general procedure, 46 mg (0.5 mmol, 1 equiv.) toluene **a1** or 84 mg (0.5 mmol, 1 equiv.) diphenylmethane **e1** was used to react with 1.33 mL (15.0 mmol, 30 equiv.) benzene with different solvents. The current was 10 mA. The yields were determined by ^{1}H NMR analysis of the crude reaction mixture using 1,3,5-trimethoxybenzene as an internal standard.

(4) Recovery of the electrolyte

Following the general procedure, 46 mg (0.5 mmol, 1 equiv.) toluene and 1.33 mL (15.0 mmol, 30 equiv.) benzene were used, the current was 10 mA. The crude mixture was purified by SiO_2 gel column chromatography with EA/EtOH (25:1). 410 mg electrolyte was recovered, thus 78% isolated yield.

(5) ^{17}O NMR experiment.

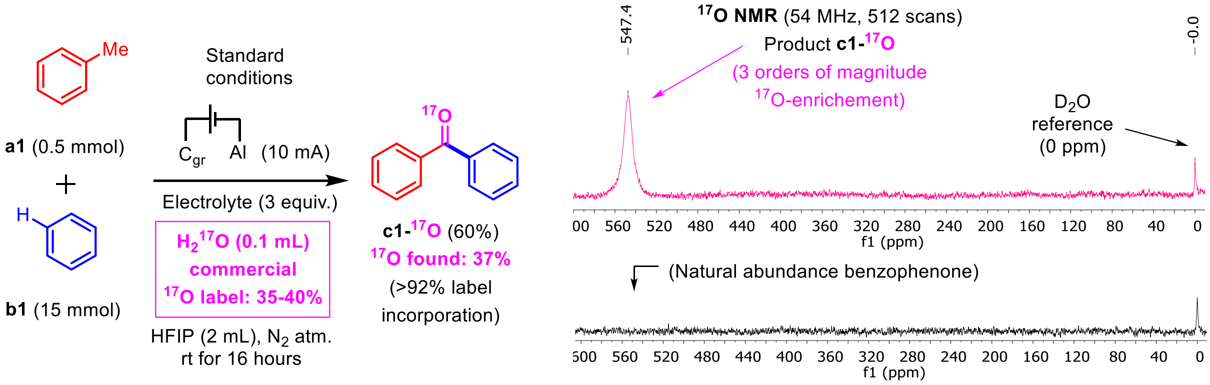

^{17}O NMR (54 MHz, 299 K, 512 scans, relaxation delay: 0.2 s, see NMR profile above. The solvent used for shimming is pure D_2O, which also serves as ^{17}O reference at 0 ppm. The D_2O is placed inside a sealed capillary, itself placed inside a standard NMR tube. The NMR tube is then loaded with a CH_2Cl_2 solution of ^{17}O-labeled benzophenone, **c1**-^{17}O) δ +547 ppm.

ESI-HRMS: mass spectrometry: m/z calc. 206.06660 $[C_{13}H_{10}Na^{17}O]^+$, measured 206.06618:

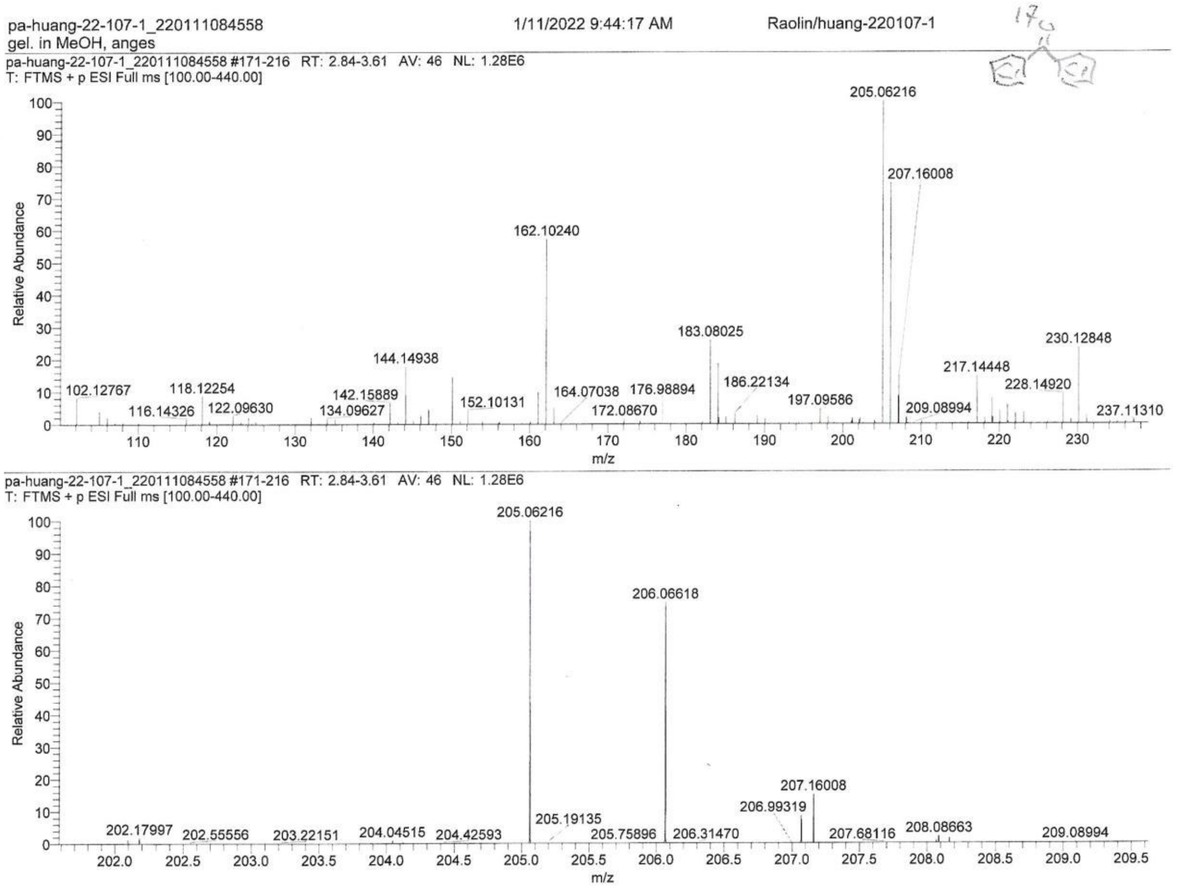

8. Copies of ¹H and ¹³C Spectra.

¹H NMR (**c1**)

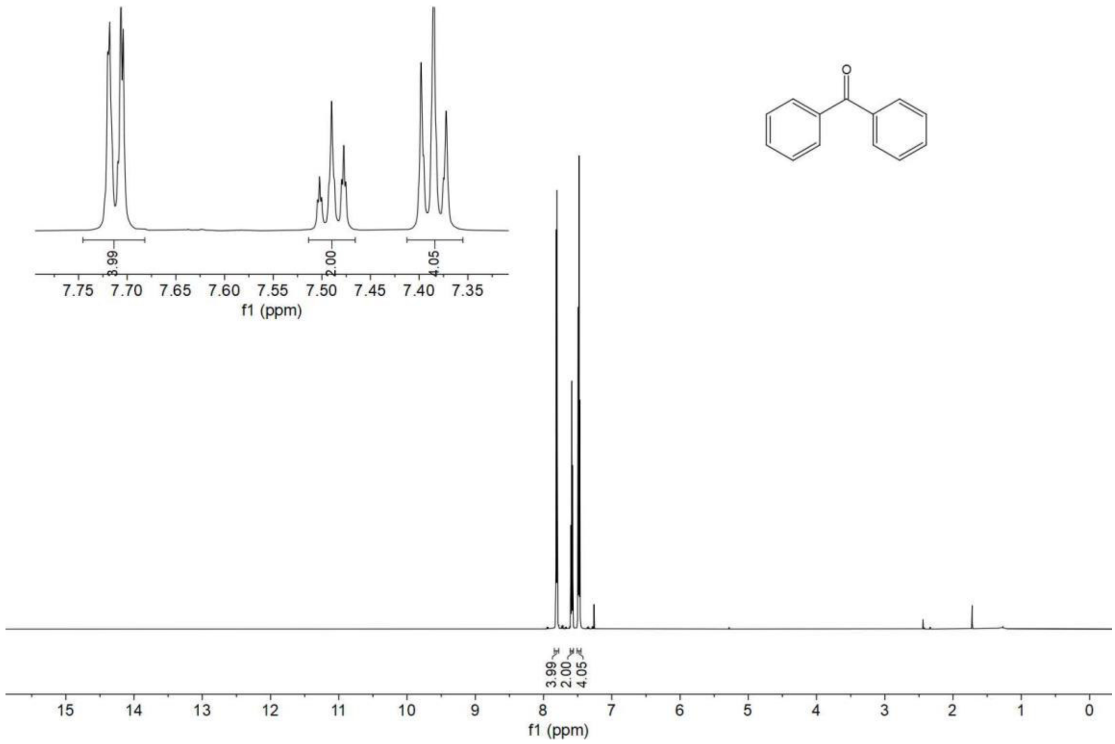

¹³C NMR (**c1**)

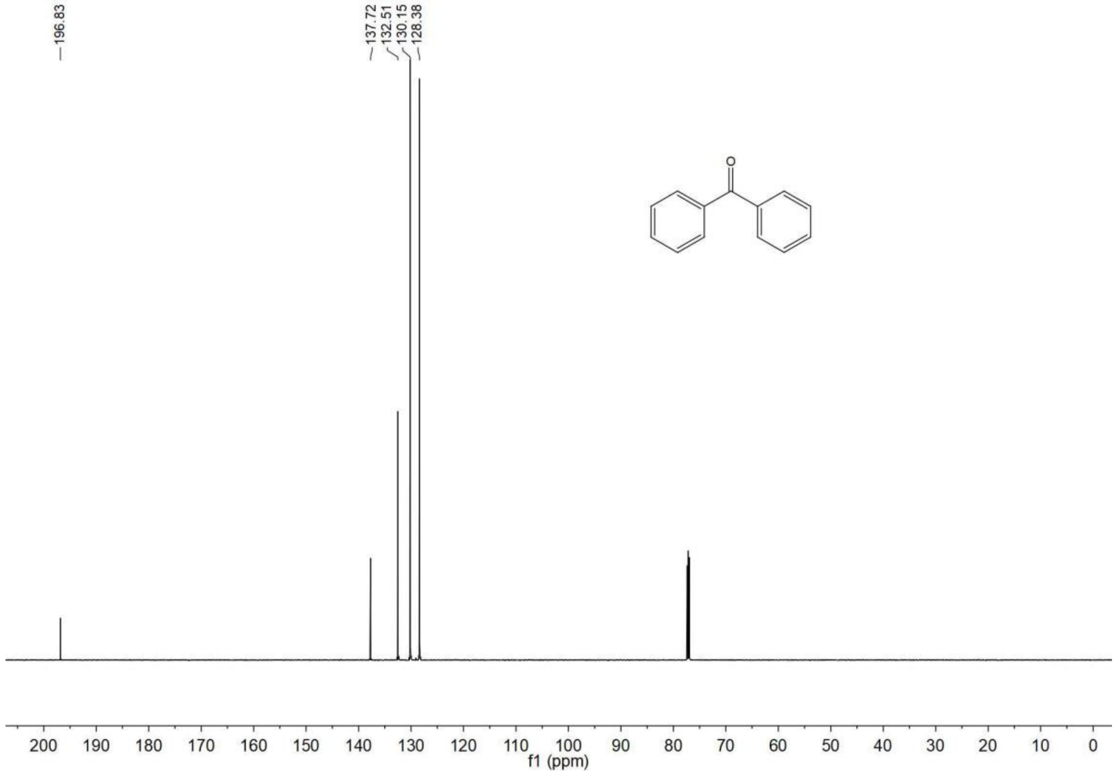

^{1}H NMR (**c2**)

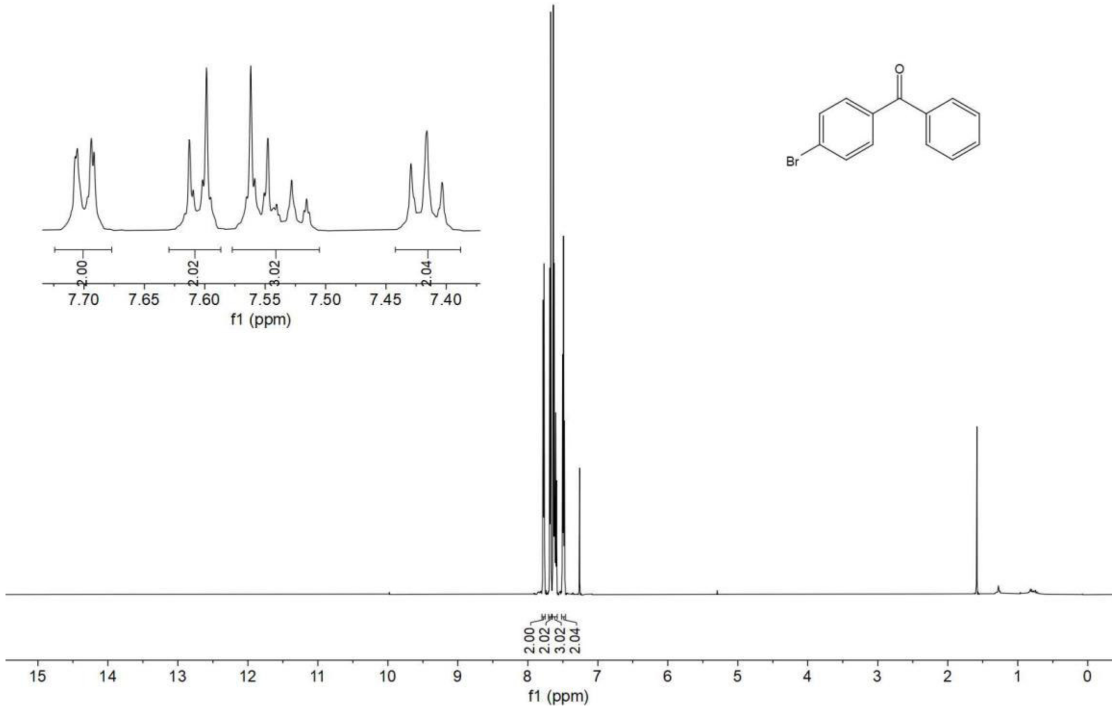

^{13}C NMR (**c2**)

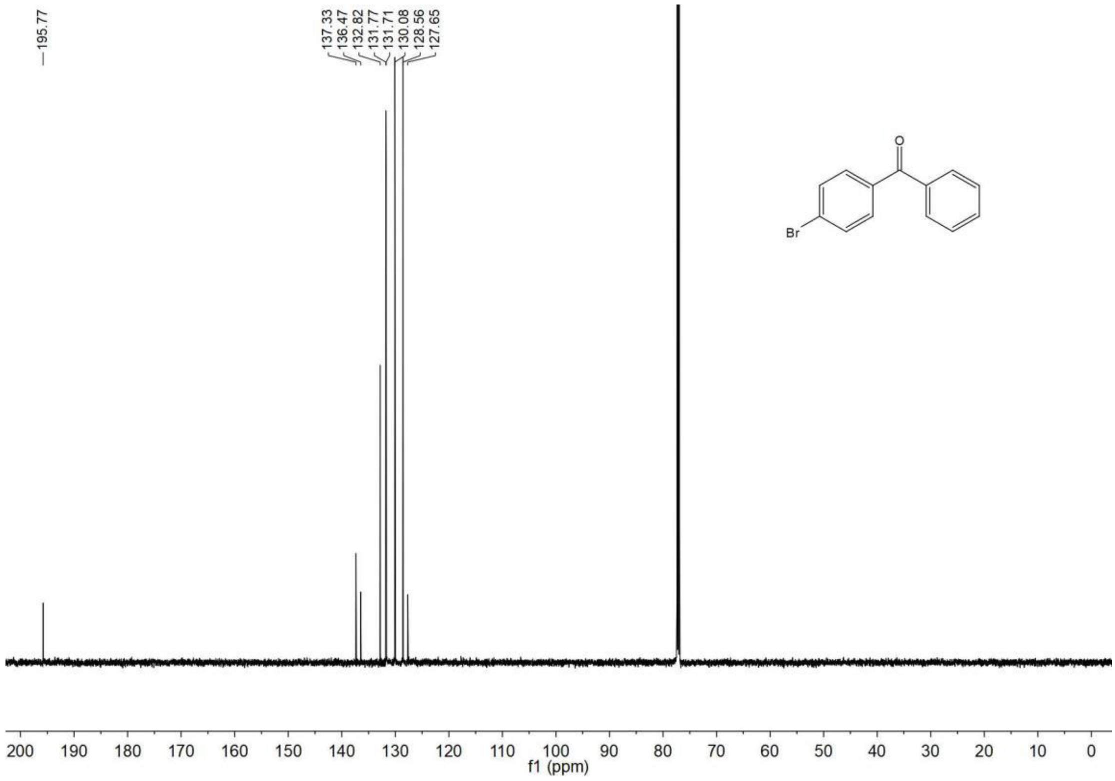

^{1}H NMR (**c3**)

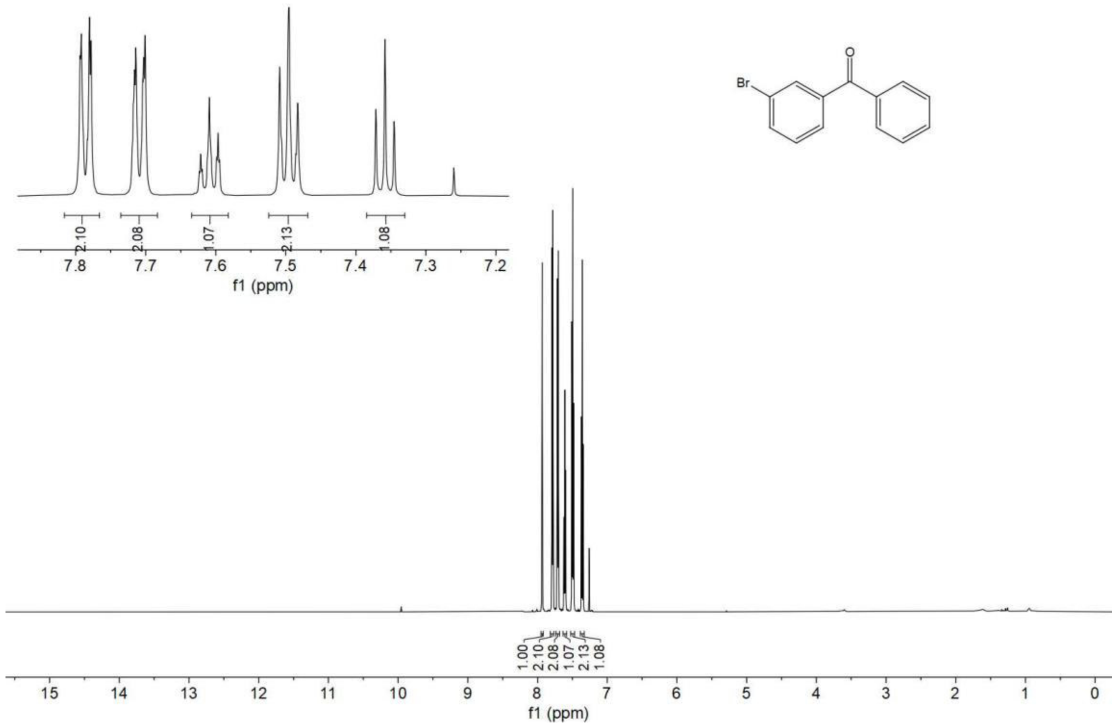

^{13}C NMR (**c3**)

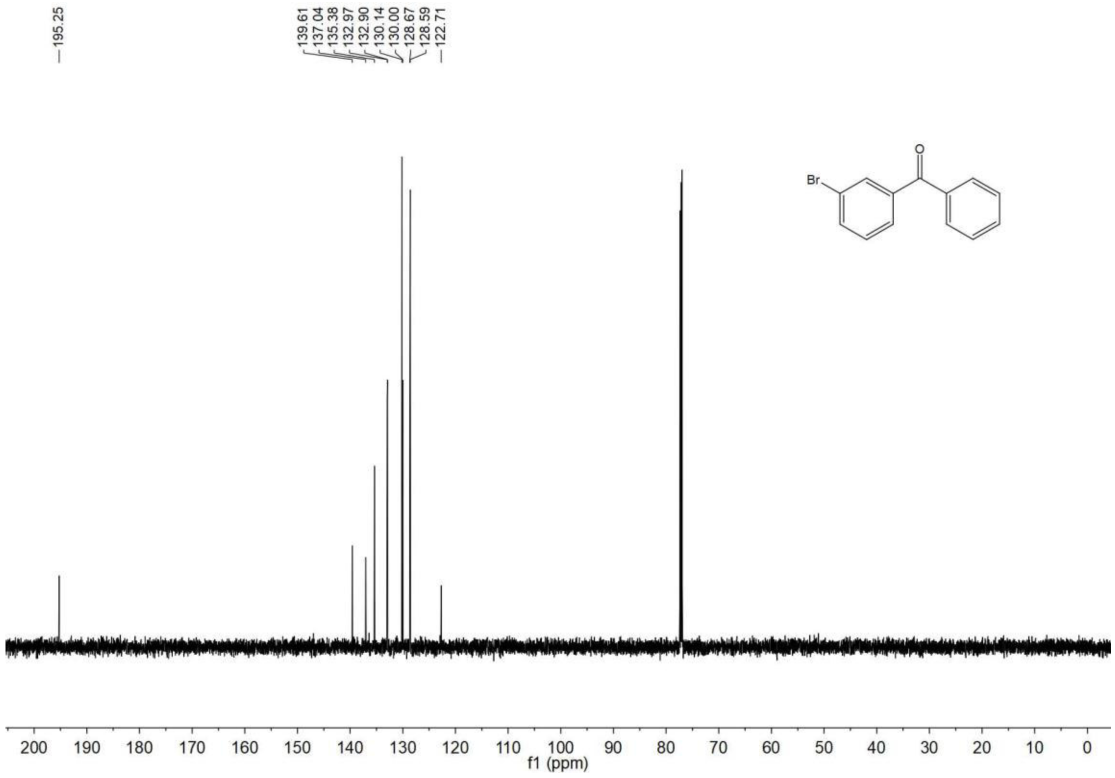

^{1}H NMR (**c4**)

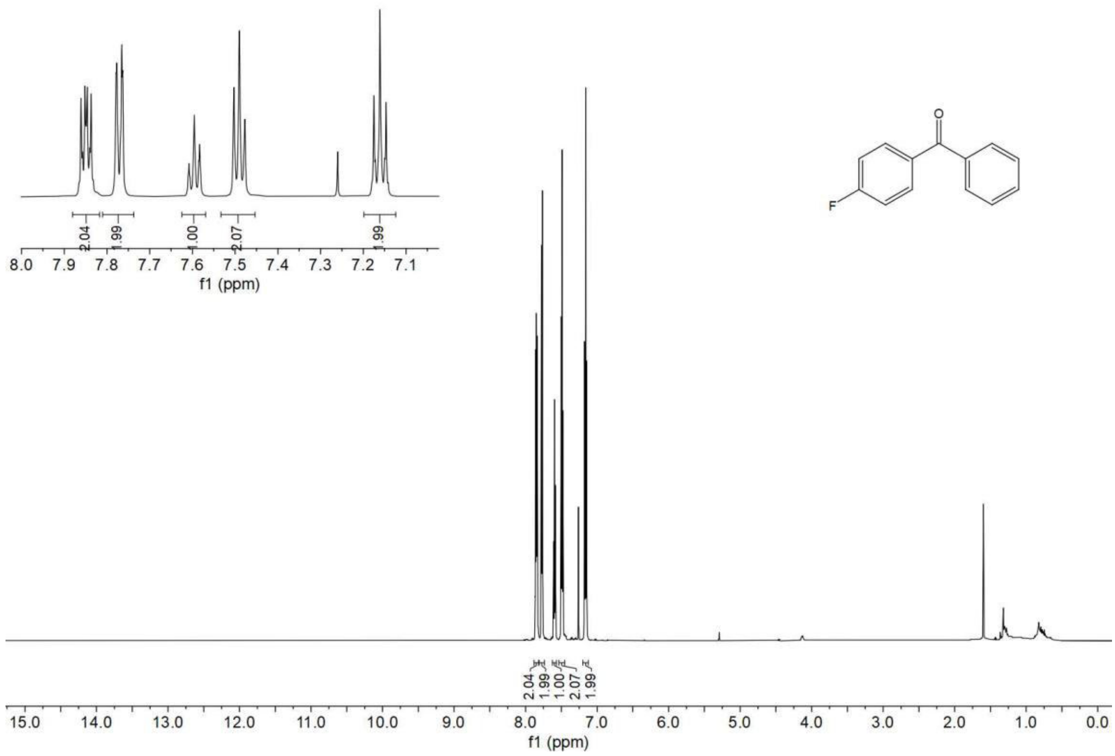

^{13}C NMR (**c4**)

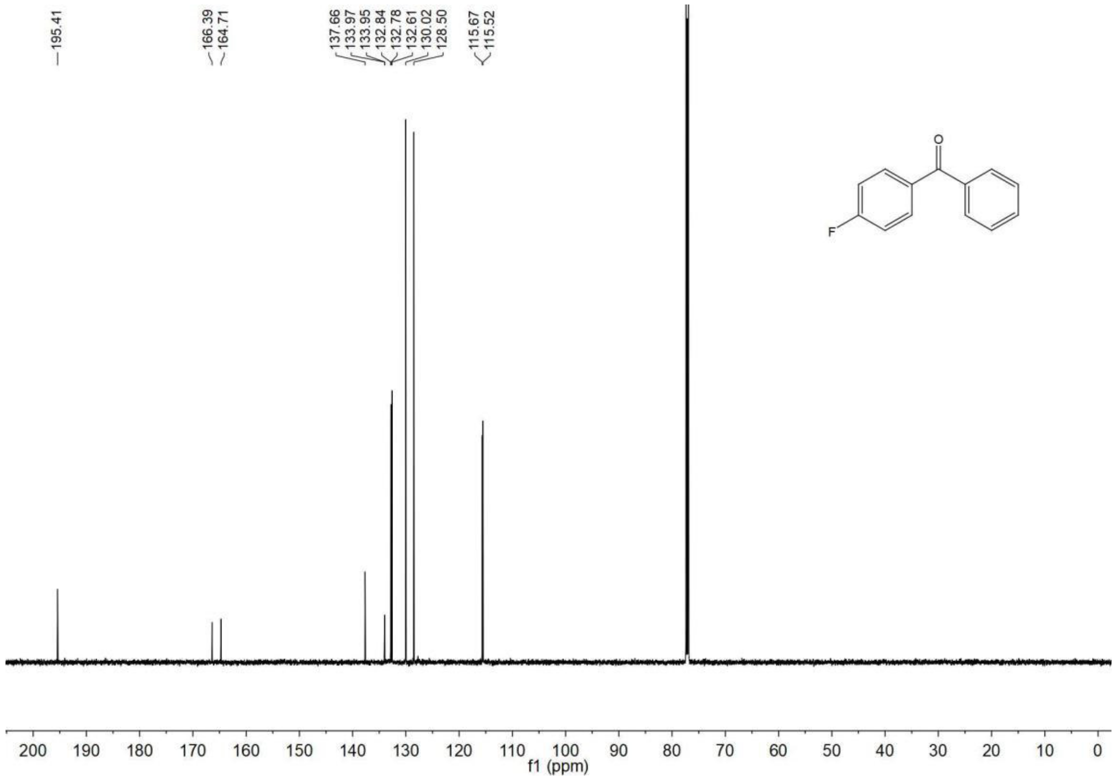

^{19}F NMR (**c4**)

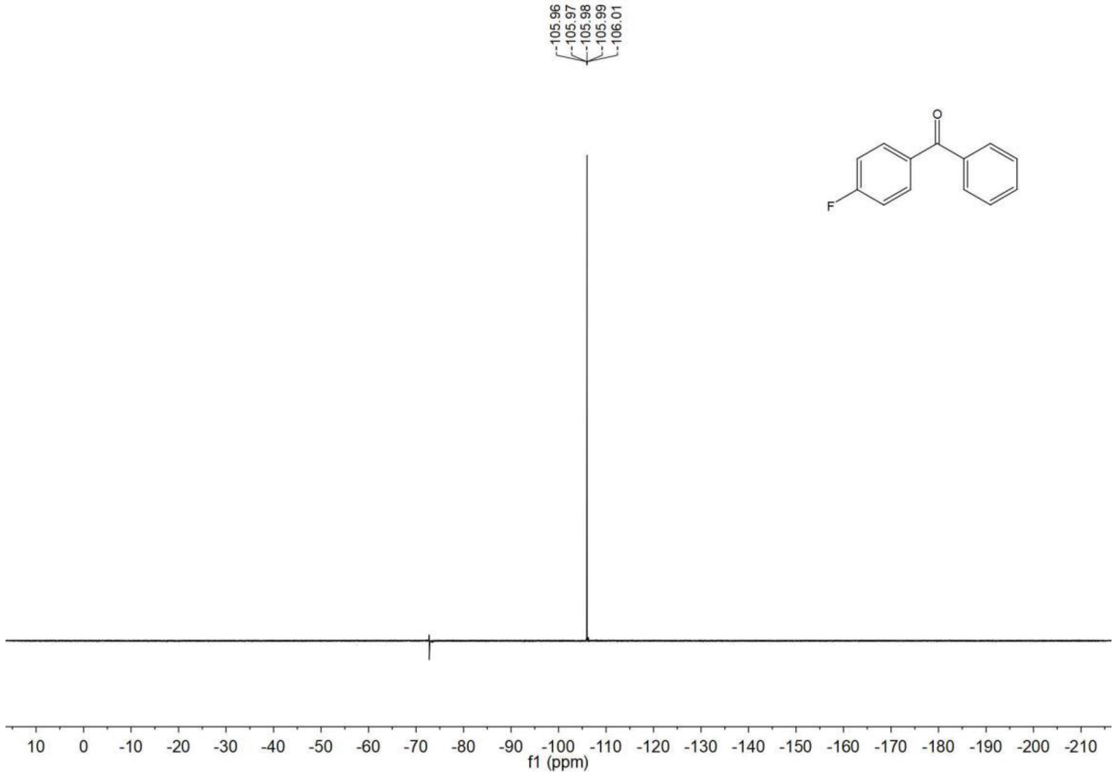

-105.96
-105.97
-105.98
-105.99
-106.01
O
F
10 0 -10 -20 -30 -40 -50 -60 -70 -80 -90 -100 -110 -120 -130 -140 -150 -160 -170 -180 -190 -200 -210
f1 (ppm)

^{1}H NMR (**c5**)

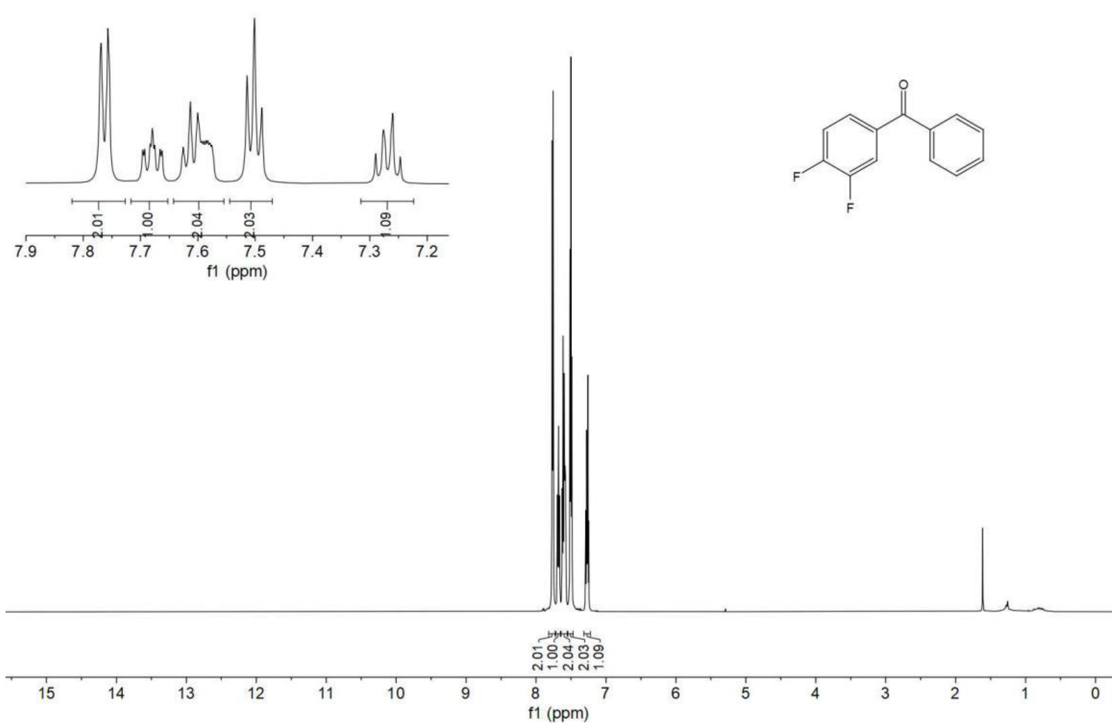

^{13}C NMR (**c5**)

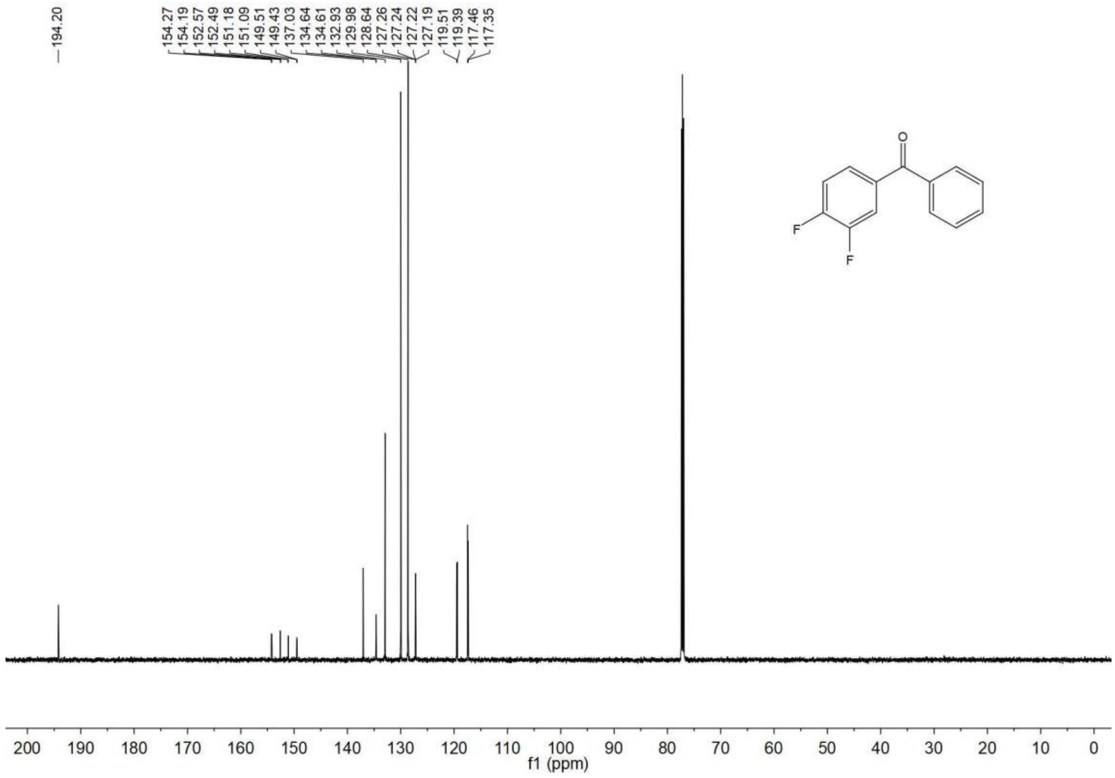

^{19}F NMR (**c5**)

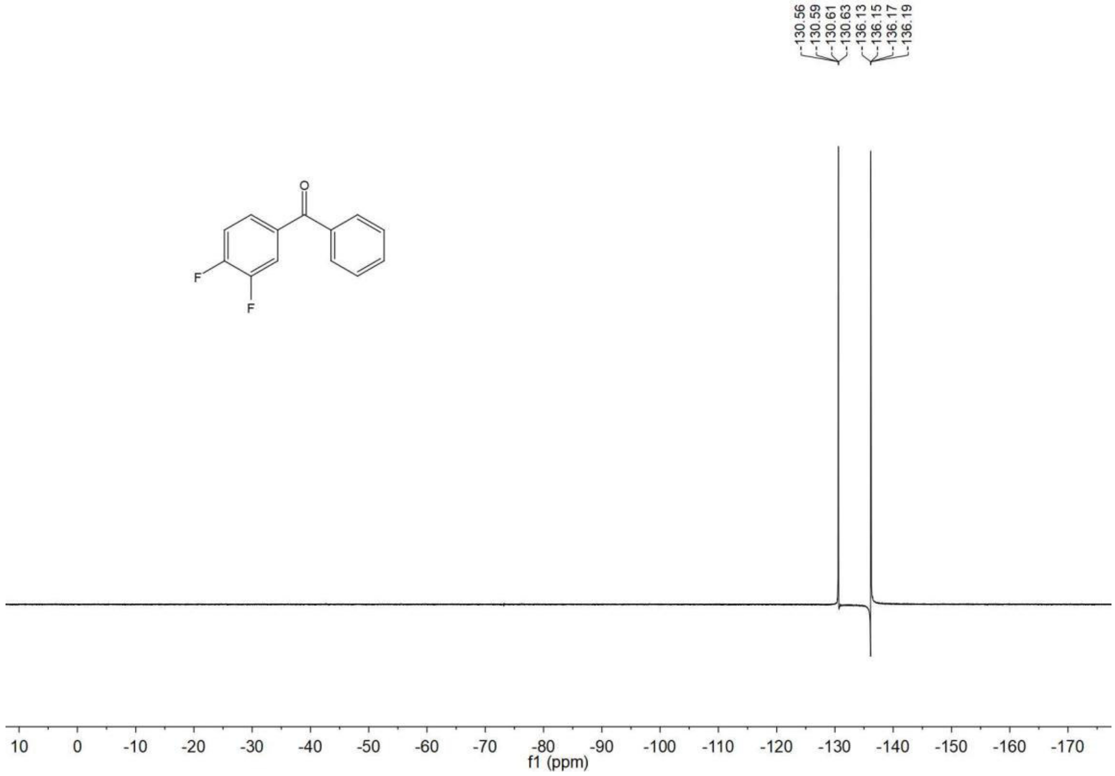

-130.56
-130.59
-130.61
-130.63
-136.13
-136.15
-136.17
-136.19
O
F
F
10 0 -10 -20 -30 -40 -50 -60 -70 -80 -90 -100 -110 -120 -130 -140 -150 -160 -170
f1 (ppm)

^{1}H NMR (**c6**)

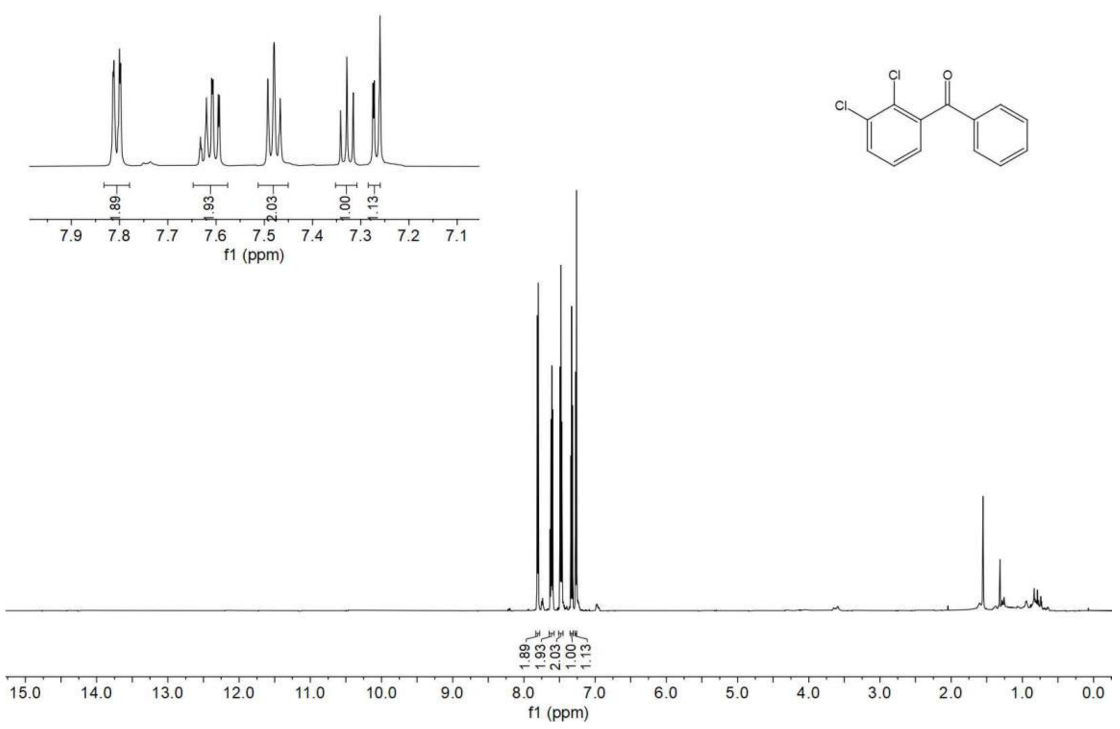

^{13}C NMR (**c6**)

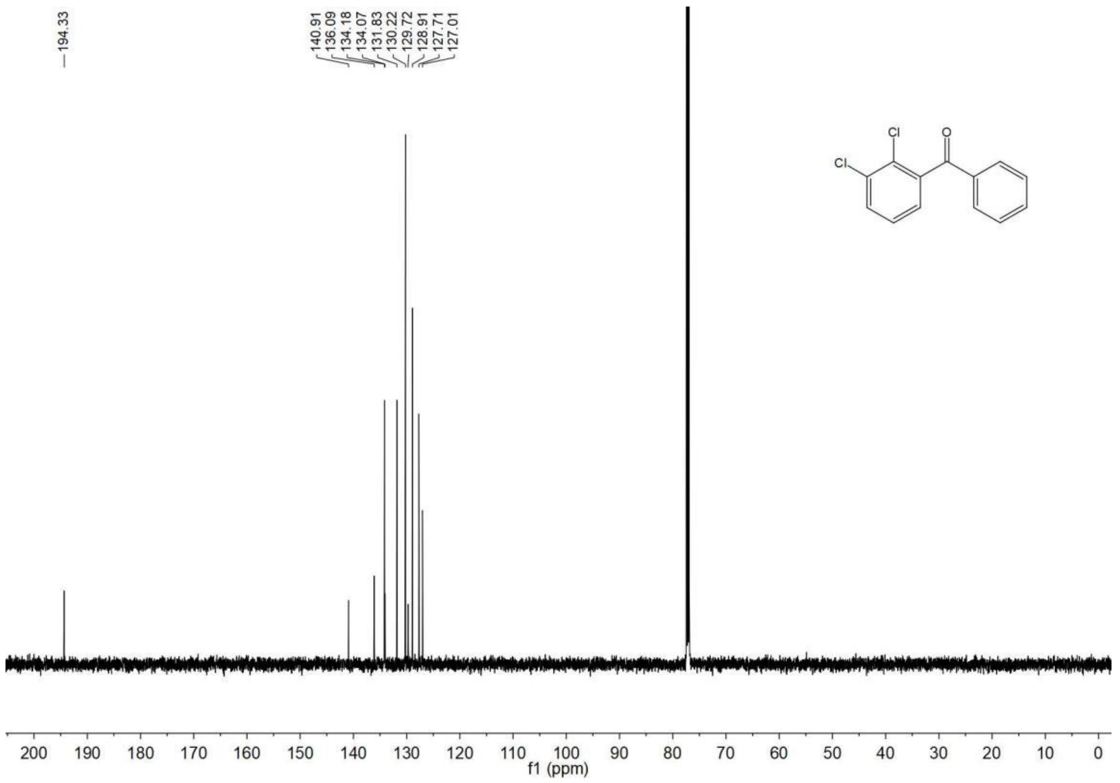

^{1}H NMR (**c7**)

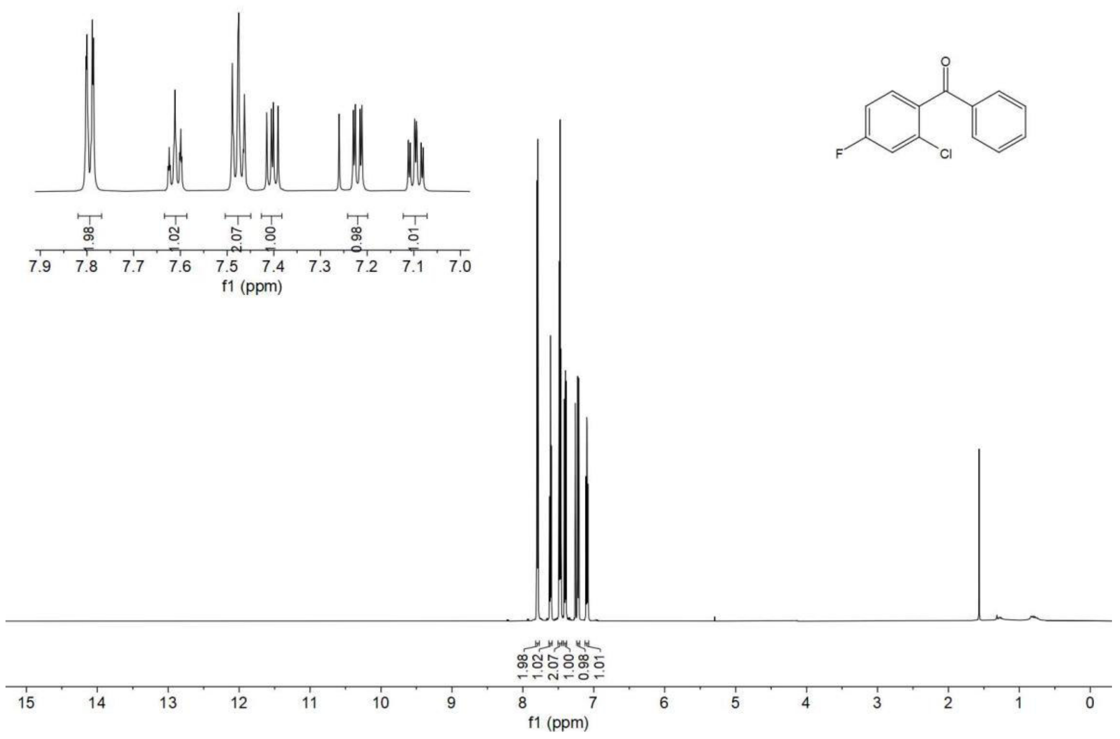

^{13}C NMR (**c7**)

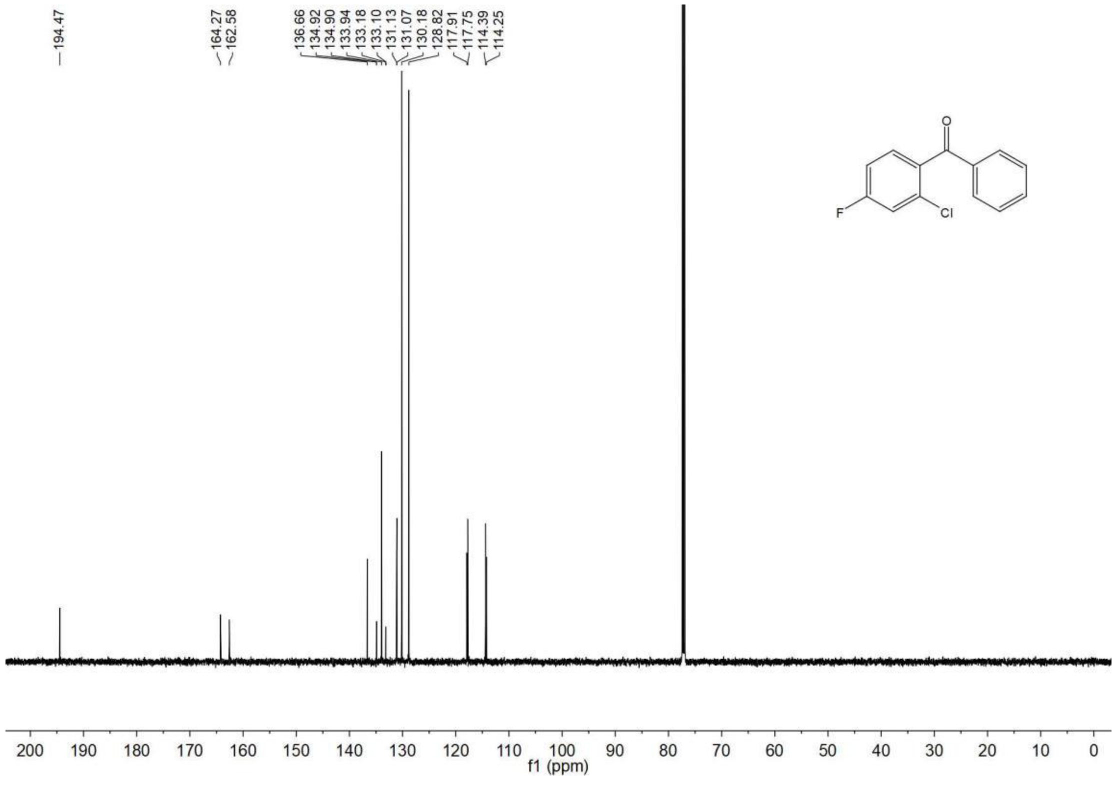

^{19}F NMR (**c7**)

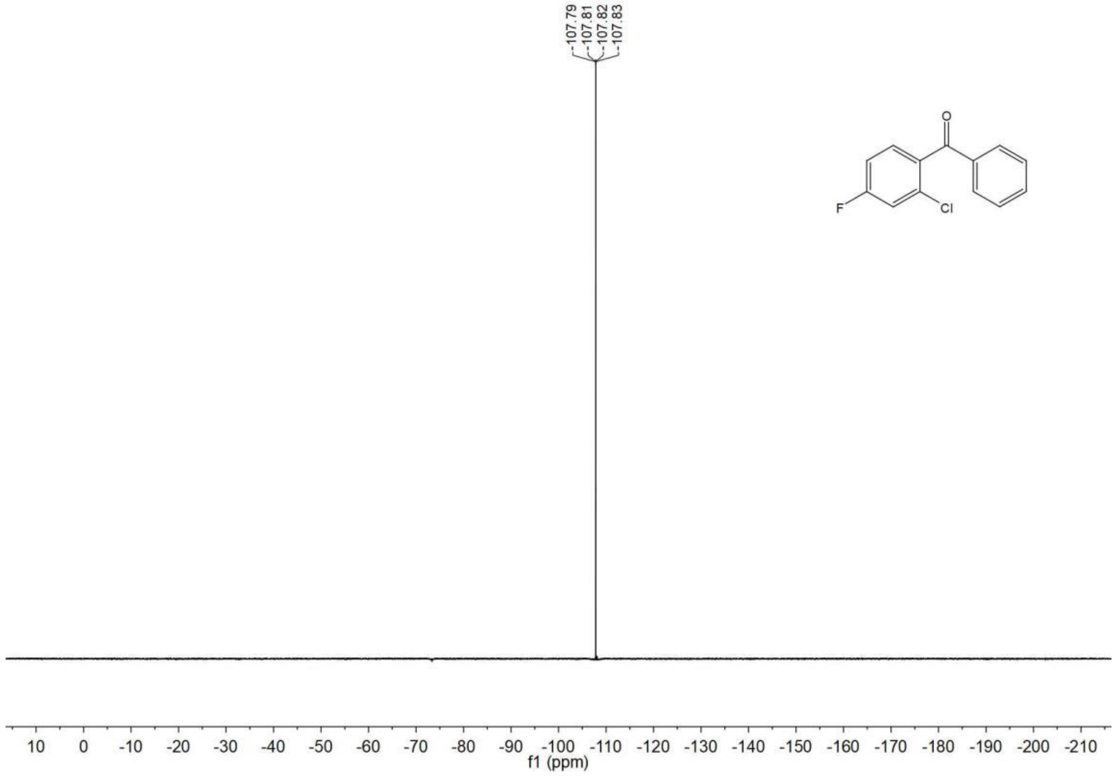
-107.79
-107.81
-107.82
-107.83
F
Cl
O
10 0 -10 -20 -30 -40 -50 -60 -70 -80 -90 -100 -110 -120 -130 -140 -150 -160 -170 -180 -190 -200 -210
f1 (ppm)

^{1}H NMR (**c8**)

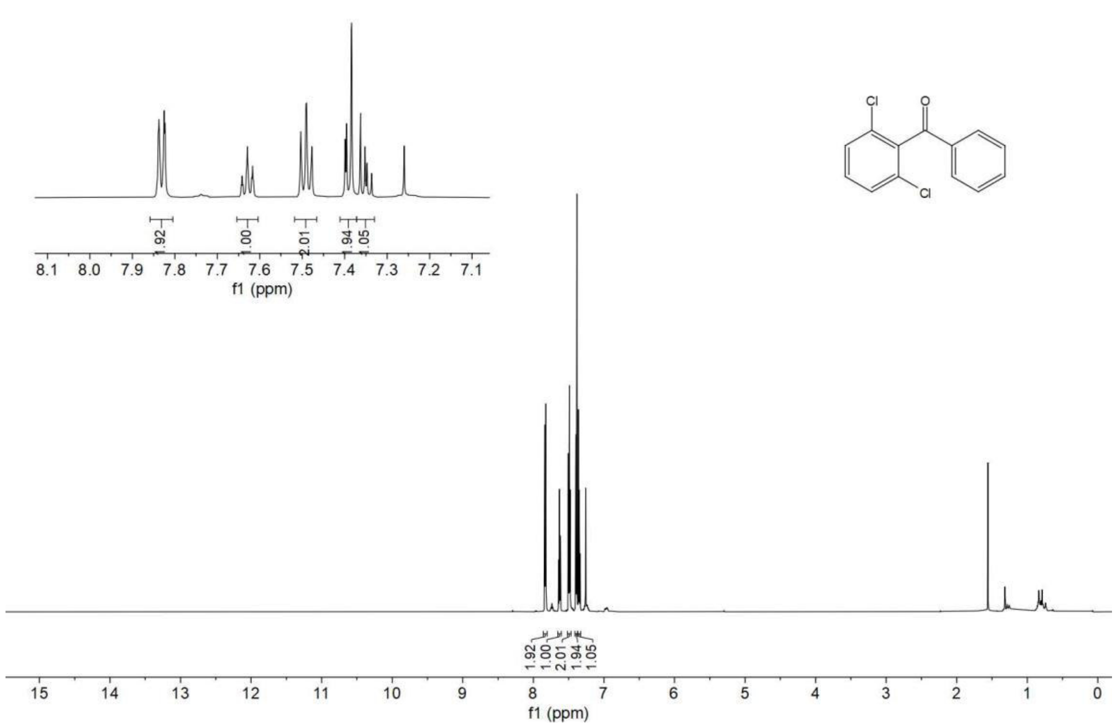

^{13}C NMR (**c8**)

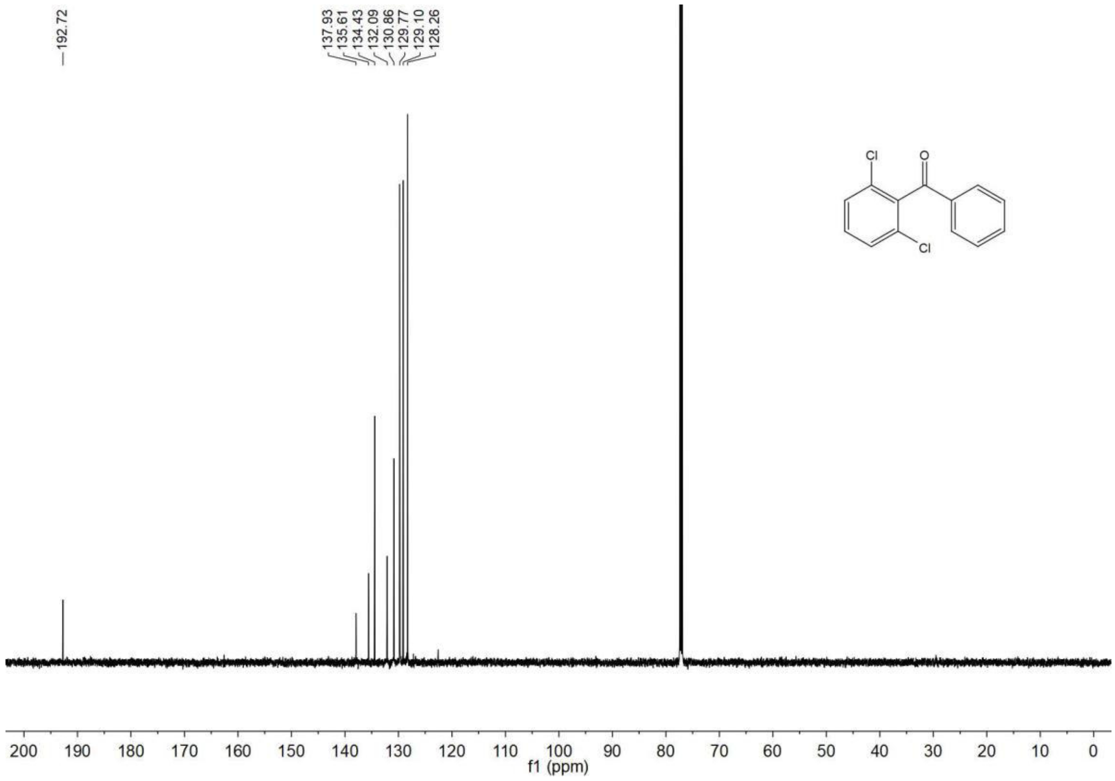

^{1}H NMR (**c9**)

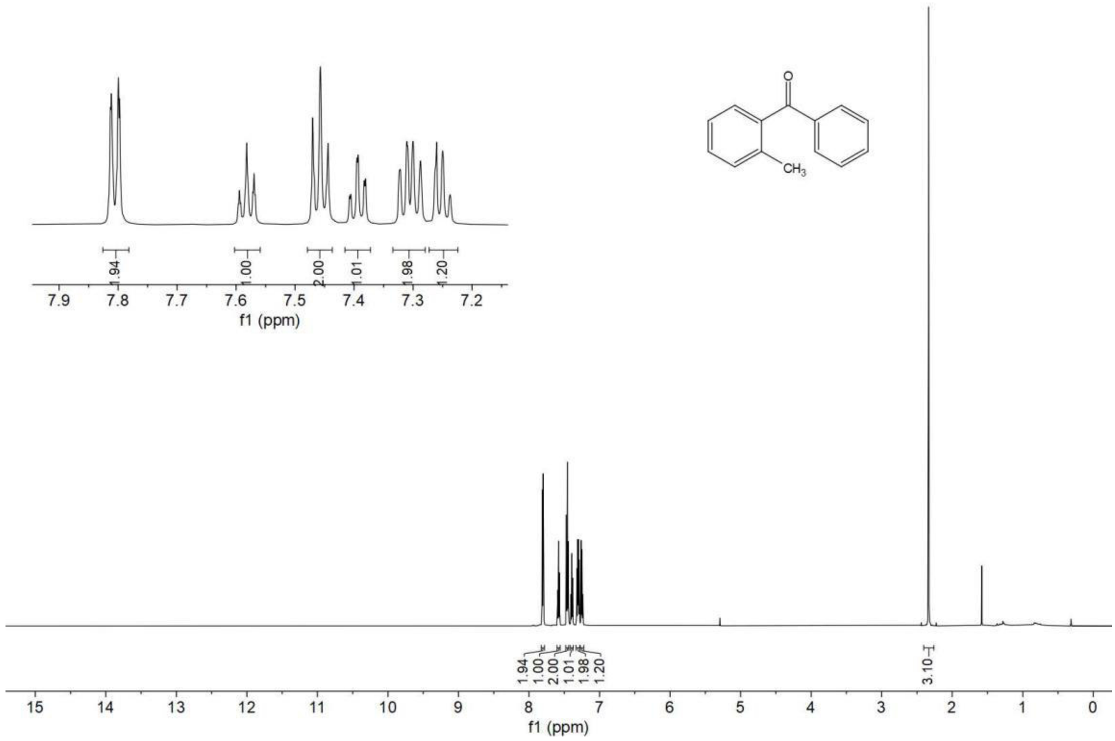

^{13}C NMR (**c9**)

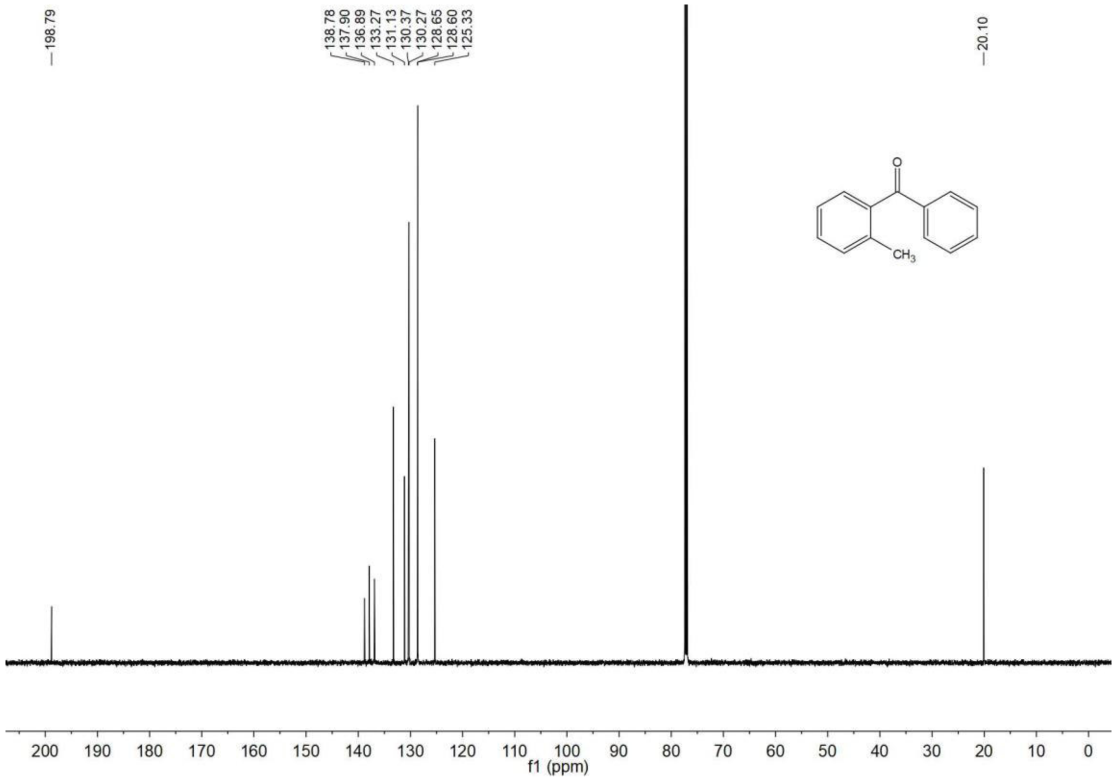

^{1}H NMR (**c10**)

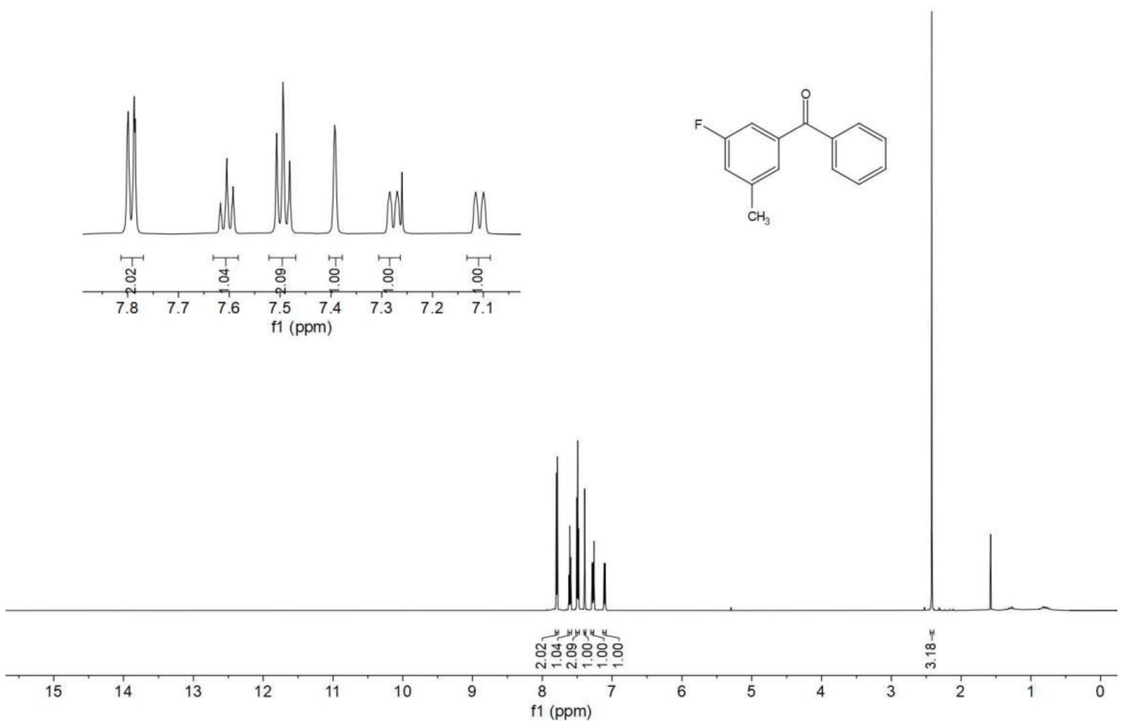

^{13}C NMR (**c10**)

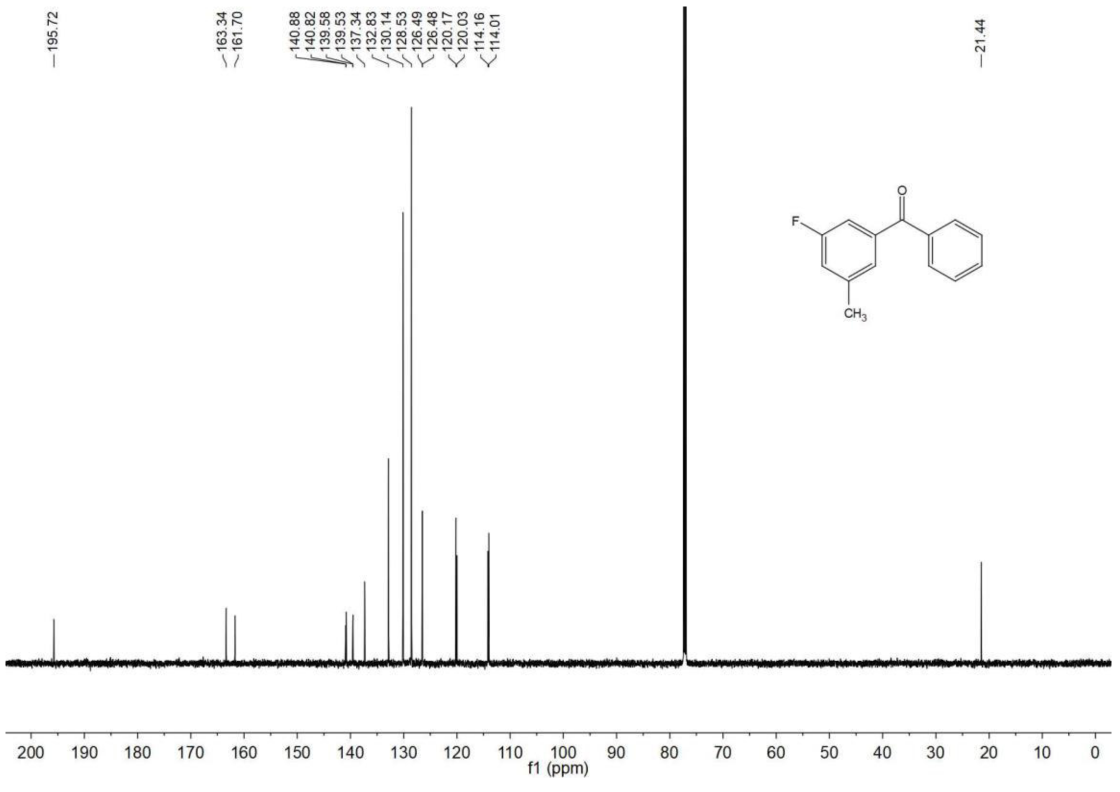

^{19}F NMR (**c10**)

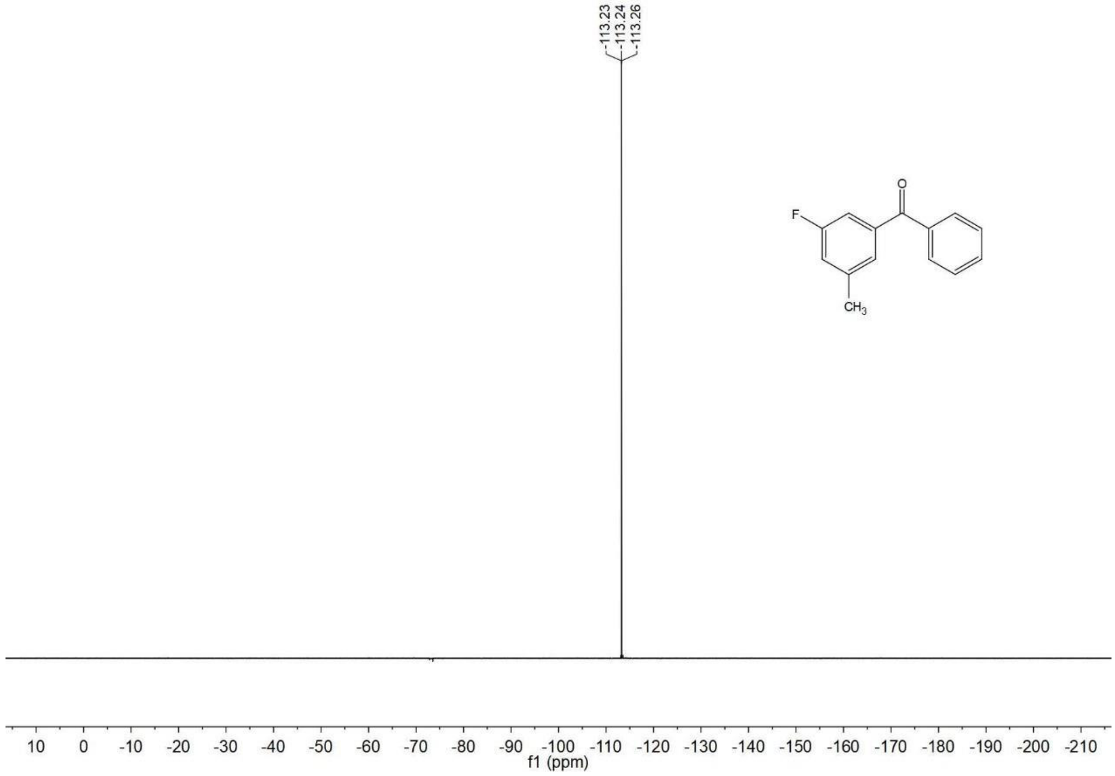

-113.23
-113.24
-113.26
F
O
CH3
10 0 -10 -20 -30 -40 -50 -60 -70 -80 -90 -100 -110 -120 -130 -140 -150 -160 -170 -180 -190 -200 -210
f1 (ppm)

^{1}H NMR (**c11**)

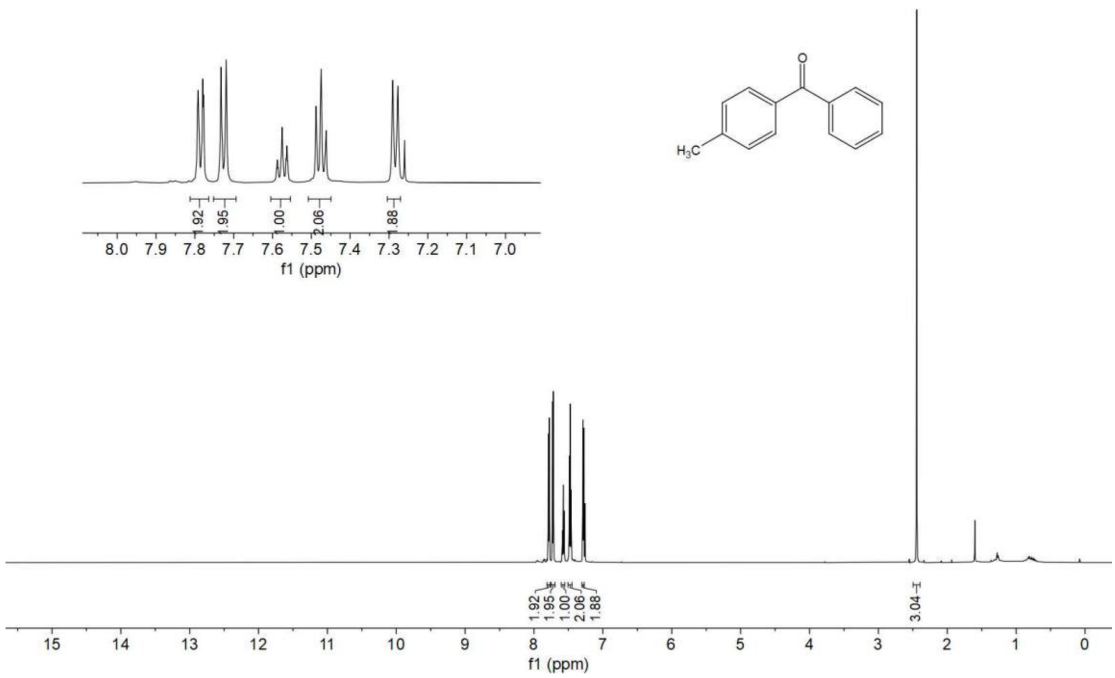

^{13}C NMR (**c11**)

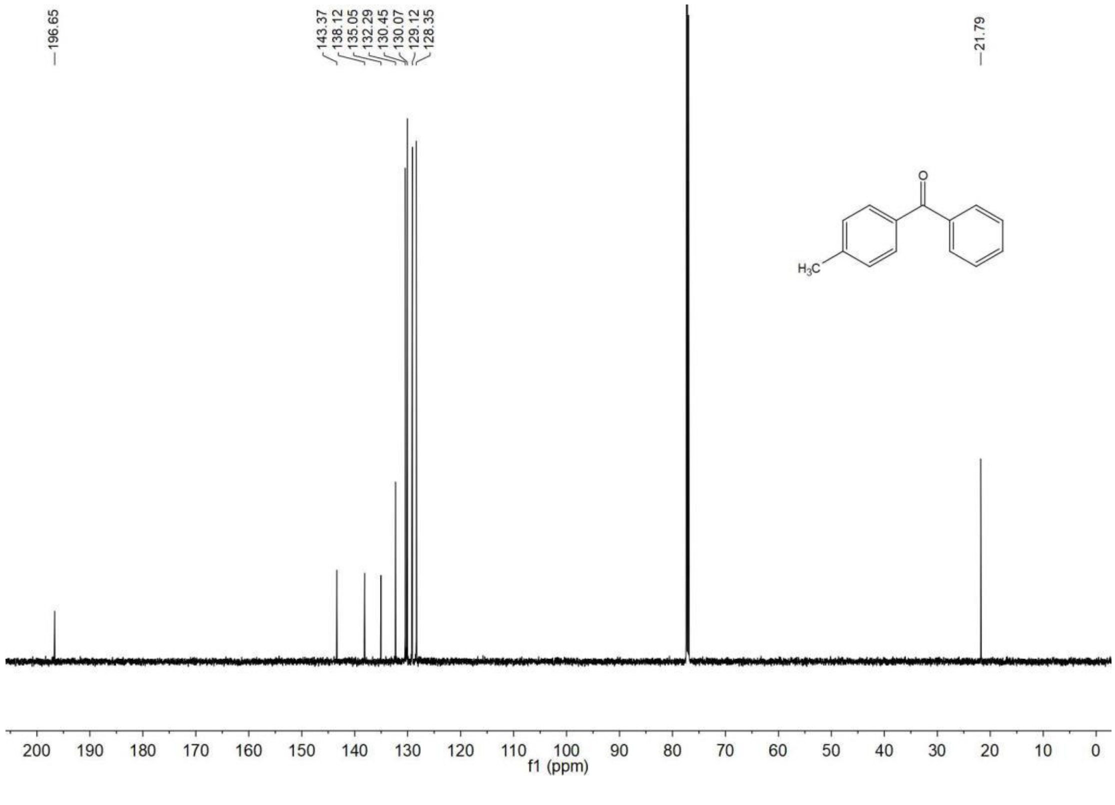

^{1}H NMR (**c12**)

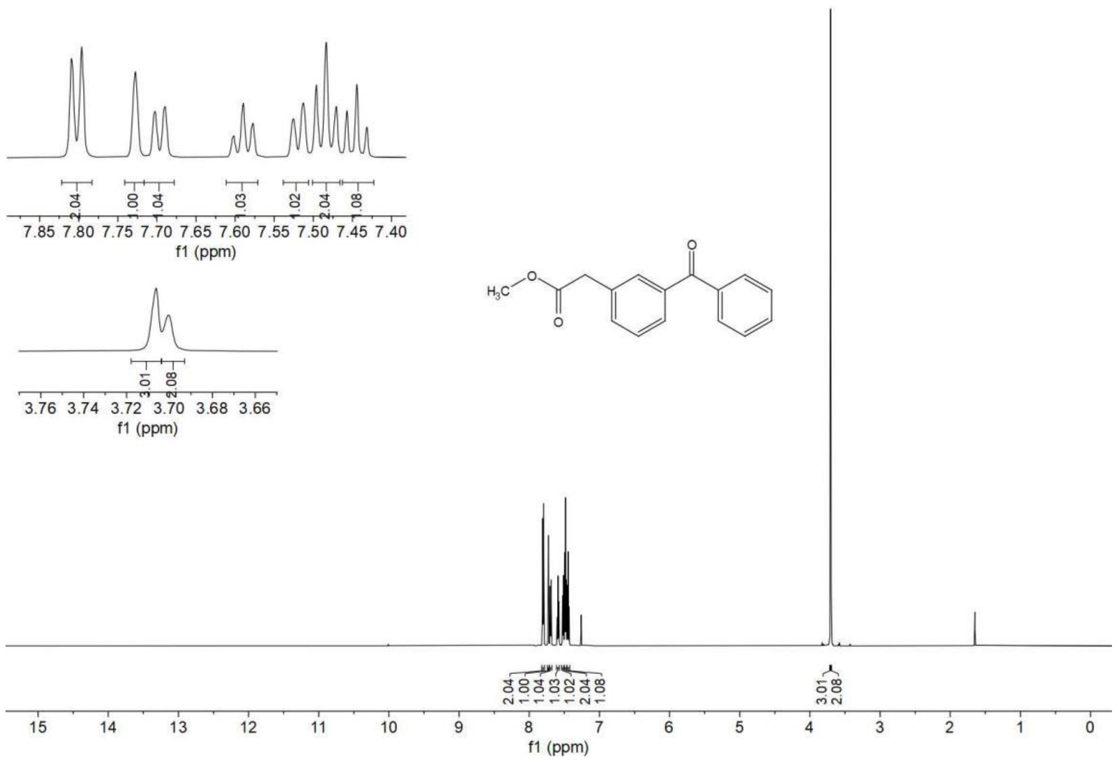

^{13}C NMR (**c12**)

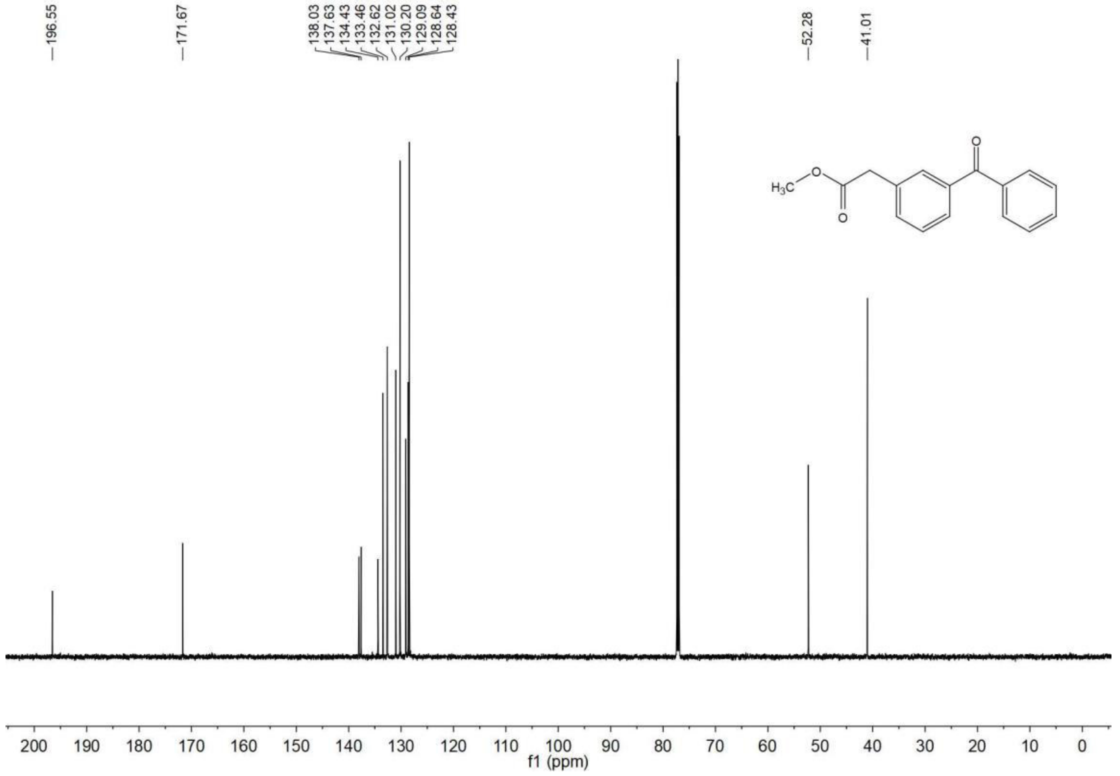

^{1}H NMR (**c13**)

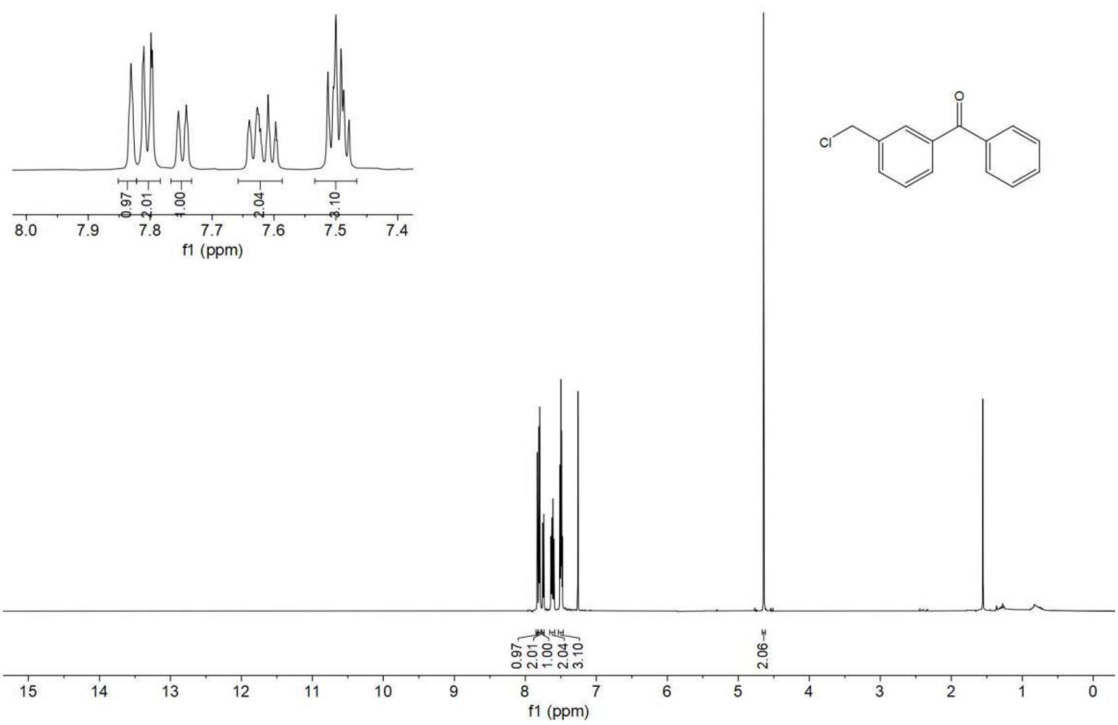

^{13}C NMR (**c13**)

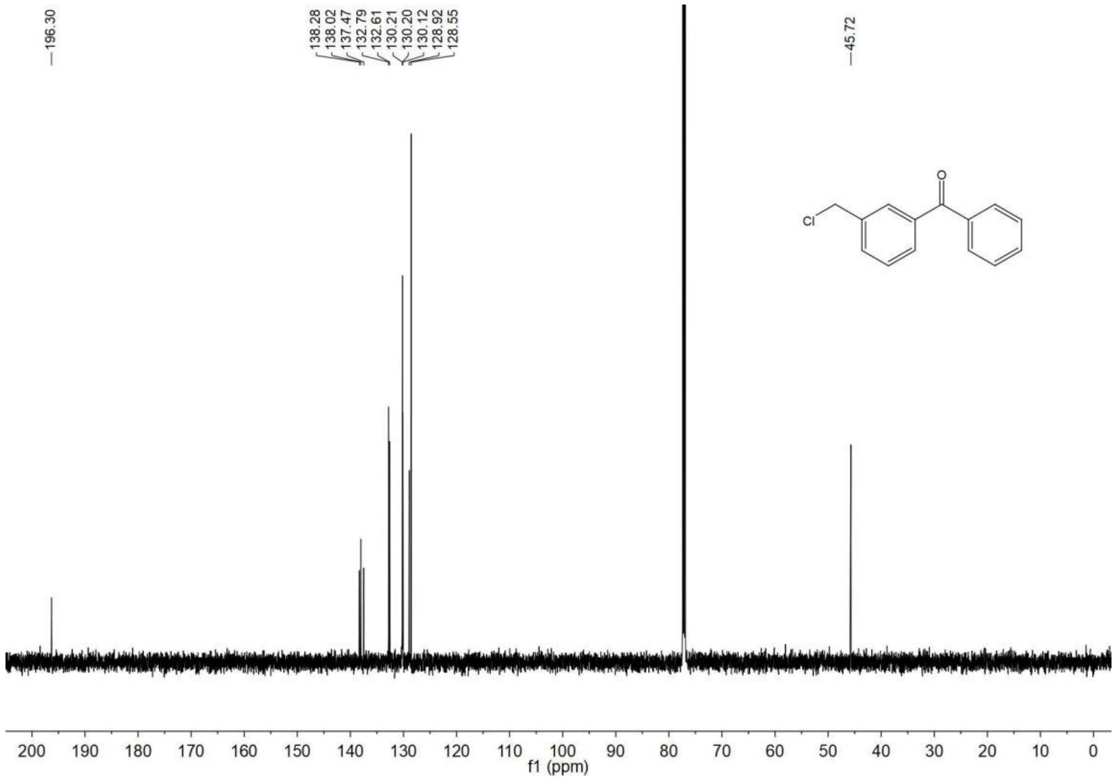

^{1}H NMR (**c14**)

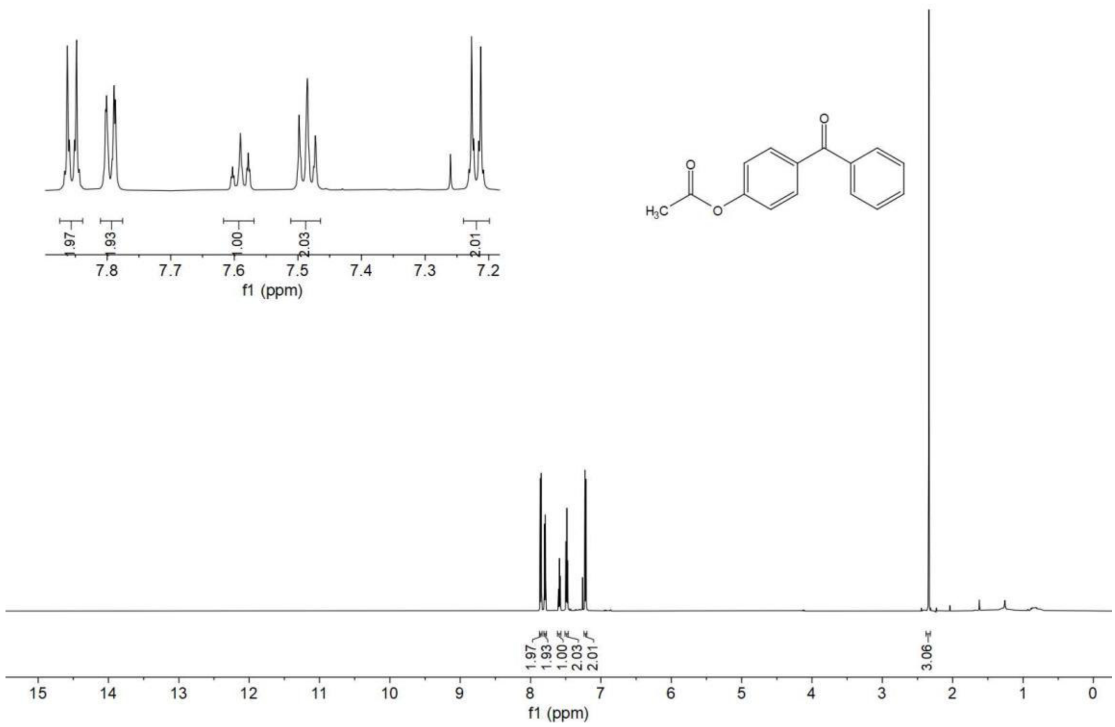

^{13}C NMR (**c14**)

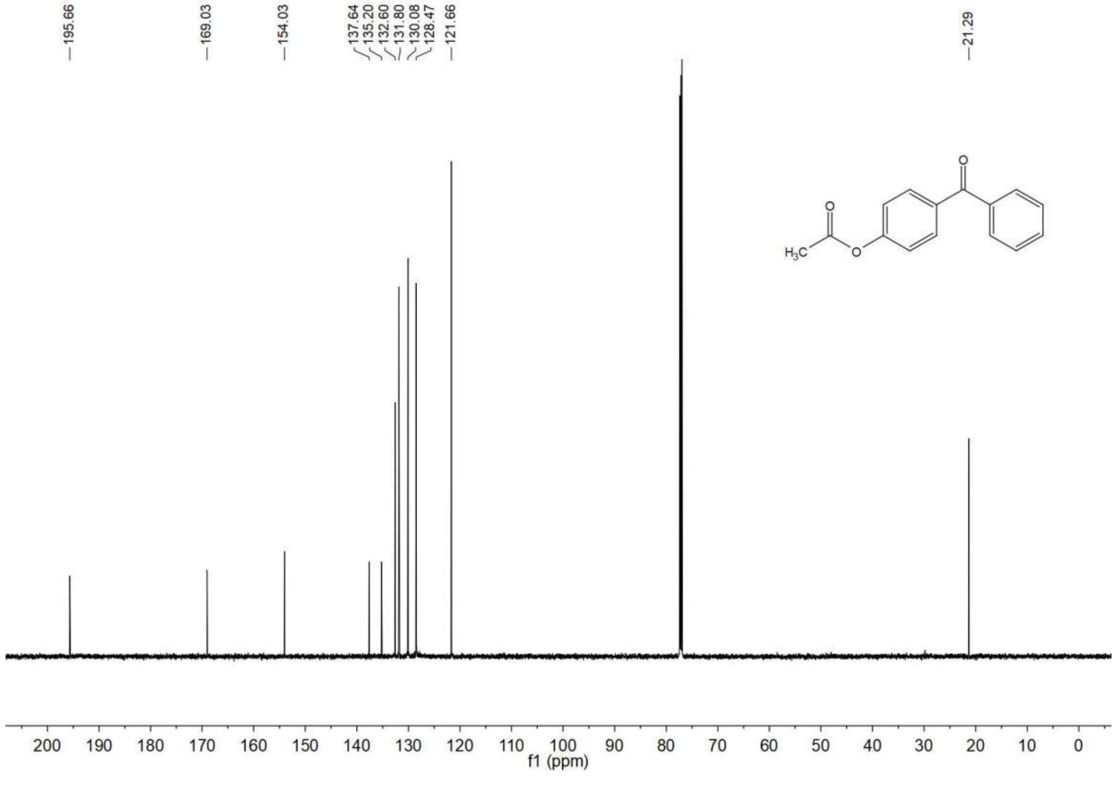

^{1}H NMR (**c15**)

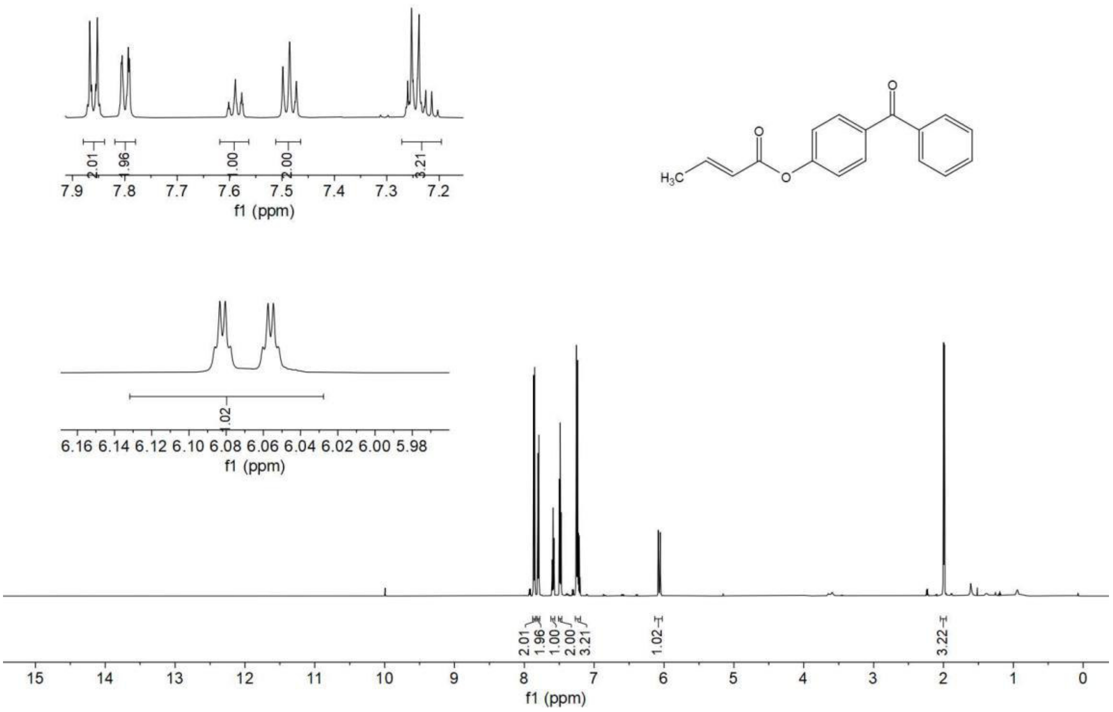

^{13}C NMR (**c15**)

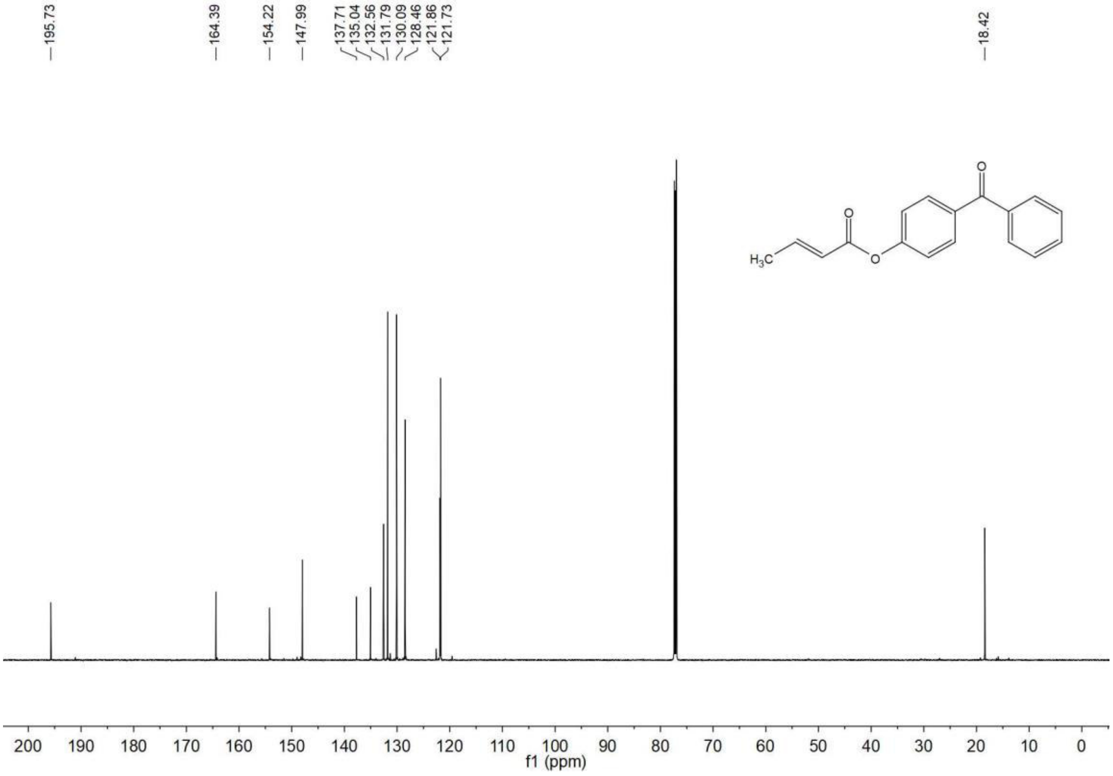

^{1}H NMR (**c16**)

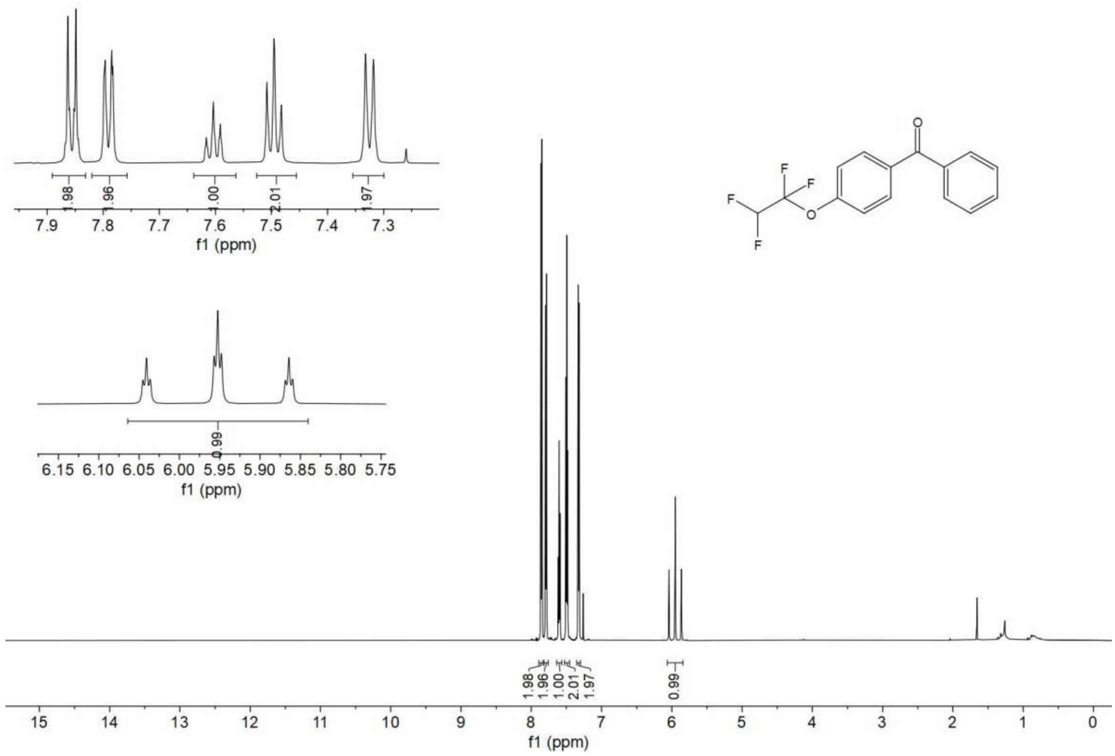

^{13}C NMR (**c16**)

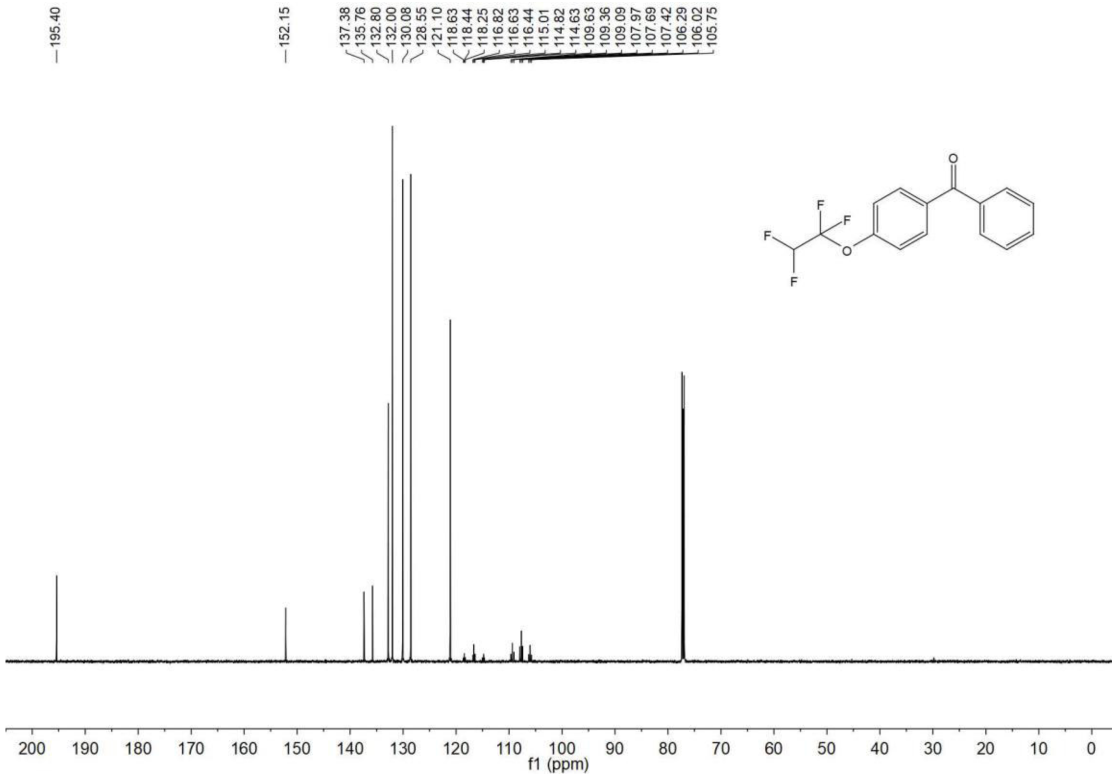

^{19}F NMR (**c16**)

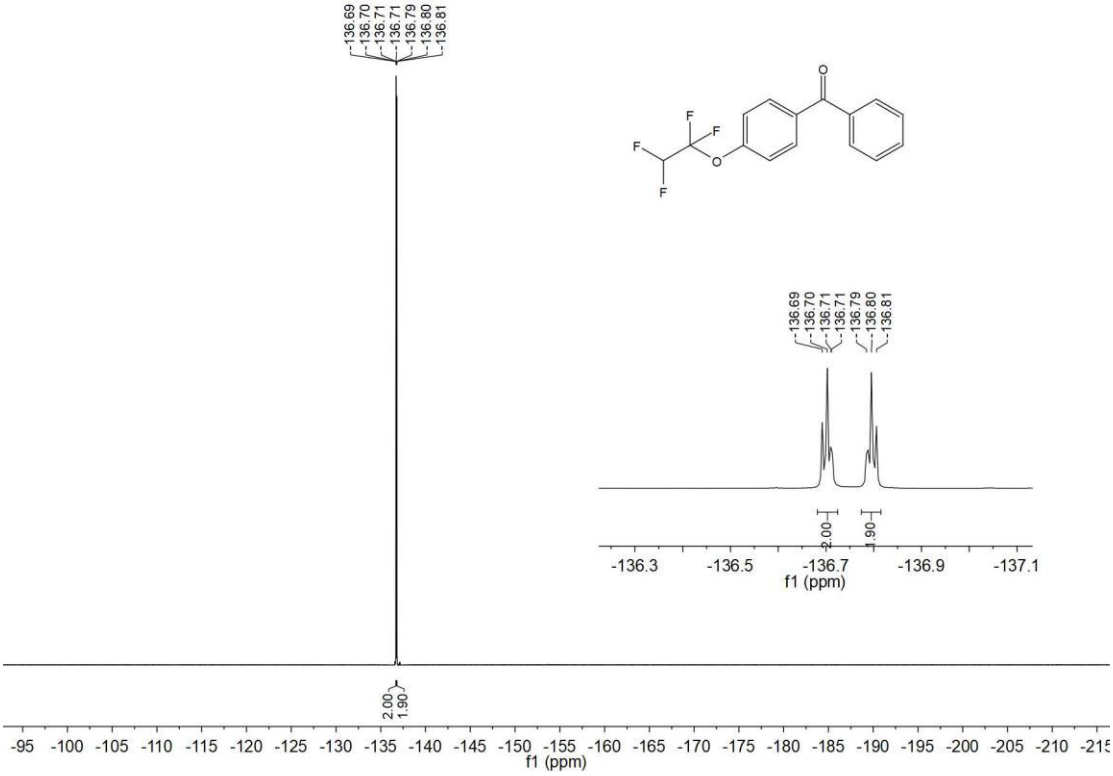

-136.69
-136.70
-136.71
-136.71
-136.79
-136.80
-136.81
F
F
F
F
O
O
2.00
1.90
-136.3
-136.5
-136.7
-136.9
-137.1
f1 (ppm)
-95 -100 -105 -110 -115 -120 -125 -130 -135 -140 -145 -150 -155 -160 -165 -170 -175 -180 -185 -190 -195 -200 -205 -210 -215
f1 (ppm)

^{1}H NMR (**c17**)

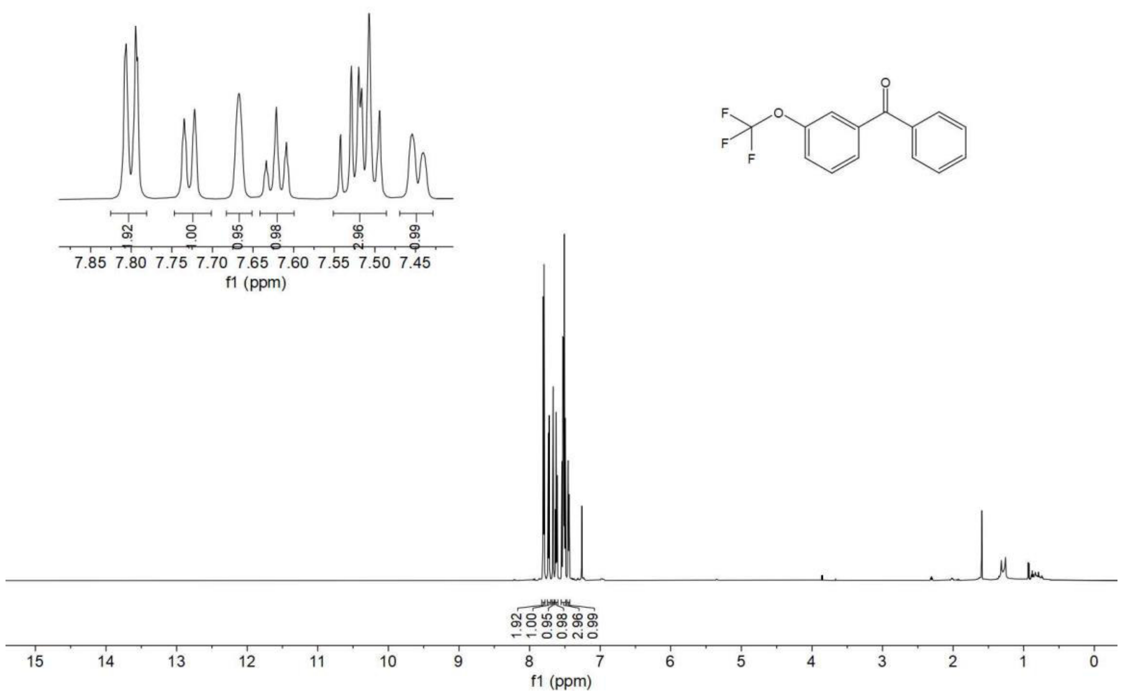

^{13}C NMR (**c17**)

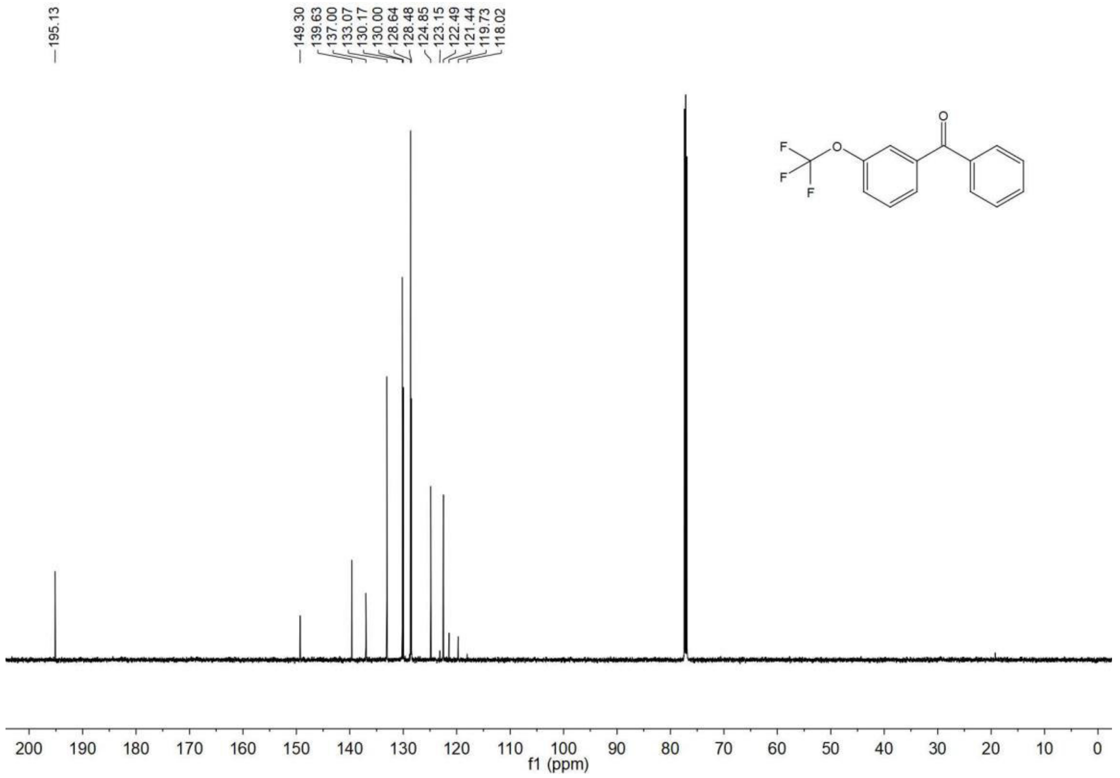

^{19}F NMR (**c17**)

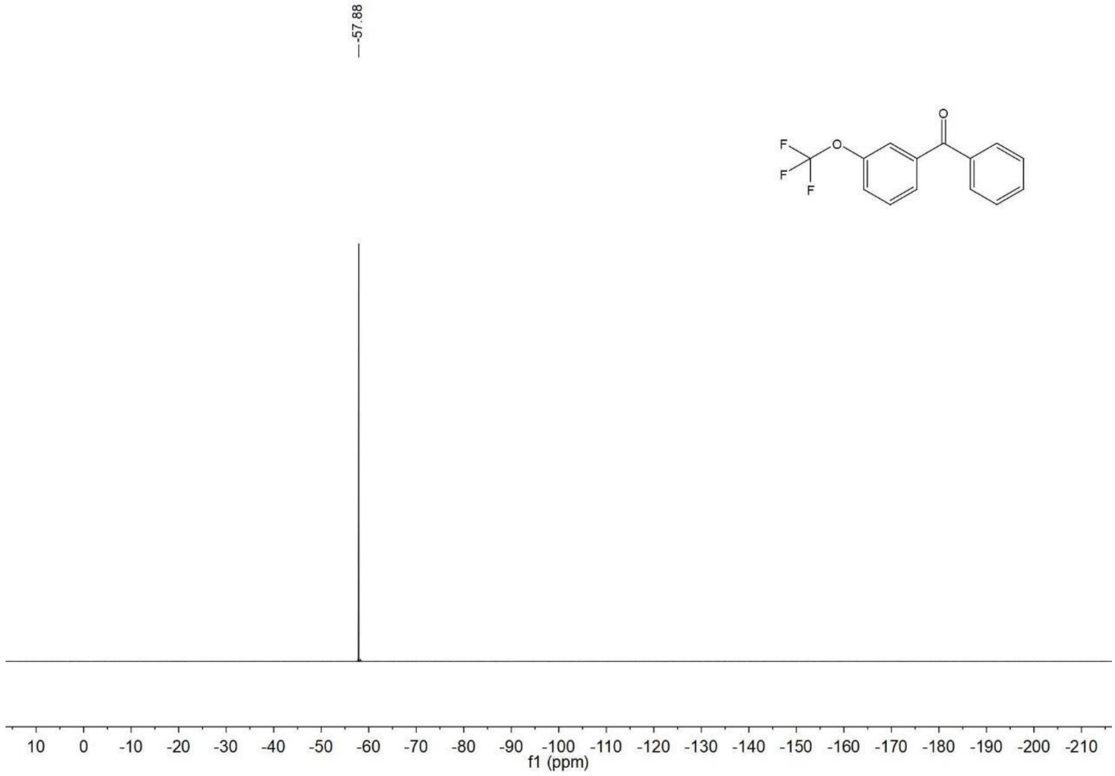

-57.88
F
F
F
O
O
10 0 -10 -20 -30 -40 -50 -60 -70 -80 -90 -100 -110 -120 -130 -140 -150 -160 -170 -180 -190 -200 -210
f1 (ppm)

^{1}H NMR (**c18**)

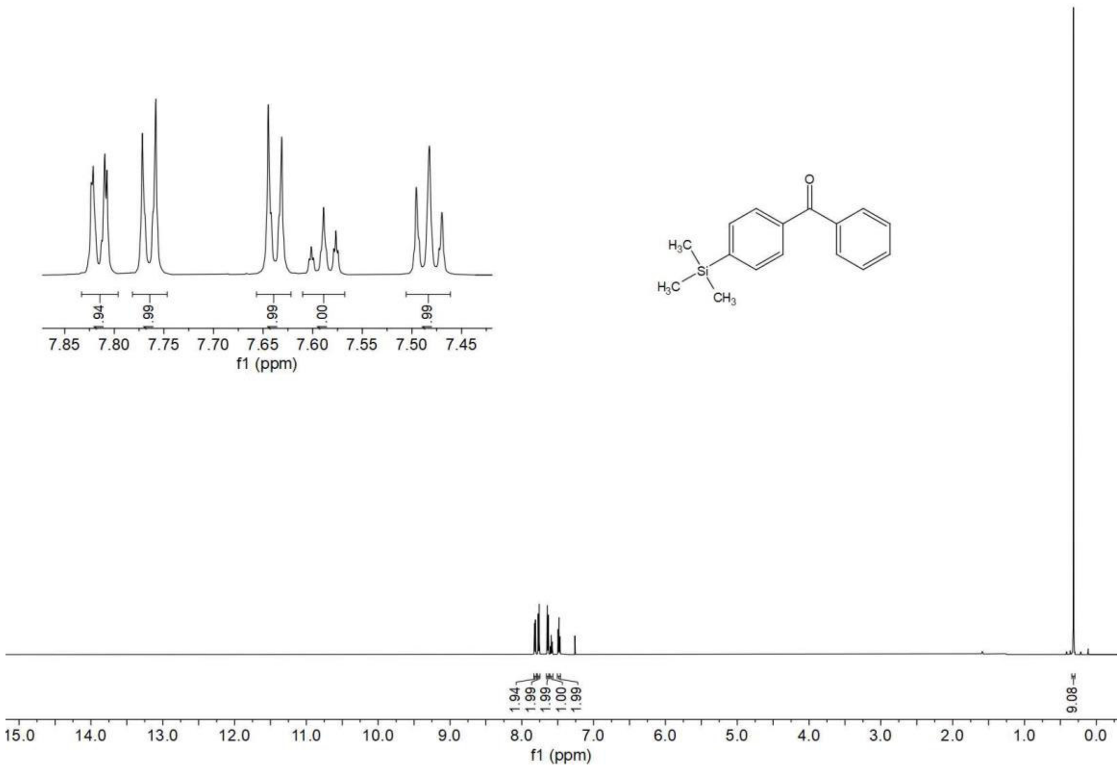

^{13}C NMR (**c18**)

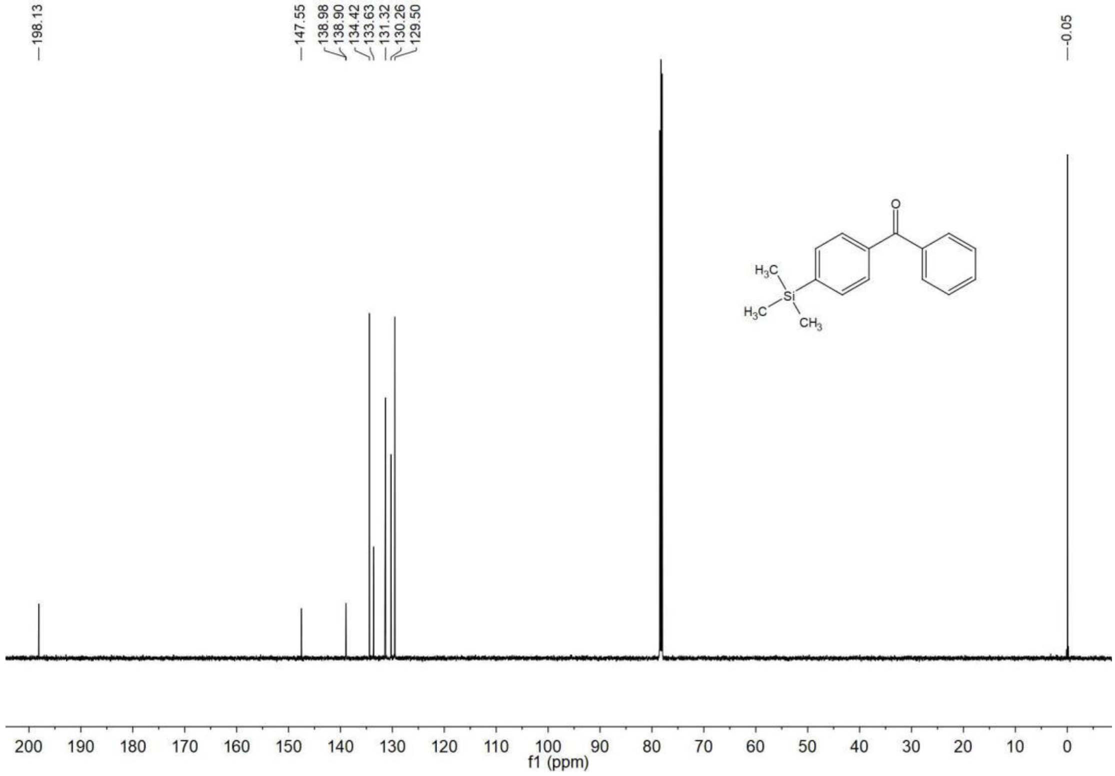

^{1}H NMR (**c19**)

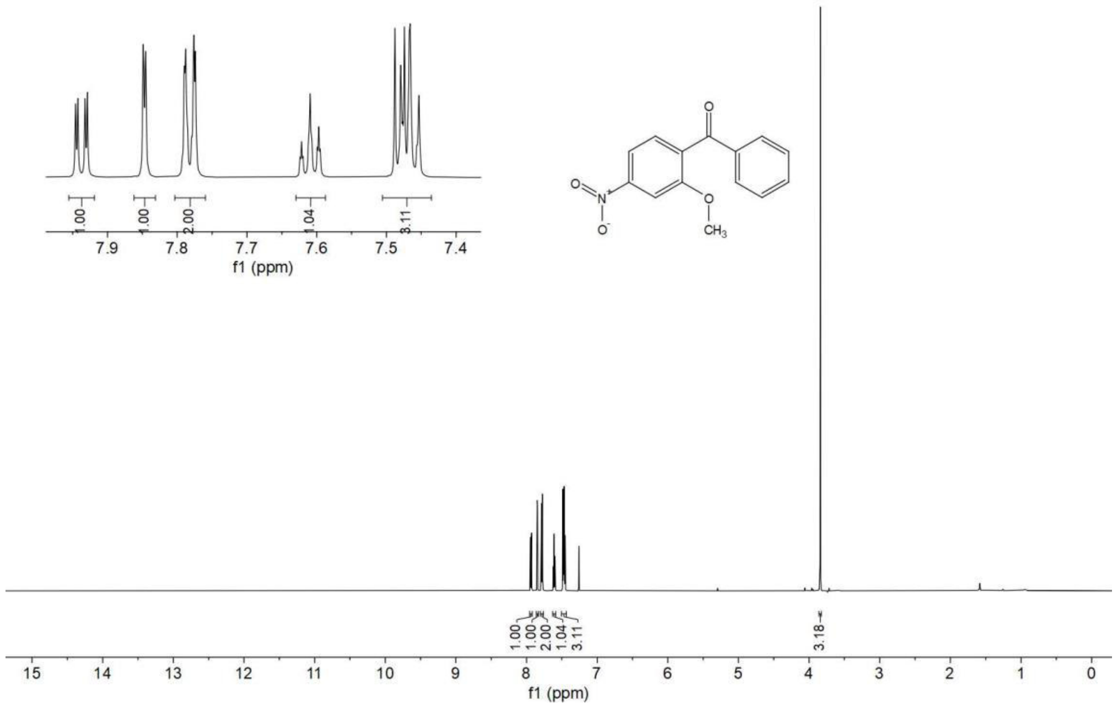

^{13}C NMR (**c19**)

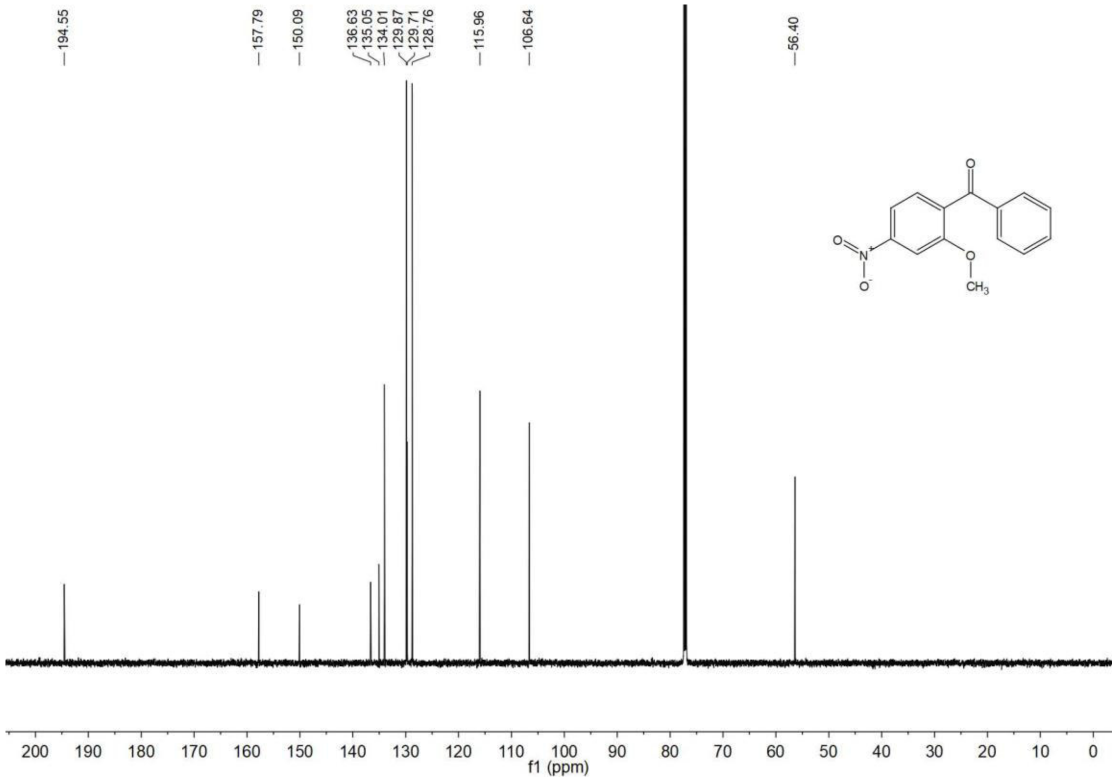

^{1}H NMR (**c20**)

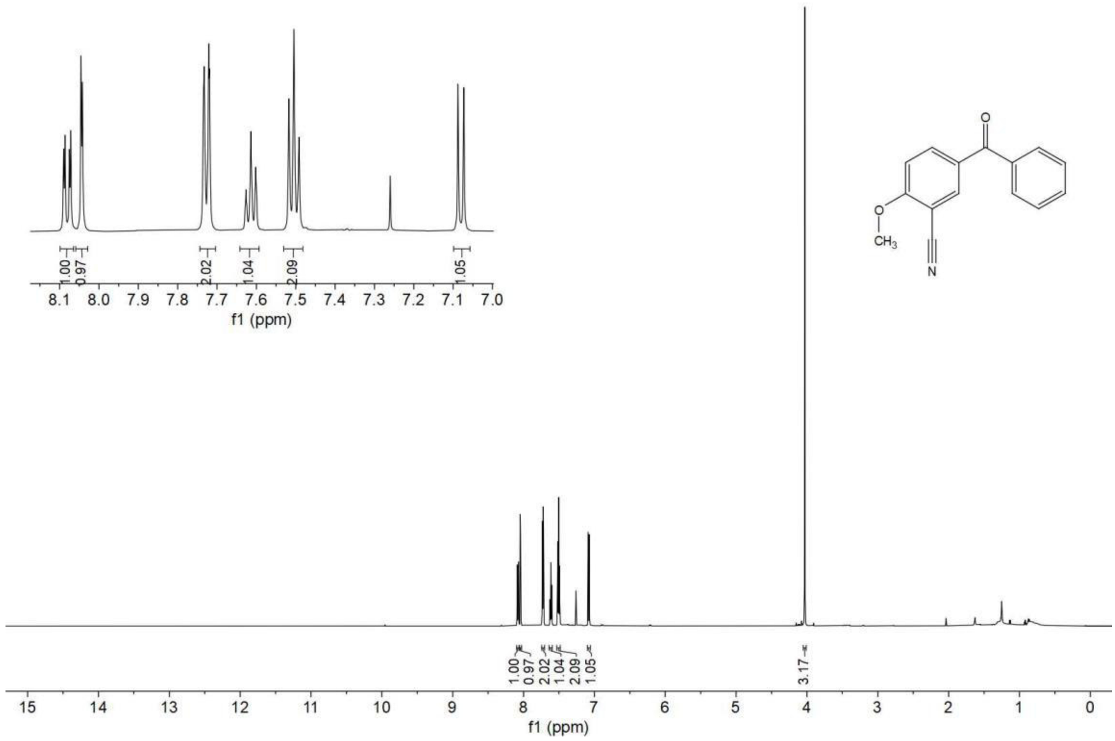

^{13}C NMR (**c20**)

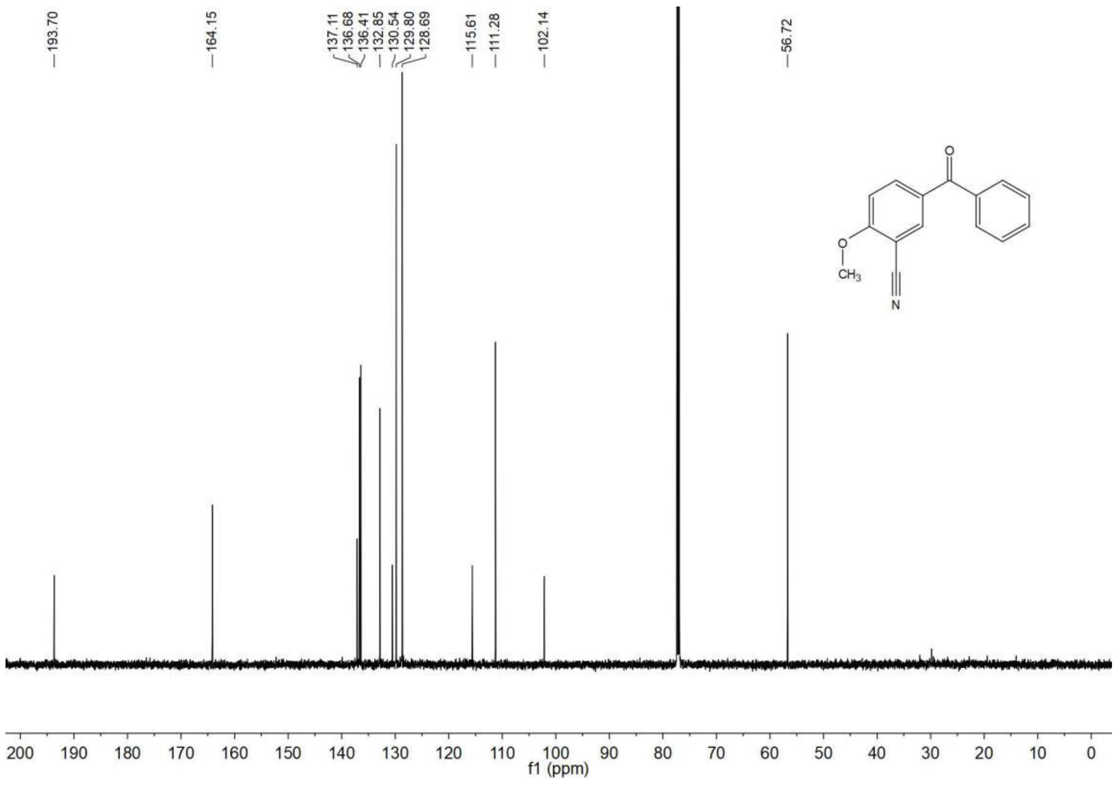

^{1}H NMR (**c21**)

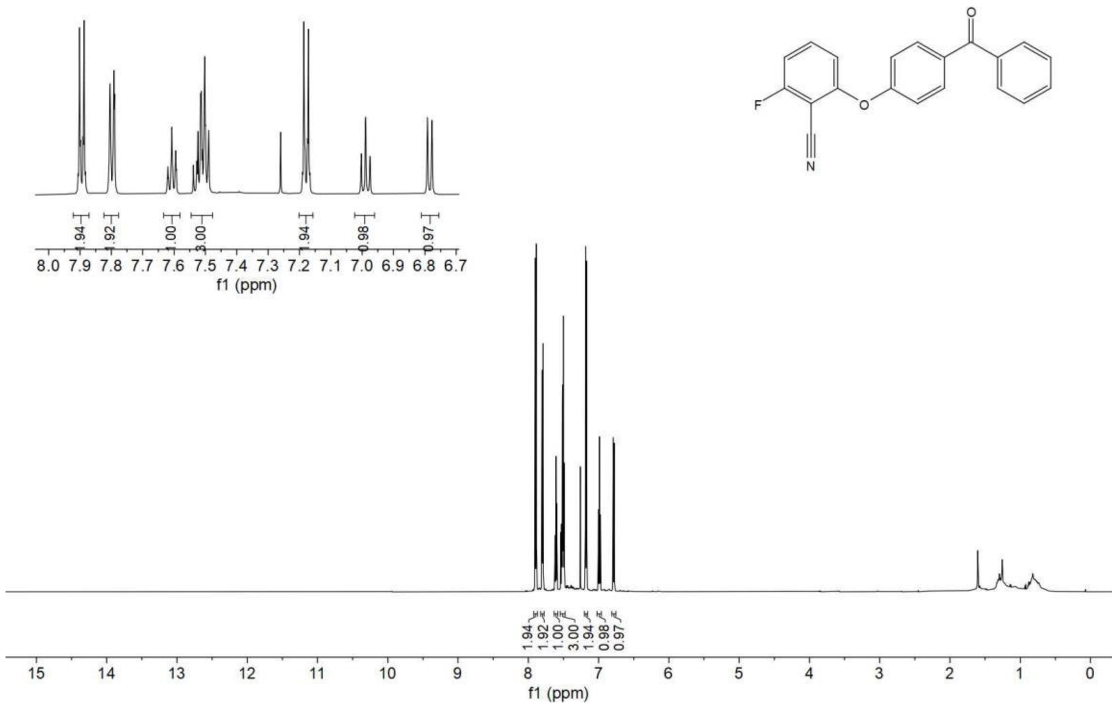

^{13}C NMR (**c21**)

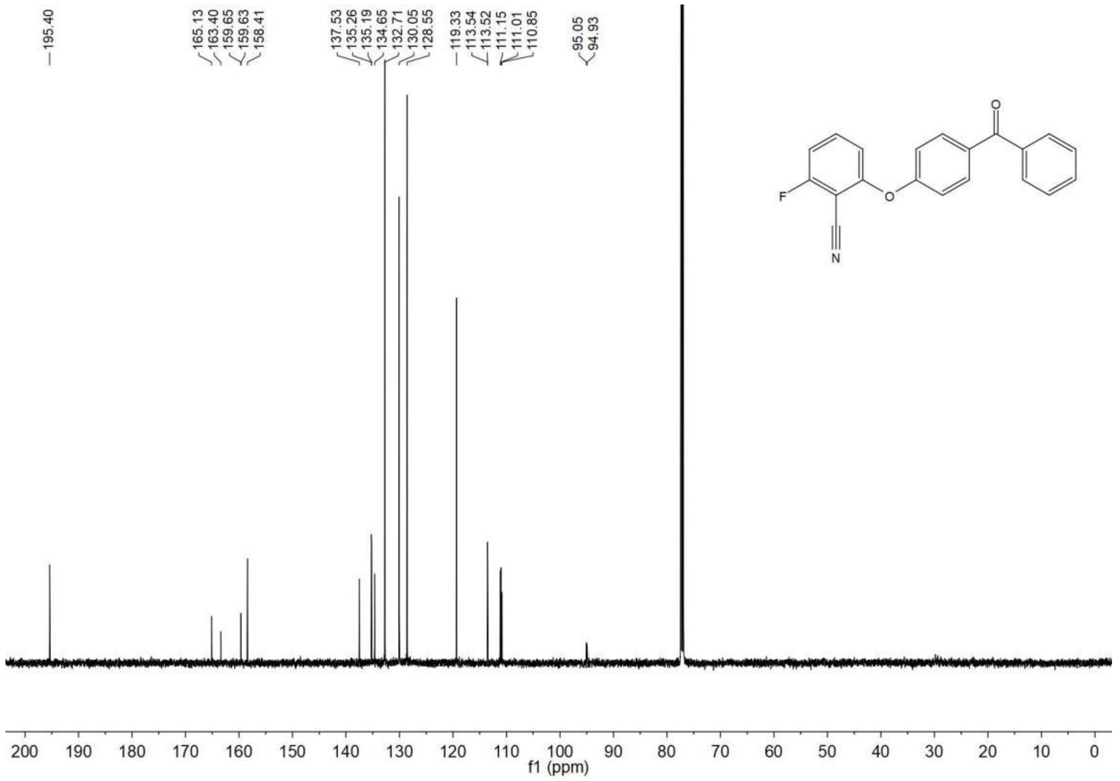

^{19}F NMR (**c21**)

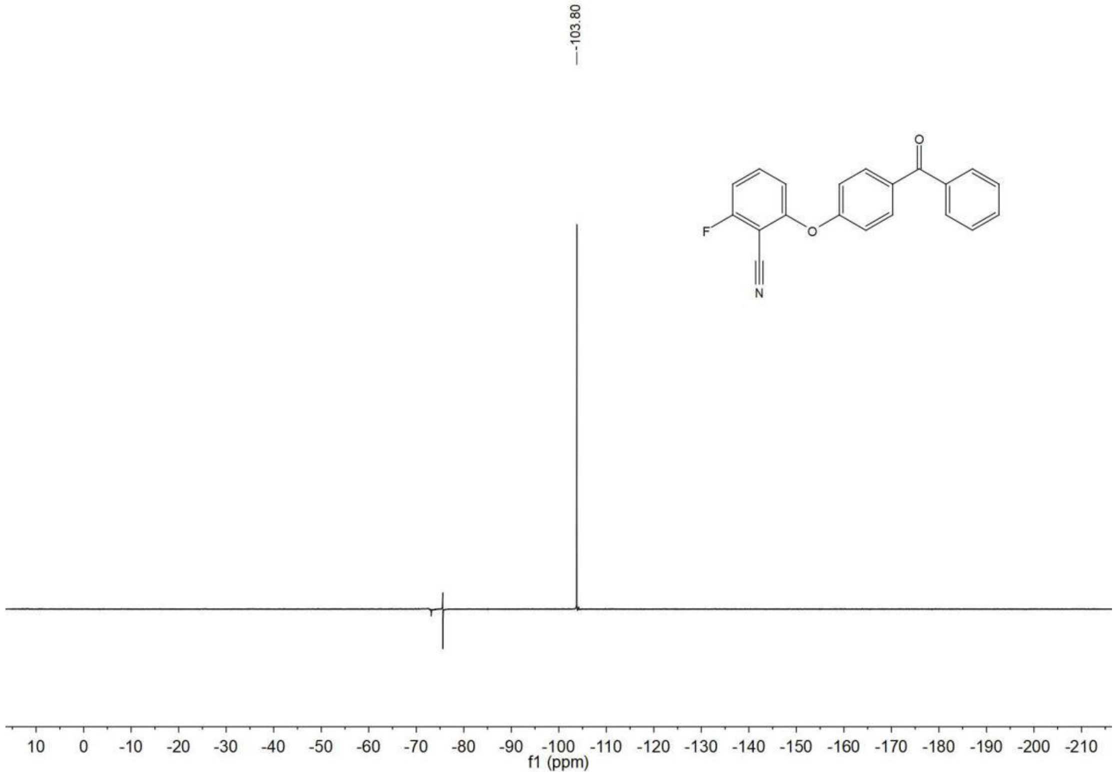

--103.80
F
O
O
N
10 0 -10 -20 -30 -40 -50 -60 -70 -80 -90 -100 -110 -120 -130 -140 -150 -160 -170 -180 -190 -200 -210
f1 (ppm)

^{1}H NMR (**c22**)

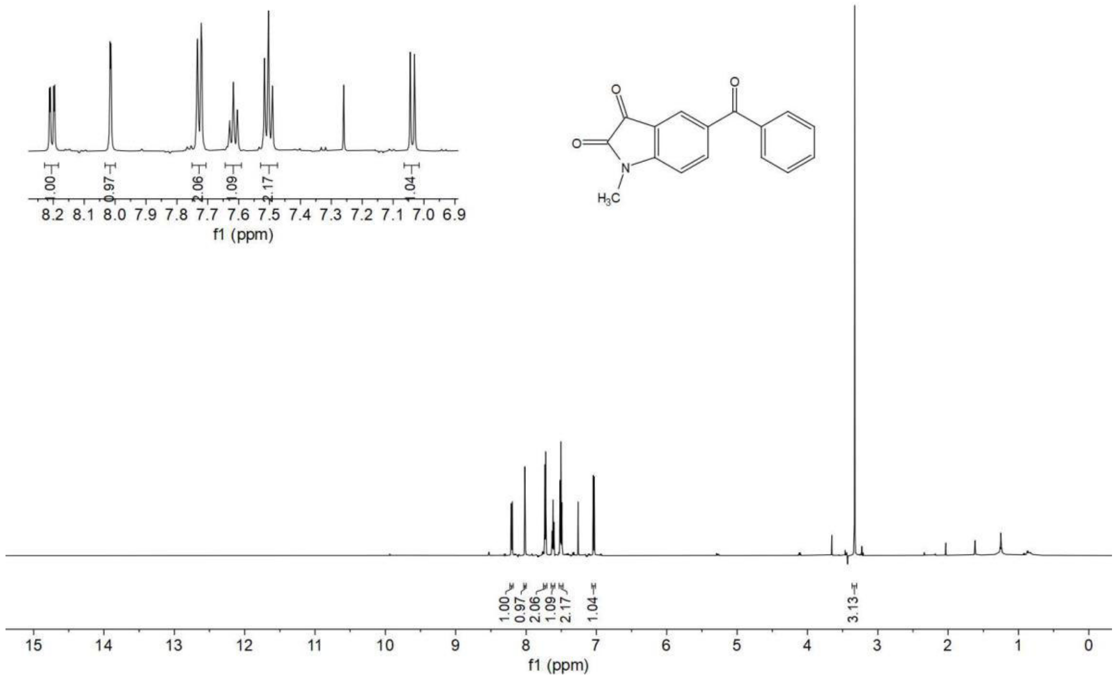

^{13}C NMR (**c22**)

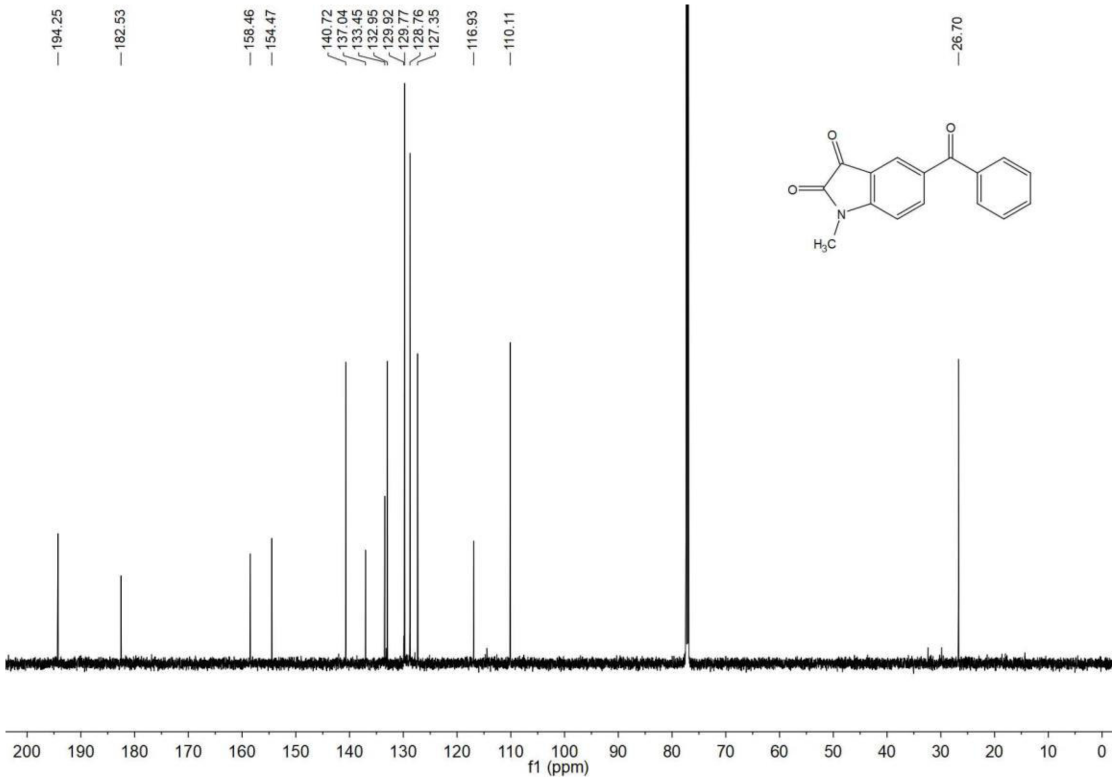

^{1}H NMR (**c23**)

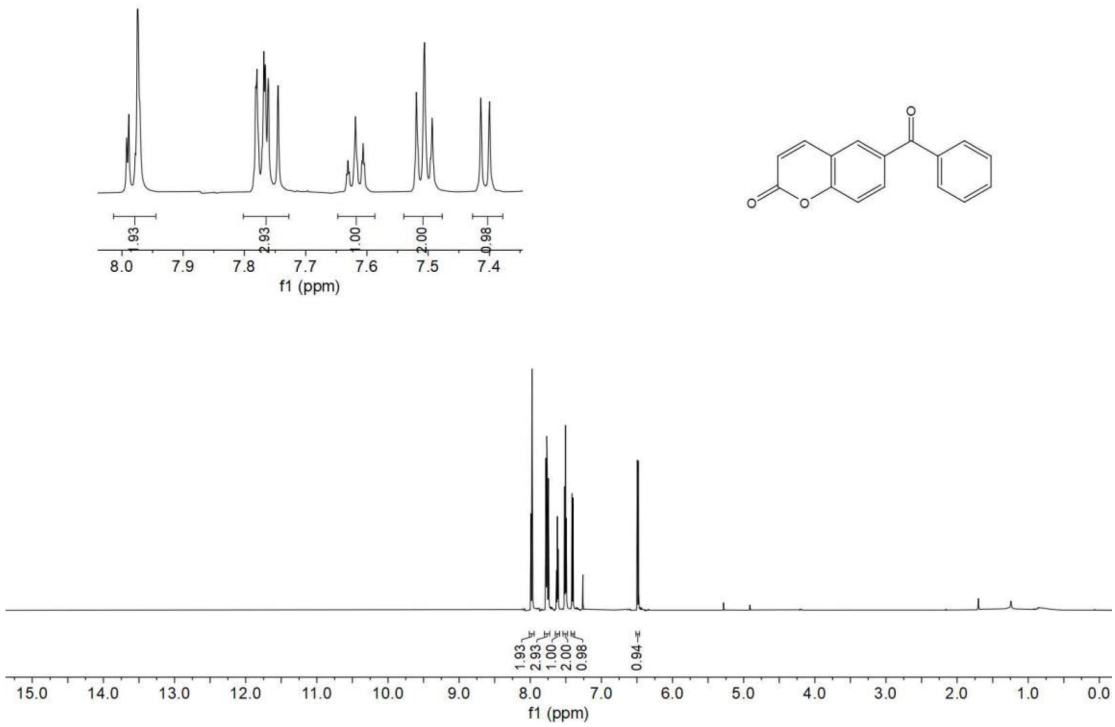

^{13}C NMR (**c23**)

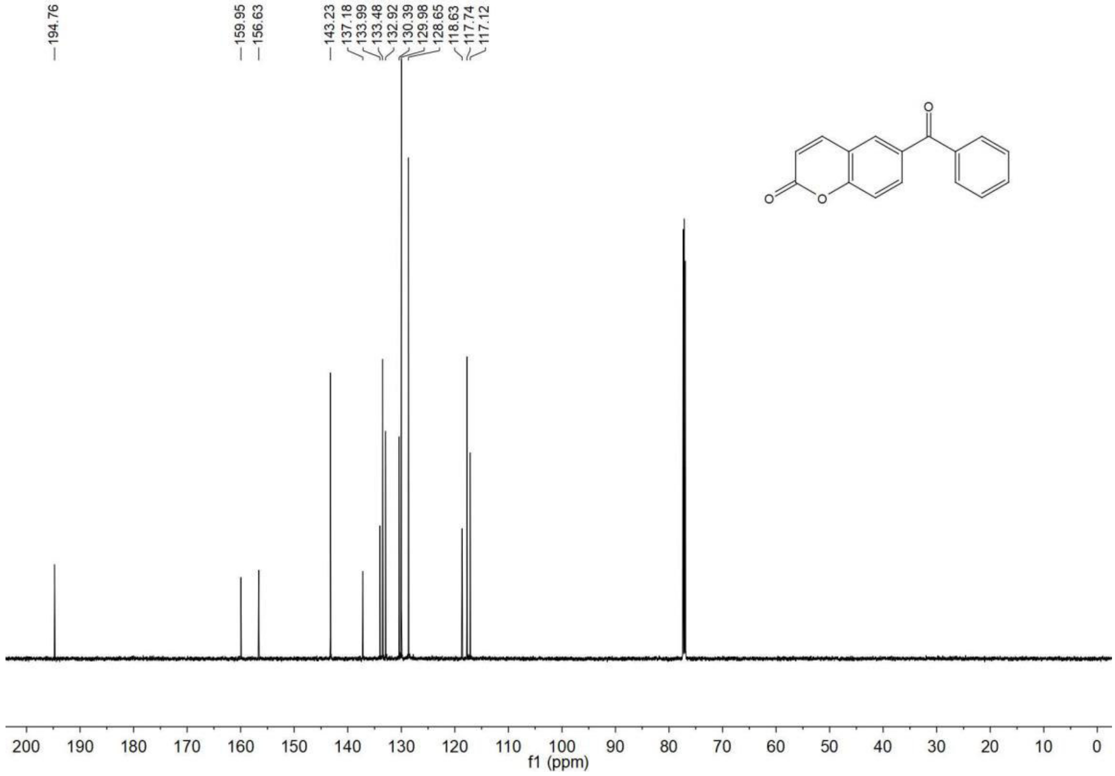

^{1}H NMR (**c24**)

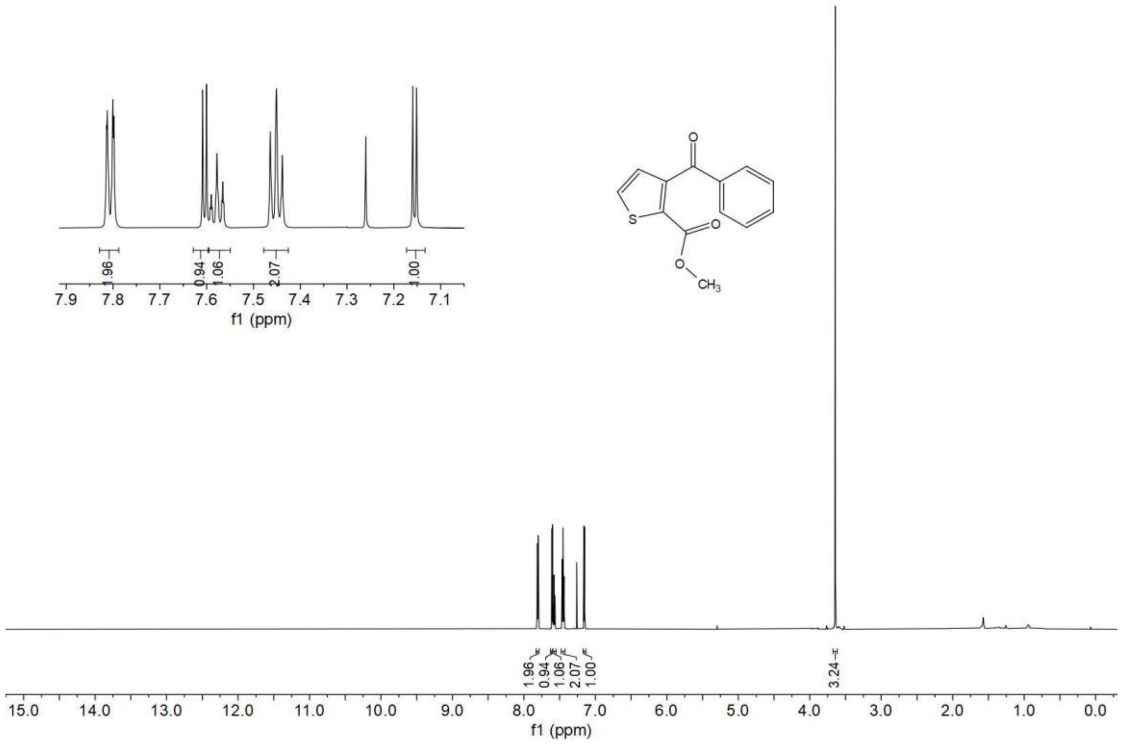

^{13}C NMR (**c24**)

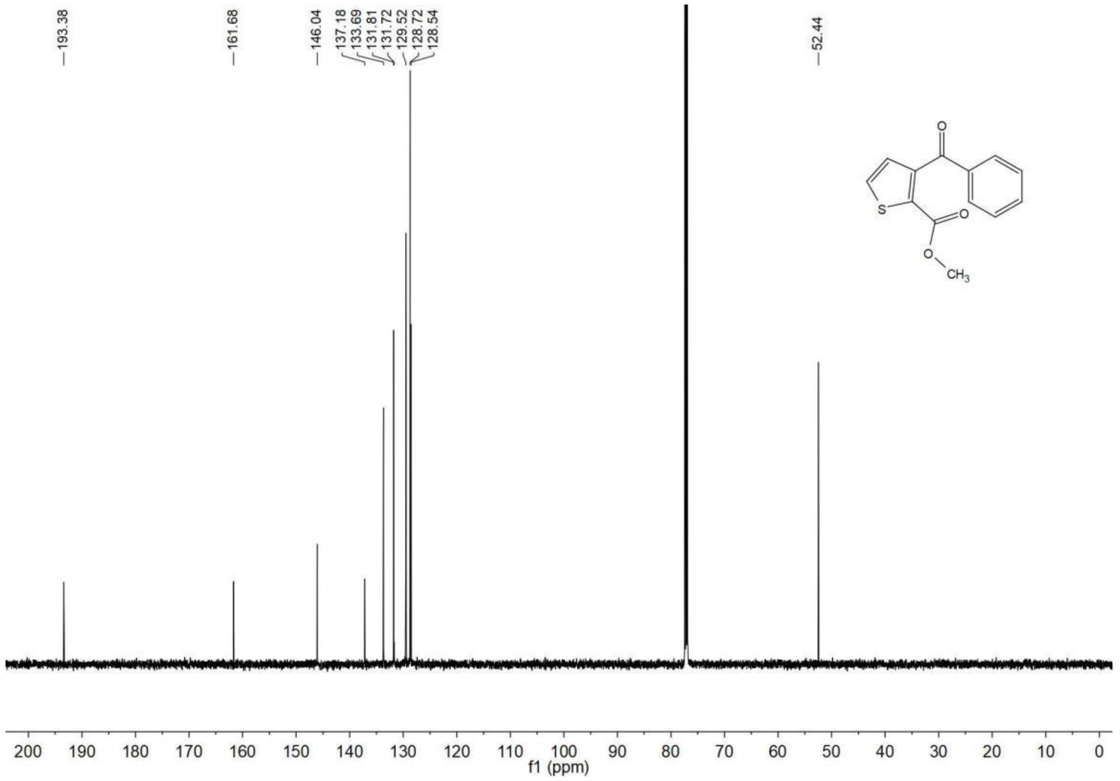

^{1}H NMR (**c25**)

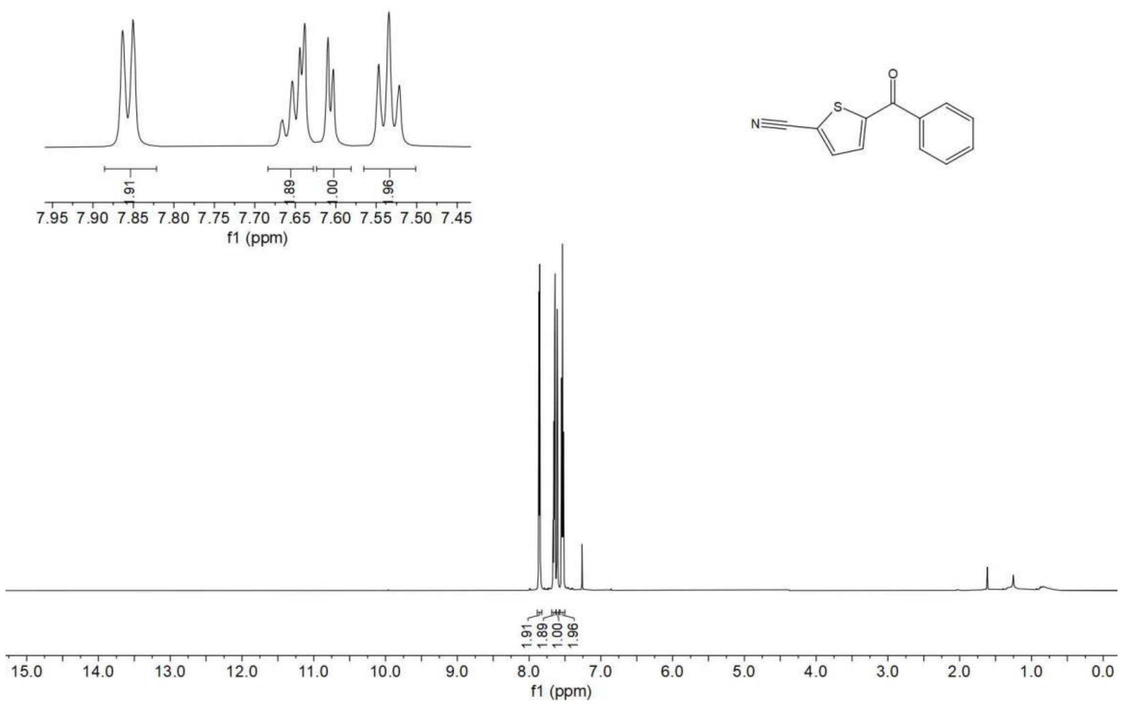

^{13}C NMR (**c25**)

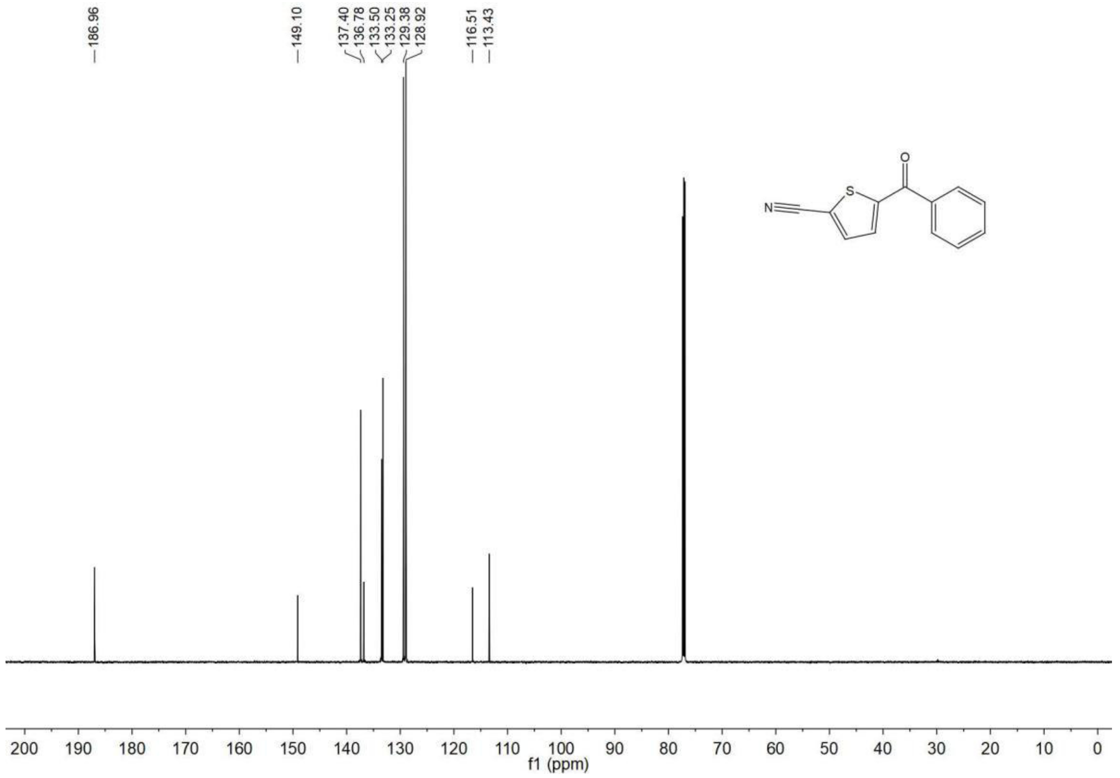

^{1}H NMR (**c26**)

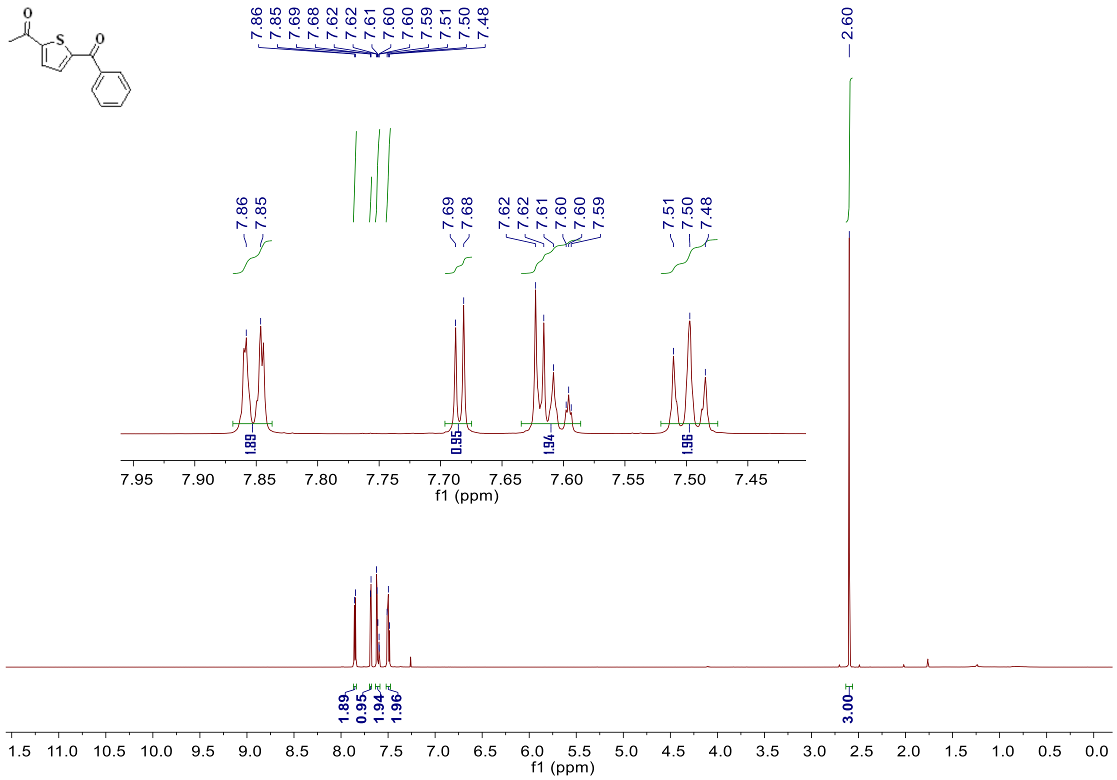

^{13}C NMR (**c26**)

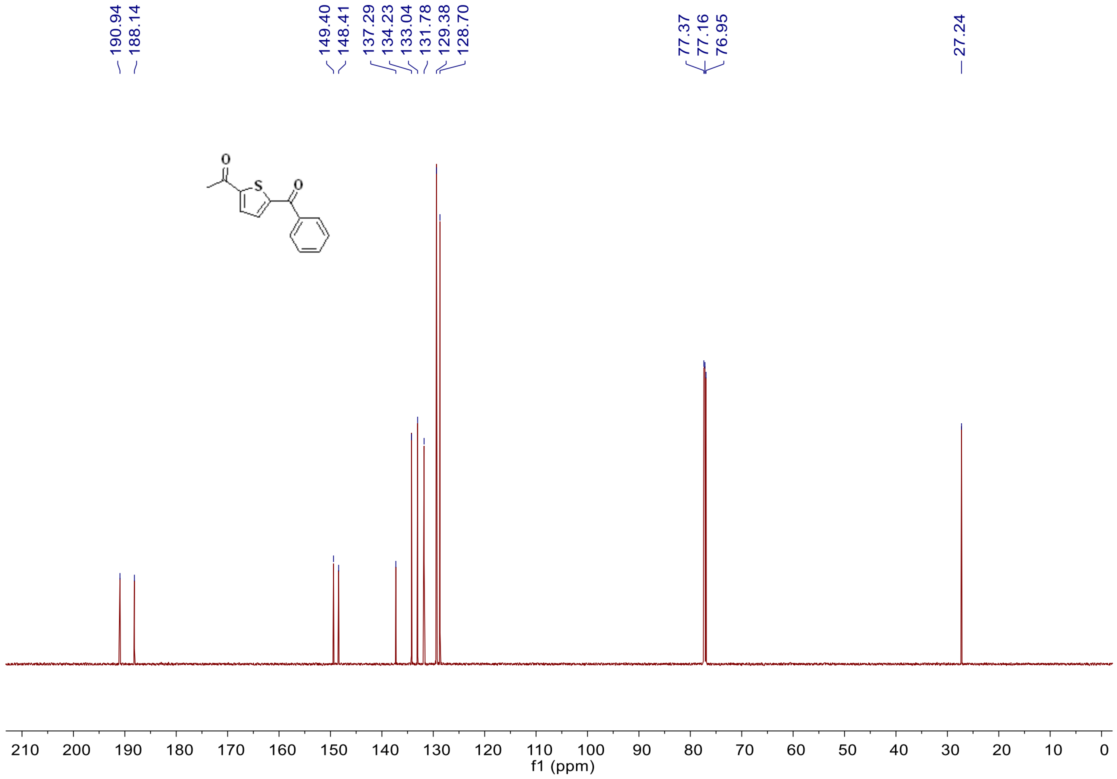

^{1}H NMR (**c27**)

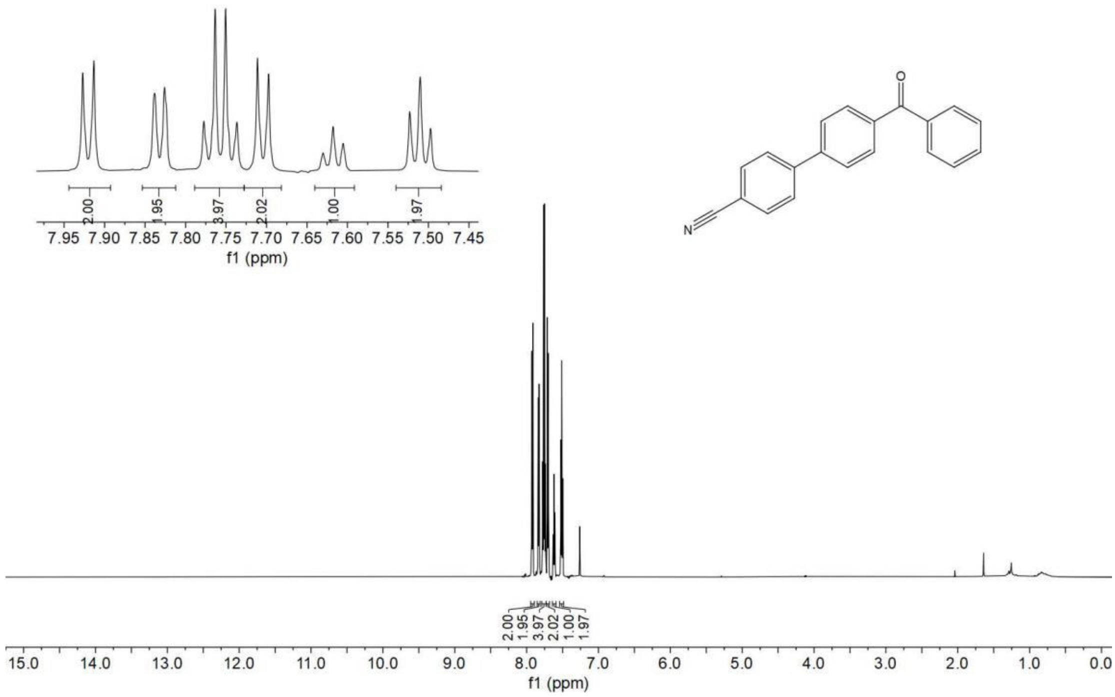

^{13}C NMR (**c27**)

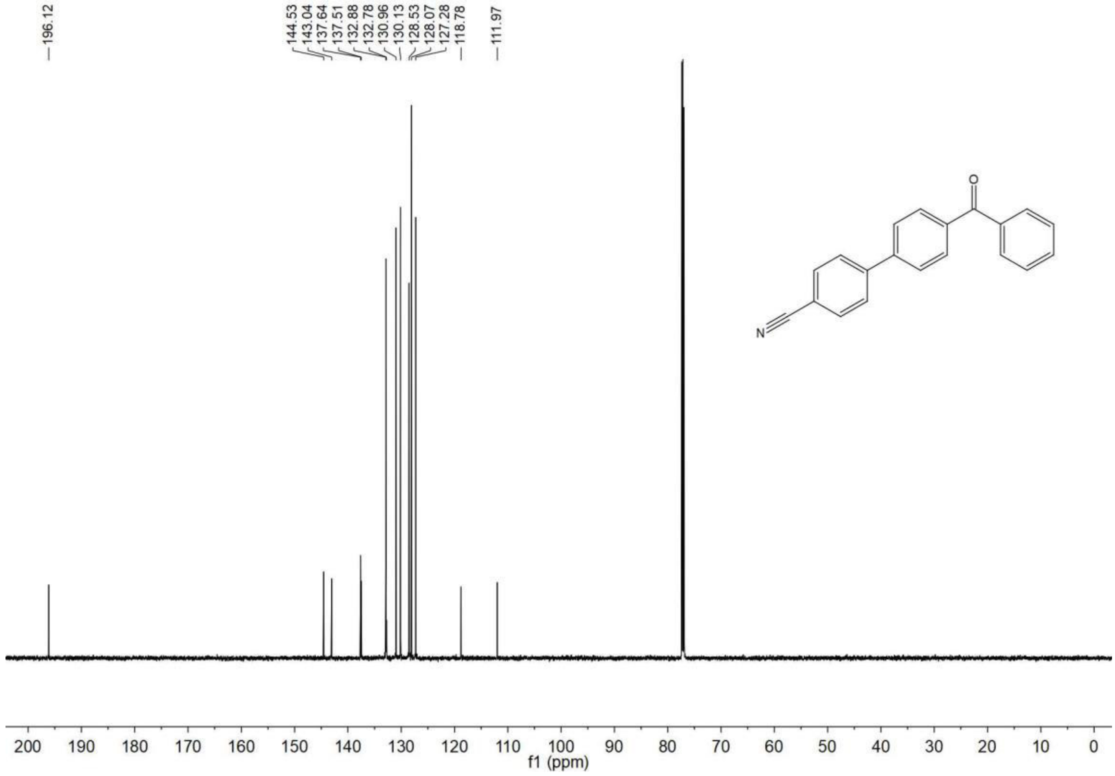

^{1}H NMR (**c28**)

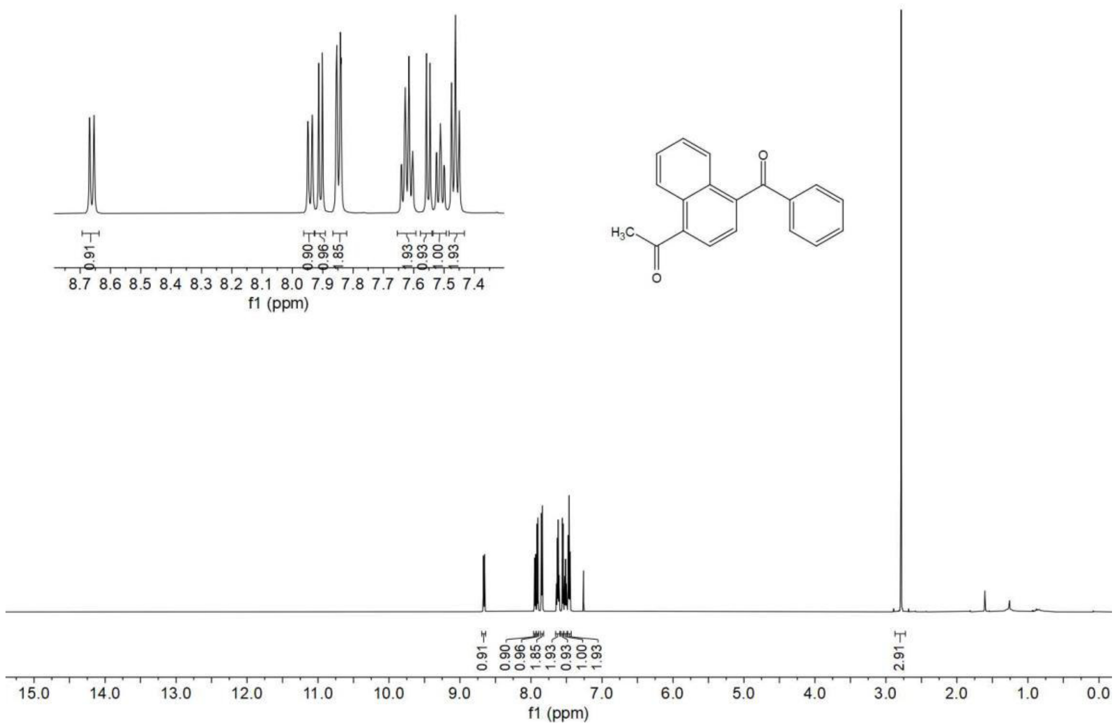

^{13}C NMR (**c28**)

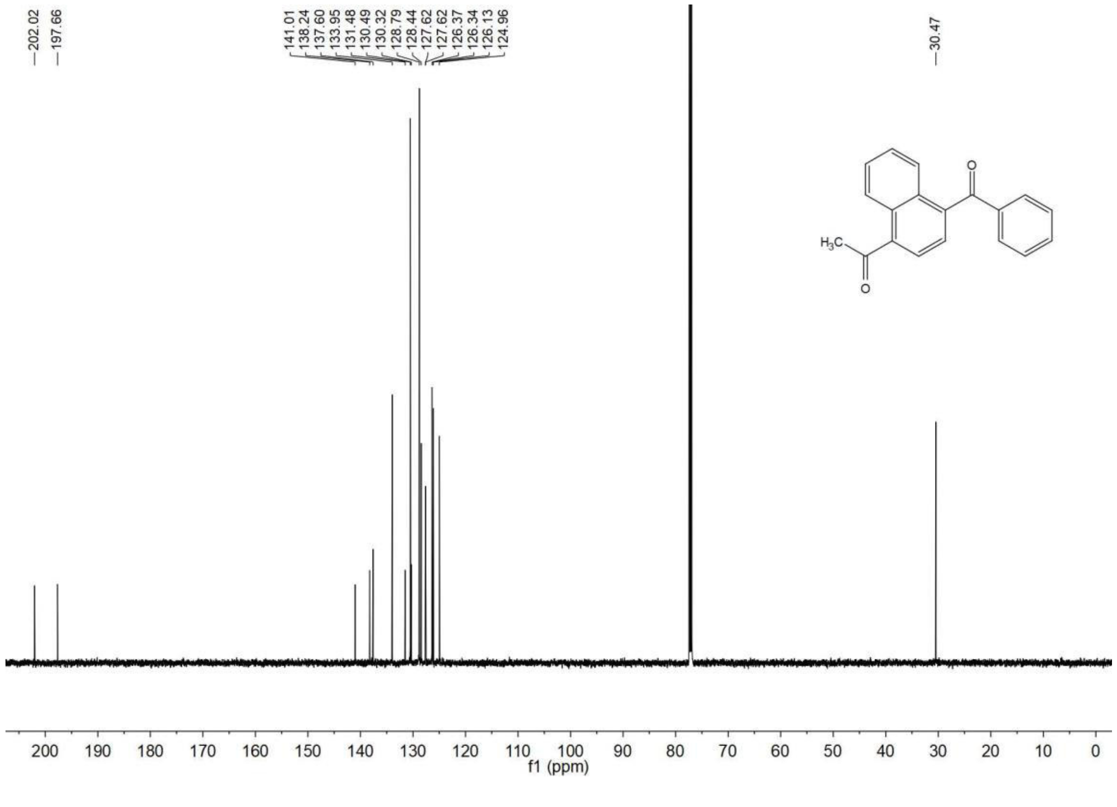

^{1}H NMR (**c29**)

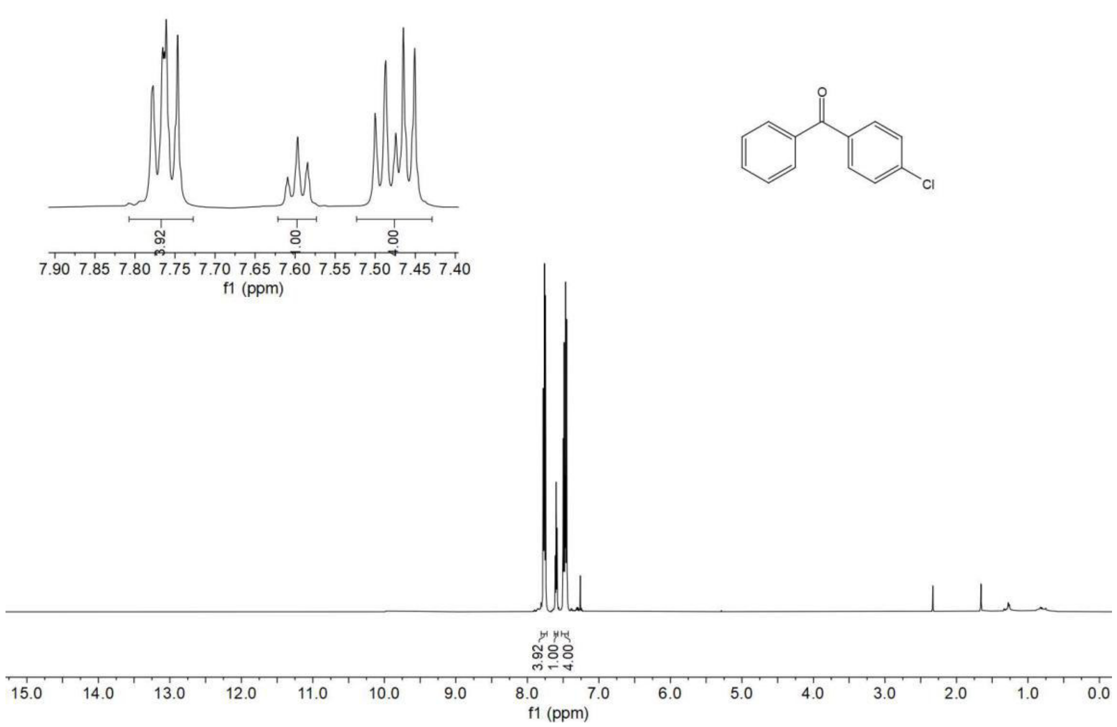

^{13}C NMR (**c29**)

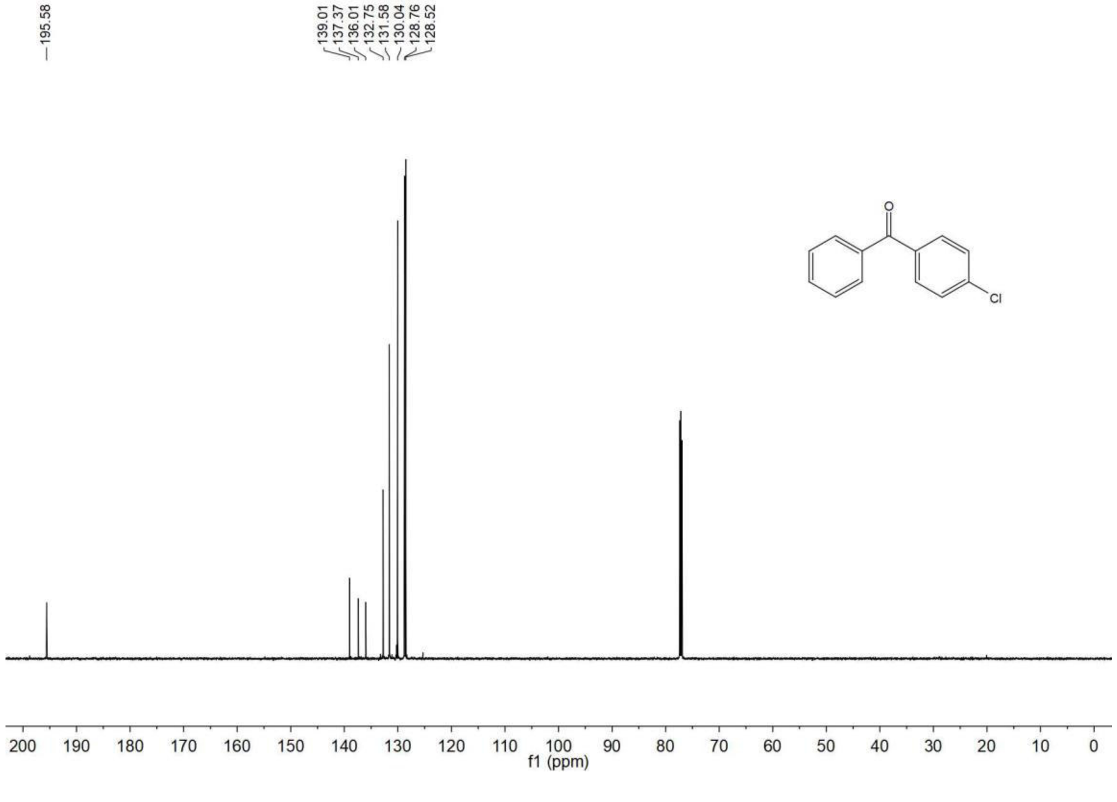

^{1}H NMR (**c30**)

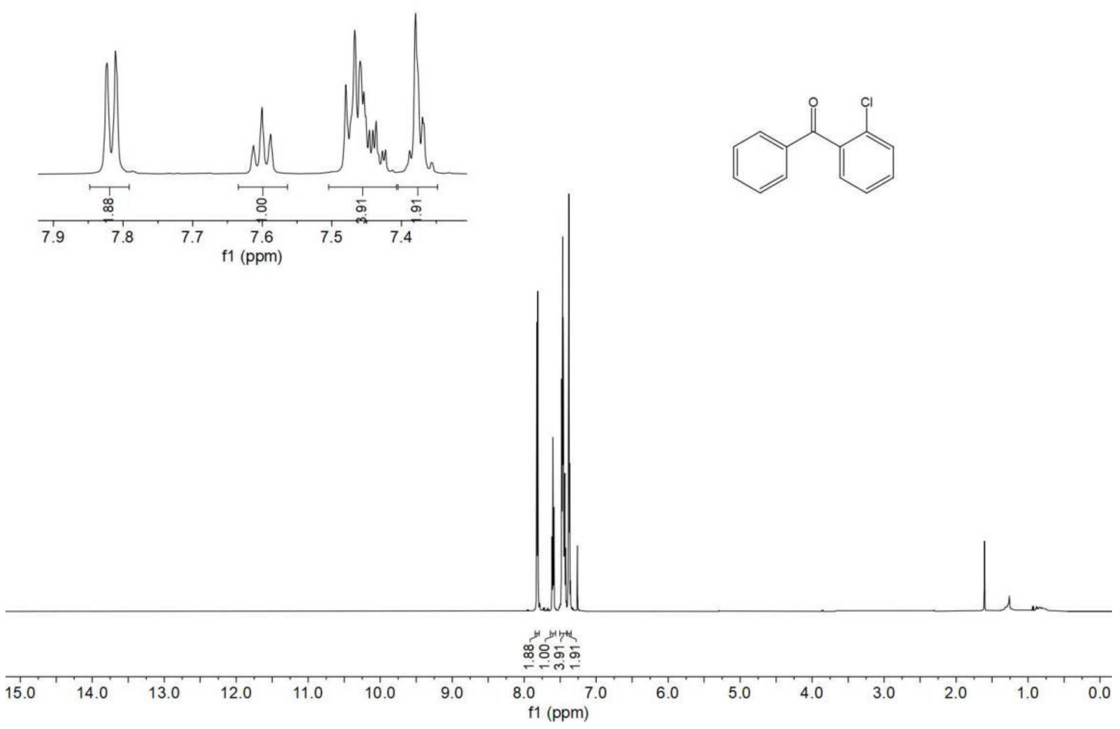

^{13}C NMR (**c30**)

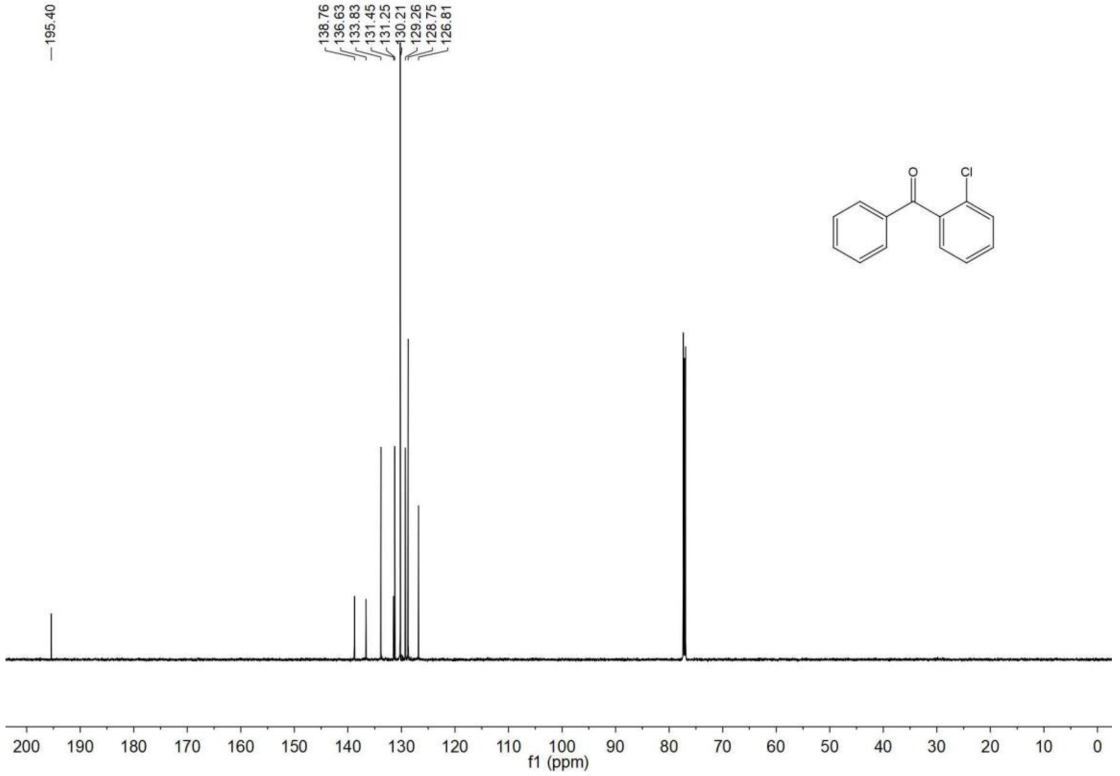

^{1}H NMR (**c31**)

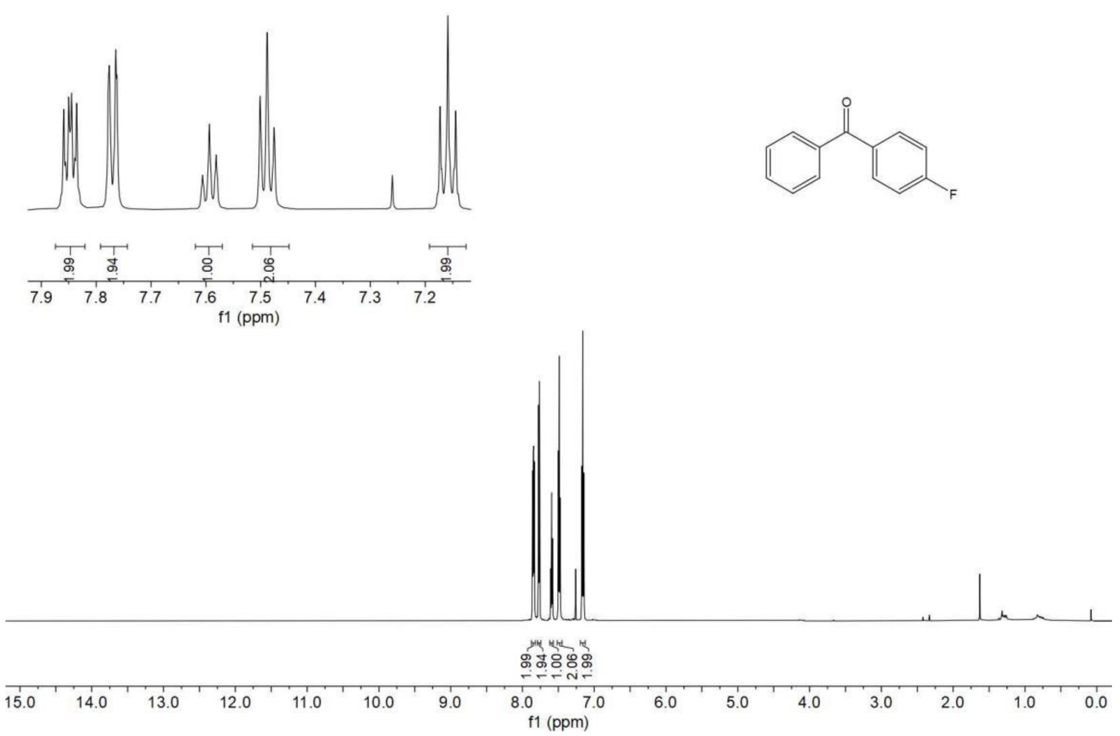

^{13}C NMR (**c31**)

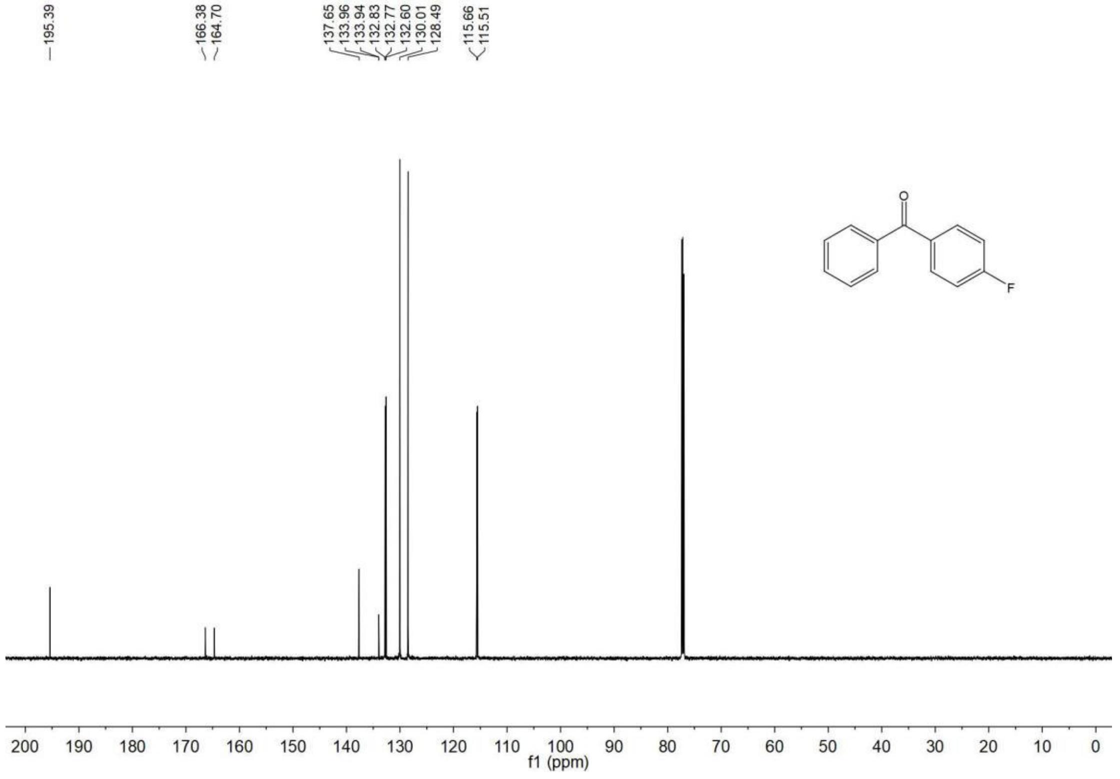

^{19}F NMR (**c31**)

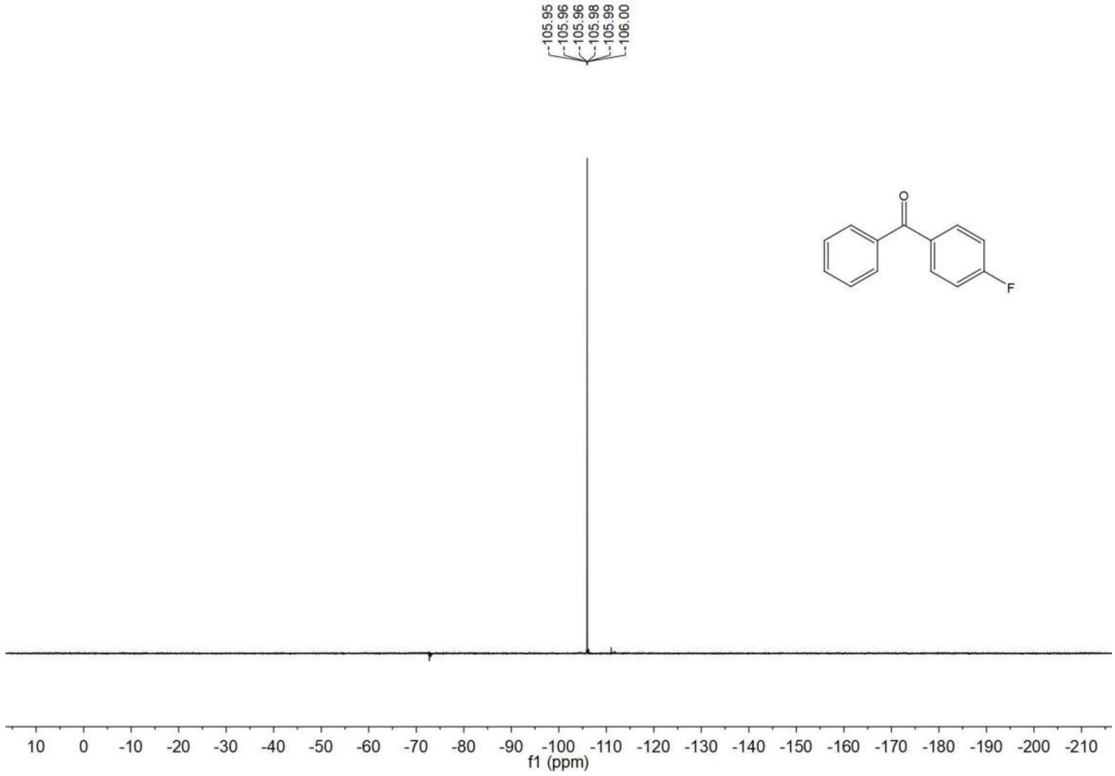

-105.95
-105.96
-105.96
-105.98
-105.99
-106.00
O
F
10 0 -10 -20 -30 -40 -50 -60 -70 -80 -90 -100 -110 -120 -130 -140 -150 -160 -170 -180 -190 -200 -210
f1 (ppm)

^{1}H NMR (**c32**)

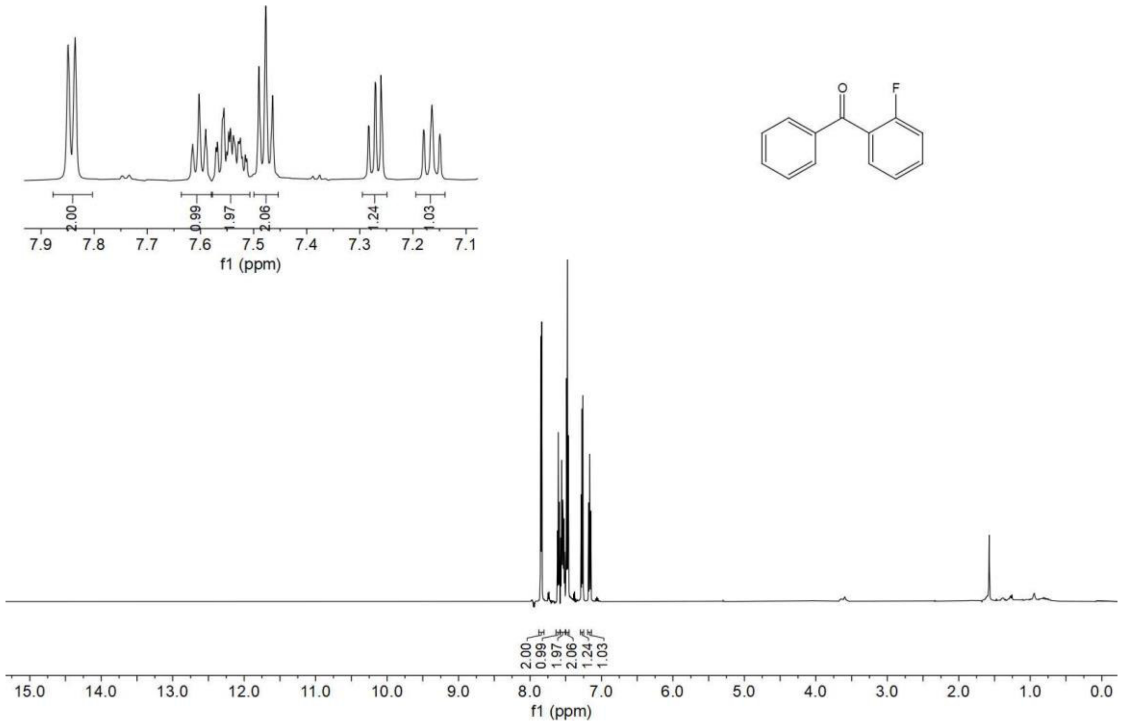

^{13}C NMR (**c32**)

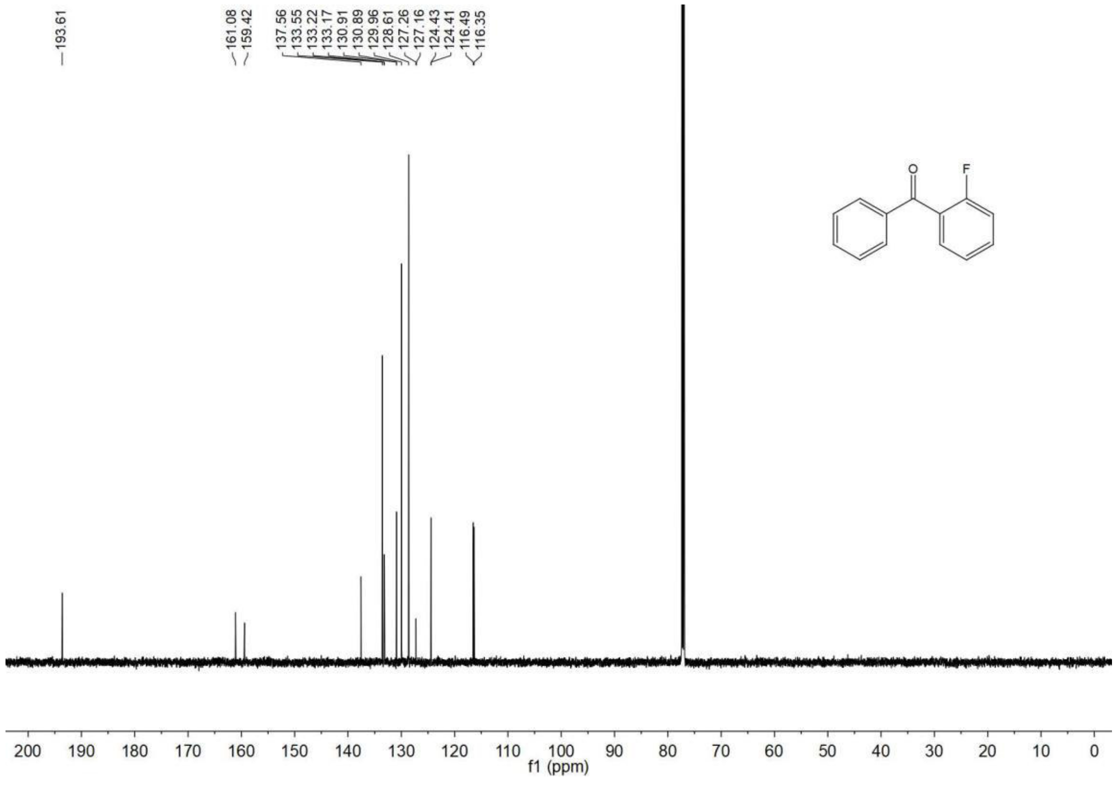

^{19}F NMR (**c32**)

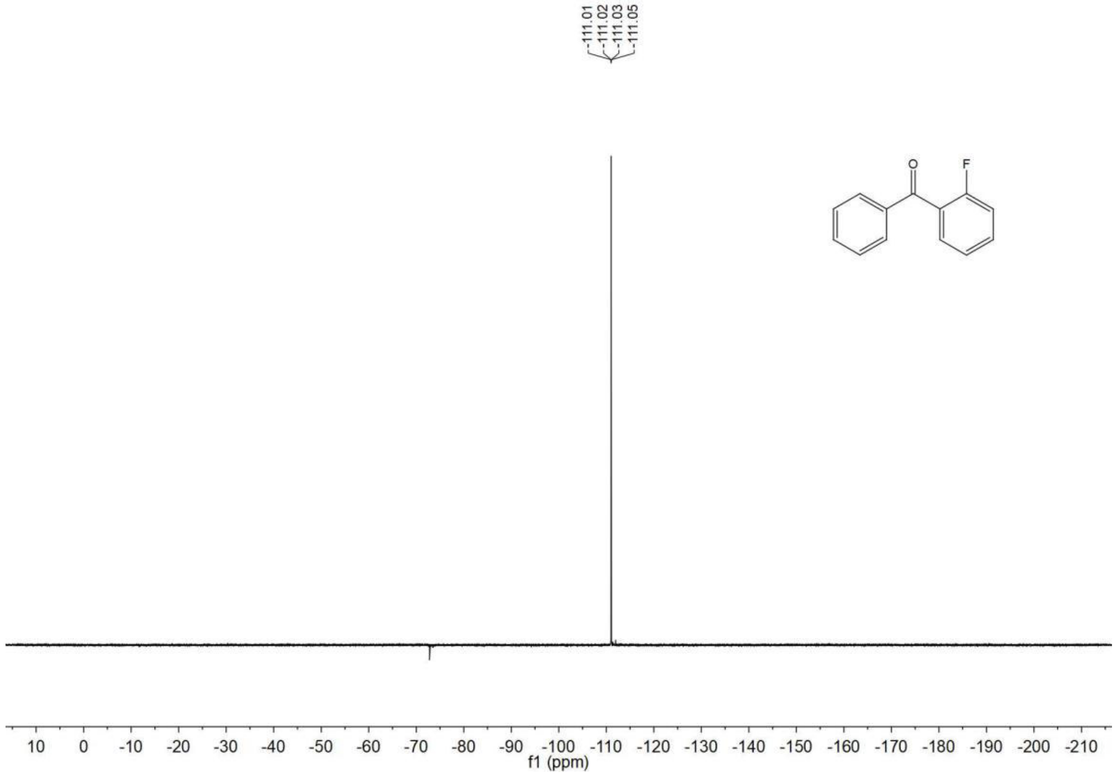

-111.01
-111.02
-111.03
-111.05
O
F
10 0 -10 -20 -30 -40 -50 -60 -70 -80 -90 -100 -110 -120 -130 -140 -150 -160 -170 -180 -190 -200 -210
f1 (ppm)

^{1}H NMR (**c33**)

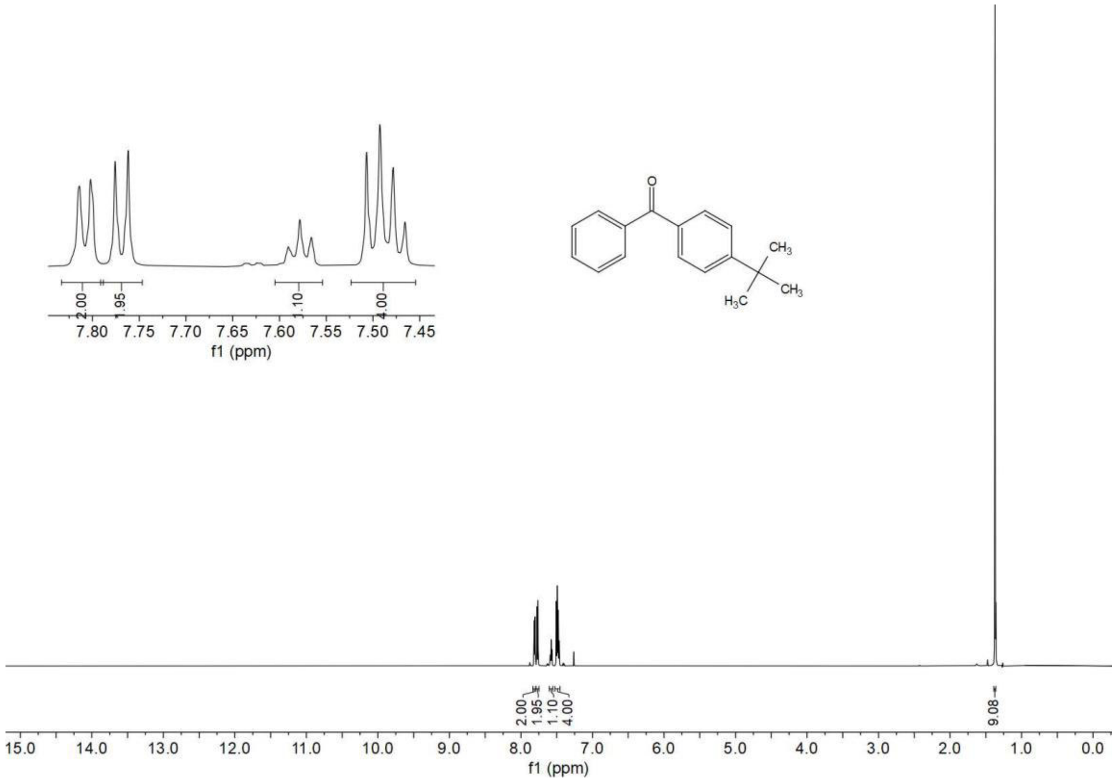

^{13}C NMR (**c33**)

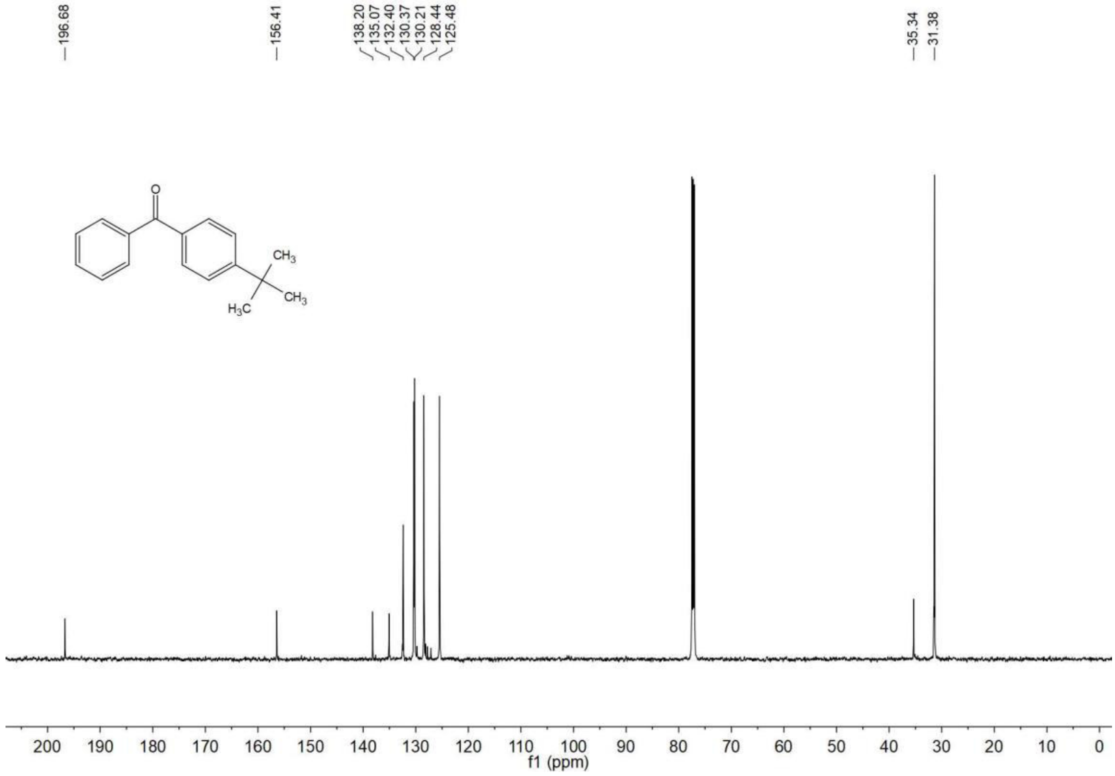

^{1}H NMR (**c34**)

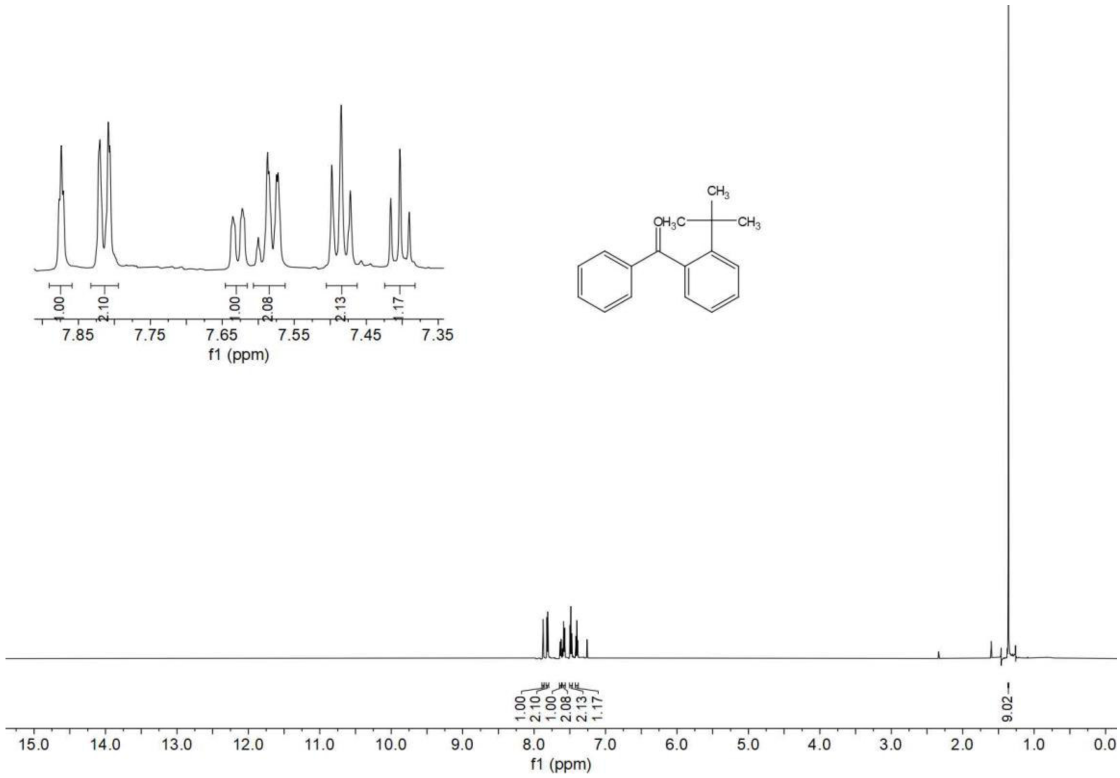

^{13}C NMR (**c34**)

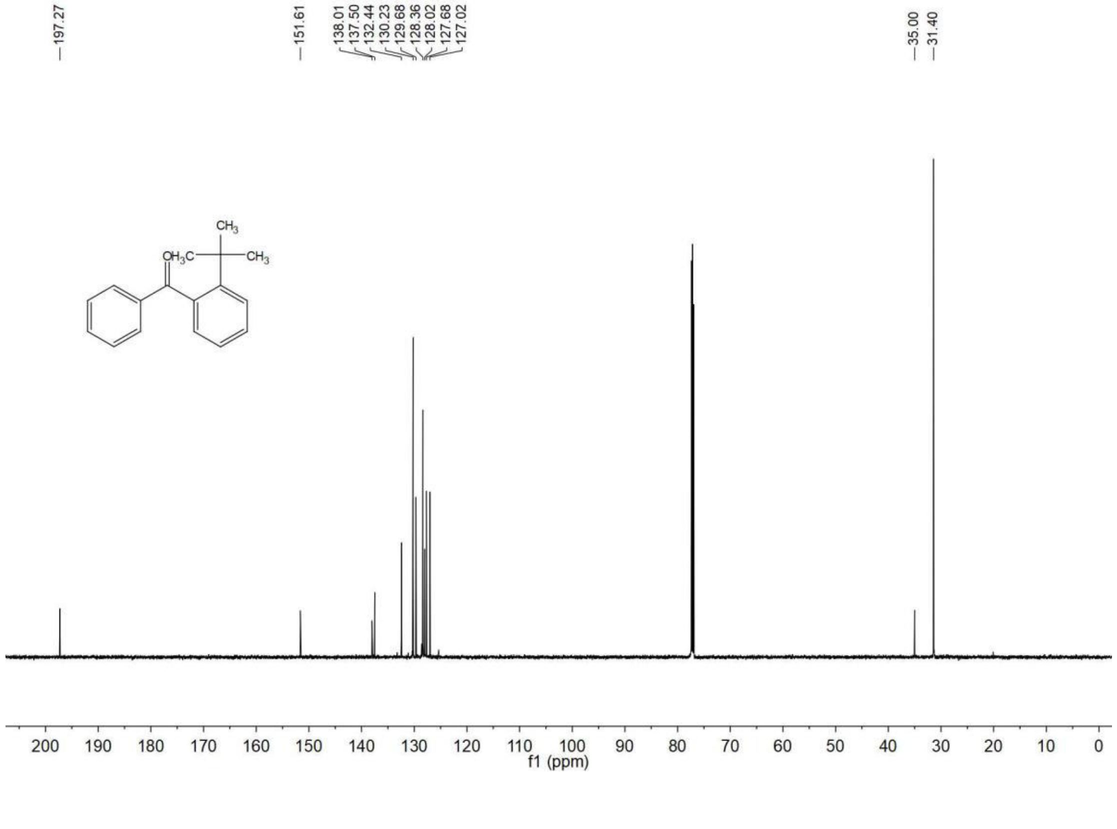

^{1}H NMR (**c36**)

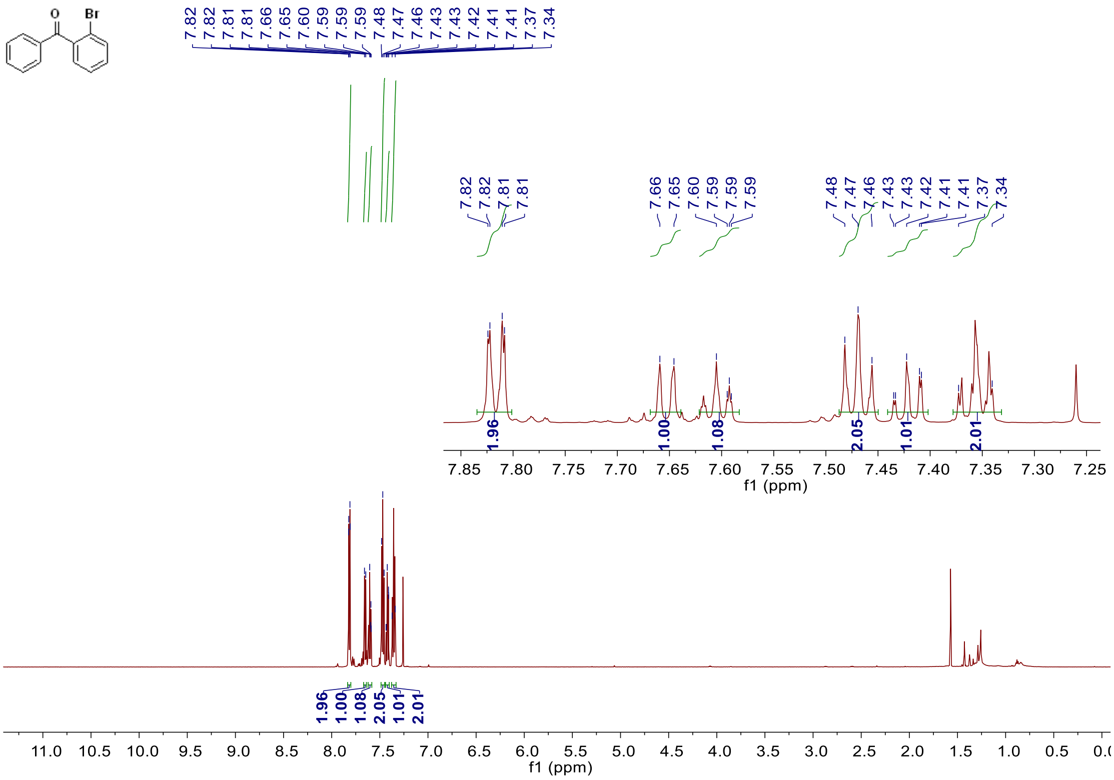

^{13}C NMR (**c36**)

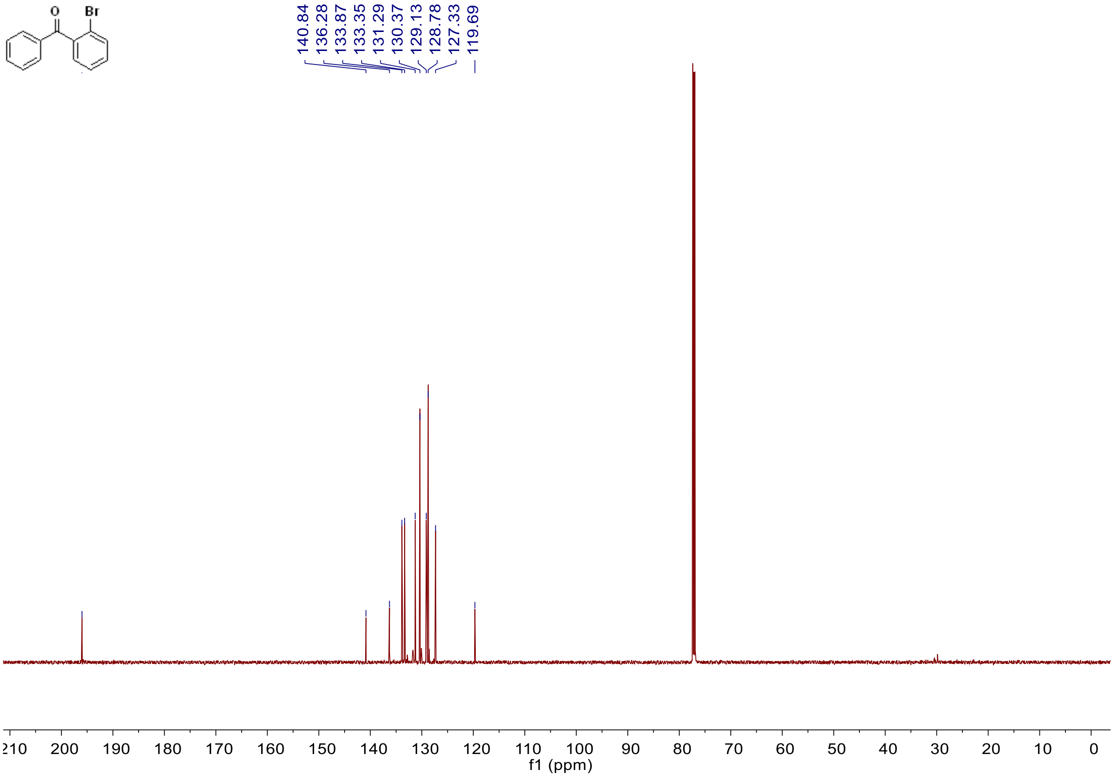

^{1}H NMR (**c37**)

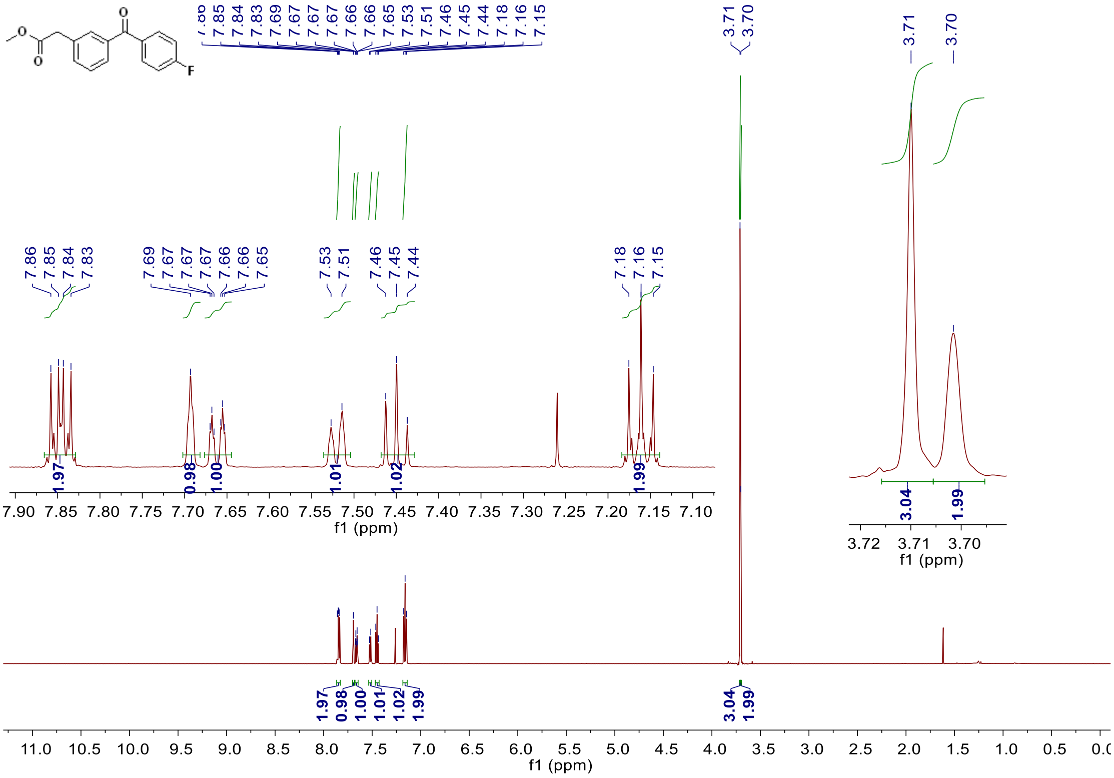

^{13}C NMR (**c37**)

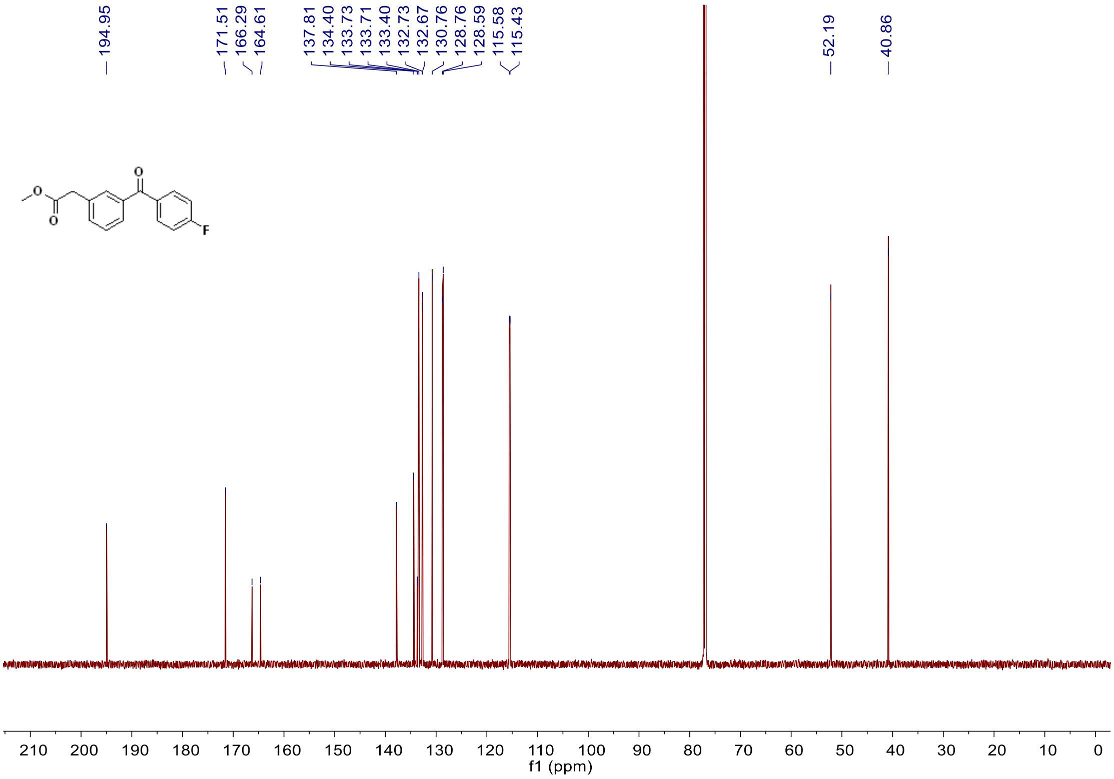

^{19}F NMR (**c37**)

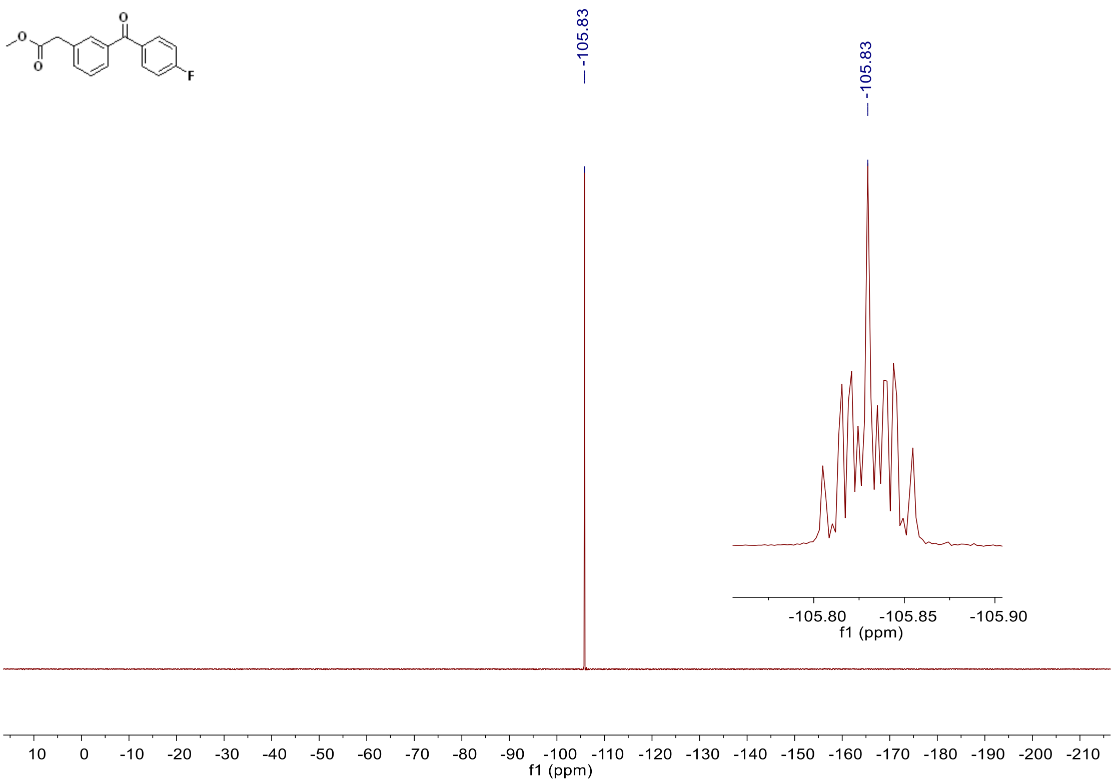

^{1}H NMR (**c38**)

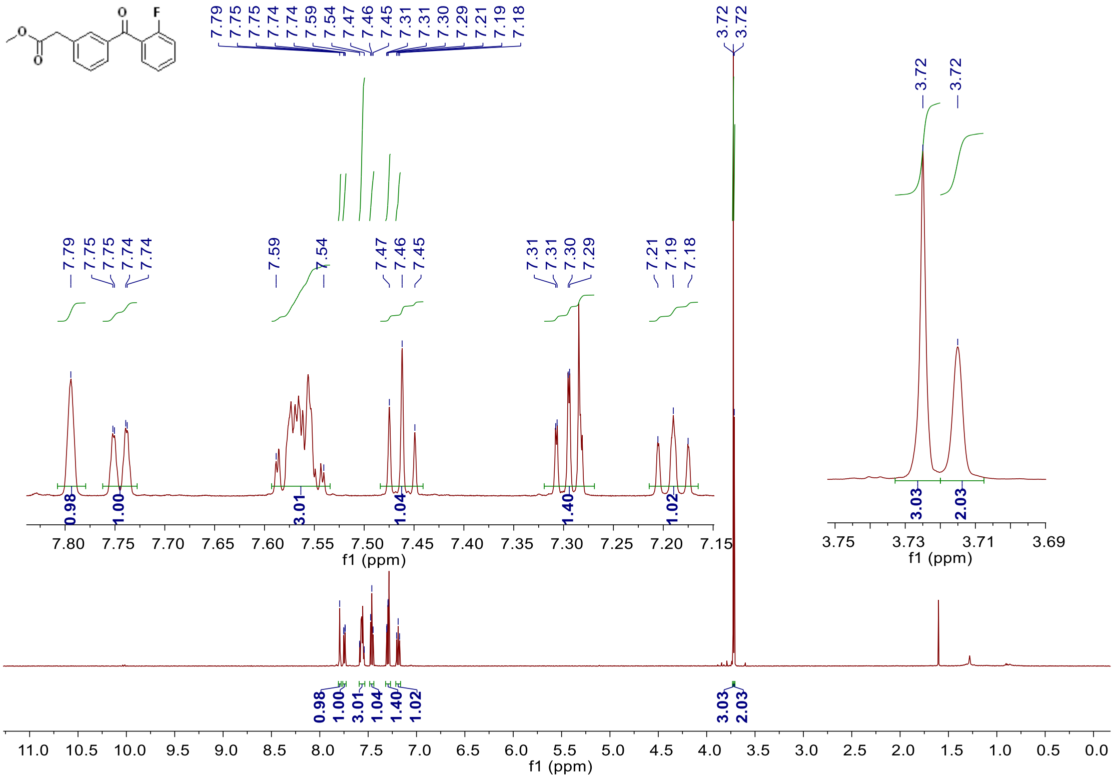

^{13}C NMR (**c38**)

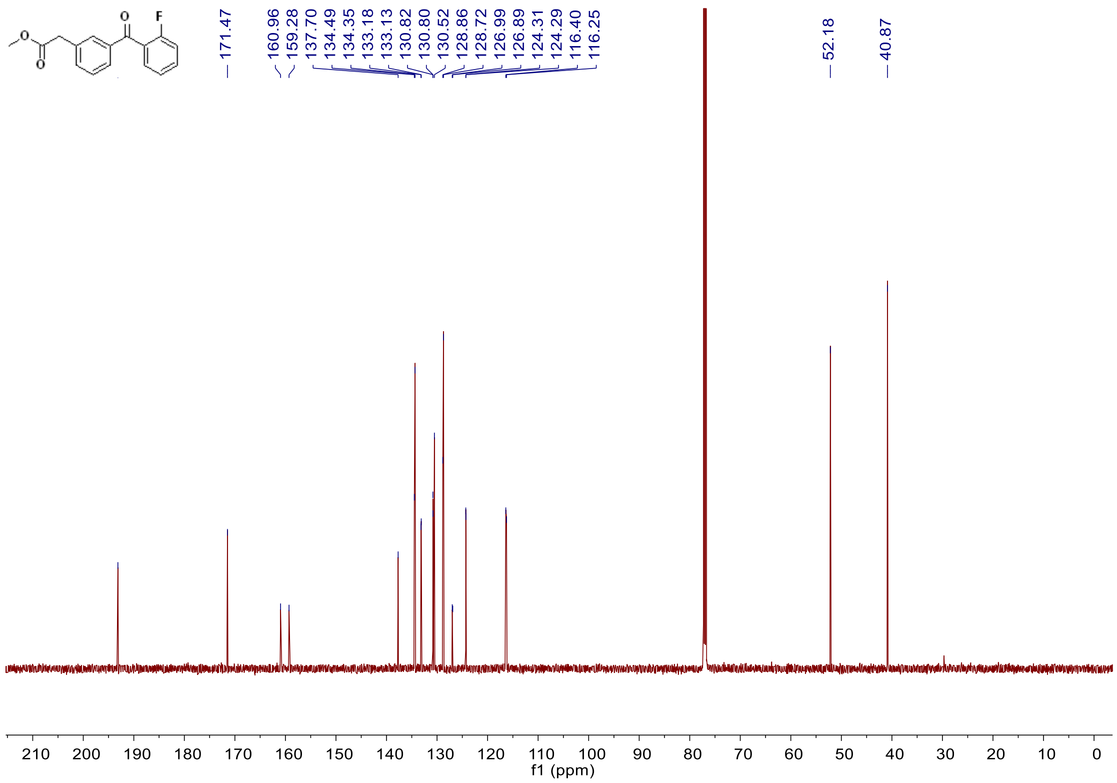

^{19}F NMR (**c38**)

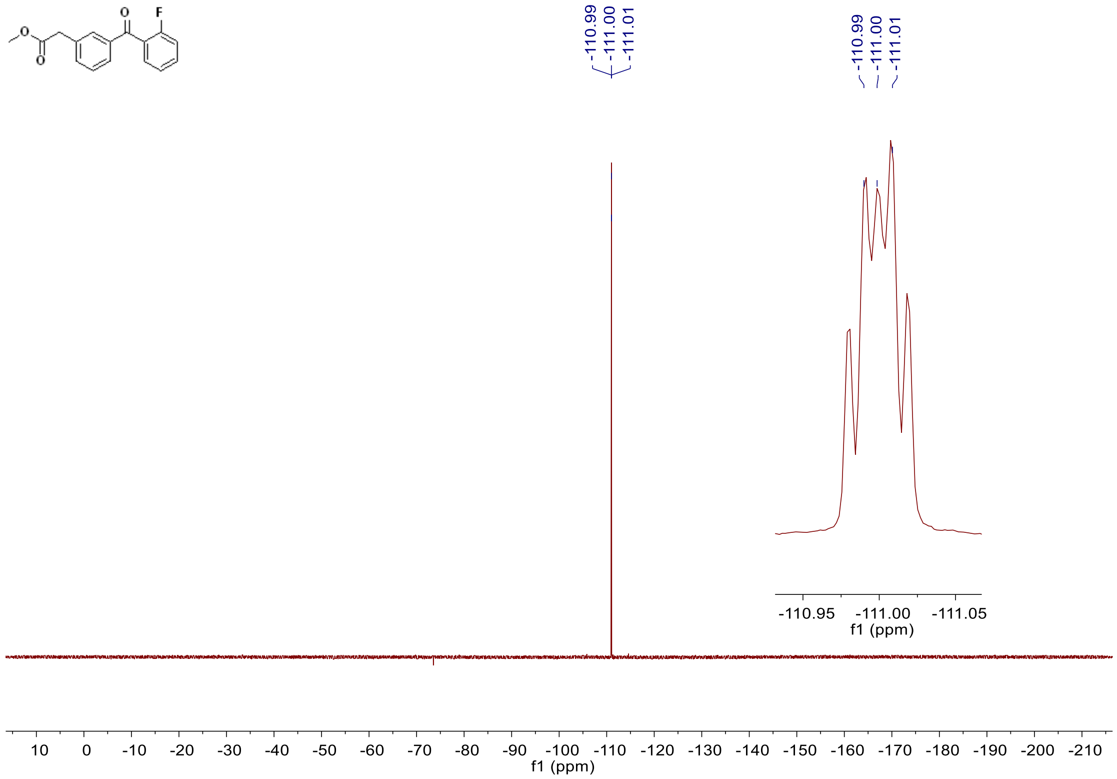

^{1}H NMR (**c39+c40**)

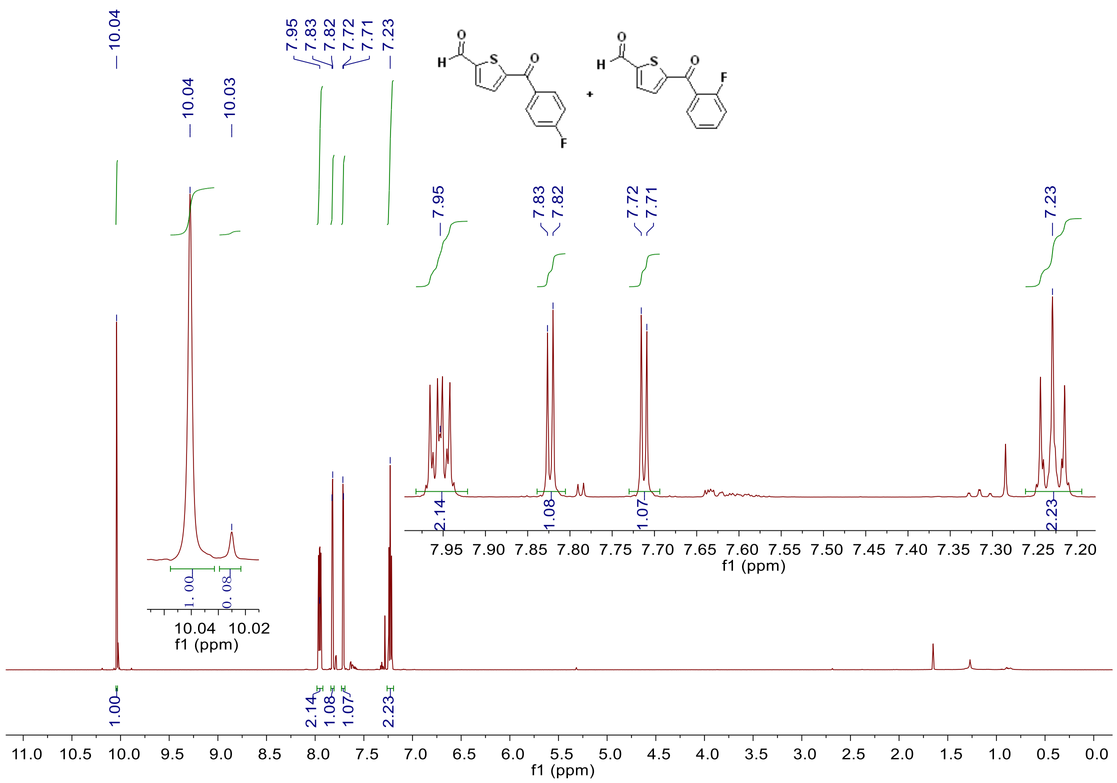

^{13}C NMR (**c39+c40**)

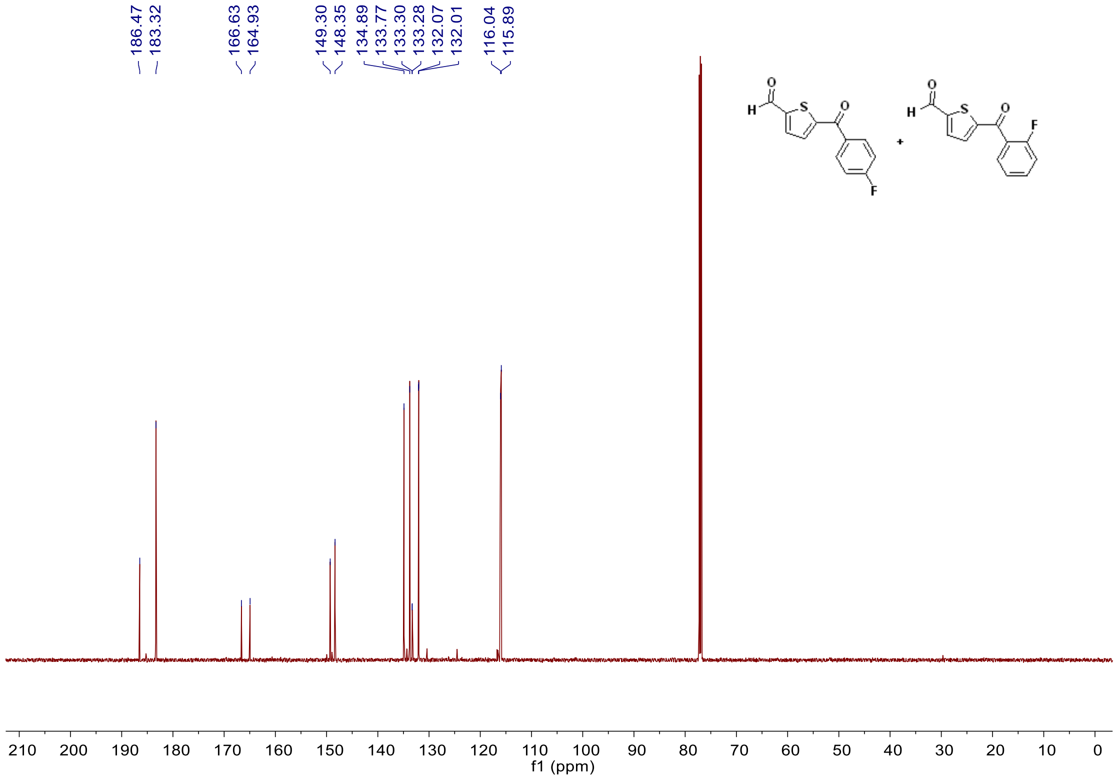

^{19}F NMR (**c39+c40**)

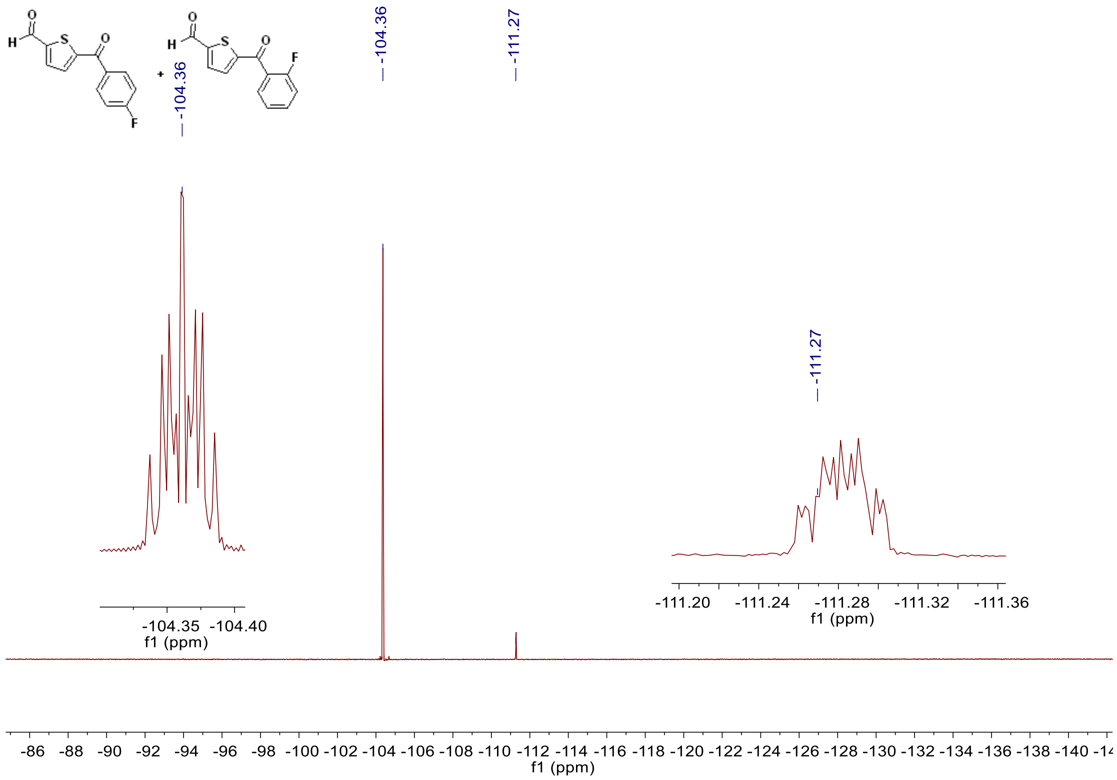

^{1}H NMR (**c41**)

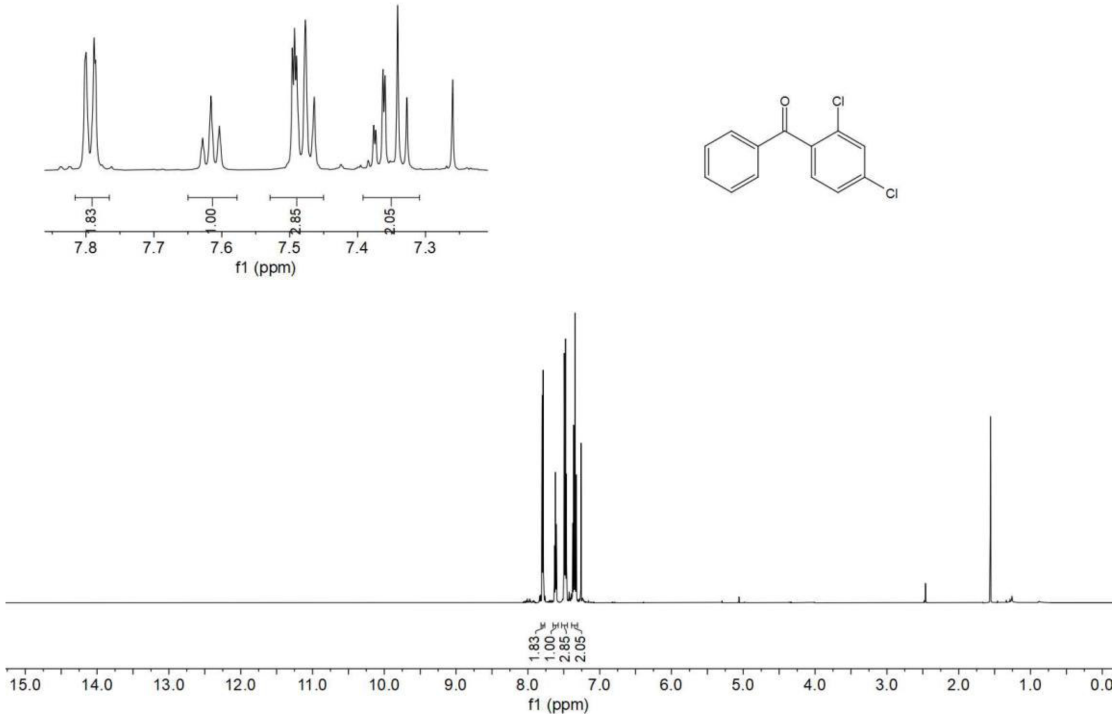

^{13}C NMR (**c41**)

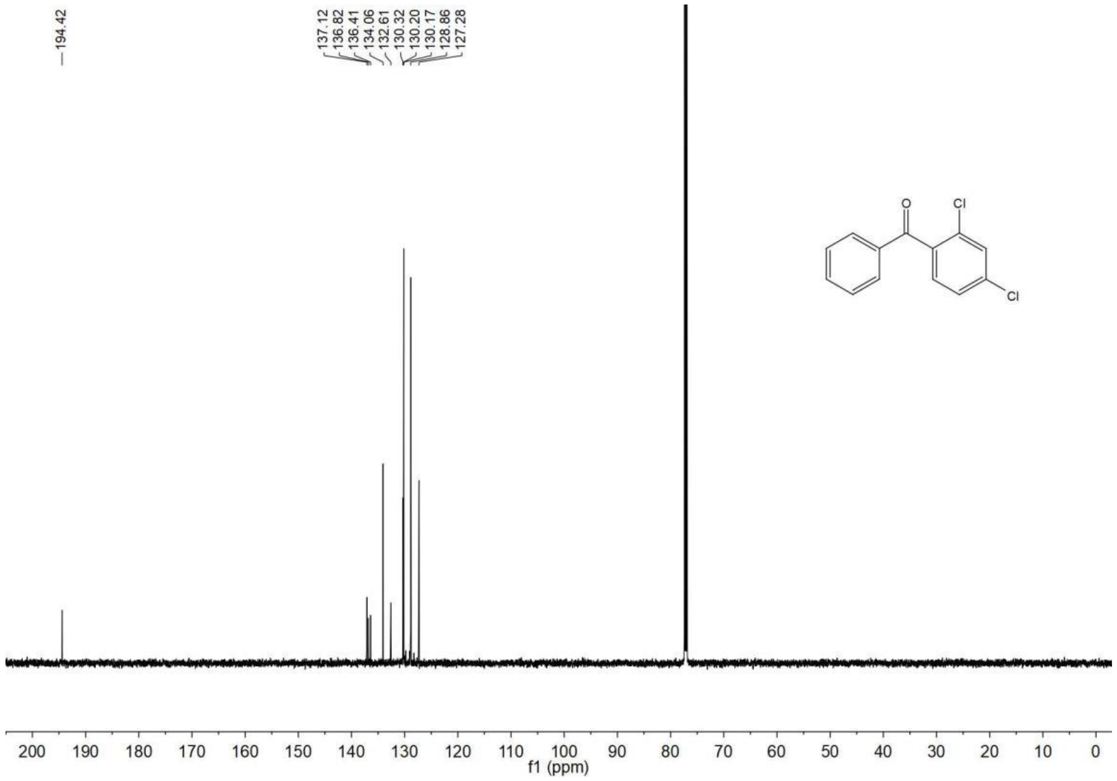

^{1}H NMR (**c42**)

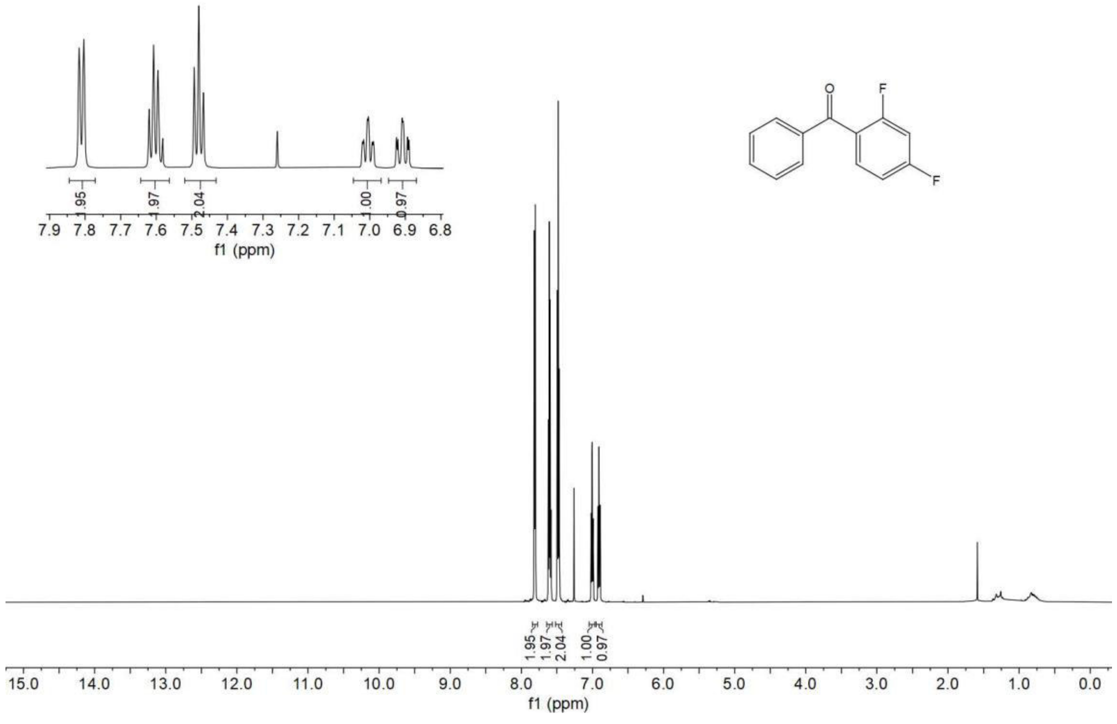

^{13}C NMR (**c42**)

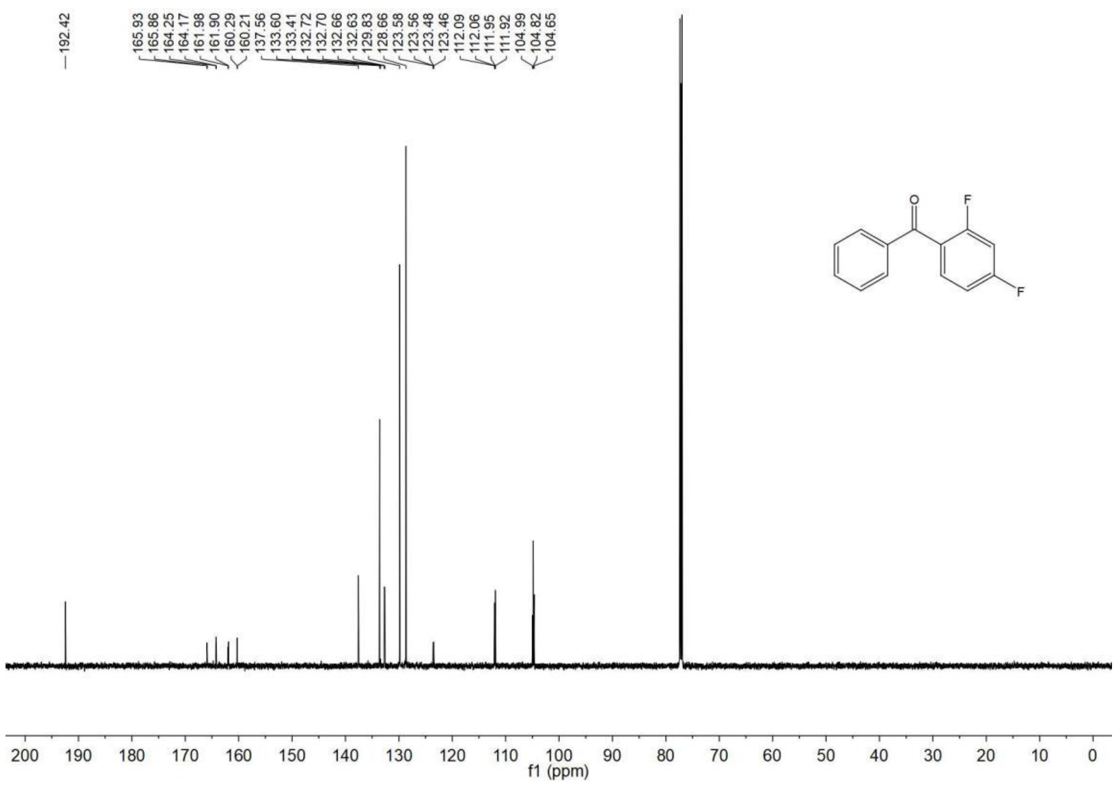

^{19}F NMR (**c42**)

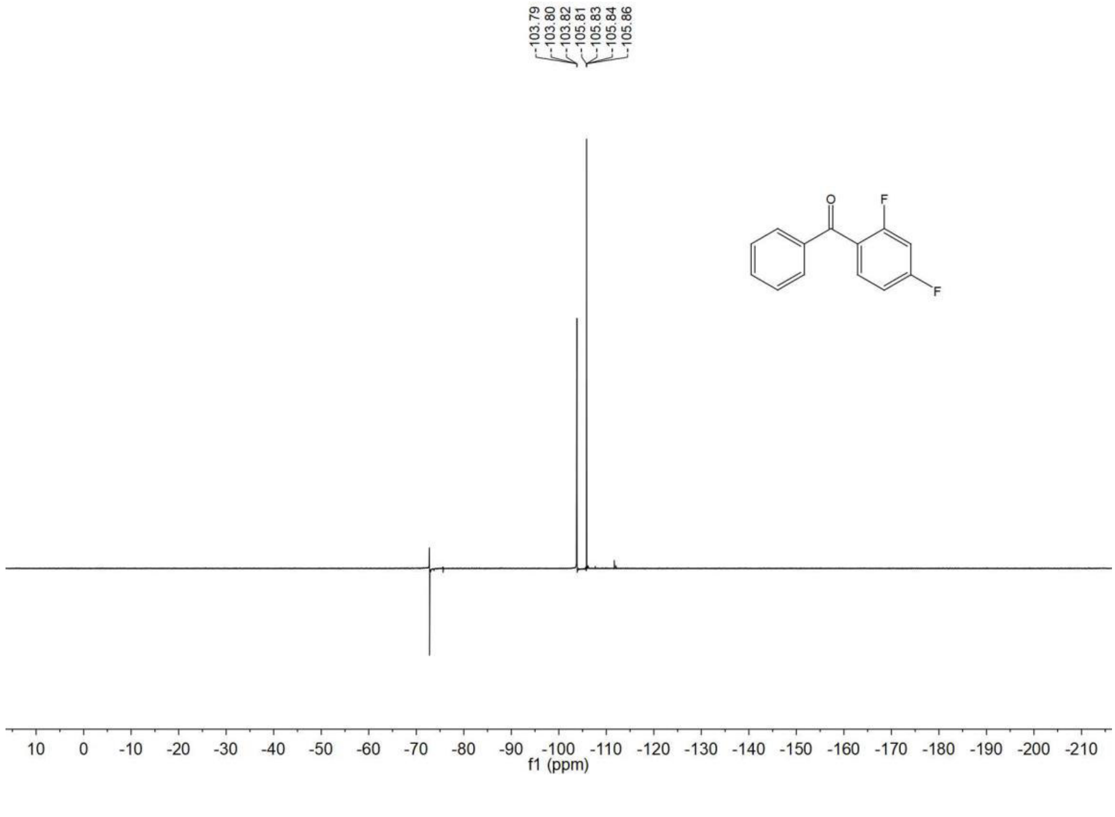

^{1}H NMR (**c43**)

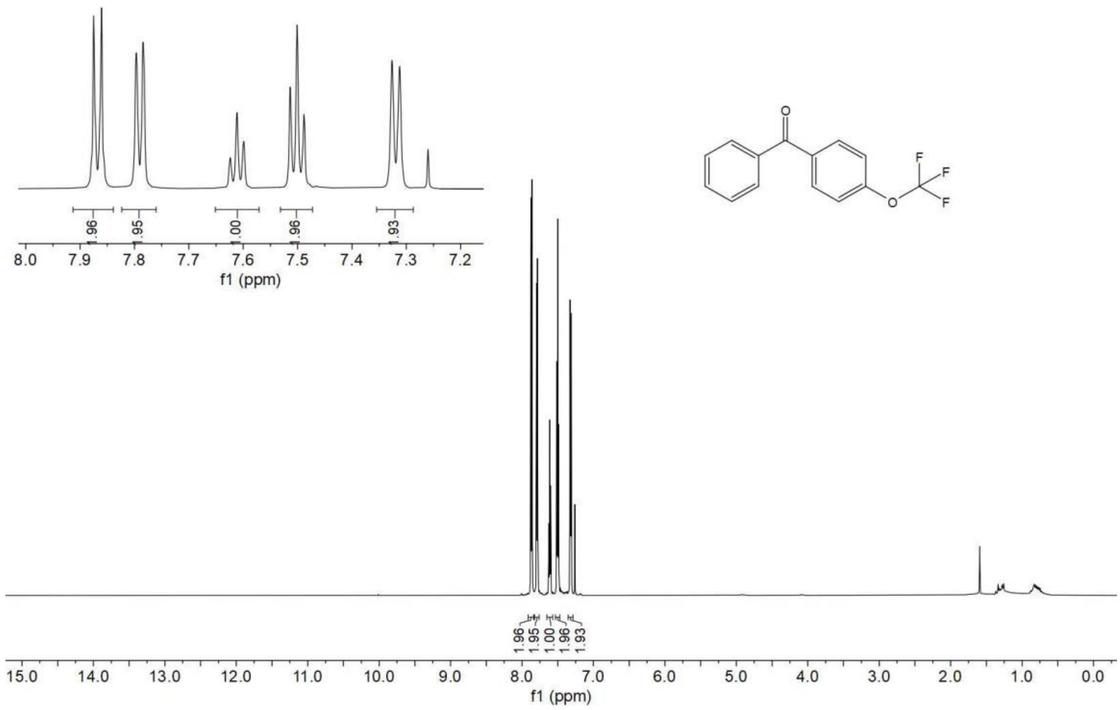

^{13}C NMR (**c43**)

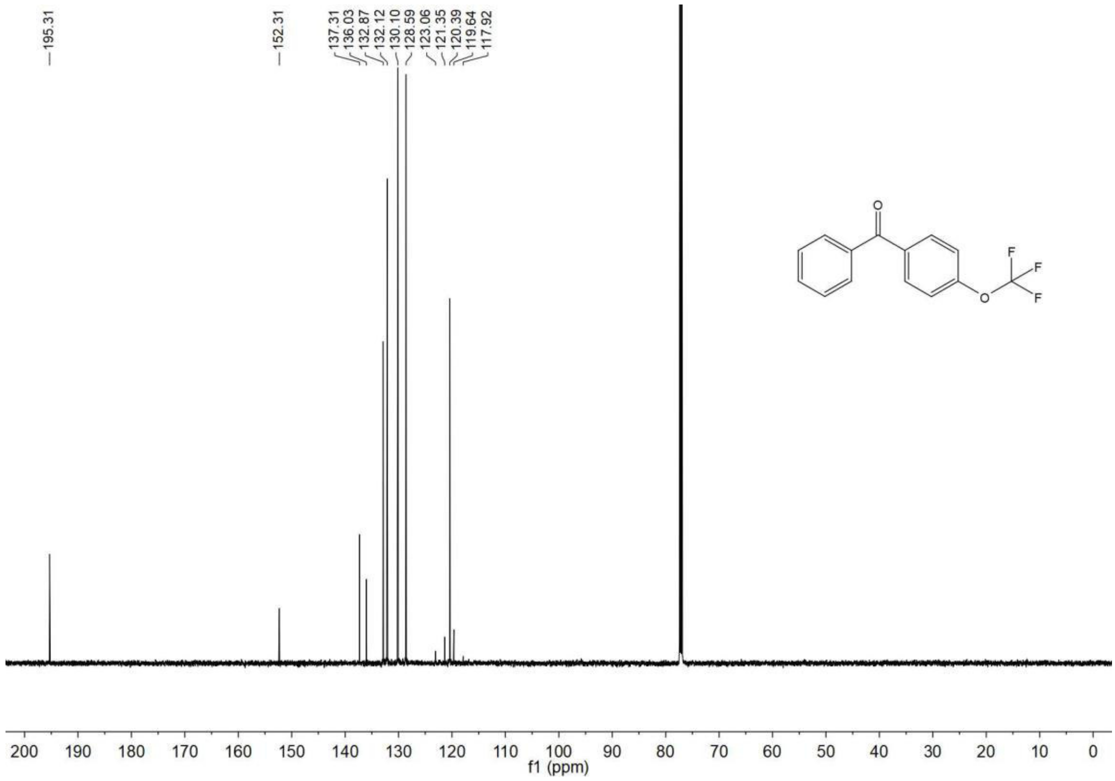

^{19}F NMR (**c43**)

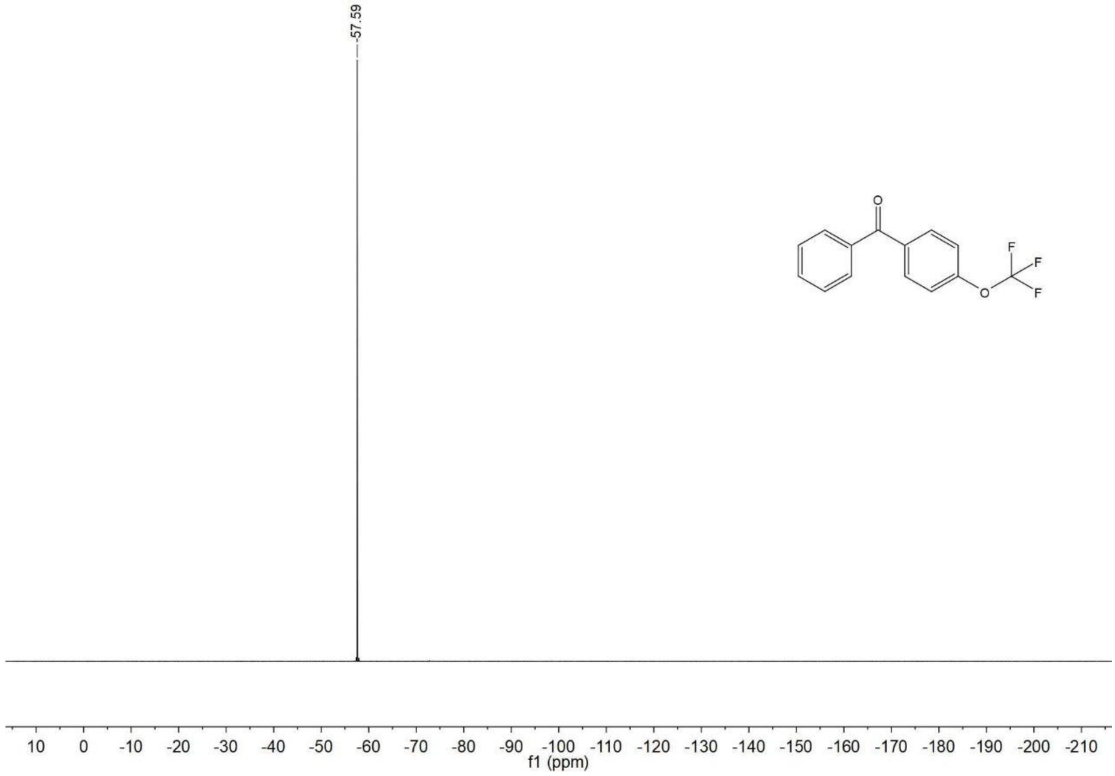

-57.59
10
0
-10
-20
-30
-40
-50
-60
-70
-80
-90
-100
-110
-120
-130
-140
-150
-160
-170
-180
-190
-200
-210
f1 (ppm)
O
F
F
F
O

^{1}H NMR (**c44**)

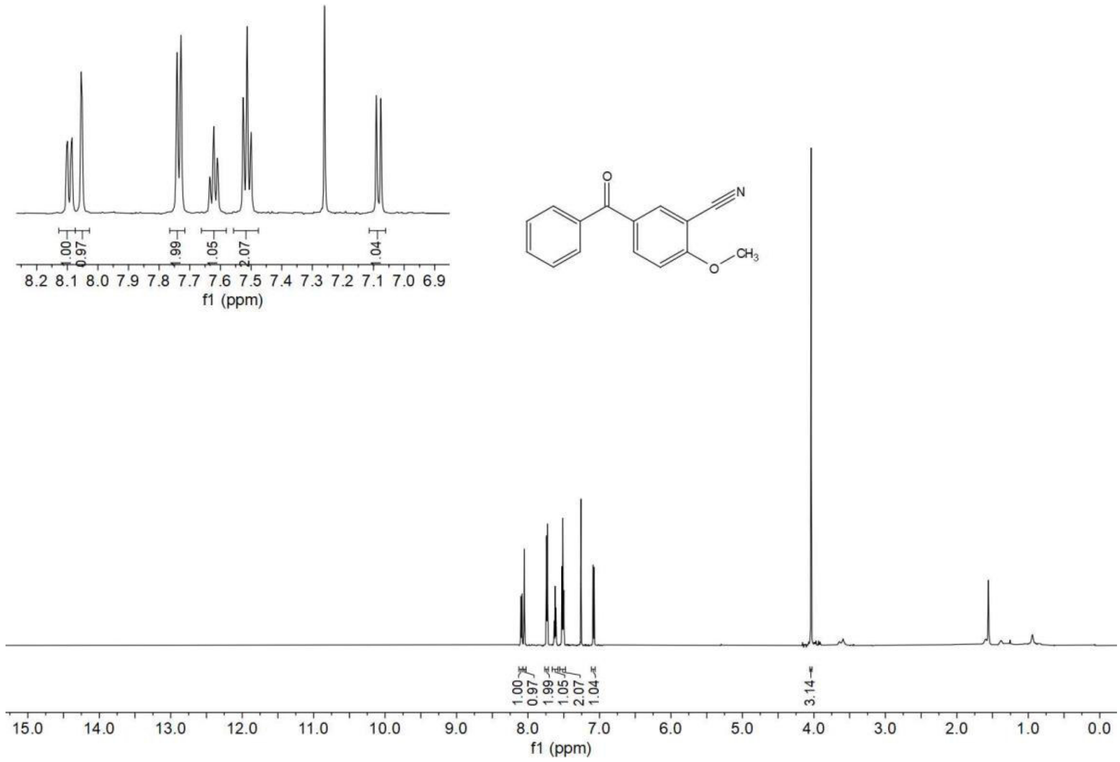

^{13}C NMR (**c44**)

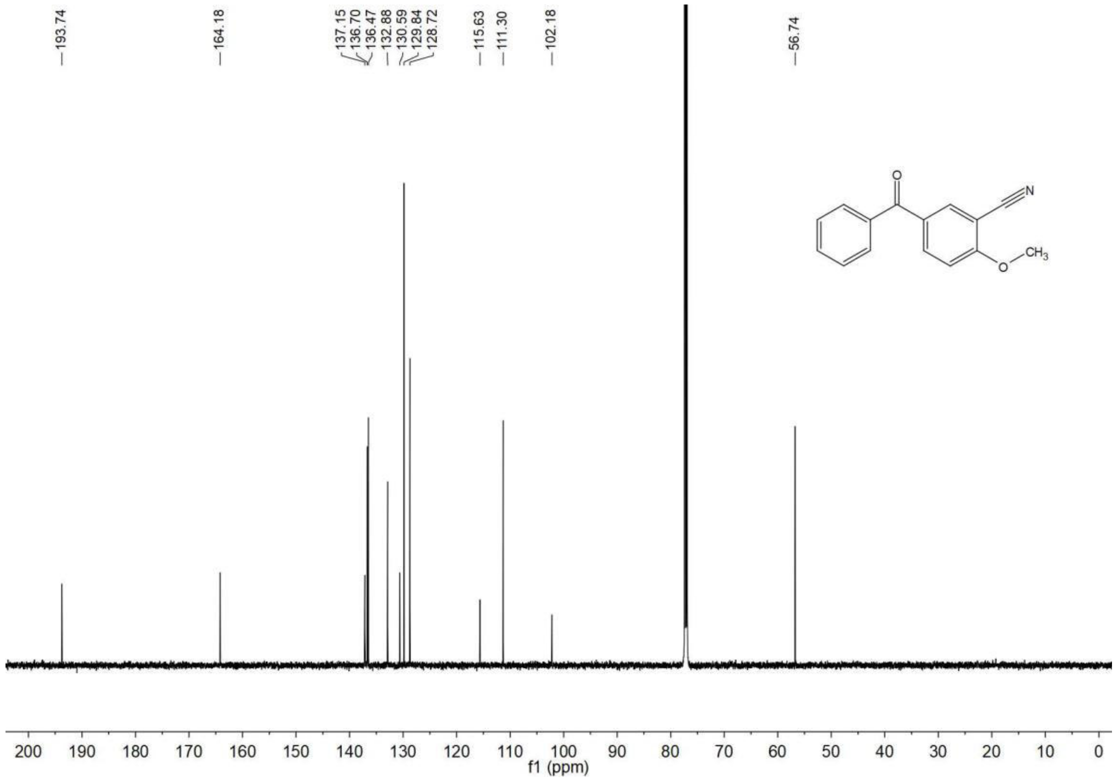

^{1}H NMR (**c45**)

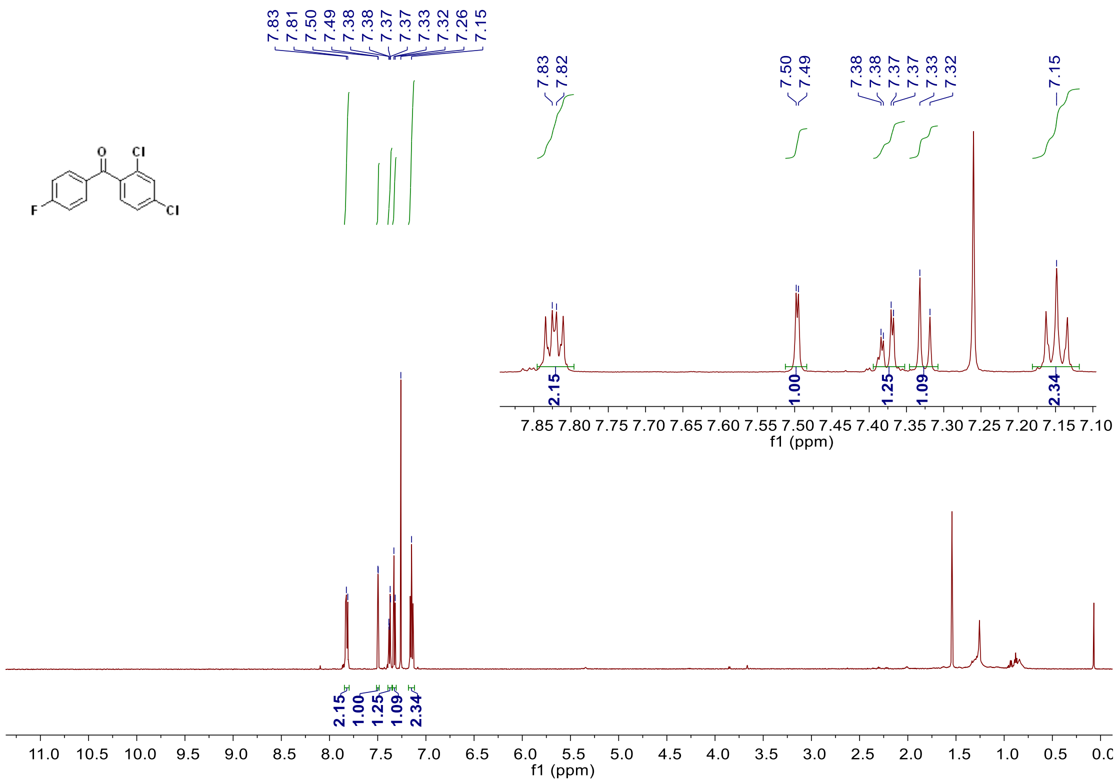

^{13}C NMR (**c45**)

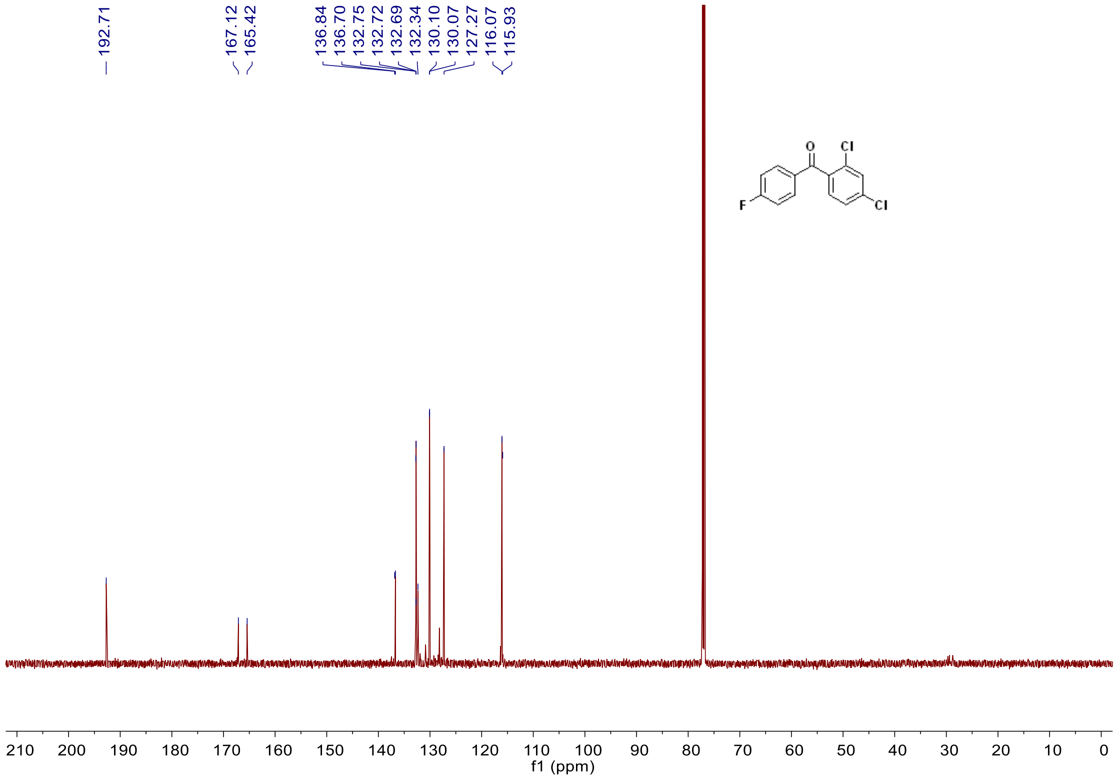

^{19}F NMR (**c45**)

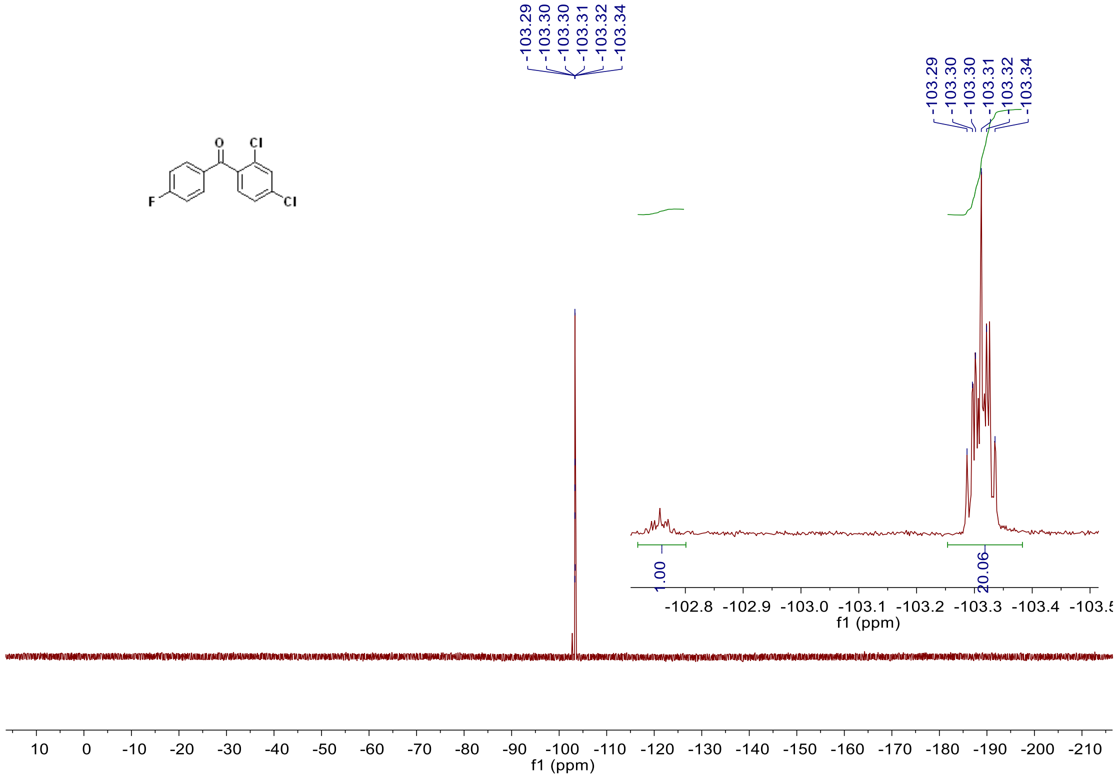

^{1}H NMR (**c46**)

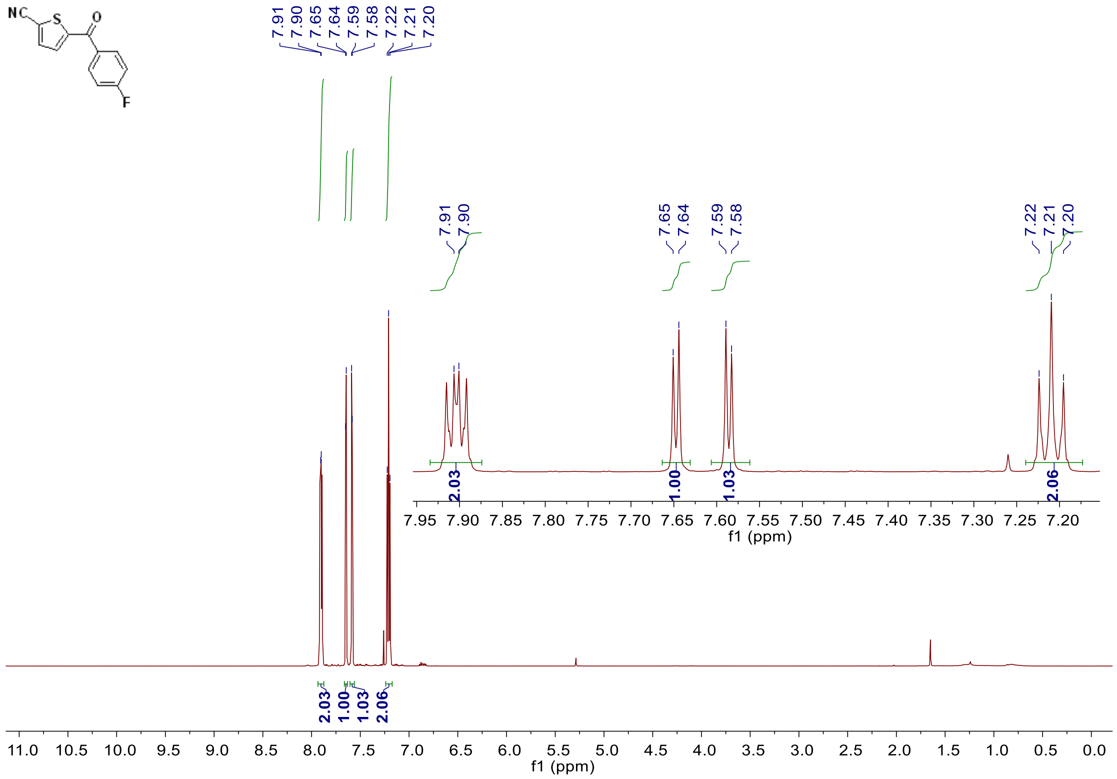

^{13}C NMR (**c46**)

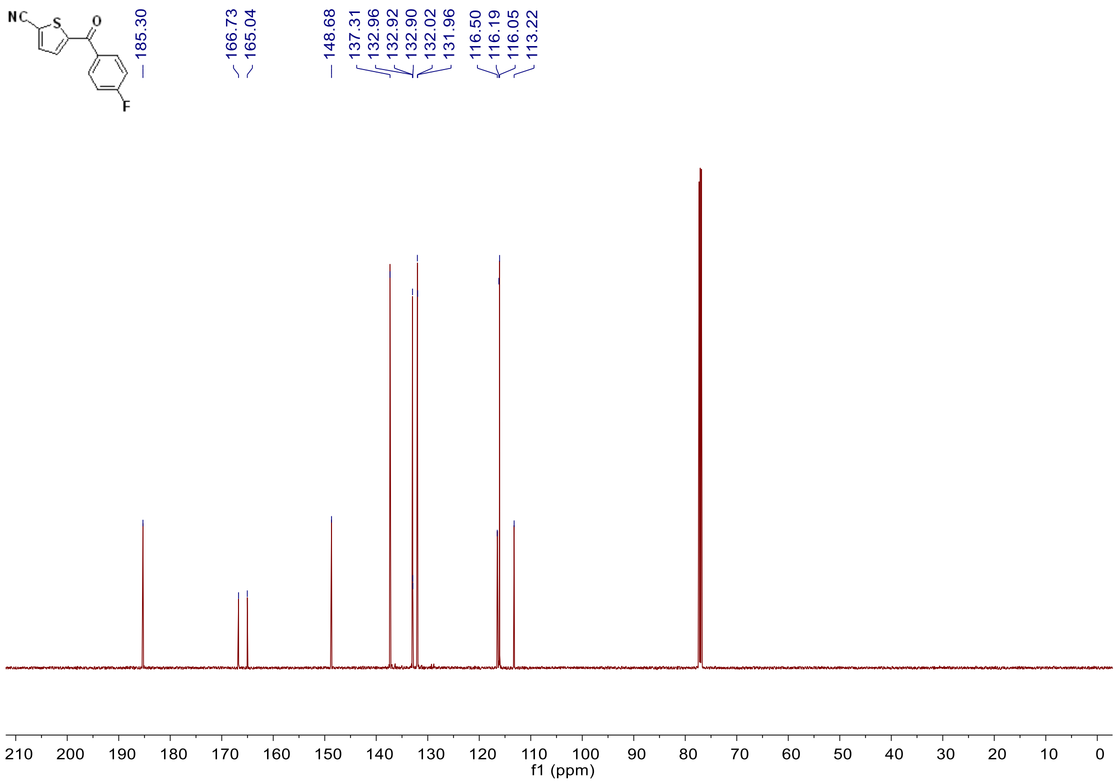

^{19}F NMR (**c46**)

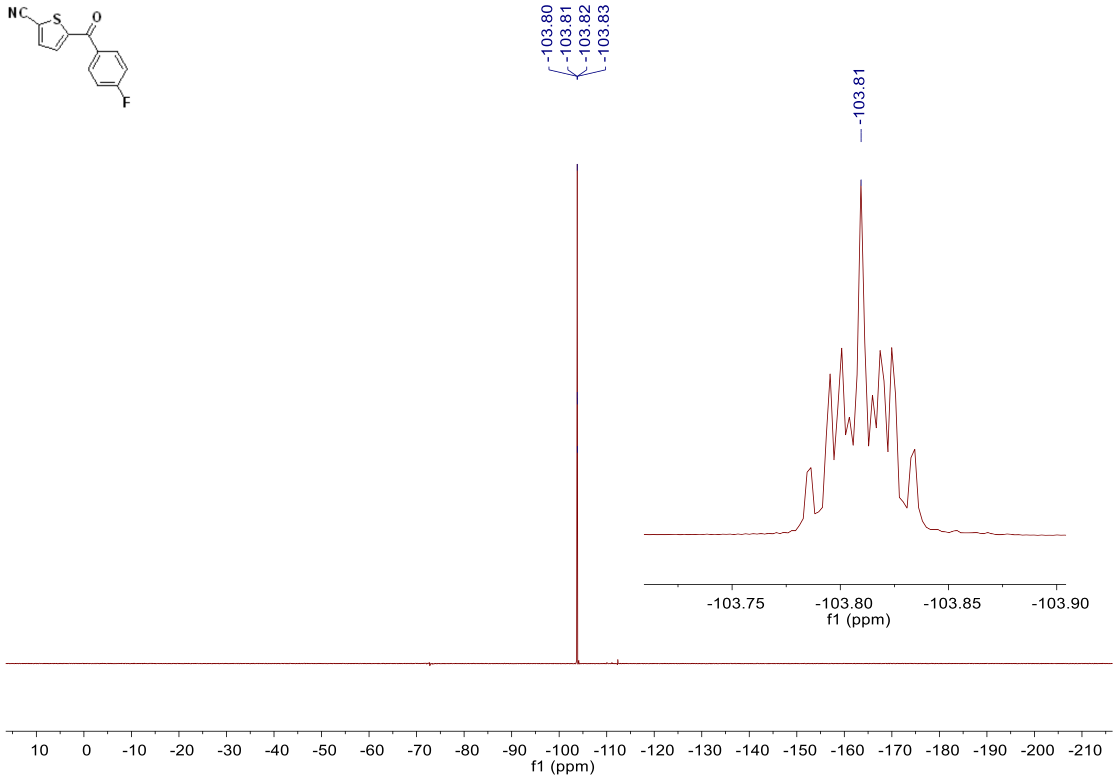

^{1}H NMR (**a47**)

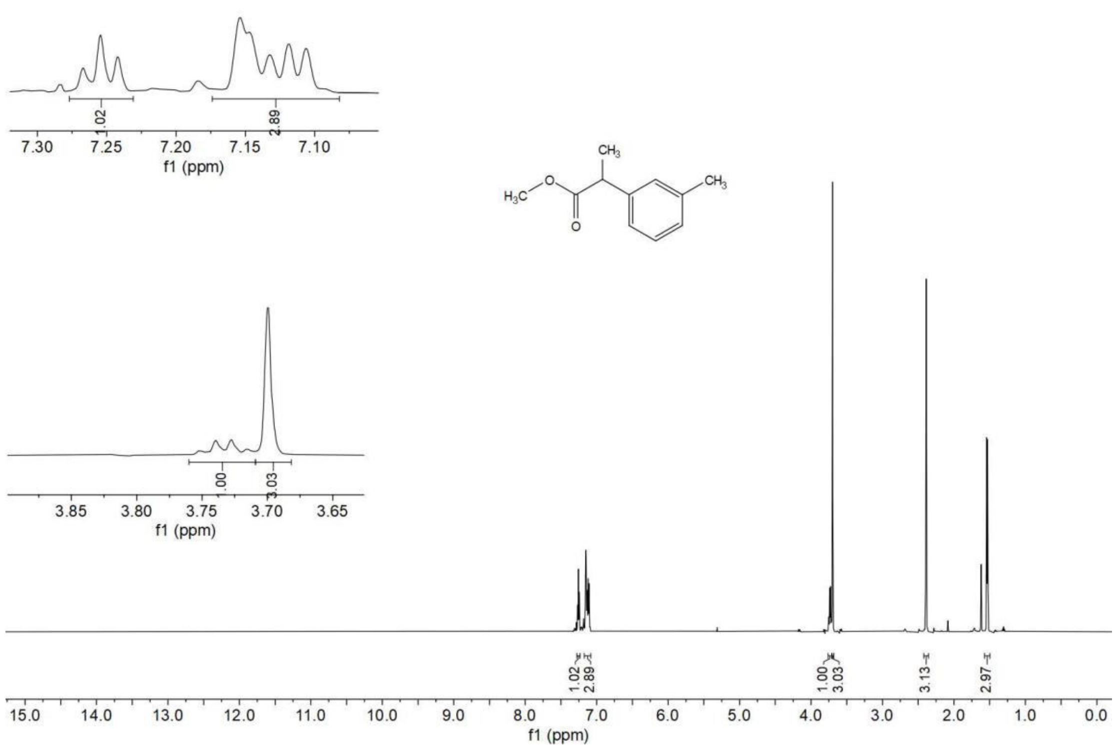

^{13}C NMR (**a47**)

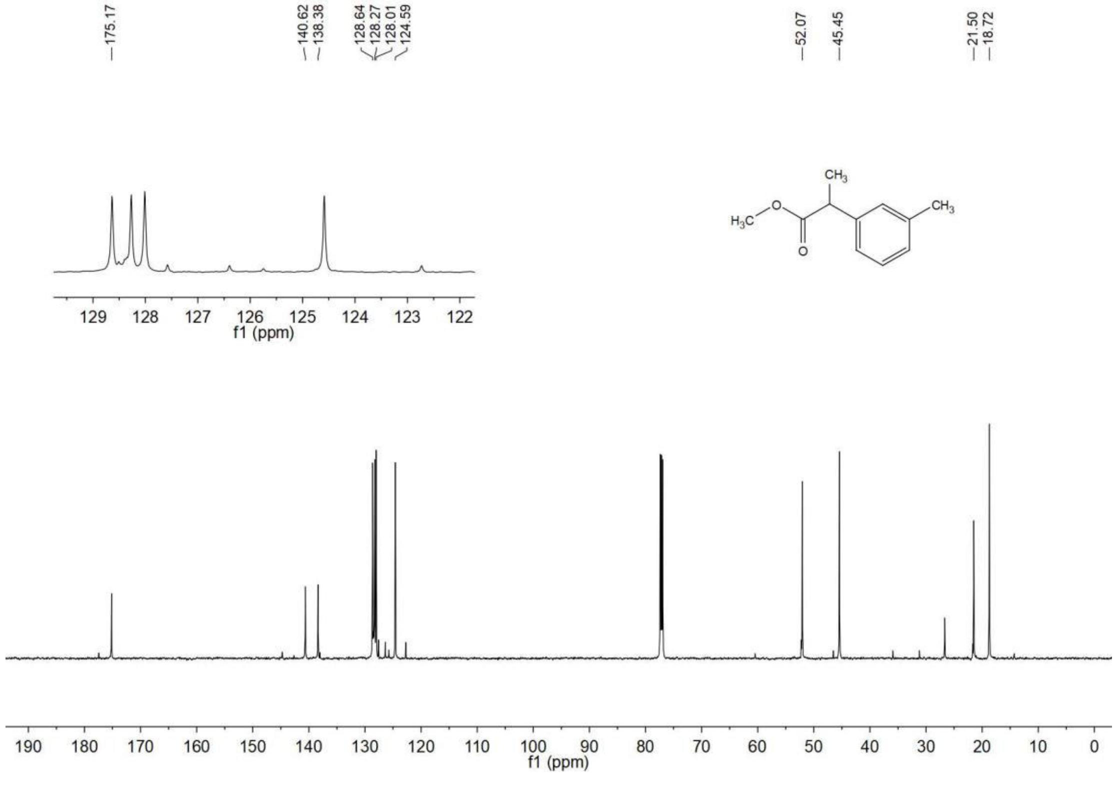

^{1}H NMR (**c47**)

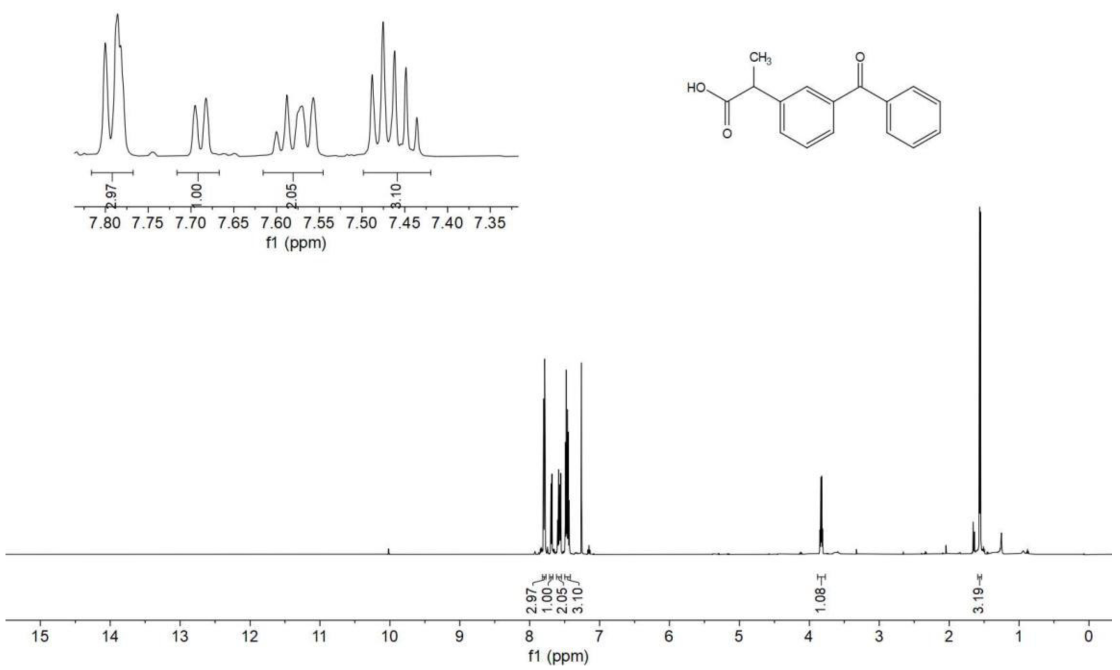

^{13}C NMR (**c47**)

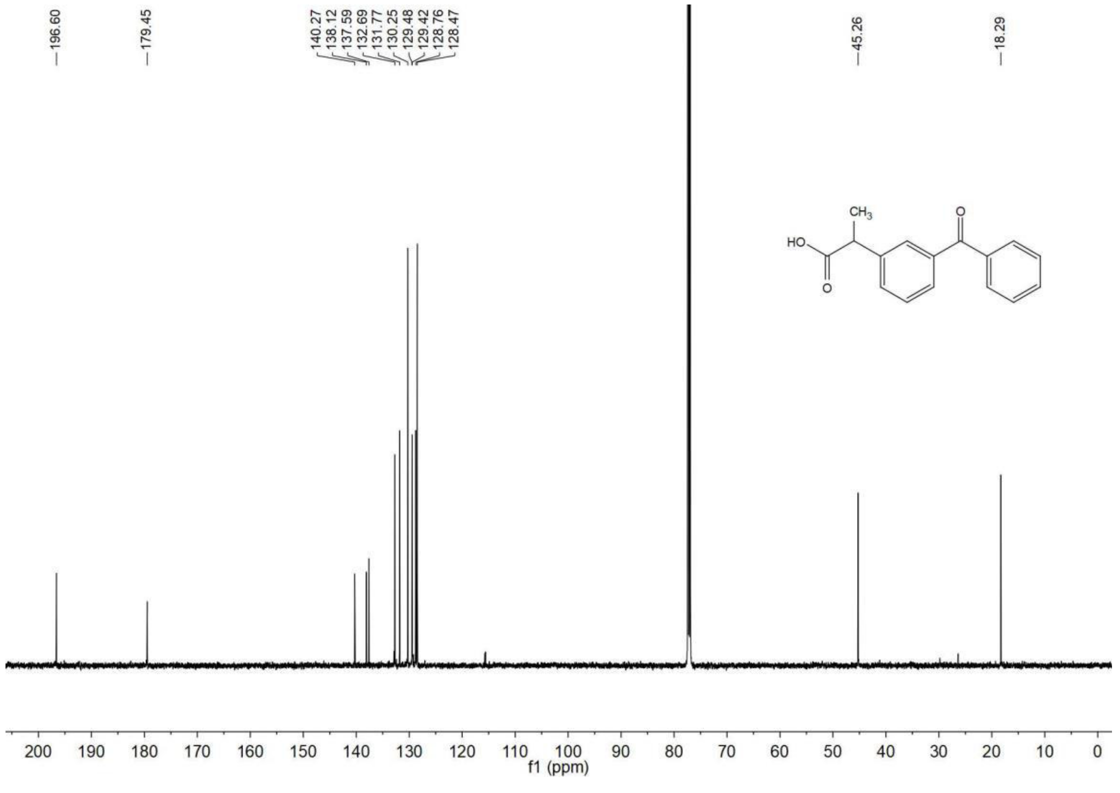

^{1}H NMR (**c48**)

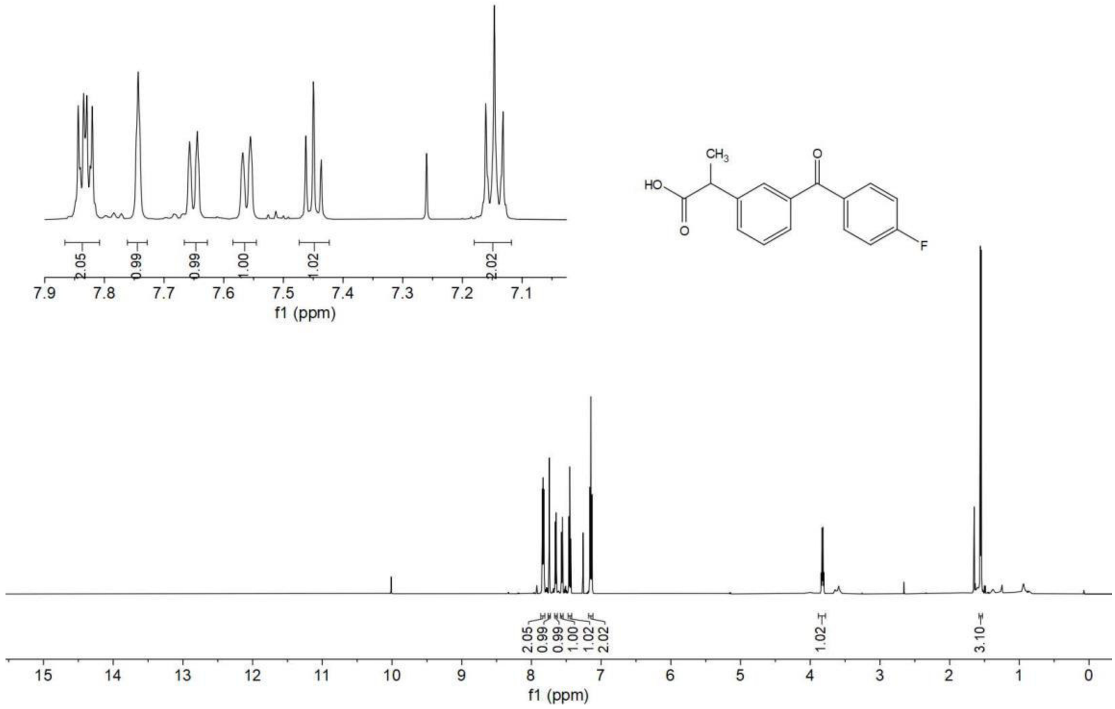

^{13}C NMR (**c48**)

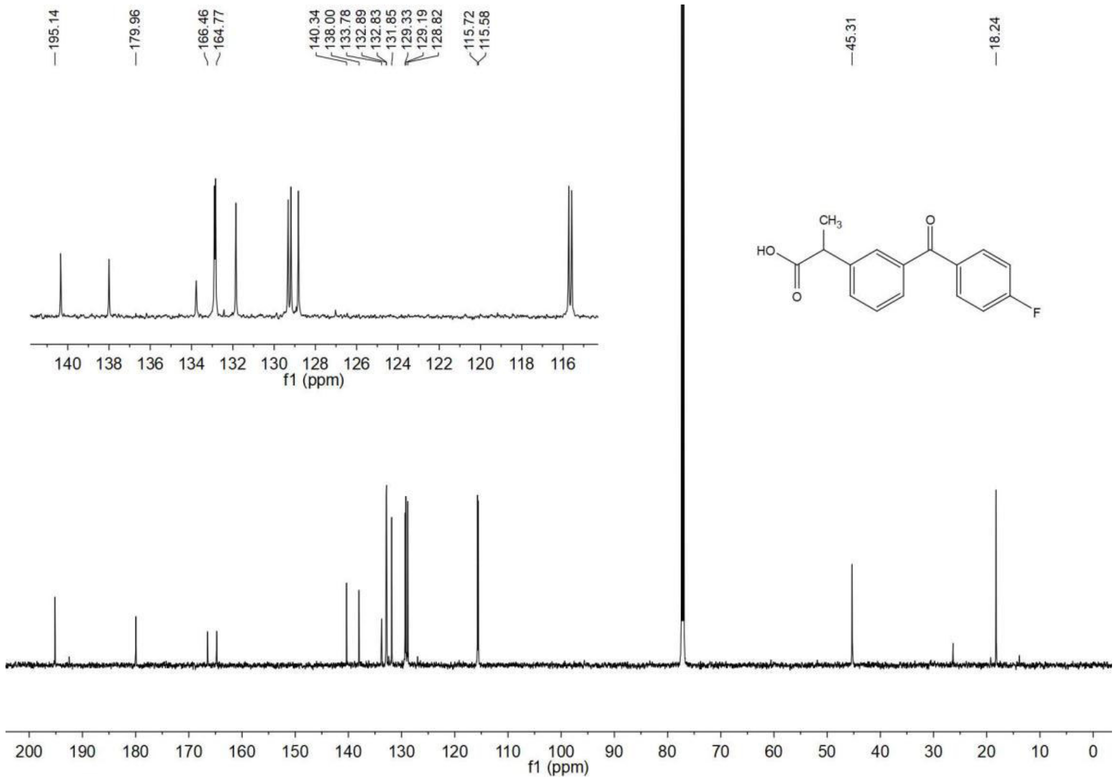

^{19}F NMR (**c48**)

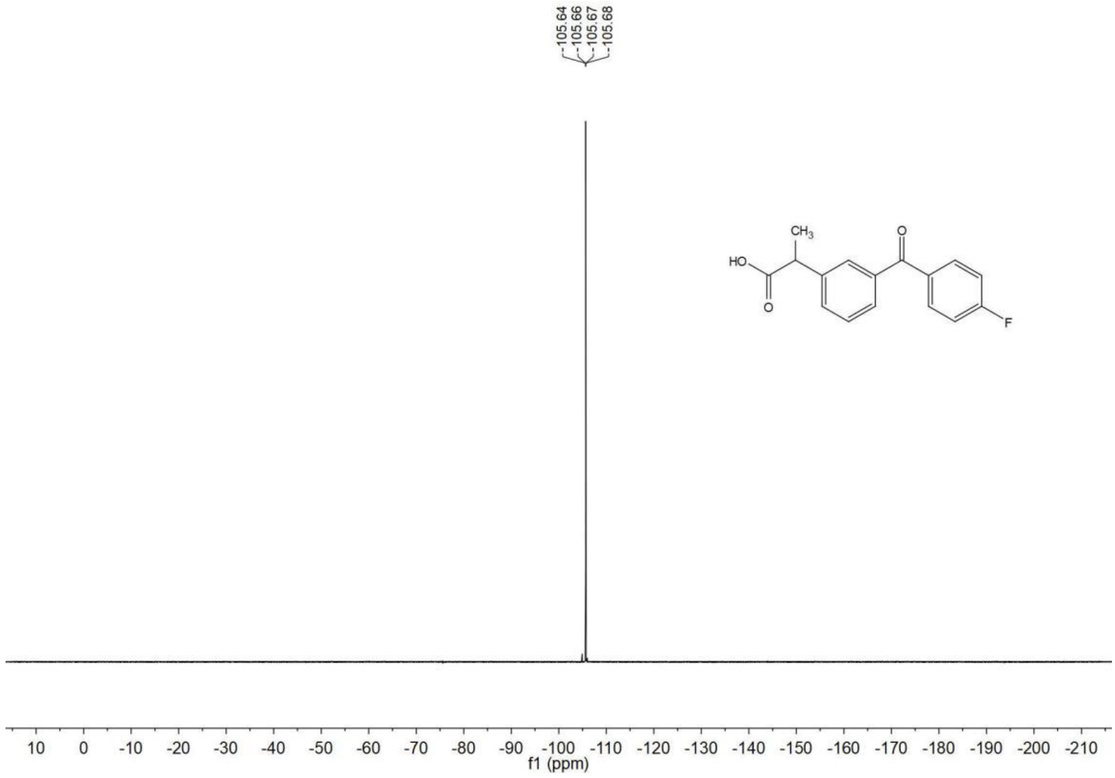
-105.64
-105.66
-105.67
-105.68
CH3
HO
O
O
F
10 0 -10 -20 -30 -40 -50 -60 -70 -80 -90 -100 -110 -120 -130 -140 -150 -160 -170 -180 -190 -200 -210
f1 (ppm)

^{1}H NMR (**c49**)

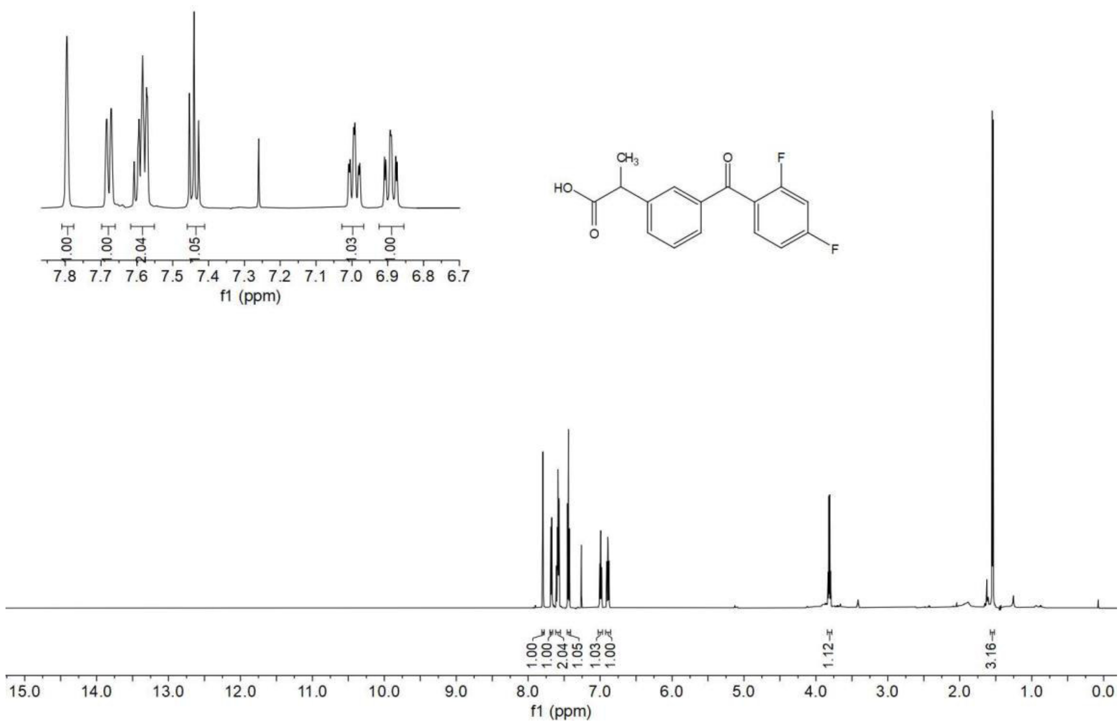

^{13}C NMR (**c49**)

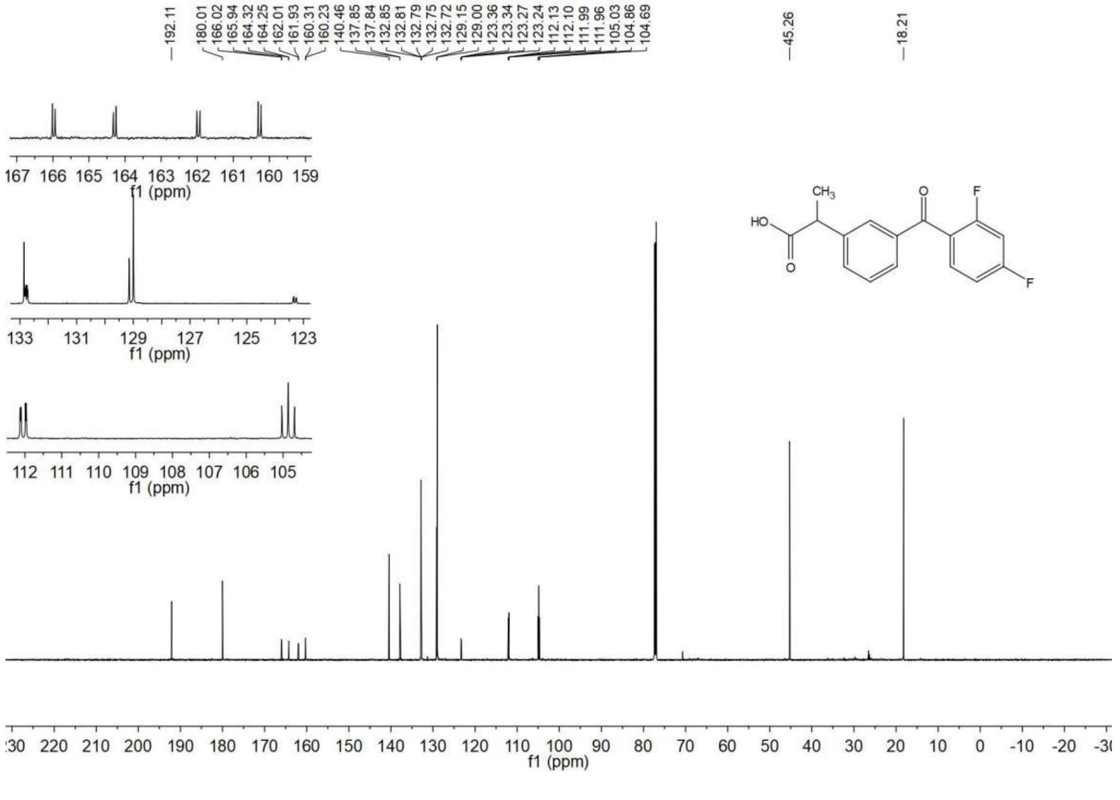

^{19}F NMR (**c49**)

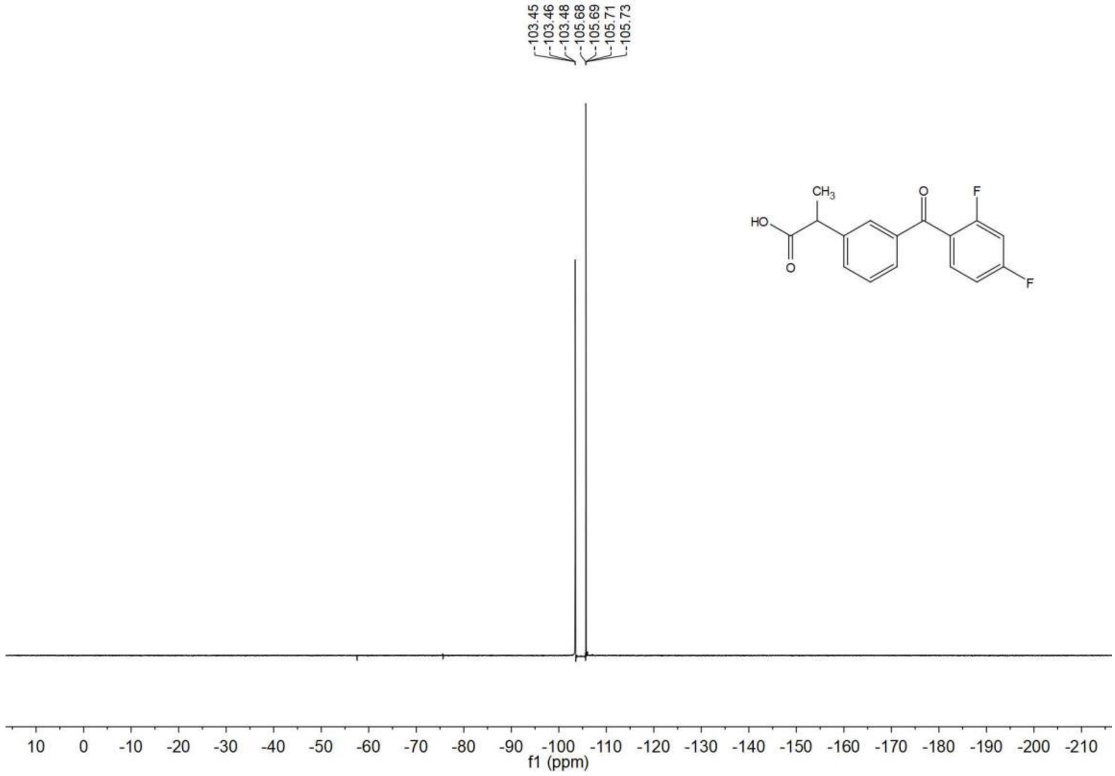
-103.45
-103.46
-103.48
-105.68
-105.69
-105.71
-105.73
CH3
O
F
HO
O
F
10 0 -10 -20 -30 -40 -50 -60 -70 -80 -90 -100 -110 -120 -130 -140 -150 -160 -170 -180 -190 -200 -210
f1 (ppm)

^{1}H NMR (**c50**)

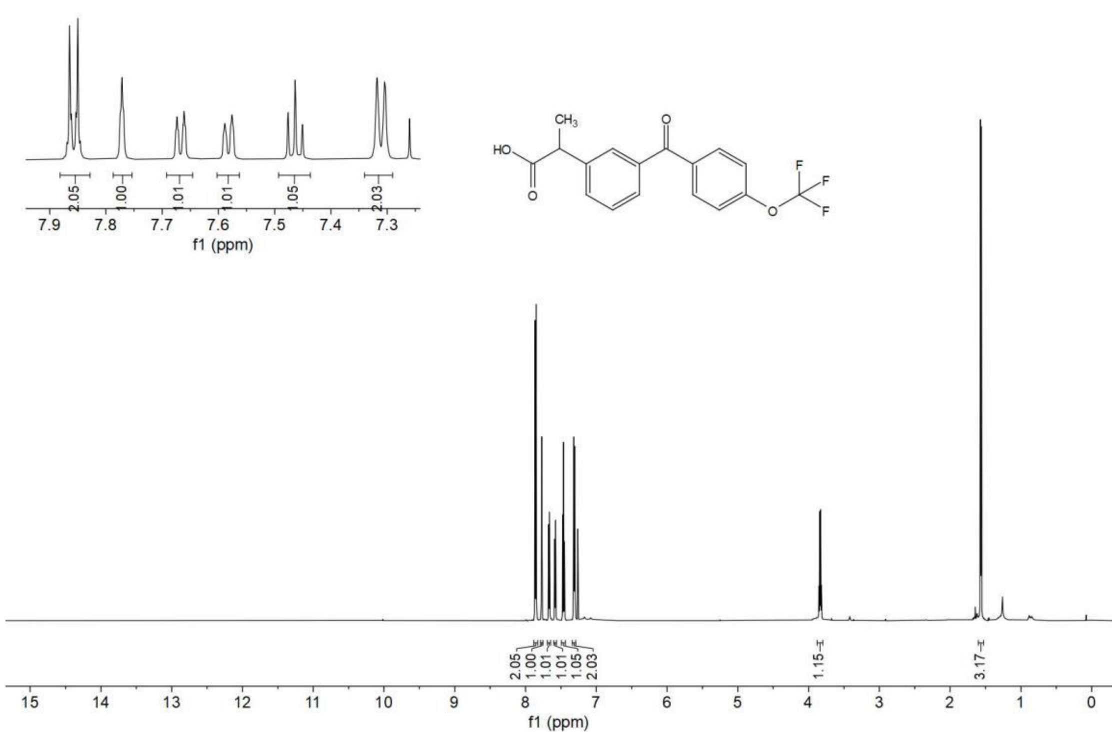

^{13}C NMR (**c50**)

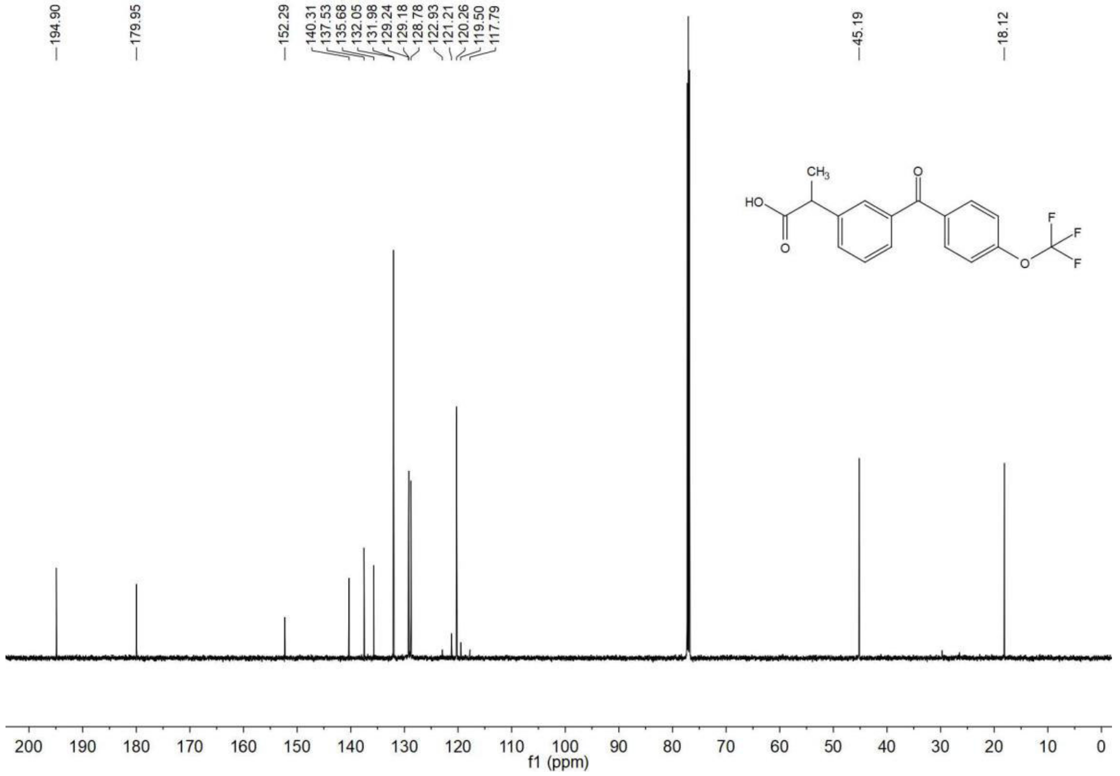

^{19}F NMR (**c50**)

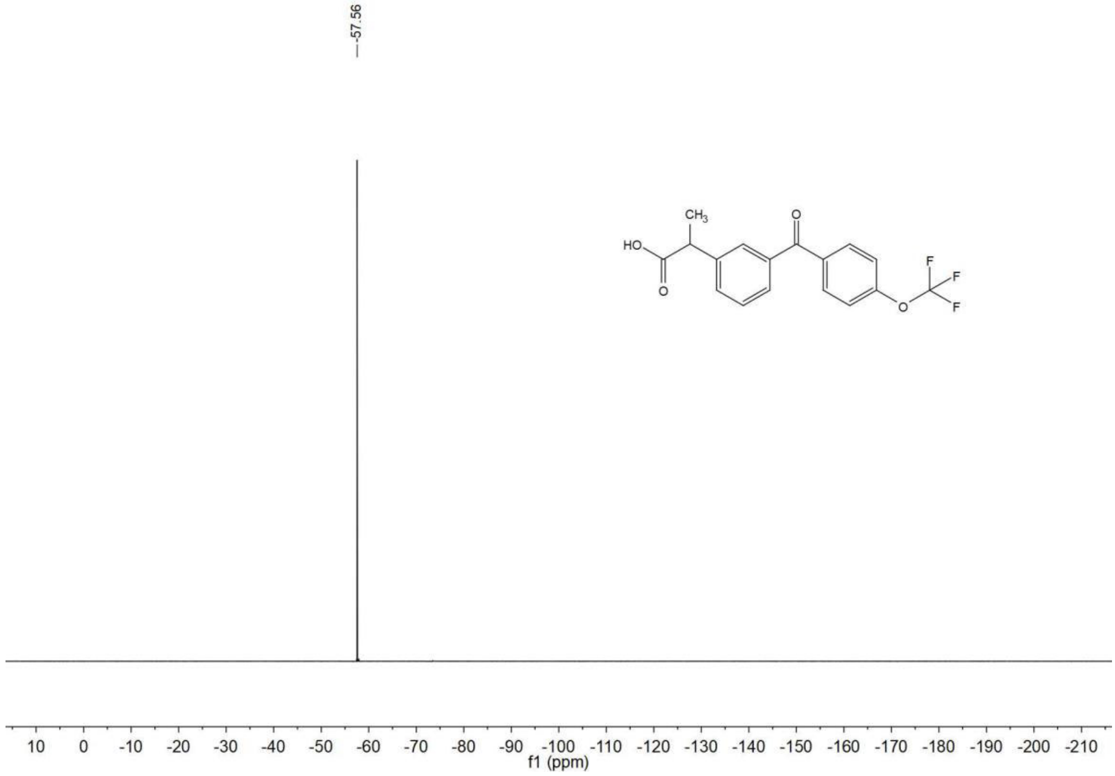

—-57.56
HO
O
CH3
O
F
F
F
O
10 0 -10 -20 -30 -40 -50 -60 -70 -80 -90 -100 -110 -120 -130 -140 -150 -160 -170 -180 -190 -200 -210
f1 (ppm)

^{1}H NMR (**f1**)

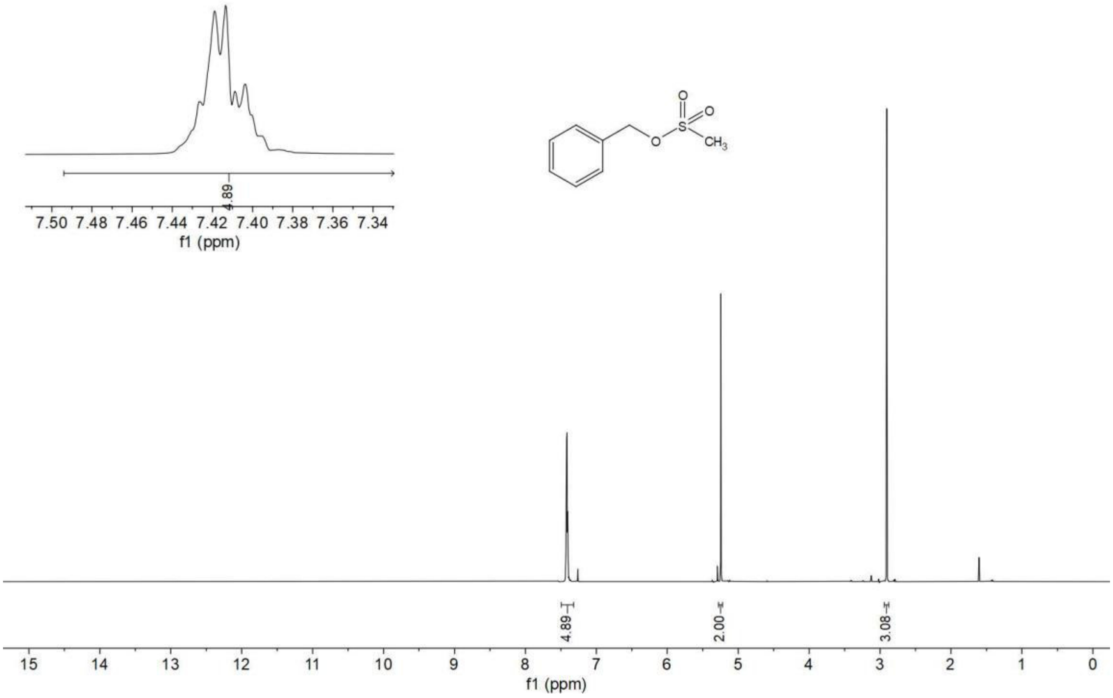

^{13}C NMR (**f1**)

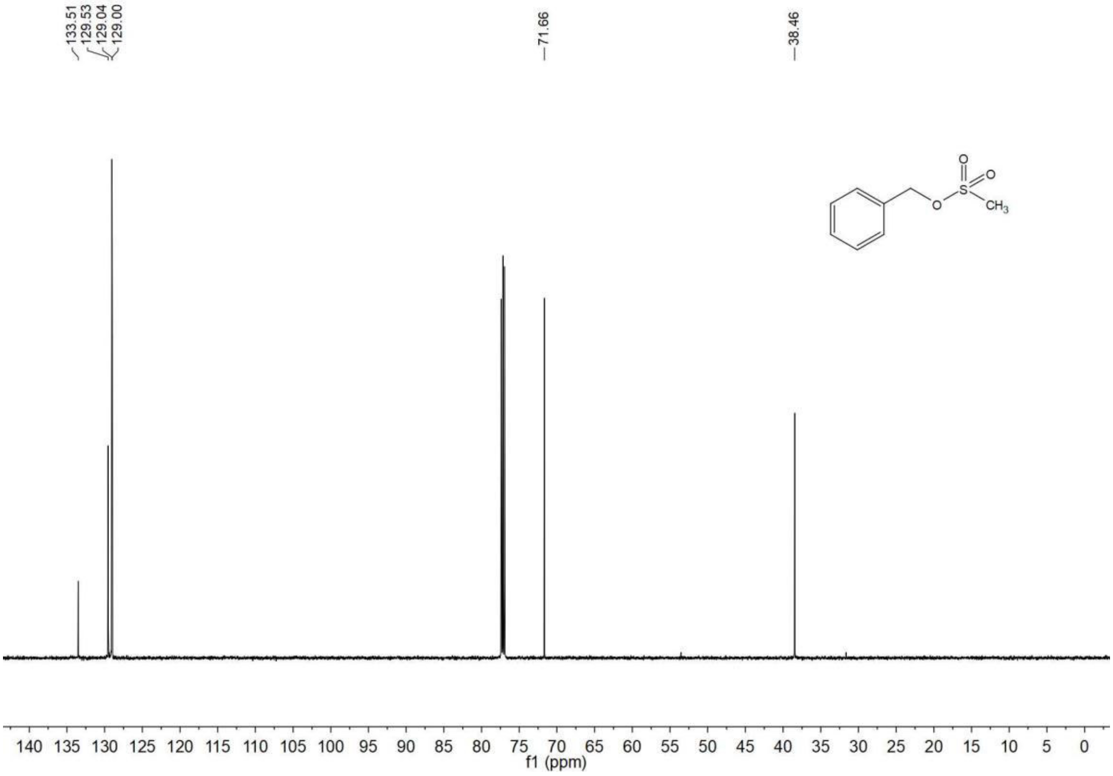

^{1}H NMR (**g1**)

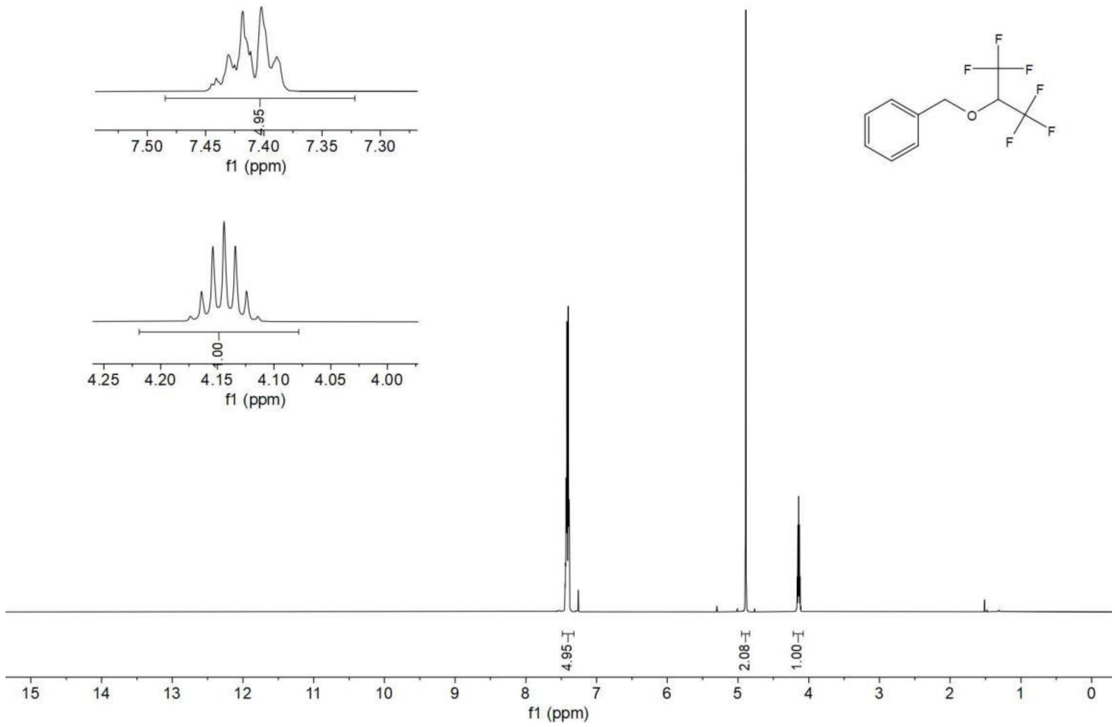

^{13}C NMR (**g1**)

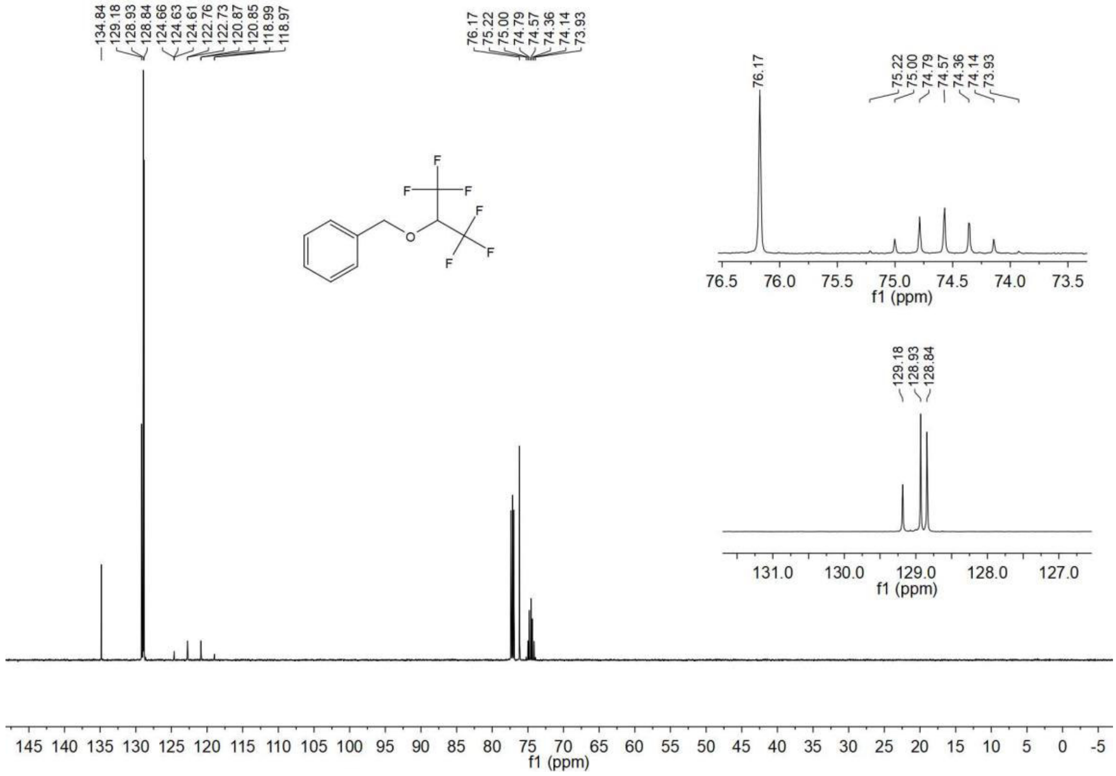

^{19}F NMR (**g1**)

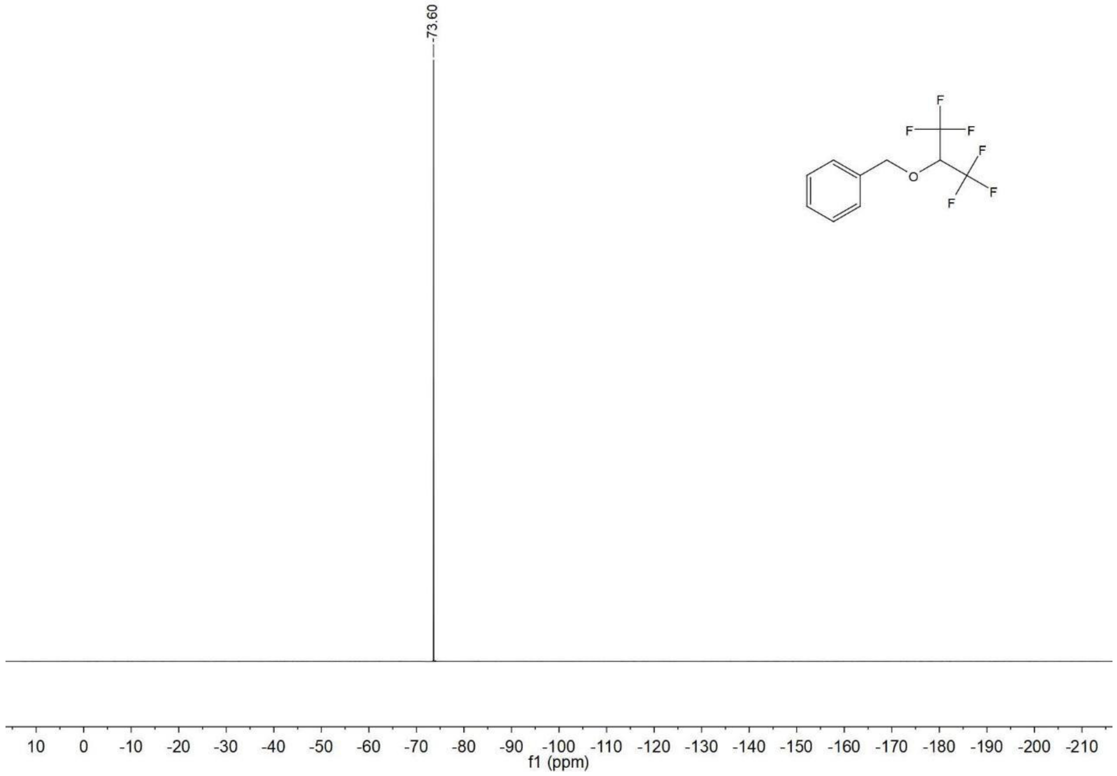

-73.60
10
0
-10
-20
-30
-40
-50
-60
-70
-80
-90
-100
-110
-120
-130
-140
-150
-160
-170
-180
-190
-200
-210
f1 (ppm)
